# Transcriptome-Wide Identification of Novel Imprinted Genes in Neonatal Mouse Brain

**DOI:** 10.1371/journal.pone.0003839

**Published:** 2008-12-04

**Authors:** Xu Wang, Qi Sun, Sean D. McGrath, Elaine R. Mardis, Paul D. Soloway, Andrew G. Clark

**Affiliations:** 1 Department of Molecular Biology & Genetics, Cornell University, Ithaca, New York, United States of America; 2 Computational Biology Service Unit, Life Sciences Core Laboratories Center, Cornell University, Ithaca, New York, United States of America; 3 The Genome Center at Washington University, Washington University School of Medicine, St. Louis, Missouri, United States of America; 4 Division of Nutritional Sciences, College of Agriculture and Life Sciences, Cornell University, Ithaca, New York, United States of America; University of Cambridge, United Kingdom

## Abstract

Imprinted genes display differential allelic expression in a manner that depends on the sex of the transmitting parent. The degree of imprinting is often tissue-specific and/or developmental stage-specific, and may be altered in some diseases including cancer. Here we applied Illumina/Solexa sequencing of the transcriptomes of reciprocal F1 mouse neonatal brains and identified 26 genes with parent-of-origin dependent differential allelic expression. Allele-specific Pyrosequencing verified 17 of them, including three novel imprinted genes. The known and novel imprinted genes all are found in proximity to previously reported differentially methylated regions (DMRs). Ten genes known to be imprinted in placenta had sufficient expression levels to attain a read depth that provided statistical power to detect imprinting, and yet all were consistent with non-imprinting in our transcript count data for neonatal brain. Three closely linked and reciprocally imprinted gene pairs were also discovered, and their pattern of expression suggests transcriptional interference. Despite the coverage of more than 5000 genes, this scan only identified three novel imprinted refseq genes in neonatal brain, suggesting that this tissue is nearly exhaustively characterized. This approach has the potential to yield an complete catalog of imprinted genes after application to multiple tissues and developmental stages, shedding light on the mechanism, bioinformatic prediction, and evolution of imprinted genes and diseases associated with genomic imprinting.

## Introduction

To date, 98 genes have been shown to undergo genomic imprinting in mouse, and 56 genes are imprinted in humans, with an overlapping set of 38 genes imprinted in both species [1]. For neither species is the list of imprinted genes complete. Genome-wide bioinformatic predictions face the challenge of a high false positive rate, mostly because the training set of known imprinted genes is small, and we do not know all the signals driving tissue- and time-specificity of imprinting [2], [3]. Attempts at exhaustive scans for imprinted genes in humans have encountered several drawbacks, including the challenge of using the most appropriate tissue and developmental stage, a problem exacerbated by reliance on lymphoblastoid cell lines (LCLs) [4]. Many imprinted genes show tissue- and developmental stage-specific expression, and many are expressed and imprinted only in specific stages of brain development. Human studies also face the challenge of a low number of informative heterozygous SNPs, so that allele-specific assays are useful for only a small subset of individuals. Hence, pedigree information is needed to distinguish genomic imprinting from stochastic monoallelic expression [5], [6]. These factors greatly amplify the effort and cost needed for a transcriptome-wide scan for imprinted genes in humans. By contrast, large-scale mouse studies have used uniparental disomy [7]–[12] to detect parent-of-origin effects. While this approach has led to the discovery of many imprinted genes, and to the refinement of phenotypic analysis of the consequences of disruptions in imprinting, not all genomic regions are covered by uniparental disomies, and there is a risk that such aberrant genome configurations may distort expression patterns. Microarray-based approaches using allele-specific probes can only detect nearly “all-or-none” imprinting with confidence, because quantitative differences between maternal vs. paternal allelic expression have high error due to the cross hybridization of the perfect-match and mismatch probes [13], [14]. In fact, genomic imprinting may occur as a continuum from complete uniparental expression to a slight but significant bias in the parental allele that is expressed, and a technology that could reliably detect quantitative differences in allele-specific expression at a transcriptome scale would greatly accelerate imprinting research.

## Results

### Illumina sequencing results and SNP coverage

Short-read sequencing (*e.g*. Illumina/Solexa sequencing) of transcripts provides many advantages in imprinting studies by providing a large number of sequence tags that allow simple counting of transcripts encoded by the two transmitted parental alleles. In this study, we performed quantitative assessments of genomic imprinting in transcripts from reciprocal cross progeny of the AKR/J and PWD/PhJ mouse strains. Total RNA was extracted from postnatal day 2 (P2) F1 female mouse whole brains. One run of Illumina sequencing was done for each F1 female brain cDNA sample. We obtained 1072.63 Mbp of sequence data from the PWD x AKR cross (listing female strain first) and 1136.35 Mbp from AKR x PWD in 32 bp reads with high quality (Figure S1.1). On average, 27.74% of the reads were aligned to the NCBI RefSeq mouse genome database. Sequence heterogeneity between alleles was great enough to produce poor performance by ELAND in mapping reads to the genome, so this mapping was performed with the NCBI BLAST program (Table S1.1). Altogether, 33,519,739 and 35,510,887 reads were aligned to the RefSeq database in the respective reciprocal crosses. The sequences covered 15,491 RefSeq genes with at least one perfectly matching Illumina read in each of the two reciprocal crosses. Within these genes, we identified 814,360 and 884,828 reads spanning Perlegen SNPs for the two respective reciprocal crosses [15]. After quality control filtering (Table S1.2), 320,804 and 327,451 high quality SNP-containing reads remained, allowing identification of parent-of-origin of each read (see Methods for more details). 5,533 RefSeq genes (5,076 unique Entrez genes) were covered in our study with a total SNP count of four or more in both reciprocal crosses (Table S1.3). From the mouse Brain EST Database, among the 5,500 cDNA clones of polyA-containing 3′-end EST sequences in P4 cerebellum, 3,500 are distinct species [16]. This contrasts with a recent SAGE study of P30 mouse brain, where the number of matched GenBank transcripts with copy number five or more per cell was 4,161 [17], but those data lacked the allele-specific identification. Based on this information, we could query the imprinting status of nearly all currently known transcribed genes with detectable expression in mouse neonatal brain with an informative number of counts.

### Detecting genomic imprinting

**Figure 1:**
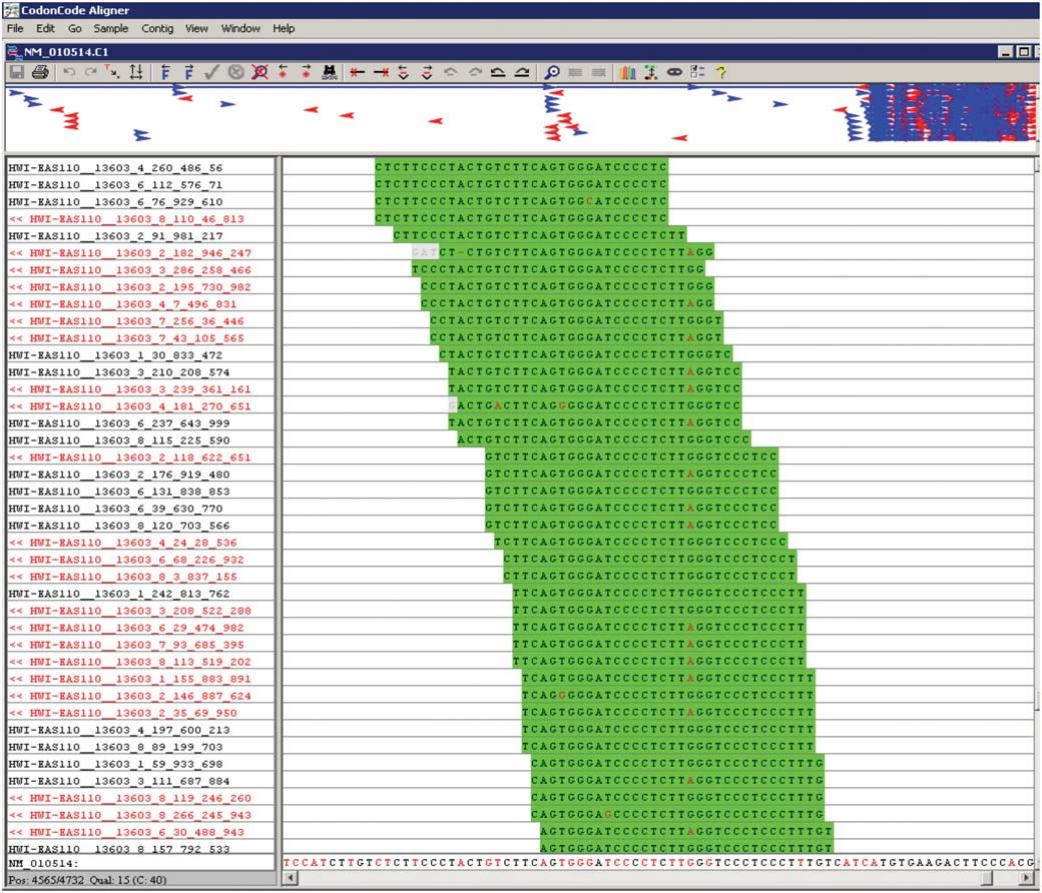
Alignment of Illumina sequence reads for *Igf2* transcript. The top panel is the summary window or all 1,253 cDNA reads that mapped to the 4,038 bp *Igf2* transcript (NM_010514). The blue arrows represent the sense reads and the red arrows represent antisense reads. From the figure, most of the reads were aligned to the 1 kb region near the 3′-end of the transcript. The bottom left panel gives the Illumina read names, and the bottom right gives the sequence alignment. Sense reads are printed in black font and the antisense reads are in red font. There are many overlapping 32-bp reads aligned uniquely to the transcript, with a quality score for each nucleotide. There is a SNP (A/G) located in the middle. By directly counting the number of reference and alternative nucleotides at the SNP, we were able to quantify the relative expression level of the two parental alleles.

The relative expression level of the two parental alleles was quantified from the counts of the AKR and PWD SNP alleles in the Illumina read data (Figure 1). We define *p_1_* to be the percentage of counts from AKR allele in PWD x AKR cross, and *p_2_* as the percentage of counts from AKR allele in AKR x PWD cross (Table S1.4). We identify a gene as a paternally expressed candidate imprinted gene if *p_1_* is significantly different from *p_2_* and where *p_1_*>0.5 and *p_2_*<0.5 (and, for maternally expressed genes, *p_1_*<0.5, and *p_2_*>0.5) (Table S1.5). The Storer-Kim test for two independent binomials [18], [19] was used to test the significance of the difference between the two binomial parameters, *p_1_* and *p_2,_* for each gene covered in the study [18]. *q*-values for each gene were calculated, and a false discovery rate cutoff of 0.05 was applied [20]. Using these criteria, we identified 13 paternally and 13 maternally expressed candidate imprinted genes with *p_1_*>0.65, *p_2_*<0.35 (*p_1_*<0.65, *p_2_*>0.35 for maternal genes) and *q*-value <0.05, respectively (Table 1).

**Table 1:** Candidate imprinted genes identified by biased allelic counts among transcripts.

Known IP genes	PWD x AKR		AKR x PWD		q-value	AKR percentage		Known status†	Verified status	Sig_SNPs (q<0.1)¶	Pyrosquencing	
	AKR*	PWD*	AKR*	PWD*		p1	p2				p1	p2
*Nnat* ^1^	1182	1	21	1853	0	99.9%	1.1%	IP	IP	4	100.0%	0.0%
*Snrpn* ^2^	898	1	1	19	0	99.9%	5.0%	IP	IP	1	100.0%	0.0%
*Snurf* ^2^	888	1	1	18	0	99.9%	5.3%	IP	IP	1	100.0%	0.0%
*Peg13* ^3^	168	0	6	74	0	100.0%	7.5%	NR	IP	3	98.8%	3.0%
*Nap1l5* ^3^	22	0	0	67	1.2E-19	100.0%	0.0%	NR	IP	1	100.0%	0.0%
*Inpp5f_v2* ^4^	41	3	14	80	1.4E-17	93.2%	14.9%	IP	IP	2	91.9%	7.8%
*Sgce* ^5^	9	0	0	54	2.0E-09	100.0%	0.0%	NR	IP	2	100.0%	1.5%
*Rasgrf1* ^6^	16	0	0	20	7.5E-09	100.0%	0.0%	IP	IP	3	100.0%	0.0%
*Impact* ^7^	15	6	8	83	1.2E-06	71.4%	8.8%	NR	IP	2	79.1%	19.8%
*Zrsr1* ^8^	11	0	1	14	6.7E-05	100.0%	6.7%	IP	IP	0	97.5%	0.4%
*Gtl2* ^9^	1	339	193	1	0	0.3%	99.5%	NR	IP	4	0.0%	100.0%
*H19* ^10^	2	14	61	1	5.8E-10	12.5%	98.4%	NR	IP	3	9.4%	100.0%
*Cdkn1c* ^11^	0	8	13	0	1.3E-04	0.0%	100.0%	NR	IP	1	3.6%	100.0%
*Commd1* ^12^	12	33	22	7	2.6E-03	26.7%	75.9%	IP	IP	0	41.2%	72.5%
**Novel IP genes**	**PWD x AKR**		**AKR x PWD**		**q-value**	**AKR percentage**		**Known status** †	**Verified status**	**Sig_SNPs (q<0.1)** ¶	**Pyrosquencing**	
	**AKR** *	**PWD** *	**AKR** *	**PWD** *		**p1**	**p2**				**p1**	**p2**
*Inpp5f*	359	19	89	1293	0	95.0%	6.4%	-	IP	7	83.2%	19.1%
*2410042D21Rik*	21	7	16	32	0.024	75.0%	33.3%	-	eQTL$	0	79.9%	83.6%
*BC043301*	8	0	3	9	0.042	100.0%	25.0%	-	eQTL	0	-	-
*1810044A24Rik*	7	20	25	5	1.1E-03	25.9%	83.3%	-	IP	1	20.6%	73.5%
*Gyg*	9	35	21	9	0.002	20.5%	70.0%	-	eQTL	1	40.9%	36.1%
*Ppfia2*	6	16	32	8	0.003	27.3%	80.0%	-	eQTL	0	-	-
*Prim1*	6	81	5	2	0.005	6.9%	71.4%	-	eQTL	1	-	-
*Asns*	24	60	27	14	0.005	28.6%	65.9%	-	eQTL	1	53.7%	56.3%
*2010012O05Rik*	6	17	41	16	0.010	26.1%	71.9%	-	eQTL	0	56.7%	57.6%
*Rgs17*	10	24	39	17	0.013	29.4%	69.6%	-	eQTL	0	54.5%	55.1%
*Pdcl*	5	13	61	23	0.018	27.8%	72.6%	-	eQTL	0	56.8%	58.9%
*Blcap*	6	13	15	2	0.025	31.6%	88.2%	-	IP	1	25.2%	73.7%

* : Counts of the AKR and PWD allele in the Illumina sequence data after filtering.

† : Reported imprinted status of the known imprinted genes in neonatal brain (IP: imprinted; NR: not reported).

¶ : Number of significant SNPs with *q*-value ≤0.10 for each gene.

$ : eQTL : Expression quantitative trait loci

**Figure 2:**
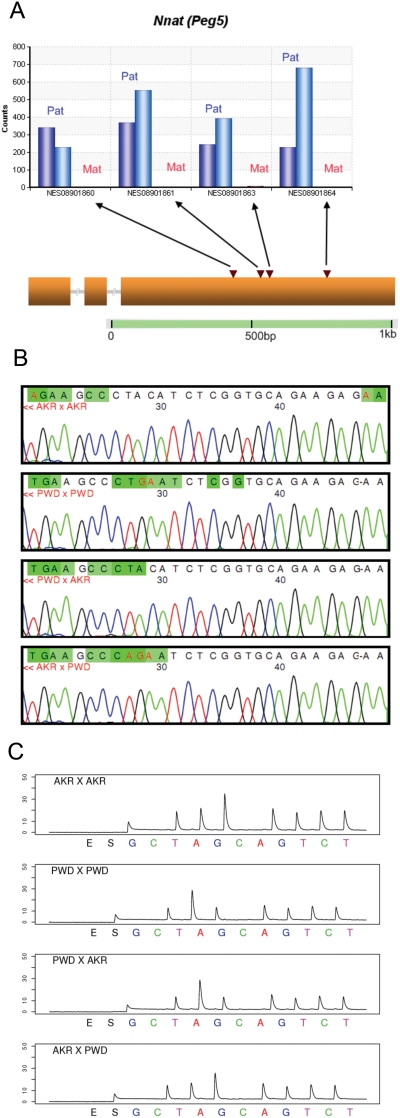
Verification for known imprinted gene *Nnat* (also known as *Peg5*). (A) Allele counts for Perlegen SNP NES08901860, NES08901861, NES08901863 and NES08901864. The blue bars (from left to right) represent the Illumina read counts from the paternal allele in PWD x AKR and AKR x PWD F1s respectively (maternal genotype listed first). The red bars represent the maternal allele Illumina read counts. (B) Sanger sequencing verification for Perlegen SNP NES08901861. We discovered an adjacent SNP position before NES08901861. The target sequence is GCCCT(AC/GA)ATCT. (C), Pyrosequencing verification for Perlegen SNP NES08901861. The target sequence is GCCCT(AC/GA)ATCT.

A total of 17 of the 26 candidate genes were verified to be imprinted by a combination of Sanger sequencing and Pyrosequencing. Of these, 14 are known imprinted genes. *Nnat* (*Peg5*), *Inpp5f_v2*, *Rasgrf1*, *Zrsr1* (*U2af1-rs1*), *Snrpn* and *Snurf* genes are known to be imprinted in mouse neonatal brain with paternal-only expression (Table 1; Supporting References S1) [21]–[25], and this was confirmed by both the Illumina sequence data and by Sanger sequencing and Pyrosequencing (Figures S1.2–S1.5). *Neuronatin* (*Nnat*), a gene on mouse chromosome 2, is known to be imprinted in mouse neonatal brain [21]. In our data, *Nnat* showed 100% paternal monoallelic expression, with a *q*-value of zero (Table 1). Four SNPs within the last exon of the gene were covered by the Illumina reads. All of them showed 100% paternal expression as scored in 3,057 observed paternal allele-bearing reads in both reciprocal F1s (Figure 2A), a result verified by Sanger sequencing (Figure 2C) and by Pyrosequencing (Figure 2C).

**Figure 3:**
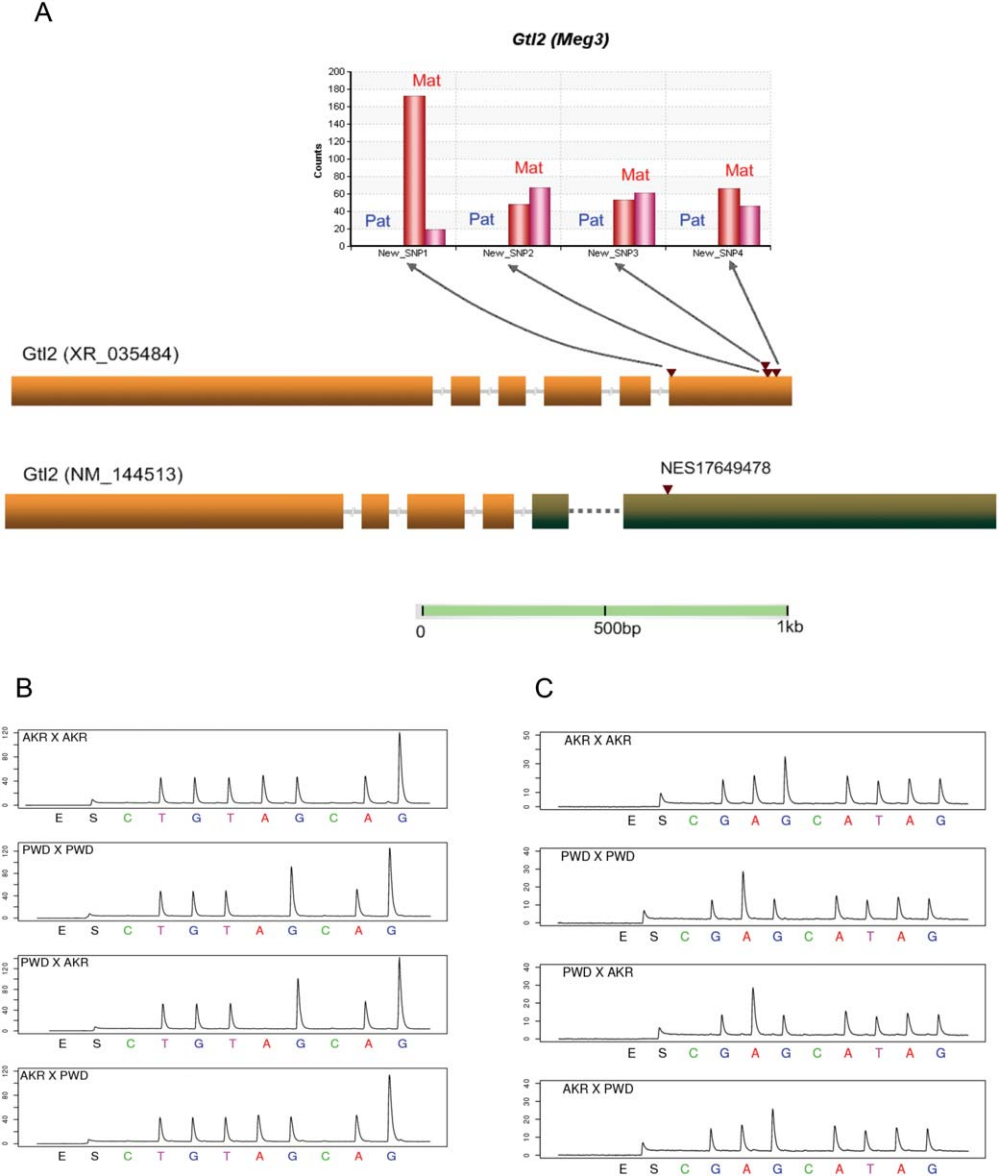
Verification for the known imprinted gene *Gtl2*. (A) Allele counts for the 4 new SNPs discovered by assembling the Solexa reads. The blue bars (from left to right) stand for the counts from the paternal allele in PWD x AKR and AKR x PWD F1s respectively. The red bars represent the maternal allele counts. Four novel SNPs were discovered in one *Gtl2* transcript (XR_035484), consistent with monoallelic expression from the maternal allele in both reciprocal crosses and confirmed by Pyrosequencing. Another splicing variant of *Gtl2*, NM_144513, previously was found by us to be imprinted using a custom Agilent allele-specific microarray (unpublished), with an 1,847-fold difference in probe intensity in PWD x AKR cross and 793-fold in the reciprocal cross. A Perlegen SNP (NES17649478) in NM_144513 but not XR_035484 was verified by Pyrosequencing. We conclude that both XR_035484 and NM_144513 are imprinted in the neonatal brain. (B) Pyrosequencing verification for novel SNP1 in *Gtl2*. The target sequence is TGT(A/G)GAGGGA. (C) Pyrosequencing verification for Perlegen SNP NES17649478. The target sequence is GA(A/G)GATAG.

The imprinting status of seven known imprinted genes have not been previously reported in neonatal brain, including the paternally expressed *Peg13*, *Sgce* and *Nap1l5* (Table 1; Figures S1.6–S1.8) [26], [27] and the maternally expressed *Gtl2* (*Meg3*), *Impact*, *H19* and *Cdkn1c* (*P57^KIP2^*) (Table 1; Figures S1.9–S1.11) [28]–[31]. Our data support their being imprinted in P2 neonatal brain (Table 1). *Gtl2* (also known as *Meg3*) is a non-coding RNA gene on mouse chromosome 12, and it is reported to be imprinted in mouse placenta [28]. Although the expression pattern of *Gtl2* has been determined in brain [32], [33], the imprinting status was not tested in neonatal brain. There is no Perlegen SNP covered in the Solexa data, but from the assembly of the Solexa reads, 4 novel SNPs were discovered and it is suggested that the *Gtl2* transcript (XR_035484) is expressed only from the maternal allele (Figure 3A). This is confirmed by Pyrosequencing (Figure 3B). Another splicing variant of *Gtl2*, NM_144513, was identified to be imprinted in our custom Agilent microarray survey of novel imprinted genes (A. Clark unpublished data), with 1,847-fold difference in probe intensity in PWD x AKR cross and 793-fold in the reciprocal cross. A Perlegen SNP (NES17649478) in NM_144513 but not XR_035484 was verified by Pyrosequencing (Figure 3C). The analysis shows unambiguously that both splice variants are imprinted. Careful examination of the *in situ* images of fetal brain of uniparental disomic mice are consistent with our findings and suggest that there is maternal expression only [34].

### Known and novel imprinted genes identified

We also discovered three novel imprinted genes by Illumina short-read sequencing, and verified by Sanger and Pyrosequencing. According to Choi et al. [22], *Inpp5f* is a splicing variant of the known imprinted gene *Inpp5f_v2*, sharing 4 exons and part of the last exon. There are seven SNPs covered in the sequence data for *Inpp5f*, with 2 of them shared by *Inpp5f_v2*. Since all seven SNPs show significant paternal-excess in expression, we conclude that *Inpp5f* is also imprinted in P2 neonatal brain (Figure S1.2). Formally, it is also possible that *Inpp5f* and *Inpp5f_v2* share the same 3′ end. Two CpG islands near the gene region were reported before [22]. CpG1 is not methylated and CpG2 is the DMR (Differentially Methylated Region) with only the paternal allele being methylated. Two previously reported non-imprinted genes, *1810044A24Rik* [35] and *Blcap* [36], are found to be predominantly maternally expressed novel imprinted genes in our sequence data (*q*-value 0.0011 and 0.025) and Pyrosequencing verified that they showed 80% expression from the maternal allele. The imprinting status of *1810044A24Rik* was also verified by Pyrosequencing in reciprocal crosses of C57BL/6 and C3H/HeJ (Figure. S1.12, S1.13). The imprinting status for *Blcap* was not verified in C57BL/6 and C3H/HeJ due to lack of exonic SNPs. Two known imprinted genes, *Peg13* and *Nnat*, are located in the introns of *1810044A24Rik* and *Blcap*, respectively. The CpG island of *Peg13* is only methylated at the maternal allele [26]. Five differentially methylated CpG sites within the gene region of *Nnat* were previously identified [26], [37], so each of the three novel imprinted genes have DMRs near or within the gene regions (Table S1.19). Nine genes attained marginal significance only after pooling across all SNPs, but showed no single SNP with a significantly skewed frequency. In all 9 cases, Pyrosequencing demonstrated unambiguously that they were not imprinted (Table 1).

### Coverage of known imprinted genes in this study

Among the 98 known imprinted genes in mouse, 45 have both RefSeq ID and SNPs between AKR and PWD strains. 33 of the 45 known imprinted genes with SNPs were covered in our short-read sequence data. The remaining 12 genes were not covered by filtered high quality SNP-containing reads due to lack of detected expression in mouse neonatal brain (Table S1.6). 14 of 33 covered known imprinted genes are significant (Table 1). In the non-significant maternally expressed imprinted genes, *Ppp1r9a, Asb4*, *Calcr* and *Ube3a* have been reported as being imprinted in brain [38]–[41], and they all have a marginally significant *P*-value. *Ube3a* imprinting was verified by Pyrosequencing. Genes that have too low a high-quality SNP-containing read count, such as *Gnas*, *Gatm*, *Tnfrsf23*, *Zim1*, *Dcn*, *Nap1l4*, *Osbpl5*, *Grb10* and *Slc22a2* have an imprinting status that remains inconclusive, but the data are not consistent with strong imprinting (Table S1.6). All known maternally expressed genes covered with adequate depth of sequence reads had a pattern of allelic bias consistent with their known imprinting status. *Gtl2*, *H19*, *Cdkn1c* and *Commd1* are significant in the Solexa data and they are verified to be imprinted in neonatal brain. *Ppp1r9a* has significant nominal *P*-value but is not significant after multiple test correction. However, the Solexa counts are consistent with preferential maternal expression (Table S1.7). *Asb4*, *Calcr*, *Ube3a* has marginal significant *P*-value due to the small number of SNP-containing reads covered in the data, suggesting that they might be imprinted in neonatal brain. We verified that *Ube3a* is imprinted in neonatal brain by the Pyrosequencing method, with the *p_1_* and *p_2_* ratios 0.392 and 0.755. The other genes covered in the data, *Gatm, Tnfrsf23, Zim1, Dcn, Nap1l4, Osbpl5,* and *Slc22a2* are not significant, which is consistent with the fact that they are known to be imprinted in placenta instead of neonatal brain (Table S1.7). *Gnas,* a known imprinted gene in the pituitary but not in the whole brain of mouse [42]–[45], is not statistically significant in the Solexa data. However, the Pyrosequencing verification showed 0.459/0.562 ratio of *p_1_*/*p_2_*, suggesting that there is slightly higher expression from the allele inherited from mother . *Grb10* is imprinted in both placenta and brain [46]–[48] but does not show a significant difference between *p_1_* and *p_2_* in the Solexa data, despite adequate expression level to provide a test of adequate power. Subsequent Pyrosequencing verified the non-imprinting status in P2 neonatal brain (Table S1.7). In fact, *Grb10* is imprinted in mouse brain with paternal-only expression, but it shows maternal-only expression in other tissues [48]. It could be possible that *Grb10* is imprinted in other stages of brain (for example, fetal brain) but not P2 brain in mouse, or it is possible that the imprinting status varies among strains, and the AKR x PWD F1 fail to imprint *Grb10*. For the paternally expressed known imprinted genes that are not statistically significant in our data, *Magel2* and *Peg3* are consistent with 100% paternal expression. *Rtl1* and *Copg2* may be maternally expressed, as suggested by the sequence count data, but there were too few reads to attain statistical significance. While *Copg2* is maternally expressed, and *Rtl1* is expressed from the paternally inherited allele, the microRNA-containing antisense transcript is expressed from the maternal allele [49]. *Igf2* and *Slc38a4* are consistent with non-imprinting and, consistent with the pattern of expression in human and mouse [50]–[53], *Igf2* is verified by Pyrosequencing to be biallelically expressed in brain (Table S1.7).

### Closely-linked pairs of imprinted genes

**Figure 4:**
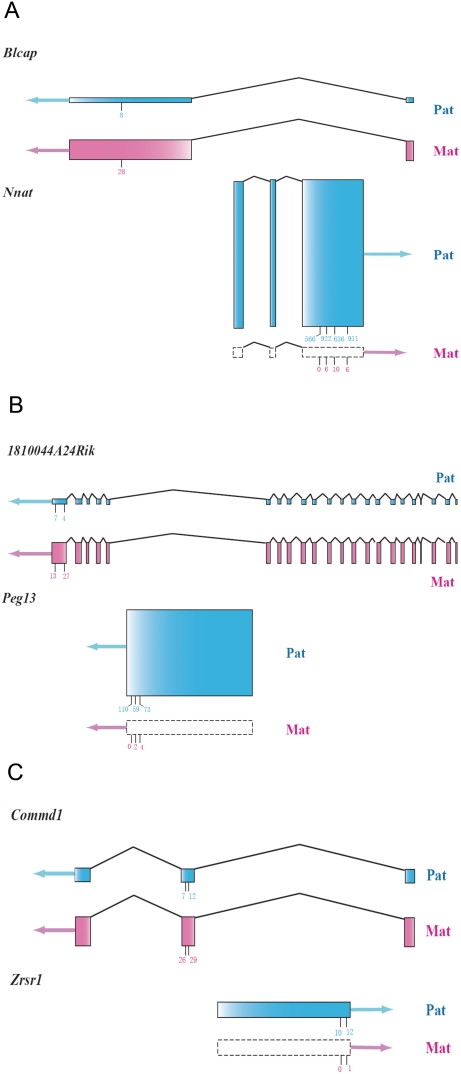
Sense-antisense gene pairs covered by the Illumina sequence data. Gene structures of the three gene pairs showing nested structures. The blue shading represents the paternal allele and the pink shading indicates for the maternal allele. Boxes with dashed lines indicate no expression. The arrows represent the direction of transcription. The sum of the heights of the two parental exons for each gene is in proportion to the expression level, which is quantified by the total counts of the perfect-match Illumina reads. The relative heights of the exons for the paternal and maternal allele within the same gene represent the relative expression level of the two parental alleles. The short vertical lines under the exons indicate the SNP positions, and the total counts of the two reciprocal crosses for the maternal and paternal allele are labeled.

Of the 10 sense-antisense pairs of known imprinted genes discovered to date [1], eight pairs are reciprocally imprinted (maternal expression for sense transcripts and paternal expression for antisense transcripts, or vice versa) [41], [49], [54]–[66] (Table S1.8). The remaining two show only paternal expression [51], [67], [68]. These cases of imprinting all were discovered and verified individually in different samples using different mouse strains (Table S1.8). In our Illumina sequence data, three reciprocally expressed closely linked sense-antisense (or sense-sense) pairs were covered adequately to perform statistical analysis (Table S1.9). Four of them are known imprinted genes (*Peg13*, *Nnat*, *Zrsr1*, *Commd1*) and two (*1810044A24Rik*, *Blcap*) are among our verified novel imprinted genes. *Peg13*, *Nnat* and *Zrsr1* are located in an intron of *1810044A24Rik*, *Blcap* and *Commd*, respectively. Interestingly, in the three pairs, *Peg13-1810044A24Rik, Nnat-Blcap* and *Zrsr1- Commd1*, the first gene is a paternally expressed imprinted gene with 100% monoallelic expression, whereas the second gene is maternally expressed partially imprinted gene (Figure 4). The pattern is consistent with the possibility that the monoallelic expression of the paternally expressed sense transcripts might reduce the relative expression of the paternal copy of the antisense transcript, resulting in predominantly maternal expression. Our hypothesis is that the paternally expressed imprinted gene is driving the apparent imprinting of the maternal gene, presumably through transcriptional interference. While this reciprocal imprinting has been noted in the literature [24], [69], [70] , this is the first genome-wide study identifying multiple, well quantified cases in mouse neonatal brains.

### Transcriptome-wide pattern of imprinting status

**Figure 5:**
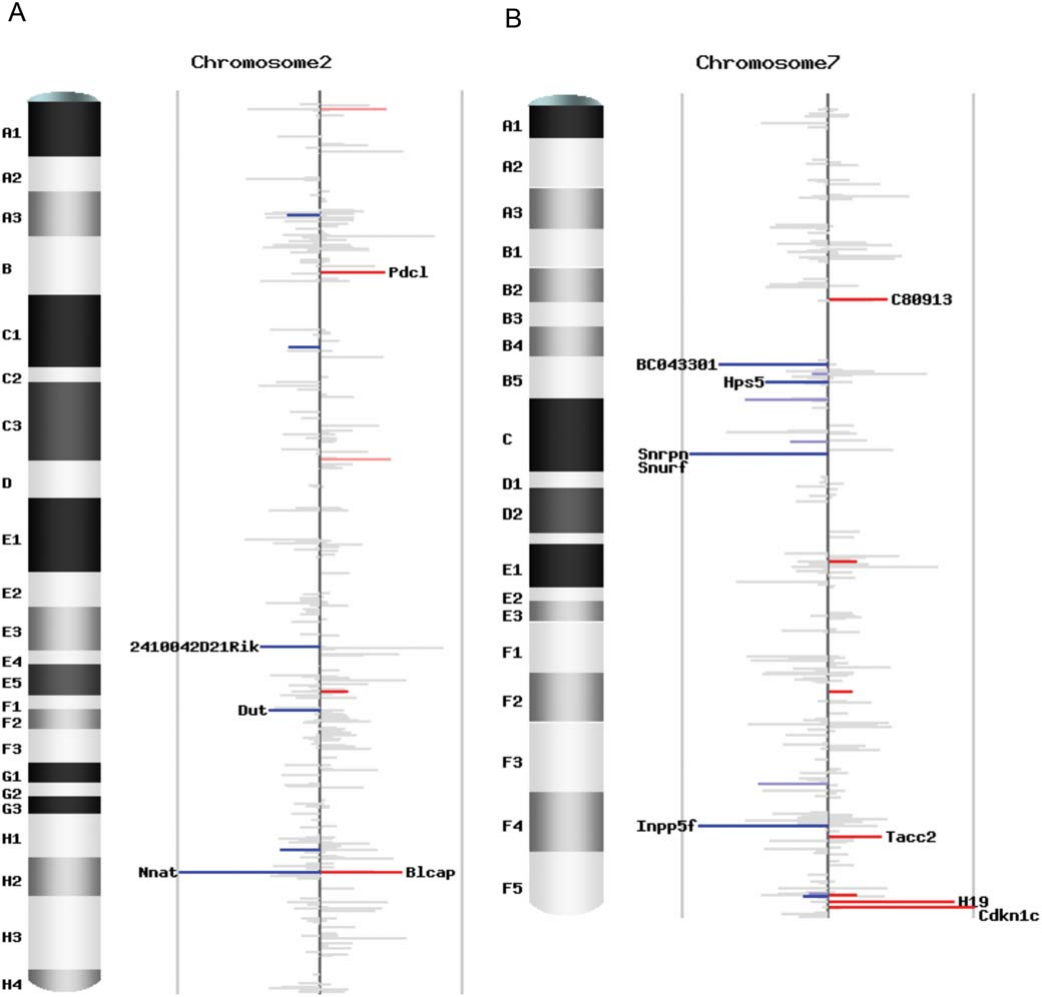
Chromosomal scans of imprinting status. (A) Imprinting status for chromosome 2. (B) Imprinting status for chromosome 7. Each plot contains unique Entrez genes covered by SNP-containing Illumina reads with counts no less than 4 in both reciprocal crosses. The height of each bar is the difference of the AKR percentage in the two reciprocal crosses (p1-p2), representing the intensity of imprinting. The color stands for the direction of imprinting, blue for paternal expression and red for maternal expression. The intensity of the color represents the significance, grey for not significant (*q*-value ≥0.10), lighter blue and pink for marginally significant (0.05≤ *q*-value <0.10), darker blue and red for significant (*q*-value <0.05). The gene name is indicated if | *p_1_*-*p_2_*| ≥0.3.

To investigate the pattern of imprinting status for all the transcripts covered by our study, we plotted the 5,076 unique Entrez genes with counts of four or more in both reciprocal crosses across the mouse genome (Figure 5; Figure S1.14). We define imprinting status as the difference between the AKR percentages in the two reciprocal crosses, which is *p_1_-p_2_* (Table S1.4). Most genes display a value of *p_1_*-*p_2_* close to zero, indicating a lack of significant imprinting. The sense-antisense pairs and the imprinted genes in known imprinting clusters are clearly demonstrated in the genome-wide plots (Figure S1.14). There are 1,606 non-significant genes with a total count 25 or more in both reciprocal crosses, forming a good tissue-specific non-imprinted dataset for computational prediction and evolutionary analysis (Table S1.10).

### Paternal-brain and maternal placenta bias of imprinted genes

When paternally- and maternally-expressed imprinted genes covered in the sequence read data are compared, we discovered an excess of paternal expression (11 paternal and 6 maternal), and most of these (9 of 11) show strong monoallelic expression (90%–100%). Three of the maternally expressed genes are only partially imprinted in brain with 70%–80% expression from the maternal allele (Table 1). Overall there is a bias toward paternally expressed imprinted genes in brain, whereas of the 29 genes reported to be imprinted in placenta, only 8 are paternally expressed (Table S1.11).

## Discussion

### Quantifying allele-specific expression with accurate ratios by directly counting the SNPs

Genomic imprinting is not always an “all-or-none” effect with 100% from the paternal or maternal allele. Instead, the degree of imprinting falls on a continuum from complete uniparental expression to equal expression of the two parental alleles. Microarray hybridization can identify uniparental expression, but it cannot give reliable ratios of the two parental alleles, since there is no good means to quantify the affinity difference between perfect and mismatch probes. The method of direct Sanger sequencing of the cDNA is not quantitative and will miss those cases with quantitative differences between maternal vs. paternal expression. To solve these problems, we sequenced the entire transcriptomes of mouse reciprocal F1 neonatal brains by the Illumina/Solexa sequencing method, and obtained relative expression ratios of the two parental alleles by counting the allele-specific sequence reads at the SNP positions within the transcripts. The method is well validated by independent methods (Pyrosequencing and Sanger sequencing). We present discoveries of the imprinting status of many genes for the neonatal brain, including genes not known to be imprinted in any tissue. Scoring allele-specific expression by short read transcriptome sequencing will be widely used whenever allele-specific differential expression is of interest, including quantification of *cis*-acting regulatory SNP effects [71].

### The path to exhaustive profiling of tissue- and developmental stage-specific genomic imprinting

The discovery of imprinted genes in humans and mice remains sporadic due to the hit-or-miss way that these genes have been discovered. Different studies used different mouse strains, testing imprinting status in different tissues and developmental time points, and none of the studies published to date has employed a truly transcriptome-wide screen for imprinting. Our study shows a way to quantitatively assess in a highly uniform manner the imprinting status of the entire transcriptome for each tissue. The uniformity of the short-read sequencing approach has clear advantages, and paves the way toward a catalog of imprinting status of all transcribed genes in the mouse and human.

### Imprinting of nested and closely-linked genes

Our short-read transcriptome sequencing approach identified three pairs of closely linked and reciprocally imprinted genes in which the paternally expressed genes showed 100% monoallelic expression whereas the maternally expressed genes are only partially imprinted in neonatal brains. These data are consistent with the scenario in which the paternally expressed gene is strongly imprinted, and by virtue of its imprinting, there is transcriptional interference, driving weaker expression of genes that are transcribed from the opposite strand (or are nested within the first transcript). This would impose an appearance of weak maternally expressed imprinting. The implications of the bias toward maternal expression in partially imprinted genes, paternal expression of strongly imprinted genes, and the apparent transcriptional interference of opposing strand transcripts all await further analysis to understand the mechanism regulating their imprinting as well as their functional and evolutionary implications.

### How many imprinted genes are there in the genome?

It has been estimated that about 1% of the genes in the mammalian genome are imprinted. However, this estimate has a wide range, from around 100 genes [2] to 600 genes [3], to more than 2,000 genes [72]. The variation is due to the ignorance of tissue-specificity of imprinting status and the inability to make inference about non-imprinted genes. Using our method, by counting the reads that correspond to the two parental alleles, we can specify the statistical confidence that a gene is not imprinted, as well as identifying those that are only partially imprinted. This enables determination of the statistical confidence that this list of imprinted genes is close to exhaustive in neonatal brains. In addition to the three novel imprinted genes we found in neonatal brain, we confirmed the imprinting status of 7 known imprinted genes and we also discovered the novel imprinting status in neonatal brain of 7 additional genes known to be imprinted in other tissues. With our coverage of more than 5,000 transcripts, we did not discover novel imprinting clusters, and only a small number of novel imprinted genes were found. Taken altogether, the data suggest that the list of genes that are imprinted in the neonatal brain is nearly complete, and the only remaining ones to be discovered are either expressed at very low levels, show a small parent-of-origin bias, or are imprinted in only a small portion of the brain.

## Materials and Methods

### Mouse Strains

Four mouse strains (C57BL/6, C3H/HeJ, AKR/J, PWD/PhJ) were purchased from the Jackson Laboratory (www.jax.org). We performed two pairs of mouse reciprocal crosses (C57BL/6 x C3H/HeJ, C3H/HeJ x C57BL/6, AKR/J x PWD/PhJ, PWD/PhJ x AKR/J). Total RNA samples were extracted from the P2 F1 mouse whole brains using the Qiagen RNeasy Lipid Tissue Mini Kit. RNA concentrations and A_260_ nm/A_280_ nm ratios were checked with a NanoDrop ND-1000 Spectrophotometer. RNA integrity was checked using the Agilent 2100 Bioanalyzer. All of the samples have a RIN (RNA integrity number) of 10.

All procedures involving mice have been approved by the Institutional Animal Care and Use Committee at Cornell University (protocol number 2002-0075, approved for three years beginning 01/27/2006). Cornell University is accredited by AAALAC.

### Illumina sequencing of the transcriptome

One Illumina Genome Analyzer run was performed for each reciprocal F1 of PWD and AKR mice at the Genome Center at Washington University. cDNA was synthesized using a modified version of the SMART Technology (ClonTech). To improve sequence coverage, we used a size selection procedure that removed cDNAs shorter than 1.3 kb in length. One Illumina Genome Analyzer run of each reciprocal F1 sample was run on the Illumina Genome Analyzer.

#### -Synopsis

Mouse total RNA was converted to first strand cDNA using a modified-SMART protocol. The first strand cDNA was then PCR amplified and size fractionated in 6% polyethylene glycol (PEG)/0.55M sodium chloride (NaCl) to enrich for cDNA ≤1250bp. SMART adapters were then excised from the cDNAs using *Mme*I and the adapters were removed from the reaction using 11% PEG/0.5M NaCl. The purified cDNA population then was fragmented and used as the source for a standard Illumina fragment library.

#### -Modified-SMART

First strand cDNA was produced from mouse total RNA according to a modified version of the Clontech SMART protocol (E. Mardis, personal communication), using approximately 1 µg of total RNA and SuperScript II (Invitrogen).

#### -Cycle optimization PCR and production PCR

The modified-SMART cDNA was used as the template in a PCR reaction to determine the number of cycles at which the reaction is no longer exponential. The cycle optimization reaction used 1 µl of the first strand cDNA reaction. Aliquots were removed at 2 cycle timepoints between 16 and 24 cycles. They were then run on a Flashgel (Lonza) for 5 min at 275 v, and the optimum cycle number was determined by observation.

The production PCR consisted of eight 100 µl reactions identical in composition to the cycle optimization reaction except that 2 µl of first-strand cDNA was used for each reaction and the empirically determined cycle optimum number was used for amplification of all eight reactions. The PCR products were purified and concentrated with two Qiaquick columns (Qiagen), according to the manufacturer's protocol, and eluted with 30 µl Buffer EB (Qiagen) per column.

#### -Size fractionation

To fractionate cDNA ≤1250 bp, the amplified cDNA from the production PCR reactions was resuspended in a 300 µl reaction of 6% PEG-8000, 0.55 M NaCl and carboxylate paramagnetic beads. The mixture was vigorously vortexed and incubated for 10 min at room temperature. The reaction was placed on a magnetic particle collector (MPC, Invitrogen) for two min and the supernatant, containing the ≤1250 bp fraction, was transferred to a clean tube. This cDNA fraction was purified over a Qiaquick column according to the manufacturer's protocol, and eluted in 50 µl Buffer EB.

#### -Adapter removal and cDNA purification

The 5′ and 3′ adapters added during cDNA synthesis, contain *Mme*I recognition sequences that are removed by digestion in a 100 µl reaction containing 1× NEB Buffer 4 (20 mM Tris-acetate, 50 mM potassium acetate, 10 mM magnesium acetate, 1 mM dithiothreitol, pH 7.9 @ 25°C), 10 µg of 10mg/ml BSA, 64 µM S-adenosylmethionine (New England Biolabs) and 12 units *Mme*I (New England Biolabs) for 30 min at 37°C. The digested cDNA was purified and concentrated with 1 Qiaquick column according to the manufacturer's protocol, and eluted with 30 µl Buffer EB.

A second round of PEG/NaCl fractionation further removes the adapter fragments released by *Mme*1 digestion. Here, the cDNAs purified by Qiaquick column were resuspended in a 300 µl reaction of 11% PEG-8000, 0.5M NaCl and carboxylate paramagnetic beads. The mixture was vigorously vortexed and incubated for 10 min at room temperature. The reaction was placed on an MPC for two min and the supernatant was then discarded. The paramagnetic beads were washed twice with 70% ethanol and air dried. The tube containing the paramagnetic beads was removed from the MPC and the beads were resuspended in 50 µl Buffer EB with vigorous vortexing. The reaction was placed on the MPC for two min and the supernatant was transferred to a clean tube. This fraction contains cDNA >150 bp and free of 5′ & 3′ adapters.

#### -Nebulization/Covaris shearing and Illumina/Solexa library prep

Sample B17 (PWD/PhJ x AKR/J): The cDNA was sheared by nebulization (2 min at 50 PSI) and the sheared DNA was purified/concentrated with a single Qiaquick column according to the manufacturer's protocol. Sample B21 (AKR/J x PWD/PhJ): The cDNA was sheared with the Covaris S2 System in 75% glycerol with the following program: 10 cycles of 4 treatments, 60 sec each; Duty cycle = 20%; intensity = 10; 1000 cycles/burst. The cDNA was purified/concentrated by ethanol precipitation.

The sheared cDNAs were then prepared for Illumina sequencing according to the manufacturer's protocols. Libraries were prepared from a 150–200 bp size-selected fraction following adapter ligation and agarose gel separation. The libraries were sequenced using a single end read protocol with 32 bp of data collected per run on the Illumina Genome Analyzer. Data analysis and base calling were performed by the Illumina instrument software.

### Illumina sequence data analysis

We obtained 33,519,739 reads (1072.63 Mbp total) from the PWD/PhJ x AKR/J cross (PWD x AKR for short) in seven lanes, and 35,510,887 reads (1136.35 Mbp total) in eight lanes for the reciprocal cross, AKR/J x PWD/PhJ (AKR x PWD for short). Both runs have high sequence quality with 95% of the bases passing Q20 (Figure S1.1).

We used a local version of the NCBI BLAST program (http://www.ncbi.nlm.nih.gov/blast/Blast.cgi) to align the 32-bp reads to the mouse RefSeq database (http://www.ncbi.nlm.nih.gov/RefSeq/). The parameters for the blastn program were optimized for short reads and our purpose. We did the BLAST jobs on 180 nodes of the CBSU clusters (http://cbsuapps.tc.cornell.edu/index.aspx) using the P-BLAST utility. 23.82% of the total reads in the PWR x AKR cross were aligned to the RefSeq database with 3.57 hits/read. 31.18% of the total reads in the AKR x PWD cross were aligned to the RefSeq database with 3.02 hits/read (Table S1.1). High quality SNP-containing reads were filtered out, with unique match to the RefSeq gene (or different transcripts of the same Entrez gene). Relative expression level of the two parental alleles was estimated by the relative counts of Illumina reads at the SNP positions in the Perlegen mouse SNP database (Tables S1.14–S1.18; Figures S1.15–S1.20). 59 out of the 98 known imprinted genes in mouse are in the mouse RefSeq database. We assembled them into ace files according to the BLAST alignment information. 20 novel SNPs were called in 12 known imprinted genes from the Illumina assembly (Tables S1.12 and S1.13).

### Estimation of the relative parental expression

To identify the SNP positions in the mouse RefSeq database, we used the SNP genotype and information in the Perlegen mouse SNP database (http://mouse.perlegen.com). Perlegen Sciences and NIEHS genotyped 8 million SNPs among 15 mouse strains with a genome coverage of 70%, including PWD and AKR. The SNP density is approximately 3 SNPs/kb and most of the genic regions are covered in the database. The genome coordinates of the reviewed and validated mouse RefSeq sequences (starting with NM and NR, see http://www.ncbi.nlm.nih.gov/RefSeq/key.html#status) were downloaded from the UCSC genome browser (www.genome.ucsc.edu, July 2007 assembly). The SNP positions in the RefSeq sequences were filtered by the RefSeq gene coordinates. To correct for gaps in the RefSeq-genomic sequence alignments, we also did text matches using 20 bp upstream and downstream the SNP positions. A total of 206,589 Perlegen SNPs were found in 18,797 RefSeq sequences (Tables S1.14 and S1.15), with an average SNP density of 11 SNPs/RefSeq sequence (Figure S1.15). 4,127 SNPs with missing data in the Perlegen SNP database were called based on the Illumina sequence reads. The genotypes of all the high quality Perlegen SNPs (*q*-score ≥10, Mismatch ≤4 for alternative allele, Mismatch ≤3 for reference allele and match length ≥28) covered in the Illumina reads were summarized in the two reciprocal F1s. 175,687 (84.71%) of the 207,407 Perlegen RefSeq SNPs were not covered or not informative (less than 1 SNP count in both direction). In the 31,720 Illumina-covered Perlegen SNPs, 25,289 (83.21%) were confirmed by Illumina reads, and 4,127 (13.58%) Perlgen SNPs with missing data (N) in AKR and PWD strains were called based on the Illumina sequence information (Figure S1.19). The newly called SNPs were included in the data analysis. From the results, the genotype of the Illumina short-read sequence identified SNPs are consistent with the Perlegen SNP, indicating high sequence quality of our Illumina Genome Analyzer run. There are only 161 inconsistent SNPs, most of which are the complementary allele and could come from the antisense transcript of the RefSeq gene.

The expression level of the RefSeq transcripts were quantified by the number of perfectly matched reads in the Illumina sequence data. 15,491 RefSeq genes were covered by at least one perfect match read in each of the two reciprocal crosses (Figure S1.20).

In order to do the quality control and filter out the true SNP-containing reads, several criteria were considered. The Illumina sequence SNPs (Perlegen SNP that are present in our Illumina reads) were classified to six categories according to their consistency with the Perlegen SNP information (Table S1.16). Classes 1–5 are the consistent SNPs. Class 1 includes SNPs that are polymorphic between AKR and PWD strains. These are the SNPs we want to use in our study to quantify the relative parental expression. Class 2 SNPs are also consistent but the SNP is not polymorphic between AKR and PWD strains. Classes 3–5 are SNPs that have missing data (N) in the Perlegen database. The rest of the Illumina SNPs are classified in class 0, which are the inconsistent SNPs. Most of the Illumina SNPs have a quality score 20 or above (Figure S1.16). The distribution of the number of mismatches showed that the pattern class 1 SNPs are consistent with perfectly matched reference and alternative alleles, an attribute not seen in any other SNP classes (Figure S1.17). So the class 1 SNPs were used in the following analysis. Regarding the match length of the SNP-containing reads, more than 80% have a full length match (32 bp), and most of the reads have a match length of 25 or more. The blastn algorithm is a local alignment algorithm, so if there are SNPs in the first or last 2 bp of a read, the alignment will be truncated, although it is still considered a full length match (Figure S1.18). Two sets of filter criteria were used before the final SNP counts for each RefSeq gene were summarized (Table S1.2). Both Filter 1 and Filter 2 are conservative and the reads after the filtering all matched uniquely to the Entrez gene database (could be more than one RefSeqs due to alternative splicing). Since there is no lane effect, the AKR and PWD counts in the two reciprocal crosses are summarized by RefSeq genes and by SNPs. 326 class 1 SNPs are not polymorphic in the Illumina sequence data due to the repetitive match of the SNP-containing sequence in the mouse genome, so we do not know where transcripts bearing these SNPs are coming from. Such SNPs are excluded from the final analysis (Table S1.17).

### Detecting genomic imprinting and Statistical analysis

We have the filtered AKR and PWD allele counts for the two reciprocal F1s. We define *p_1_* as the AKR allele proportion in the PWD x AKR cross and *p_2_* as the AKR allele proportion in the AKR x PWD cross (Table S1.4). If a gene has equal expression from the two parental alleles, *p_1_* and *p_2_* will be approximately 0.5. If a gene is an expression QTL (eQTL) with higher expression from the AKR-derived allele, *p_1_* will be approximately equal to *p_2_* and both *p_1_* and *p_2_* will be greater than 0.5. A paternally expressed imprinted gene will have the pattern of *p_1_*>0.5 and *p_2_* <0.5, whereas a maternally expressed imprinted gene will have the pattern of *p_1_* <0.5 and *p_2_*>0.5 (Table S1.5). The advantage of having the reciprocal crosses is that we could distinguish an eQTL from true genomic imprinting.

A formal statistical test is needed to test the significance. We did not use Fisher's exact test because it is a conservative test and results in substantial loss of power, especially when the total counts are small [73]. Rather, we used a modern statistical method, the Storer-Kim method for two independent binomials to test whether there is a significant difference between the two binomial parameters, *p_1_* and *p_2_* [18]. The *P*-values were calculated using Wilcox's code [19] in R (version 2.60, www.r-project.org). The 95% confidence intervals for *p_1_* and *p_2_* were also obtained by the Wilson method [74] (R, the binom package). False discovery rate (*q*-value) was calculated by the qvalue package in R [20].

### Sanger and Pyrosequencing verification

We designed Pyrosequencing PCR and sequencing primers for the candidate imprinted genes using Pyrosequencing Assay Design Software Version 1.0.6 (Biotage AB). To guarantee that there are no SNPs within the primers, SNP positions in the Perlegen database were labeled and excluded when designing the primers. PCR amplification for Pyrosequencing was done using TaqGold Enzyme (Applied Biosystems) with a 45 cycles of 3-step PCR (95°C for 45 s, 46–58°C for 30 s and 72°C for 10–20 s) followed by a final extension of 10 min. The PCR products (80–300 bp) were purified by Exonuclease I and Shrimp Alkaline Phosphatase and sequenced bidirectionally using the original Pyro PCR primers on ABI 3730xl DNA analyzer (Applied Biosystems) with BigDye Terminator v3.1. The sequence chromatograms were analyzed with CodonCode Aligner version 2.0.4 (CodonCode Corporation Software for DNA Sequencing). PCR products for Pyrosequencing were amplified using the same condition with biotin labeled forward (or reverse) primer. The Pyrosequencing was done on a PSQ™ 96 MA Pyrosequencer (Biotage, AB) with the Pyro Gold Reagents (Biotage, AB). The relative level of the two parental alleles was quantified by the software for PSQ™ 96 MA Pyrosequencer (Version 2.02 RC 5.8, Biotage, AB) using the allele quantification method.

## Supporting Information

Figure S1 This file contains figures S1.1 through S1.20 as a bookmarked pdf.
(2.71 MB PDF)

Table S1 This file contains tables S1.1 through S1.19 as a bookmarked pdf.
(0.35 MB PDF)

References S1 Supporting references for Table 1.
(0.03 MB DOC)

## References

[1] Morison IM, Ramsay JP, Spencer HG (2005). A census of mammalian imprinting.. Trends Genet.

[2] Luedi PP, Dietrich FS, Weidman JR, Bosko JM, Jirtle RL (2007). Computational and experimental identification of novel human imprinted genes.. Genome Res.

[3] Luedi PP, Hartemink AJ, Jirtle RL (2005). Genome-wide prediction of imprinted murine genes.. Genome Res.

[4] Pollard KS, Serre D, Wang X, Tao H, Grundberg E (2008). A genome-wide approach to identifying novel-imprinted genes.. Hum Genet.

[5] Lomvardas S, Barnea G, Pisapia DJ, Mendelsohn M, Kirkland J (2006). Interchromosomal interactions and olfactory receptor choice.. Cell.

[6] Gimelbrant A, Hutchinson JN, Thompson BR, Chess A (2007). Widespread monoallelic expression on human autosomes.. Science.

[7] Schulz R, Menheniott TR, Woodfine K, Wood AJ, Choi JD (2006). Chromosome-wide identification of novel imprinted genes using microarrays and uniparental disomies.. Nucleic Acids Res.

[8] Yamazawa K, Kagami M, Ogawa M, Horikawa R, Ogata T (2008). Placental hypoplasia in maternal uniparental disomy for chromosome 7.. Am J Med Genet A.

[9] Ogata T, Kagami M, Ferguson-Smith AC (2008). Molecular mechanisms regulating phenotypic outcome in paternal and maternal uniparental disomy for chromosome 14.. Epigenetics.

[10] Cattanach BM, Kirk M (1985). Differential activity of maternally and paternally derived chromosome regions in mice.. Nature.

[11] Cattanach BM, Barr JA, Evans EP, Burtenshaw M, Beechey CV (1992). A candidate mouse model for Prader-Willi syndrome which shows an absence of Snrpn expression.. Nat Genet.

[12] Ferguson-Smith AC, Cattanach BM, Barton SC, Beechey CV, Surani MA (1991). Embryological and molecular investigations of parental imprinting on mouse chromosome 7.. Nature.

[13] Serre D, Gurd S, Ge B, Sladek R, Sinnett D (2008). Differential Allelic Expression in the Human Genome: A Robust Approach To Identify Genetic and Epigenetic Cis-Acting Mechanisms Regulating Gene Expression.. PLoS Genetics.

[14] Bjornsson HT, Albert TJ, Ladd-Acosta CM, Green RD, Rongione MA (2008). SNP-specific array-based allele-specific expression analysis.. Genome Res.

[15] Frazer KA, Eskin E, Kang HM, Bogue MA, Hinds DA (2007). A sequence-based variation map of 8.27 million SNPs in inbred mouse strains.. Nature.

[16] Matoba R, Kato K, Saito S, Kurooka C, Maruyama C (2000). Gene expression in mouse cerebellum during its development.. Gene.

[17] Chrast R, Scott HS, Papasavvas MP, Rossier C, Antonarakis ES (2000). The mouse brain transcriptome by SAGE: differences in gene expression between P30 brains of the partial trisomy 16 mouse model of Down syndrome (Ts65Dn) and normals.. Genome Res.

[18] Storer BE, Kim C (1990). Exact Properties of Some Exact Test Statistics for Comparing 2 Binomial Proportions.. Journal of the American Statistical Association.

[19] Wilcox RR (2003). Applying contemporary statistical techniques..

[20] Storey JD, Taylor JE, Siegmund D (2004). Strong control, conservative point estimation and simultaneous conservative consistency of false discovery rates: a unified approach.. Journal of the Royal Statistical Society Series B-Statistical Methodology.

[21] Kagitani F, Kuroiwa Y, Wakana S, Shiroishi T, Miyoshi N (1997). Peg5/Neuronatin is an imprinted gene located on sub-distal chromosome 2 in the mouse.. Nucleic Acids Res.

[22] Choi JD, Underkoffler LA, Wood AJ, Collins JN, Williams PT (2005). A novel variant of Inpp5f is imprinted in brain, and its expression is correlated with differential methylation of an internal CpG island.. Mol Cell Biol.

[23] Plass C, Shibata H, Kalcheva I, Mullins L, Kotelevtseva N (1996). Identification of Grf1 on mouse chromosome 9 as an imprinted gene by RLGS-M.. Nat Genet.

[24] Wang Y, Joh K, Masuko S, Yatsuki H, Soejima H (2004). The mouse Murr1 gene is imprinted in the adult brain, presumably due to transcriptional interference by the antisense-oriented U2af1-rs1 gene.. Mol Cell Biol.

[25] Leff SE, Brannan CI, Reed ML, Ozcelik T, Francke U (1992). Maternal imprinting of the mouse Snrpn gene and conserved linkage homology with the human Prader-Willi syndrome region.. Nat Genet.

[26] Smith RJ, Dean W, Konfortova G, Kelsey G (2003). Identification of novel imprinted genes in a genome-wide screen for maternal methylation.. Genome Res.

[27] Piras G, El Kharroubi A, Kozlov S, Escalante-Alcalde D, Hernandez L (2000). Zac1 (Lot1), a potential tumor suppressor gene, and the gene for epsilon-sarcoglycan are maternally imprinted genes: identification by a subtractive screen of novel uniparental fibroblast lines.. Mol Cell Biol.

[28] Schmidt JV, Matteson PG, Jones BK, Guan XJ, Tilghman SM (2000). The Dlk1 and Gtl2 genes are linked and reciprocally imprinted.. Genes Dev.

[29] Hagiwara Y, Hirai M, Nishiyama K, Kanazawa I, Ueda T (1997). Screening for imprinted genes by allelic message display: identification of a paternally expressed gene impact on mouse chromosome 18.. Proc Natl Acad Sci U S A.

[30] Hemberger M, Redies C, Krause R, Oswald J, Walter J (1998). H19 and Igf2 are expressed and differentially imprinted in neuroectoderm-derived cells in the mouse brain.. Dev Genes Evol.

[31] Hatada I, Mukai T (1995). Genomic imprinting of p57KIP2, a cyclin-dependent kinase inhibitor, in mouse.. Nat Genet.

[32] McLaughlin D, Vidaki M, Renieri E, Karagogeos D (2006). Expression pattern of the maternally imprinted gene Gtl2 in the forebrain during embryonic development and adulthood.. Gene Expr Patterns.

[33] Yevtodiyenko A, Steshina EY, Farner SC, Levorse JM, Schmidt JV (2004). A 178-kb BAC transgene imprints the mouse Gtl2 gene and localizes tissue-specific regulatory elements.. Genomics.

[34] da Rocha ST, Tevendale M, Knowles E, Takada S, Watkins M (2007). Restricted co-expression of Dlk1 and the reciprocally imprinted non-coding RNA, Gtl2: implications for cis-acting control.. Dev Biol.

[35] Davies W, Smith RJ, Kelsey G, Wilkinson LS (2004). Expression patterns of the novel imprinted genes Nap1l5 and Peg13 and their non-imprinted host genes in the adult mouse brain.. Gene Expr Patterns.

[36] Evans HK, Weidman JR, Cowley DO, Jirtle RL (2005). Comparative phylogenetic analysis of blcap/nnat reveals eutherian-specific imprinted gene.. Mol Biol Evol.

[37] Kikyo N, Williamson CM, John RM, Barton SC, Beechey CV (1997). Genetic and functional analysis of neuronatin in mice with maternal or paternal duplication of distal Chr 2.. Dev Biol.

[38] Ono R, Shiura H, Aburatani H, Kohda T, Kaneko-Ishino T (2003). Identification of a large novel imprinted gene cluster on mouse proximal chromosome 6.. Genome Res.

[39] Mizuno Y, Sotomaru Y, Katsuzawa Y, Kono T, Meguro M (2002). Asb4, Ata3, and Dcn are novel imprinted genes identified by high-throughput screening using RIKEN cDNA microarray.. Biochem Biophys Res Commun.

[40] Hoshiya H, Meguro M, Kashiwagi A, Okita C, Oshimura M (2003). Calcr, a brain-specific imprinted mouse calcitonin receptor gene in the imprinted cluster of the proximal region of chromosome 6.. J Hum Genet.

[41] Albrecht U, Sutcliffe JS, Cattanach BM, Beechey CV, Armstrong D (1997). Imprinted expression of the murine Angelman syndrome gene, Ube3a, in hippocampal and Purkinje neurons.. Nat Genet.

[42] Weinstein LS, Liu J, Sakamoto A, Xie T, Chen M (2004). Minireview: GNAS: normal and abnormal functions.. Endocrinology.

[43] Weinstein LS, Yu S, Warner DR, Liu J (2001). Endocrine manifestations of stimulatory G protein alpha-subunit mutations and the role of genomic imprinting.. Endocr Rev.

[44] Weinstein LS, Yu S, Ecelbarger CA (2000). Variable imprinting of the heterotrimeric G protein G(s) alpha-subunit within different segments of the nephron.. Am J Physiol Renal Physiol.

[45] Yu S, Yu D, Lee E, Eckhaus M, Lee R (1998). Variable and tissue-specific hormone resistance in heterotrimeric Gs protein alpha-subunit (Gsalpha) knockout mice is due to tissue-specific imprinting of the gsalpha gene.. Proc Natl Acad Sci U S A.

[46] Mergenthaler S, Hitchins MP, Blagitko-Dorfs N, Monk D, Wollmann HA (2001). Conflicting reports of imprinting status of human GRB10 in developing brain: how reliable are somatic cell hybrids for predicting allelic origin of expression?. Am J Hum Genet.

[47] Blagitko N, Mergenthaler S, Schulz U, Wollmann HA, Craigen W (2000). Human GRB10 is imprinted and expressed from the paternal and maternal allele in a highly tissue- and isoform-specific fashion.. Hum Mol Genet.

[48] Hikichi T, Kohda T, Kaneko-Ishino T, Ishino F (2003). Imprinting regulation of the murine Meg1/Grb10 and human GRB10 genes; roles of brain-specific promoters and mouse-specific CTCF-binding sites.. Nucleic Acids Res.

[49] Seitz H, Youngson N, Lin SP, Dalbert S, Paulsen M (2003). Imprinted microRNA genes transcribed antisense to a reciprocally imprinted retrotransposon-like gene.. Nat Genet.

[50] Ohlsson R, Hedborg F, Holmgren L, Walsh C, Ekstrom TJ (1994). Overlapping patterns of IGF2 and H19 expression during human development: biallelic IGF2 expression correlates with a lack of H19 expression.. Development.

[51] DeChiara TM, Robertson EJ, Efstratiadis A (1991). Parental imprinting of the mouse insulin-like growth factor II gene.. Cell.

[52] Jones BK, Levorse J, Tilghman SM (2001). Deletion of a nuclease-sensitive region between the Igf2 and H19 genes leads to Igf2 misregulation and increased adiposity.. Hum Mol Genet.

[53] Charalambous M, Menheniott TR, Bennett WR, Kelly SM, Dell G (2004). An enhancer element at the Igf2/H19 locus drives gene expression in both imprinted and non-imprinted tissues.. Dev Biol.

[54] Peters J, Wroe SF, Wells CA, Miller HJ, Bodle D (1999). A cluster of oppositely imprinted transcripts at the Gnas locus in the distal imprinting region of mouse chromosome 2.. Proc Natl Acad Sci U S A.

[55] Coombes C, Arnaud P, Gordon E, Dean W, Coar EA (2003). Epigenetic properties and identification of an imprint mark in the Nesp-Gnasxl domain of the mouse Gnas imprinted locus.. Mol Cell Biol.

[56] Lee YJ, Park CW, Hahn Y, Park J, Lee J (2000). Mit1/Lb9 and Copg2, new members of mouse imprinted genes closely linked to Peg1/Mest(1).. FEBS Lett.

[57] Kim J, Noskov VN, Lu X, Bergmann A, Ren X (2000). Discovery of a novel, paternally expressed ubiquitin-specific processing protease gene through comparative analysis of an imprinted region of mouse chromosome 7 and human chromosome 19q13.4.. Genome Res.

[58] Kim J, Bergmann A, Wehri E, Lu X, Stubbs L (2001). Imprinting and evolution of two Kruppel-type zinc-finger genes, ZIM3 and ZNF264, located in the PEG3/USP29 imprinted domain.. Genomics.

[59] Chamberlain SJ, Brannan CI (2001). The Prader-Willi syndrome imprinting center activates the paternally expressed murine Ube3a antisense transcript but represses paternal Ube3a.. Genomics.

[60] Paulsen M, Davies KR, Bowden LM, Villar AJ, Franck O (1998). Syntenic organization of the mouse distal chromosome 7 imprinting cluster and the Beckwith-Wiedemann syndrome region in chromosome 11p15.5.. Hum Mol Genet.

[61] Gould TD, Pfeifer K (1998). Imprinting of mouse Kvlqt1 is developmentally regulated.. Hum Mol Genet.

[62] Fitzpatrick GV, Soloway PD, Higgins MJ (2002). Regional loss of imprinting and growth deficiency in mice with a targeted deletion of KvDMR1.. Nat Genet.

[63] Barlow DP, Stoger R, Herrmann BG, Saito K, Schweifer N (1991). The mouse insulin-like growth factor type-2 receptor is imprinted and closely linked to the Tme locus.. Nature.

[64] Hu JF, Balaguru KA, Ivaturi RD, Oruganti H, Li T (1999). Lack of reciprocal genomic imprinting of sense and antisense RNA of mouse insulin-like growth factor II receptor in the central nervous system.. Biochem Biophys Res Commun.

[65] Kay GF, Barton SC, Surani MA, Rastan S (1994). Imprinting and X chromosome counting mechanisms determine Xist expression in early mouse development.. Cell.

[66] Sado T, Wang Z, Sasaki H, Li E (2001). Regulation of imprinted X-chromosome inactivation in mice by Tsix.. Development.

[67] Jong MT, Carey AH, Caldwell KA, Lau MH, Handel MA (1999). Imprinting of a RING zinc-finger encoding gene in the mouse chromosome region homologous to the Prader-Willi syndrome genetic region.. Hum Mol Genet.

[68] Moore T, Constancia M, Zubair M, Bailleul B, Feil R (1997). Multiple imprinted sense and antisense transcripts, differential methylation and tandem repeats in a putative imprinting control region upstream of mouse Igf2.. Proc Natl Acad Sci U S A.

[69] Sleutels F, Tjon G, Ludwig T, Barlow DP (2003). Imprinted silencing of Slc22a2 and Slc22a3 does not need transcriptional overlap between Igf2r and Air.. Embo J.

[70] Sleutels F, Zwart R, Barlow DP (2002). The non-coding Air RNA is required for silencing autosomal imprinted genes.. Nature.

[71] Nagalakshmi U, Wang Z, Waern K, Shou C, Raha D (2008). The transcriptional landscape of the yeast genome defined by RNA sequencing.. Science.

[72] Nikaido I, Saito C, Mizuno Y, Meguro M, Bono H (2003). Discovery of imprinted transcripts in the mouse transcriptome using large-scale expression profiling.. Genome Res.

[73] Lehmann EL, Romano JP (2005). Testing statistical hypotheses..

[74] Wilson EB (1927). Probable inference, the law of succession, and statistical inference.. Journal of the American Statistical Association.

