# Supplementary material for: Transcriptome-Wide Identification of Novel Imprinted Genes in Neonatal Mouse Brain
Source: PLoS One. 2008 Dec 4;3(12):e3839. doi: 10.1371/journal.pone.0003839 (PMC2585789; doi:10.1371/journal.pone.0003839)
Supplement: Figure S1 — This file contains figures S1.1 through S1.20 as a bookmarked pdf. (2.71 MB PDF) [file pone.0003839.s001.pdf]

Figure S1.1. The quality score distribution of the Solexa data.

A. 1) Quality score for lane 1, PWD x AKR.

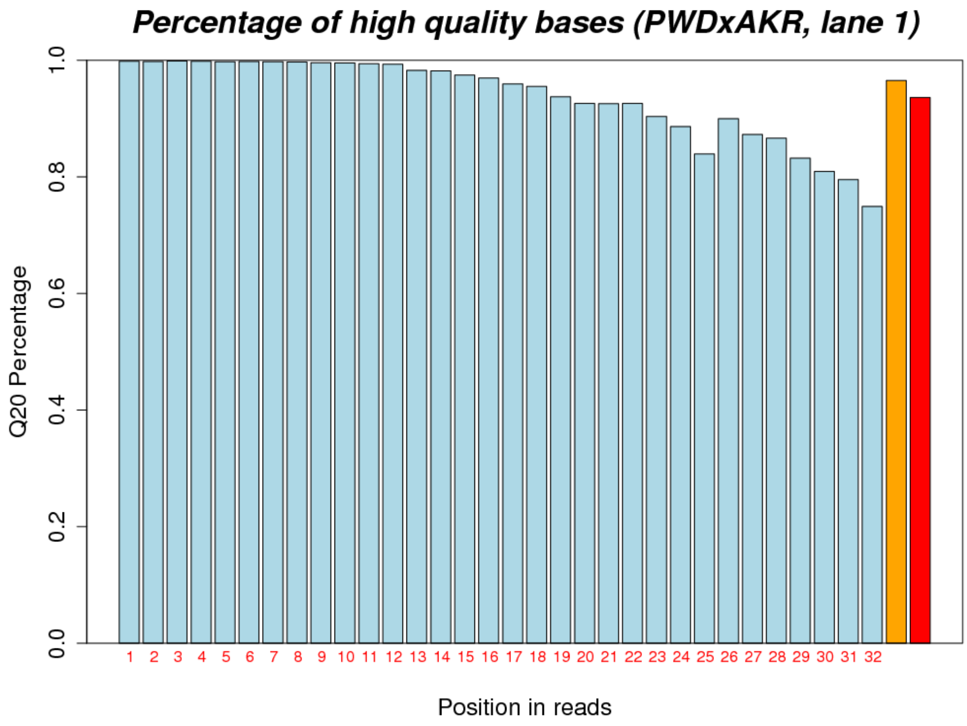

A. 2) Quality score for lane 2, PWD x AKR.

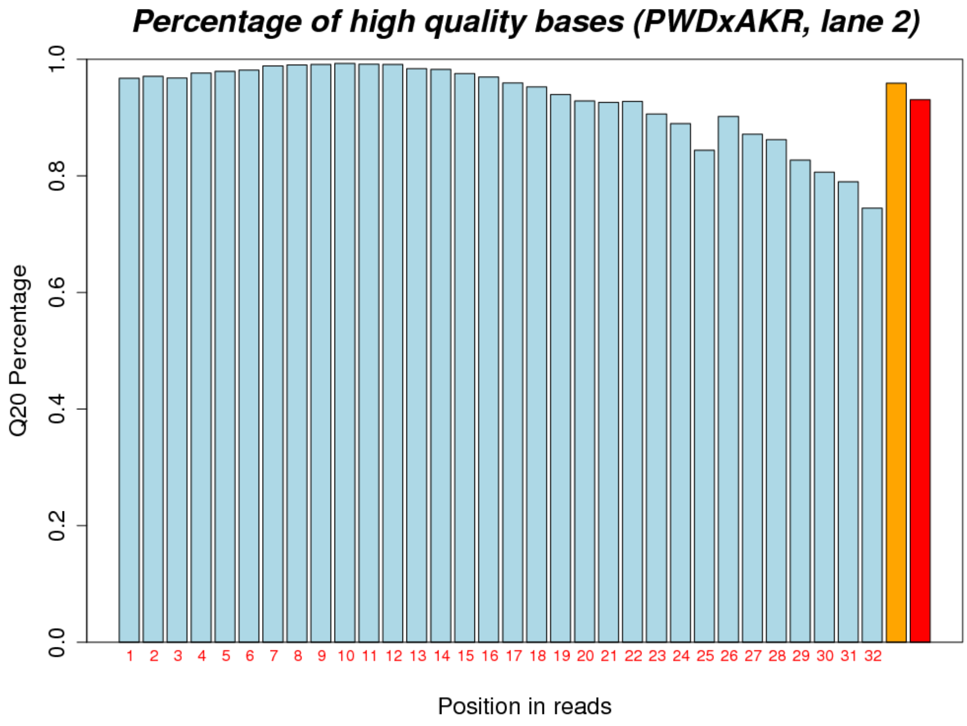

A. 3) Quality score for lane 3, PWD x AKR.

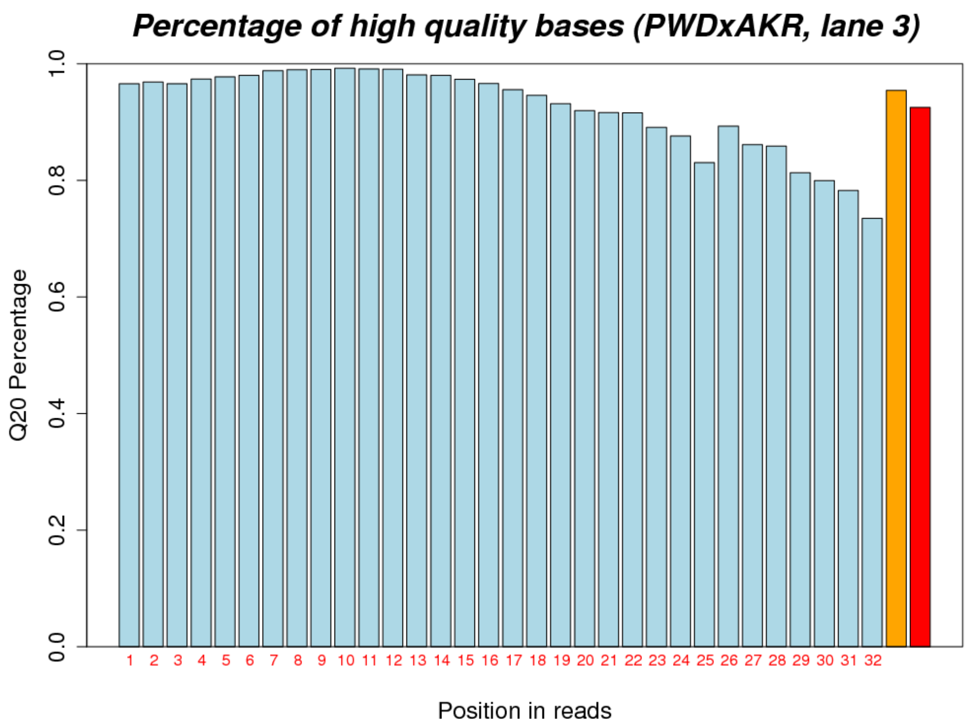

A. 4) Quality score for lane 4, PWD x AKR.

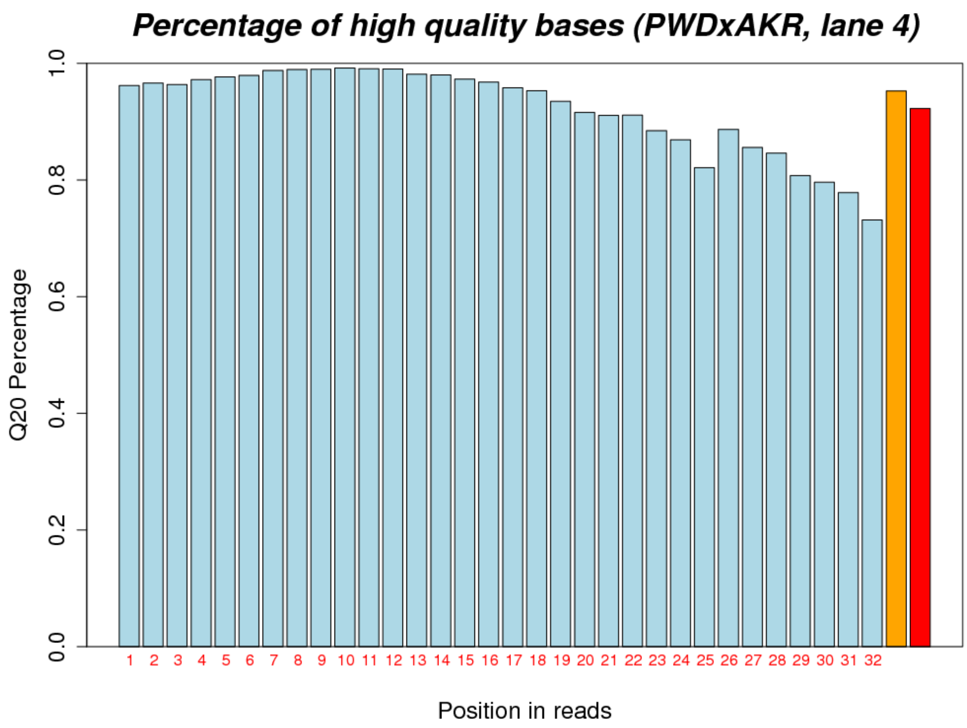

A. 5) Quality score for lane 6, PWD x AKR.

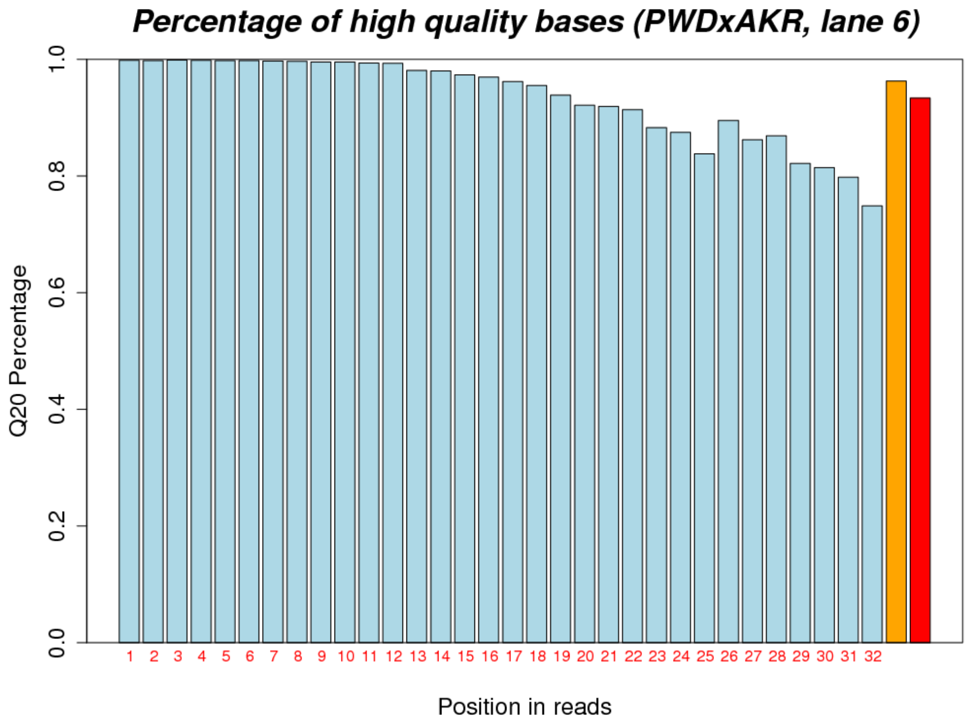

A. 6) Quality score for lane 7, PWD x AKR.

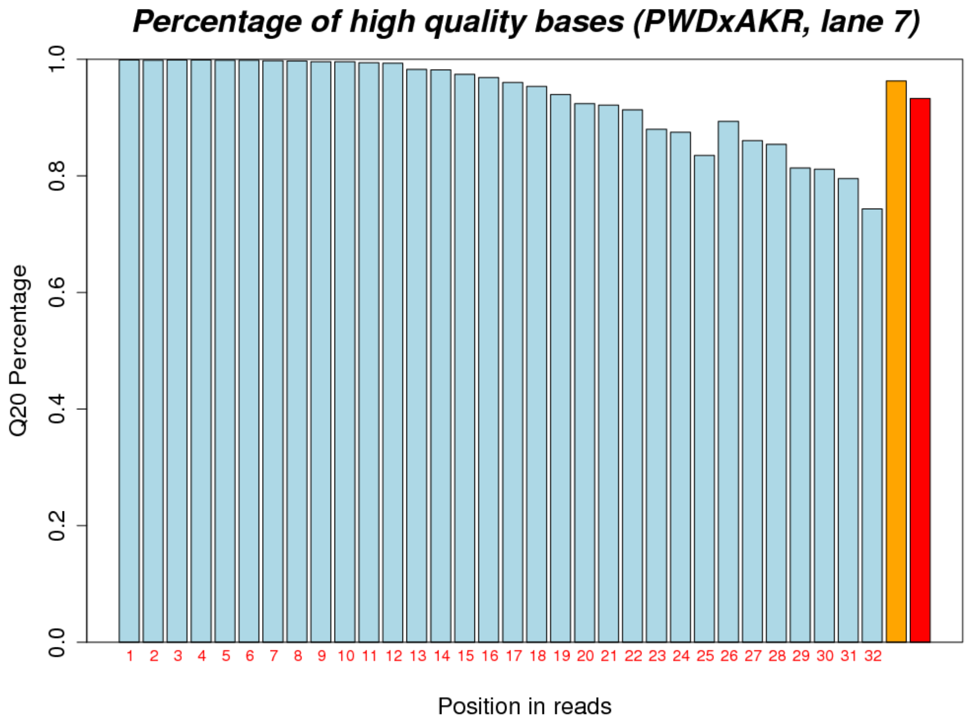

A. 7) Quality score for lane 8, PWD x AKR.

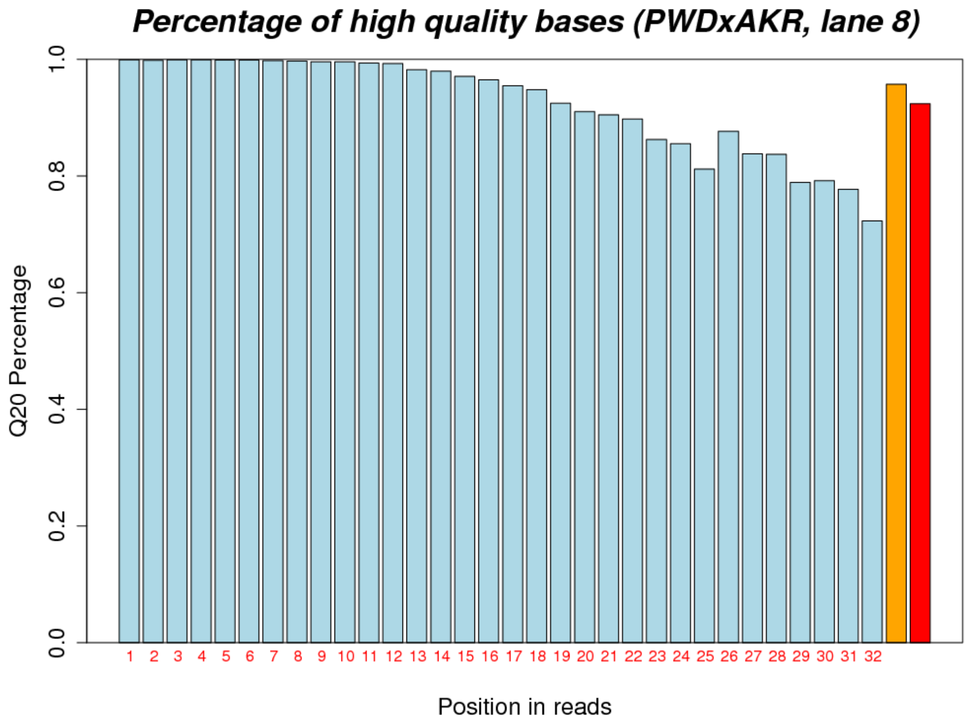

B. 1) Quality score for lane 1, AKR x PWD.

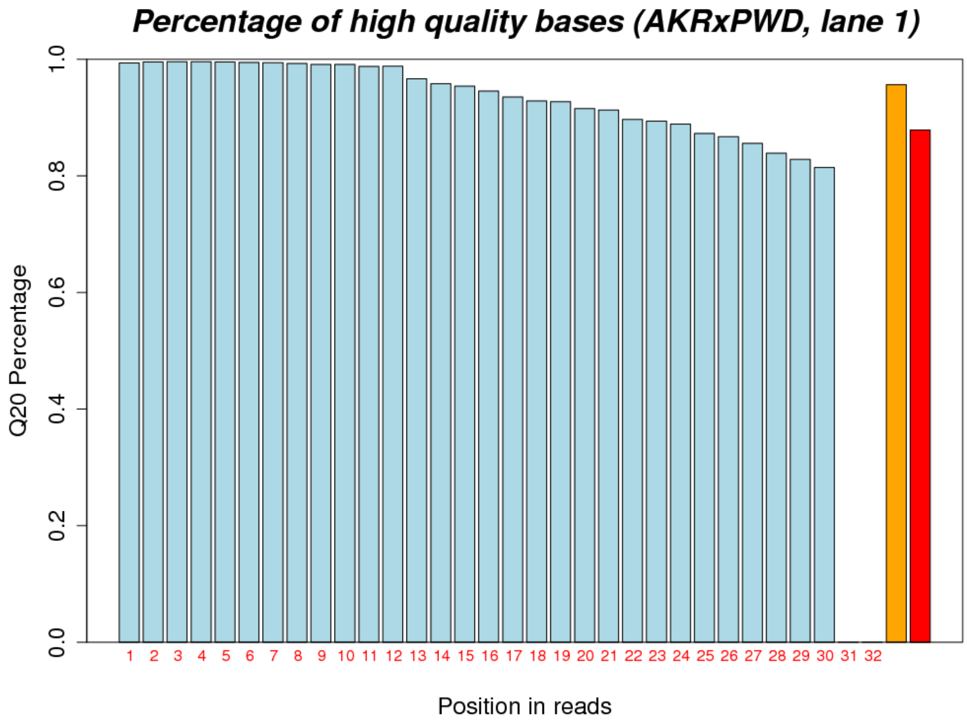

B. 2) Quality score for lane 2, AKR x PWD.

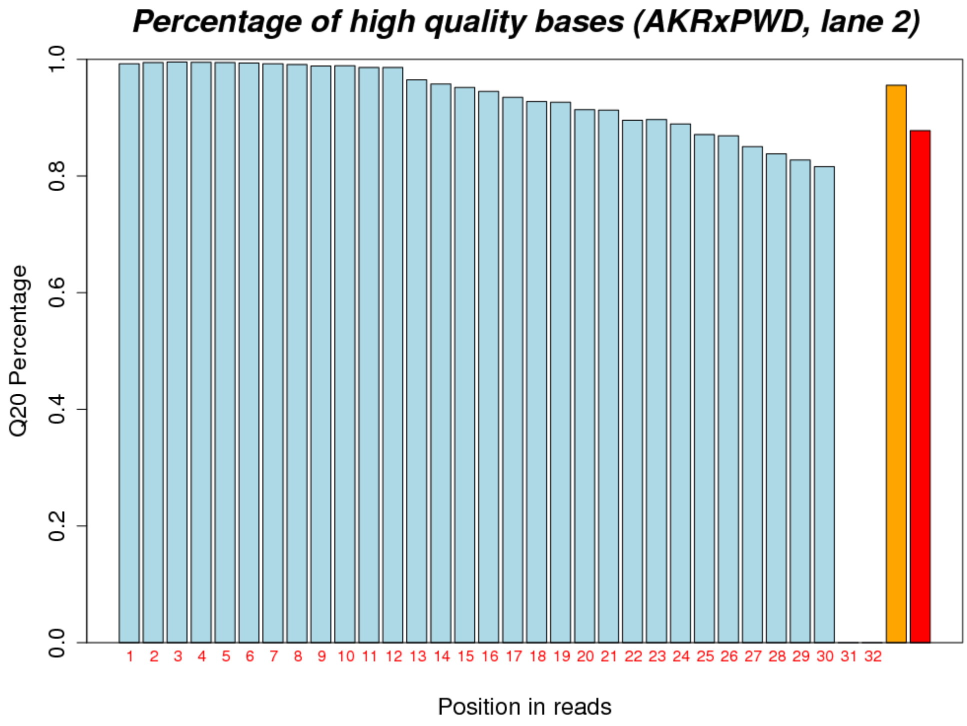

B. 3) Quality score for lane 3, AKR x PWD.

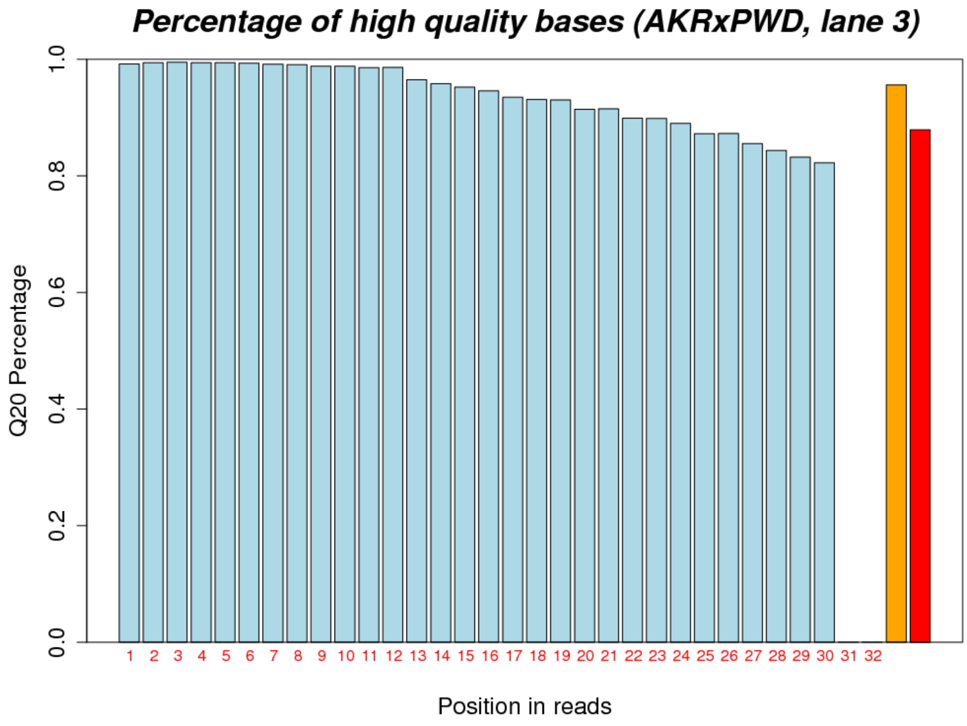

B. 4) Quality score for lane 4, AKR x PWD.

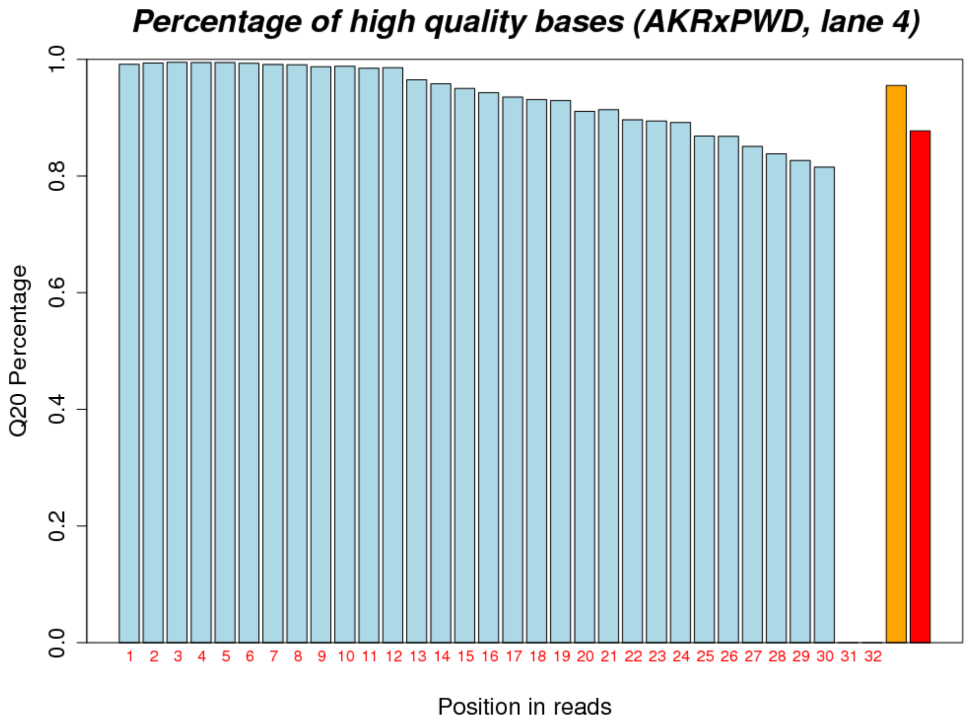

B. 5) Quality score for lane 5, AKR x PWD.

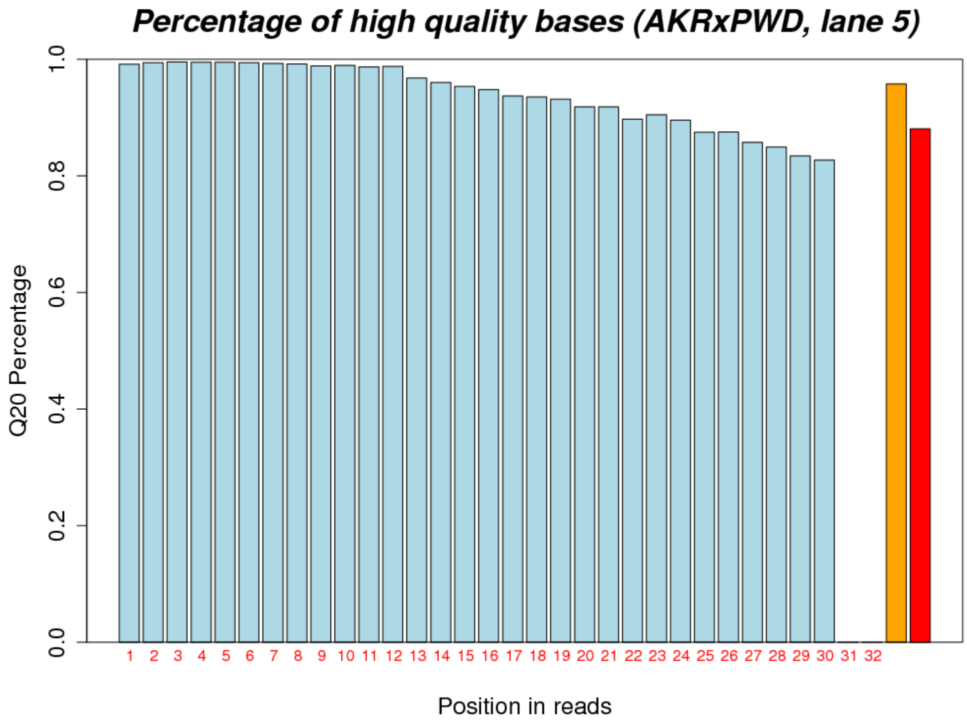

B. 6) Quality score for lane 6, AKR x PWD.

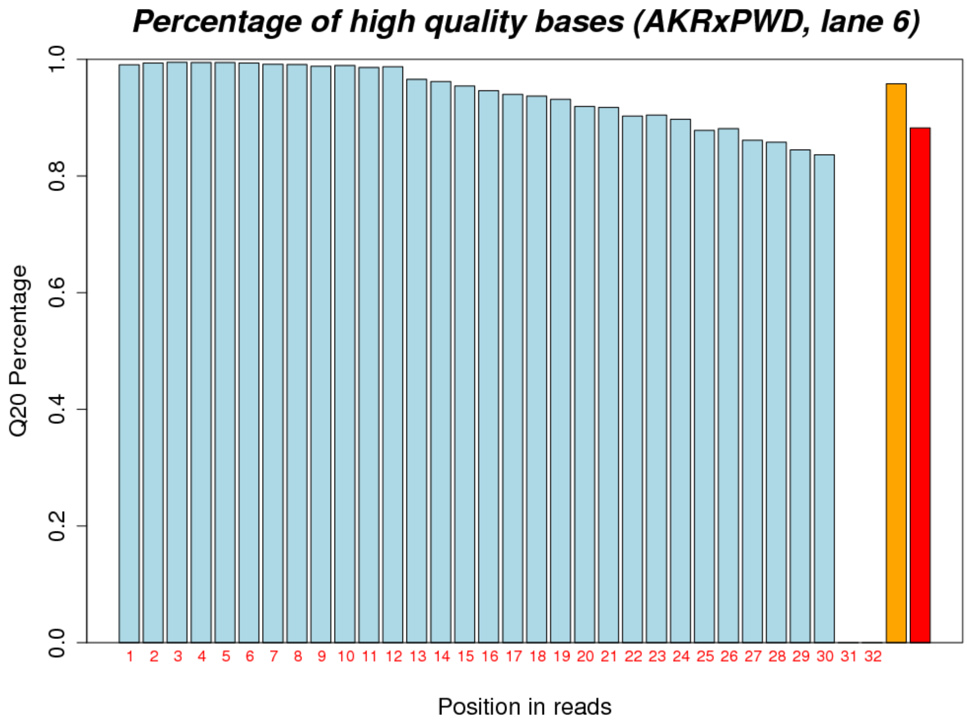

B. 7) Quality score for lane 7, AKR x PWD.

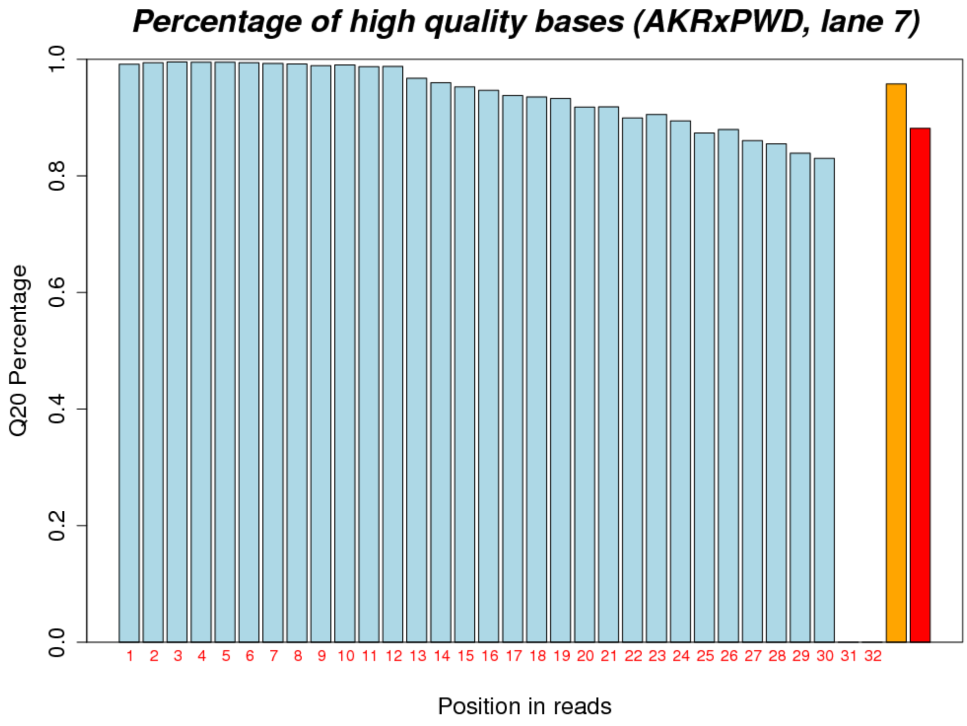

B. 8) Quality score for lane 8, AKR x PWD.

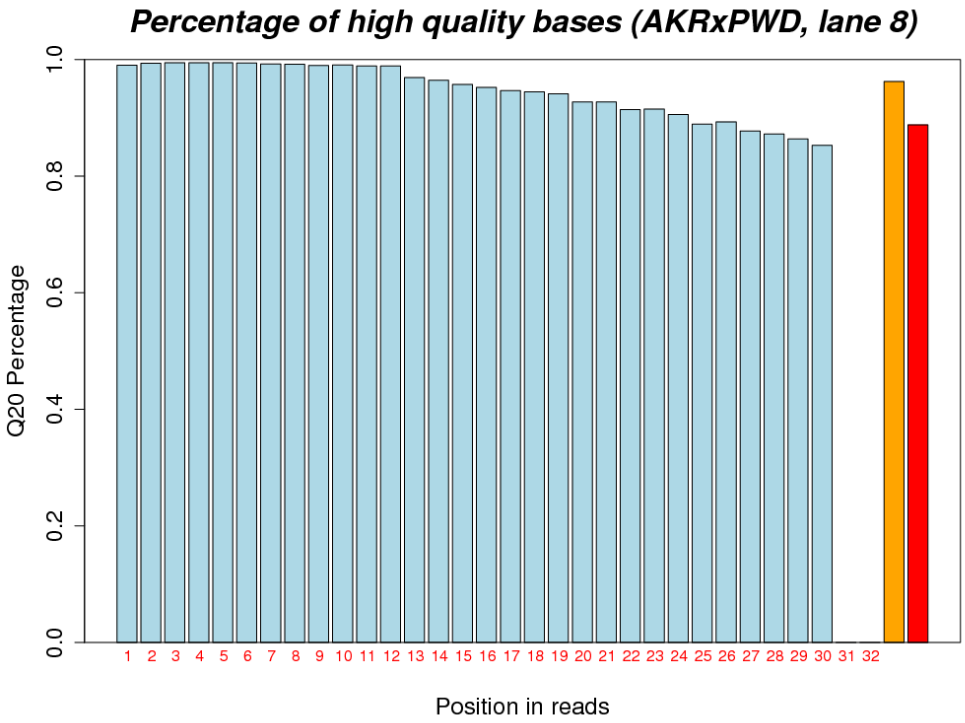

Figure S1.1. The quality score distribution of the Solexa data. (A). Quality score distribution for the seven lanes in the Solexa run of the PWD x AKR sample. (B). Quality score distribution for the eight lanes in the Solexa run of the AKR x PWD sample. Blue bar: position 1-32 in the Solexa reads. Orange bar: the first 25 bps. Red bar: all 32 bps.

Figure S1.2 Verification for known imprinted gene *Inpp5f_v2 and* novel imprinted gene *Inpp5f*.

A. SNP counts in the Solexa data.

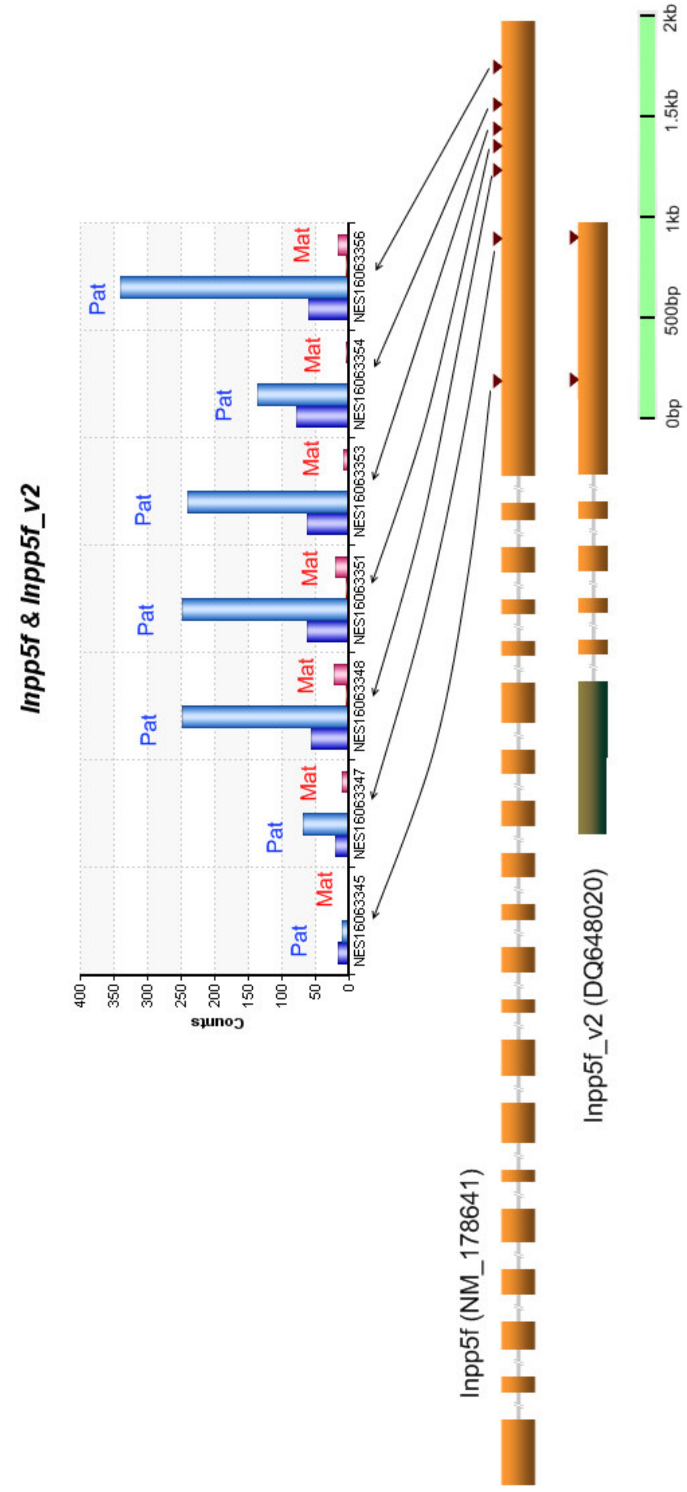

B. Sanger sequencing verification (NES16063356).

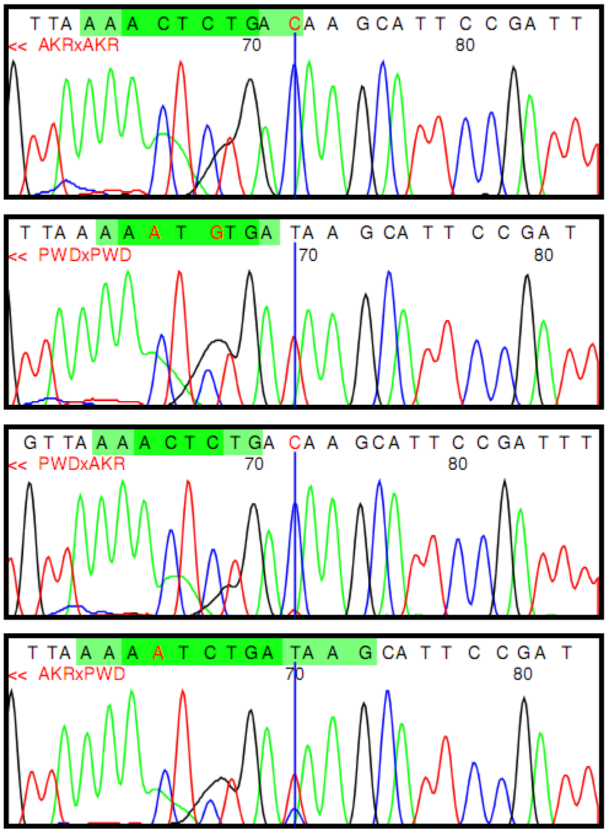

C. Pyrosequencing verification (NES16063356).

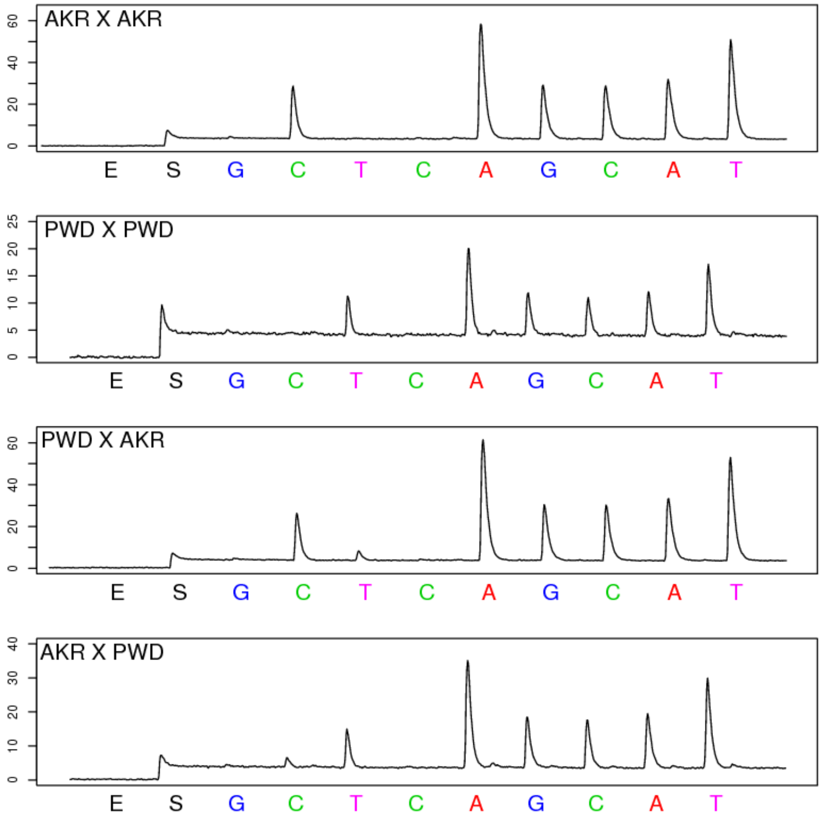

D. Pyrosequencing verification (NES16063345).

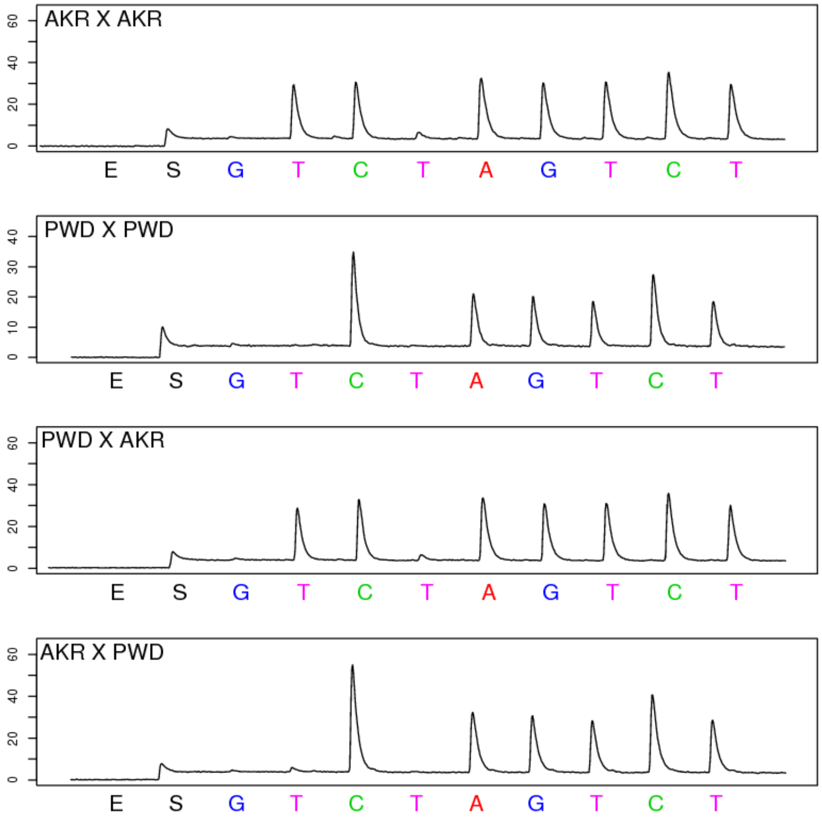

Figure S1.2. Verification for known imprinted gene *Inpp5f_v2 and* novel imprinted gene *Inpp5f*. A, Allele counts for Perlegen SNP NES16063345, NES16063347, NES16063348, NES16063351, NES16063353, NES16063354 and NES16063356. The blue bars (from left to right) stand for the counts from the paternal allele in PWD x AKR and AKR x PWD F1s respectively. The red bars represent the maternal allele counts. **B,** Sanger sequencing verification for Perlegen SNP NES16063356. The target sequence is CTCTGA(C/T)AAGCA. The only polymorphic site is the one with a blue straight line. The other one (the seventh nucleotide from the left) is a miscall from the software (please see the actual peak). **C,** Pyrosequencing sequencing verification for Perlegen SNP NES16063356. The target sequence is CTCTGA(C/T)AAGCA. **D,** Pyrosequencing sequencing verification for Perlegen SNP NES16063345. The target sequence is CGGTC(C/T)CAGTCT.

Figure S1.3. Verification for known imprinted gene *Rasgrf1* (Novel SNP1).

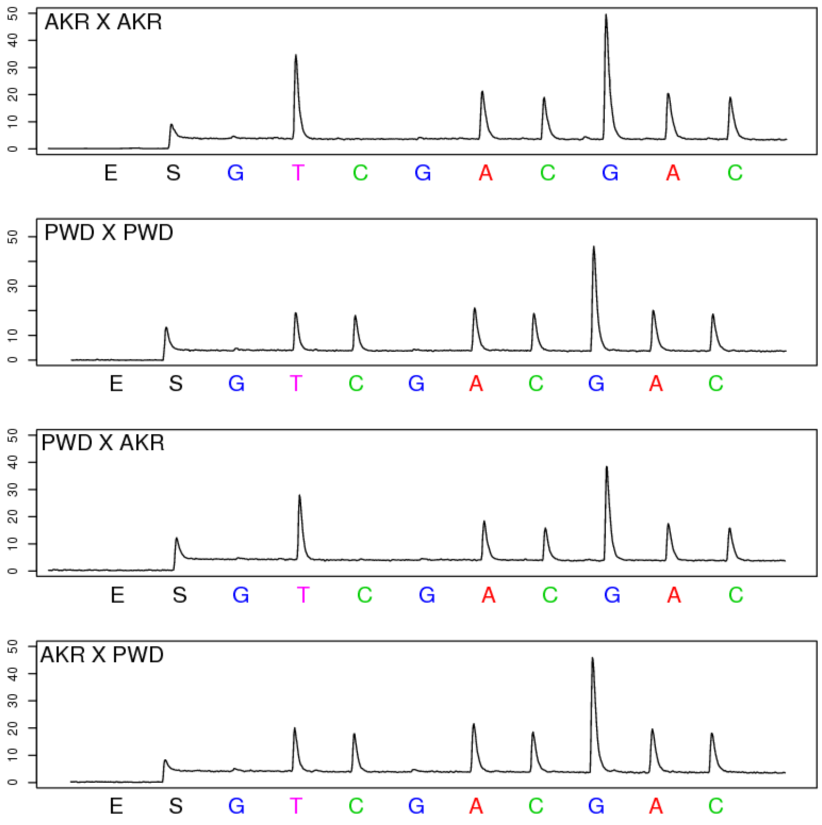

Figure S1.3. Verification for known imprinted gene *Rasgrf1*. Pyrosequencing verification for Novel SNP1 in *Rasgrf1*. The target sequence is T(C/T)ACGGGACAA.

Figure S1.4. Verification for known imprinted gene *Zrsr1*.

A. Sanger sequencing verification (NES08366940).

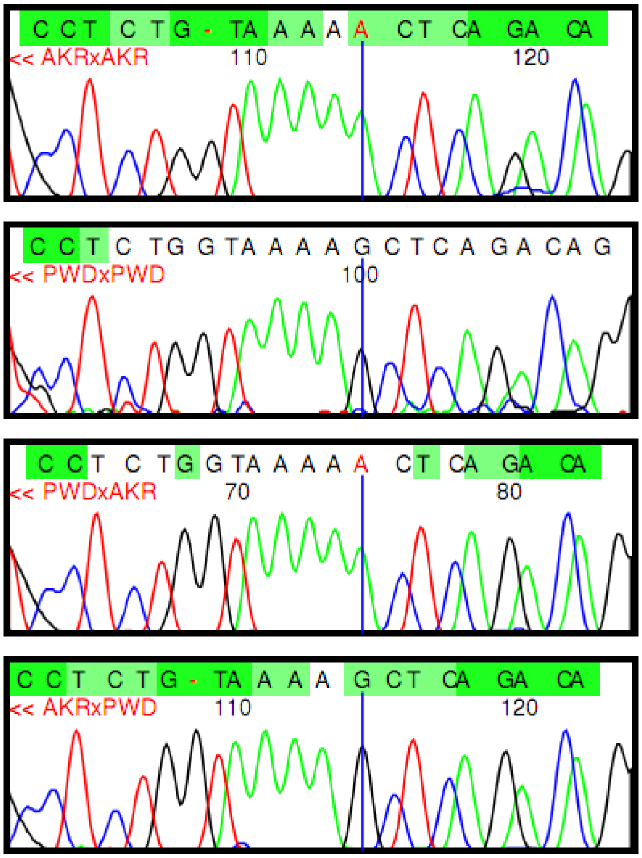

B. Pyrosequencing verification (NES08366940).

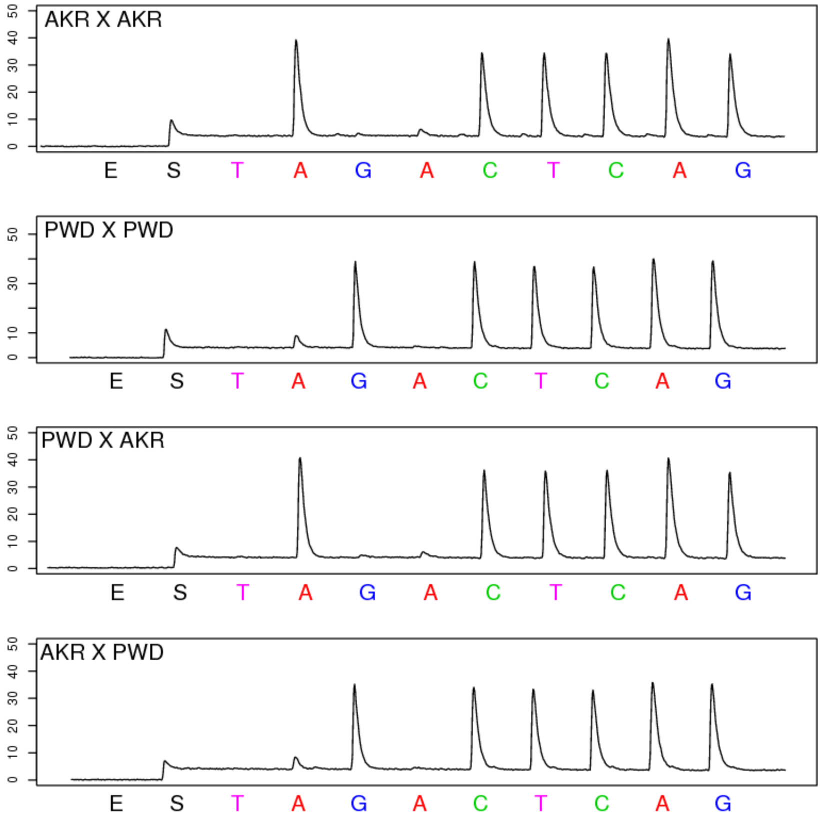

Figure S1.4. Verification for known imprinted gene *Zrsr1*. A, Sanger sequencing verification for Perlegen SNP NES08366940. The target sequence is GGTAAAA(A/G)CTCAGA. The only polymorphic site is the one with a blue straight line. The other one (the seventh nucleotide from the left) is a miscall from the software (please see the actual peak). **B,** Pyrosequencing sequencing verification for Perlegen SNP NES08366940. The target sequence is (A/G)CTCAGAC.

Figure S1.5. Verification for known imprinted genes *Snrpn* and *Snurf* (NES16116930).

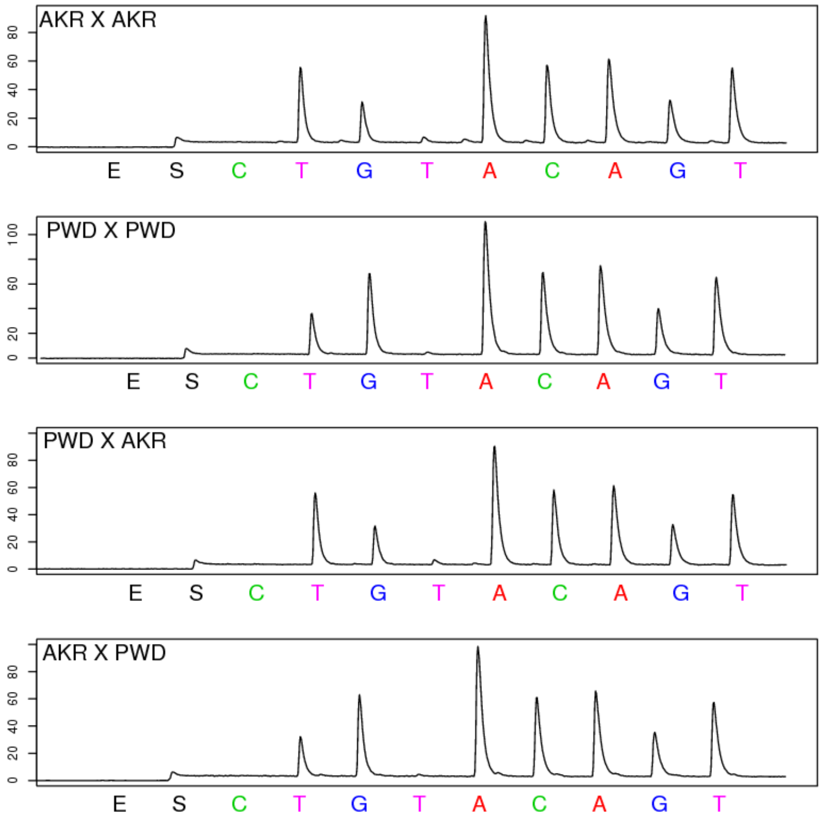

Figure S1.5. Verification for known imprinted gene *Snrpn and Snurf.* Pyrosequencing sequencing verification for Perlegen SNP NES16116930. The target sequence is (G/T)GAAACCAAGTTCT.

Figure S1.6. Pyrosequencing verification of *Peg13* (Novel SNP3).

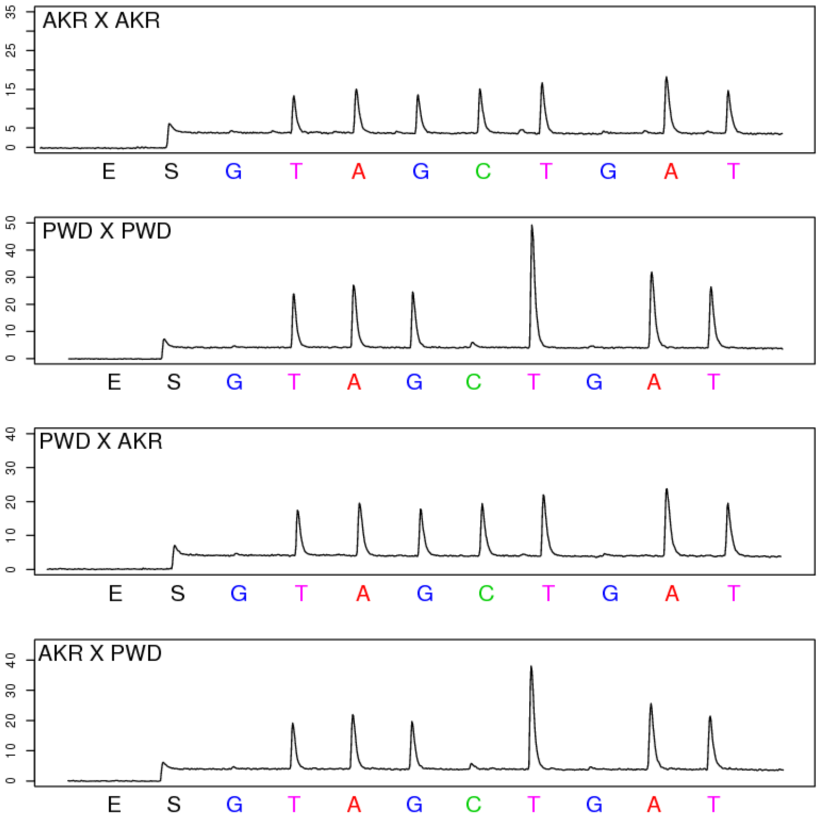

Figure S1.6. Verification for known imprinted gene *Peg13*. Pyrosequencing sequencing verification for novel SNP3 in *Peg13*. The target sequence is TAG(C/T)TATAG.

Figure S1.7. Pyrosequencing verification of *Sgce*.

A. Sanger sequencing verification (NES10338539).

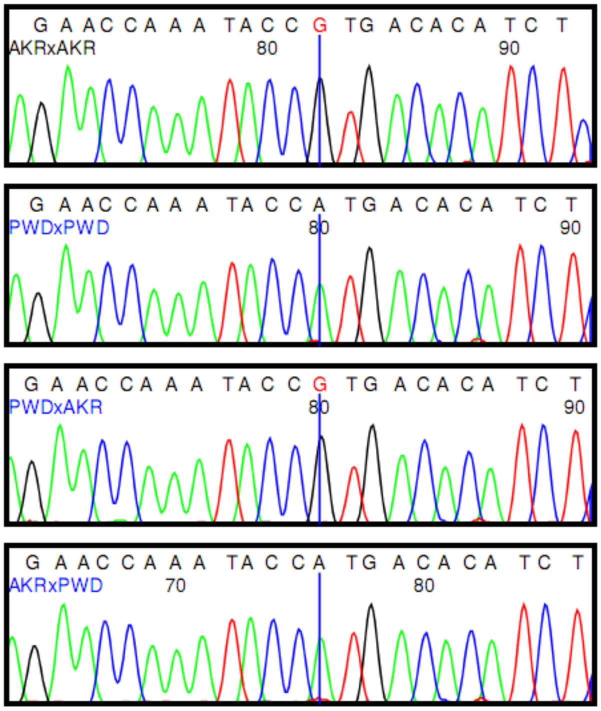

B. Pyrosequencing verification (NES10338539).

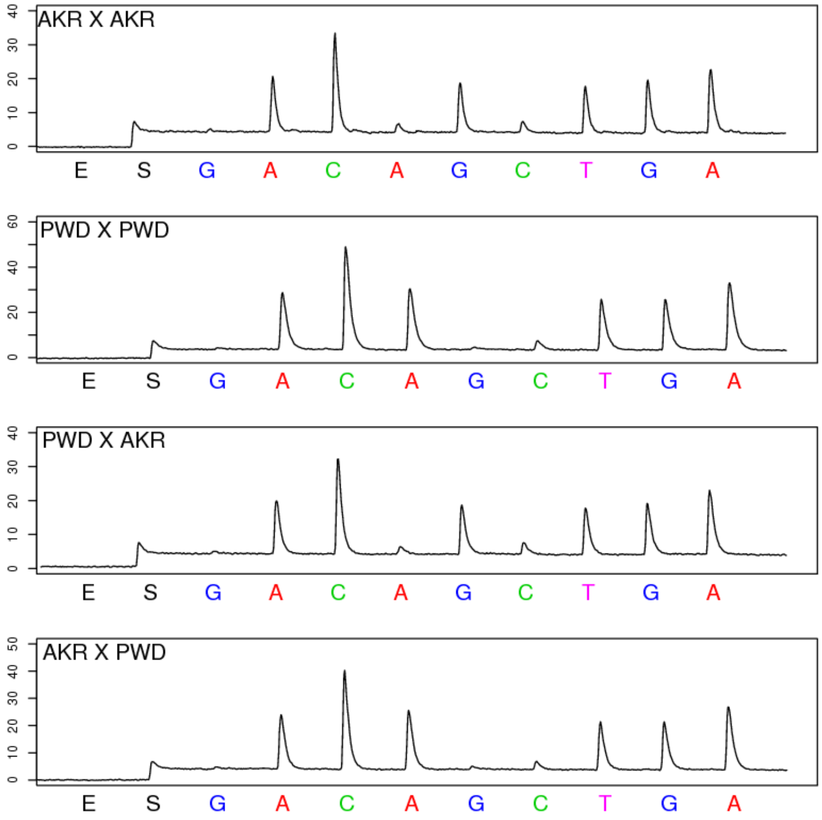

Figure S1.7. Verification for known imprinted gene *Sgce*. A, Sanger sequencing verification for Perlegen SNP NES10338539. The target sequence is ACC(G/A)TGACACA. **B,** Pyrosequencing sequencing verification for Perlegen SNP NES10338539. The target sequence is ACC(G/A)TGACACA.

Figure S1.8. Pyrosequencing verification of *Nap1l5* (Novel SNP1).

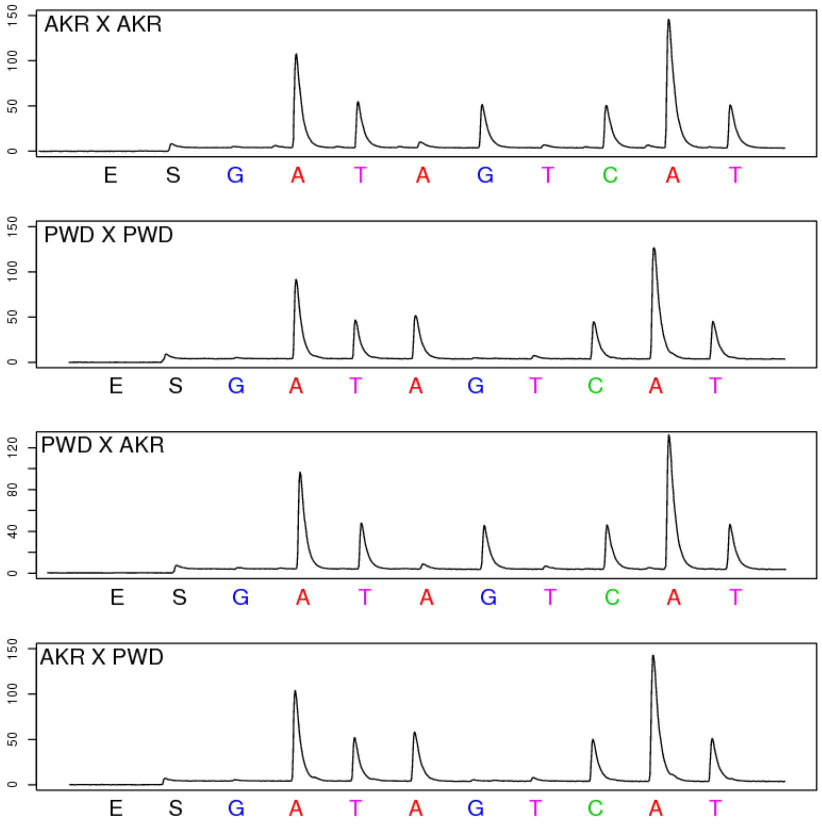

Figure S1.8. Verification for known imprinted gene *Nap1l5*. Pyrosequencing sequencing verification for novel SNP1 in Nap1l5. The target sequence is AAT(A/G)CAAATATTTA.

Figure S1.9. Pyrosequencing verification of *Impact* (NES12698107).

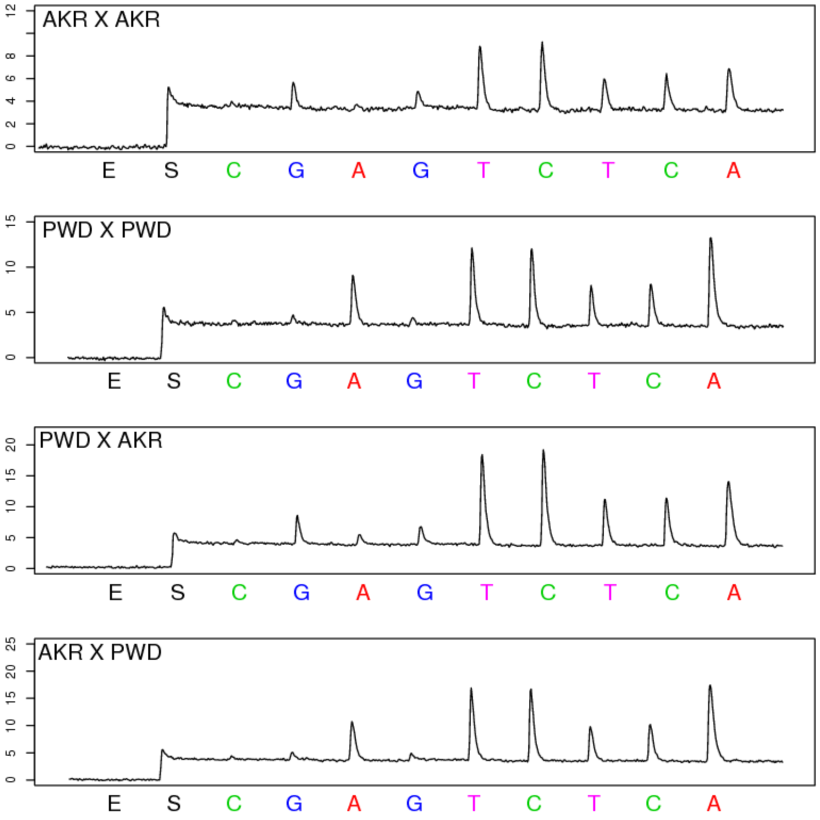

Figure S1.9. Verification for known imprinted gene *Impact*. Pyrosequencing sequencing verification for Perlegon SNP NES12698107. The target sequence is (G/A)TTCCTCAC.

Figure S1.10. Pyrosequencing verification of *H19* and *Igf2*.

A. Pyrosequencing verification of *H19* (Novel SNP1).

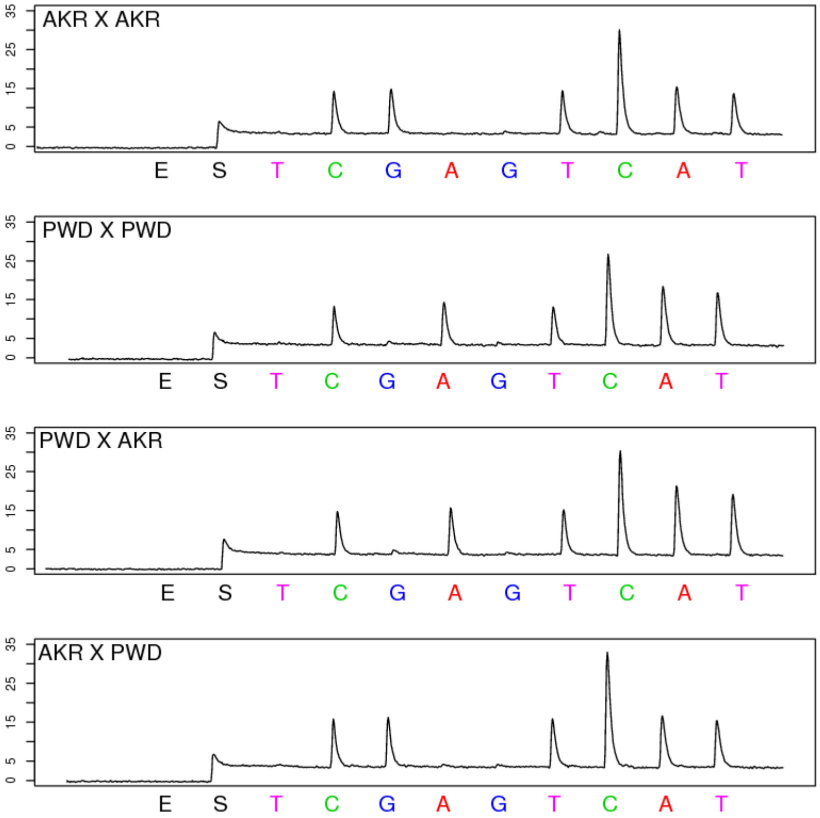

B. Pyrosequencing verification of *Igf2* (Novel SNP1).

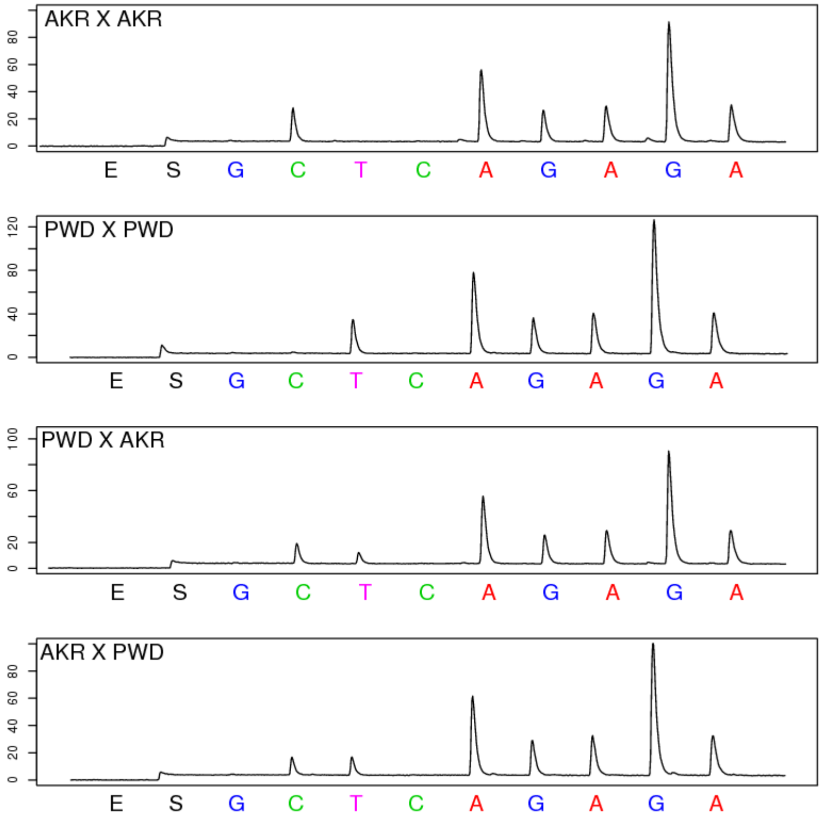

Figure S1.10. Verification for known imprinted gene *H19 and Igf2*. A, Pyrosequencing sequencing verification for novel SNP1 in *H19*. The target sequence is C(G/A)TCCATC. **B,** Pyrosequencing sequencing verification for new SNP1 in *Igf2*. The target sequence is (C/T)AAGAGGGGAT.

Figure S1.11. Pyrosequencing verification of *Cdkn1c* (Novel SNP1).

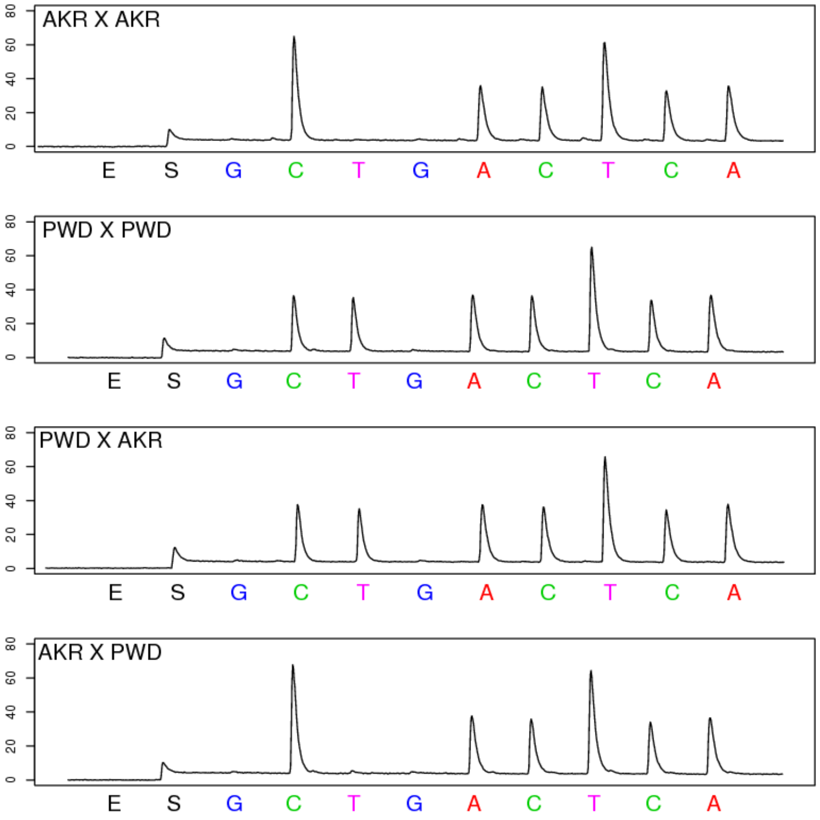

Figure S1.11. Verification for known imprinted gene *Cdkn1c*. Pyrosequencing sequencing verification for novel SNP1 in *Cdkn1c*. The target sequence is C(C/T)ACTTCAT .

Figure S1.12. Verification for novel imprinted gene *1810044A24Rik*.

A. Sanger sequencing verification (NES12099717).

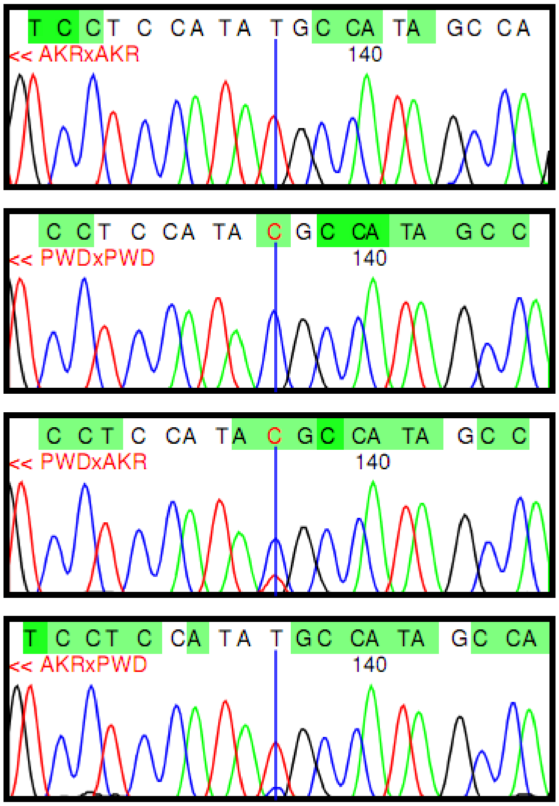

B. Pyrosequencing verification in AKR and PWD reciprocal cross (NES12097854).

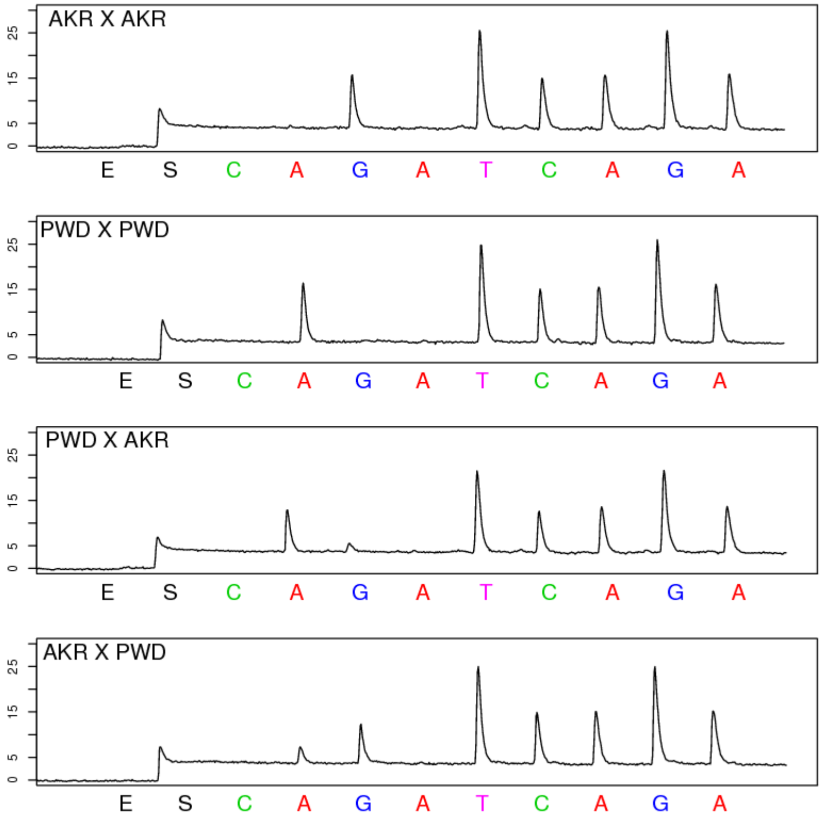

C. Pyrosequencing verification in C3H and B6 reciprocal cross (NES12098495).

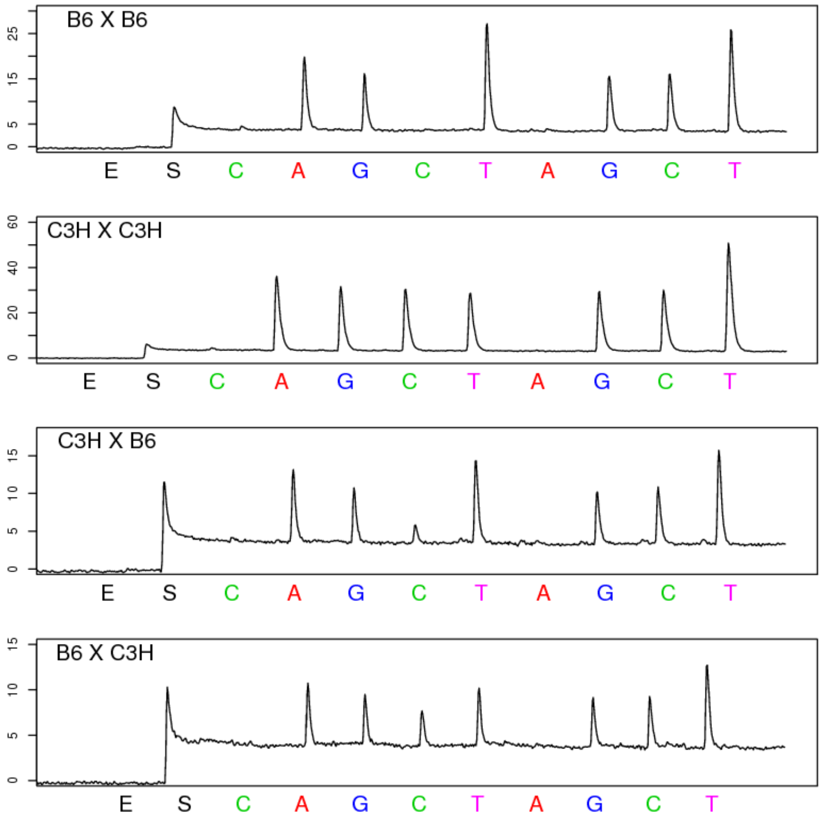

Figure S1.12. Verification for known imprinted gene *1810044A24Rik*. A, Sanger sequencing verification for Perlegen SNP NES12099717. The target sequence is TCCATA(T/C)GCCATA. **B,** Pyrosequencing sequencing verification for Perlegen SNP NES12097854 in AKR and PWD reciprocal cross. The target sequence is (A/G)TTCAGGA. **C,** Pyrosequencing sequencing verification for Perlegen SNP NES12098495 in C3H and B6 reciprocal cross. The target sequence is AG(C/T)TGCTT.

Figure S1.13. Verification for novel imprinted gene *Blcap*.

A. Pyrosequencing verification (NES08901938).

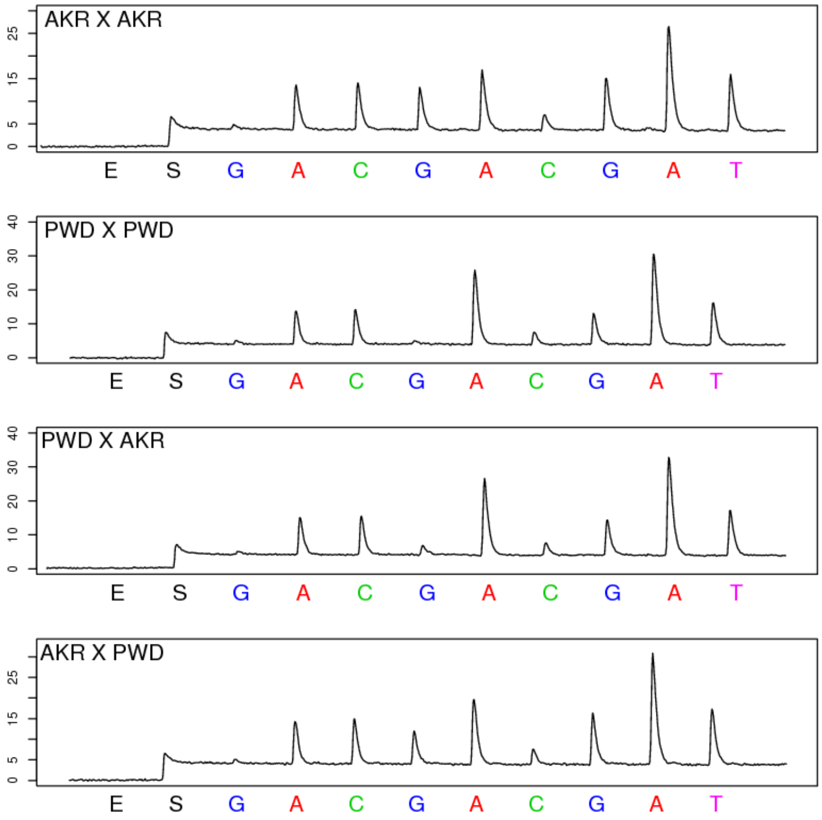

B. Pyrosequencing verification (NES08901938, using different PCR and Pyrosequencing primers).

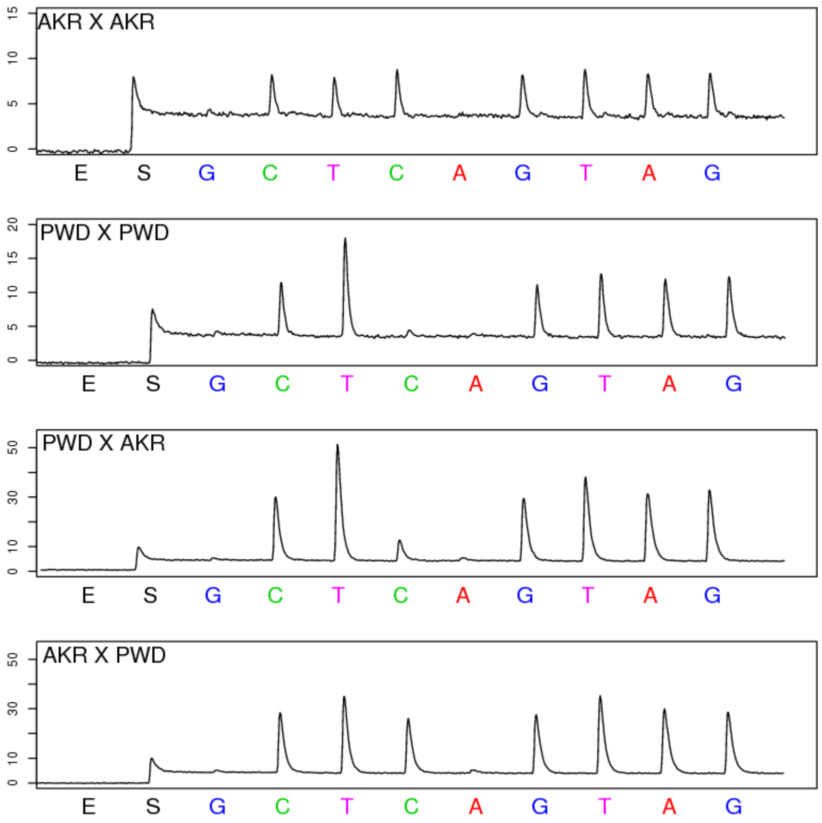

Figure S1.13. Verification for known imprinted gene *Blcap*. A, Pyrosequencing sequencing verification for Perlegen SNP. The target sequence is AC(A/G)AGAATA. **B,** Pyrosequencing sequencing verification for Perlegen SNP NES12098495. The target sequence is CT(C/T)GTAGA.

Figure S1.14.. Genome-wide plot of imprinting status.

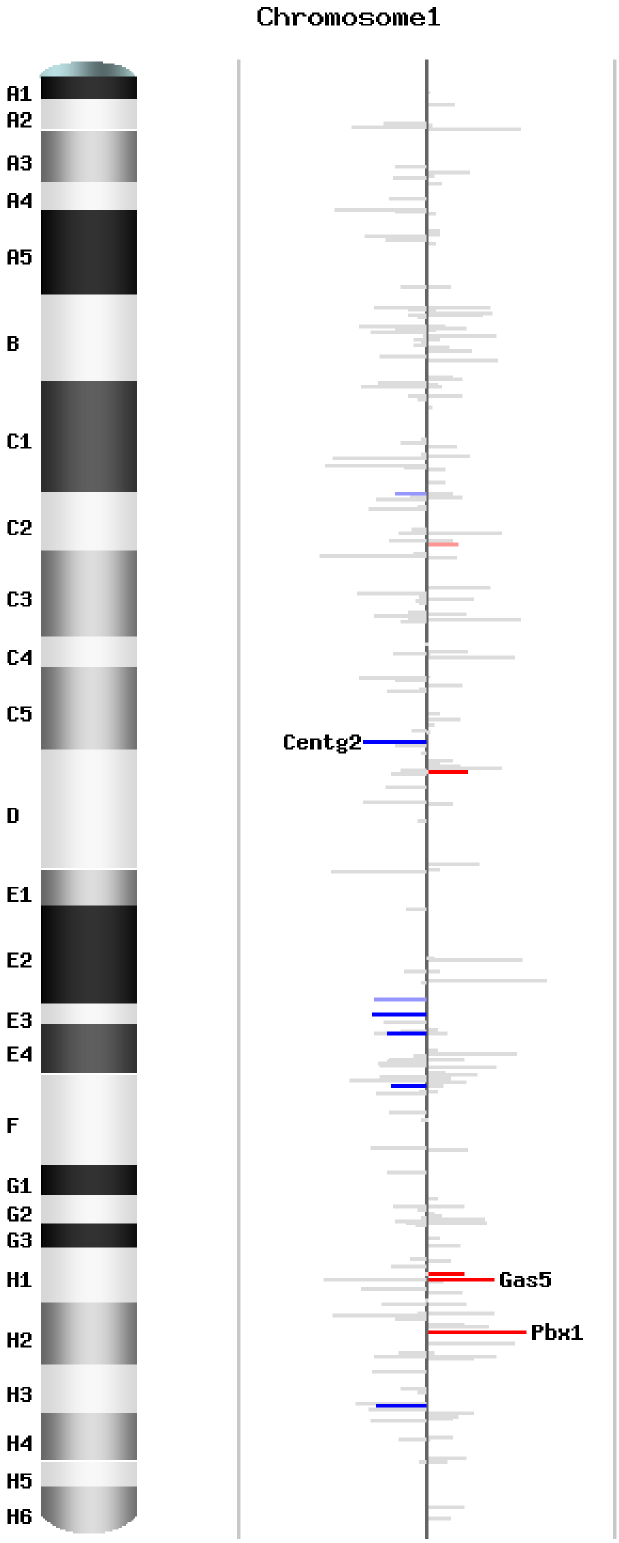

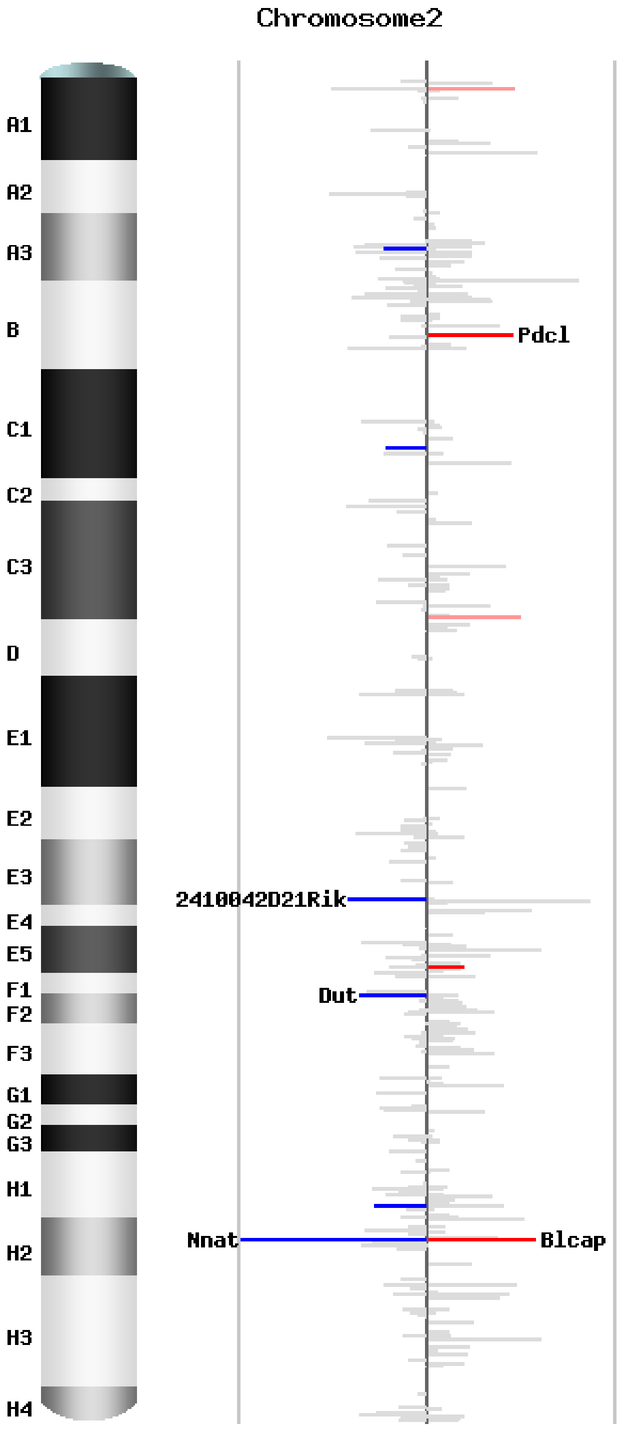

Chromosome2
A1
A2
A3
B
C1
C2
C3
D
E1
E2
E3
E4
E5
F1
F2
F3
G1
G2
G3
H1
H2
H3
H4
Pdcl
2410042D21Rik
Dut
Nnat
Blcap

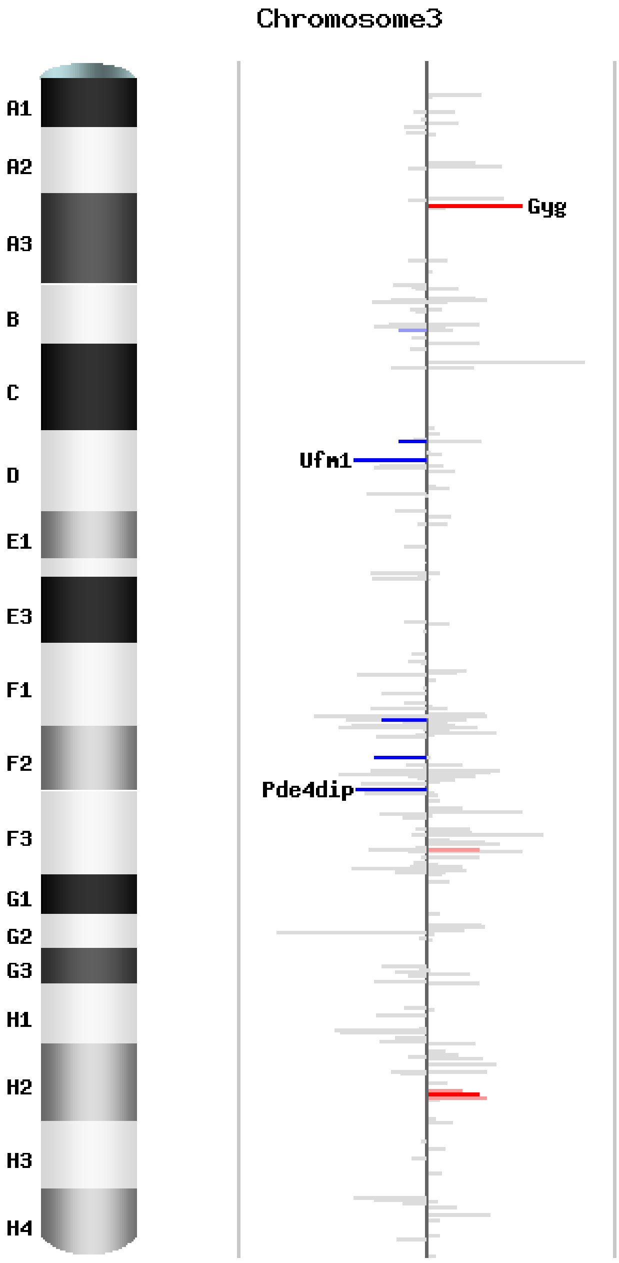
Chromosome3
A1
A2
A3
B
C
D
E1
E3
F1
F2
F3
G1
G2
G3
H1
H2
H3
H4
Gyg
Ufm1
Pde4dip

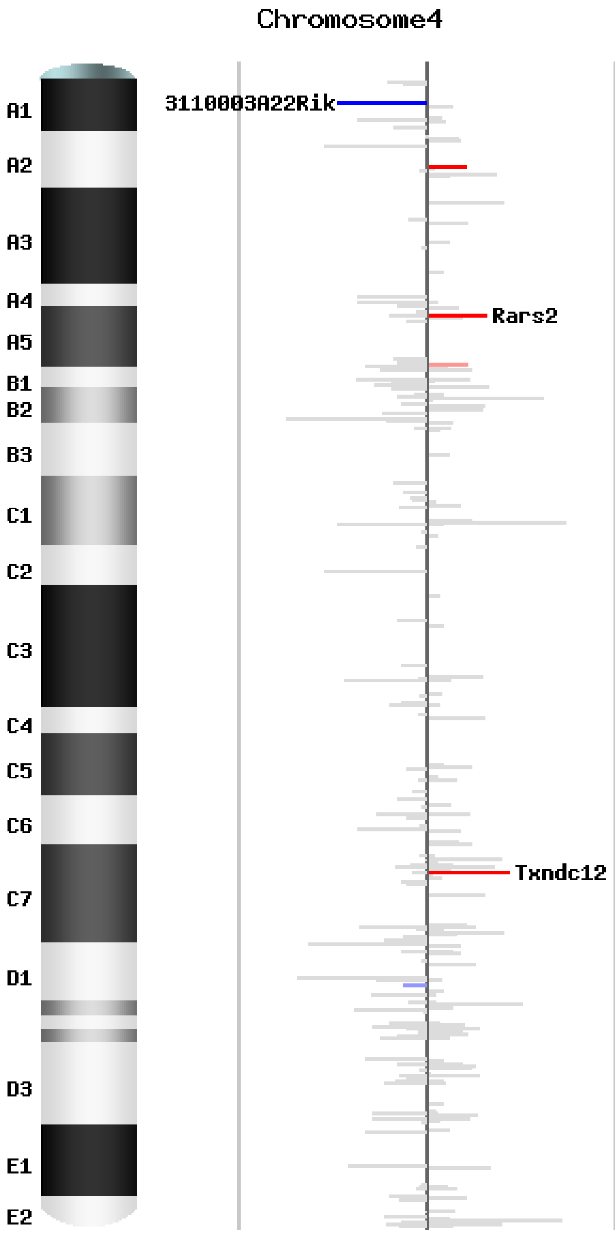
Chromosome4
A1
A2
A3
A4
A5
B1
B2
B3
C1
C2
C3
C4
C5
C6
C7
D1
D3
E1
E2
3110003A22Rik
Rars2
Txndc12

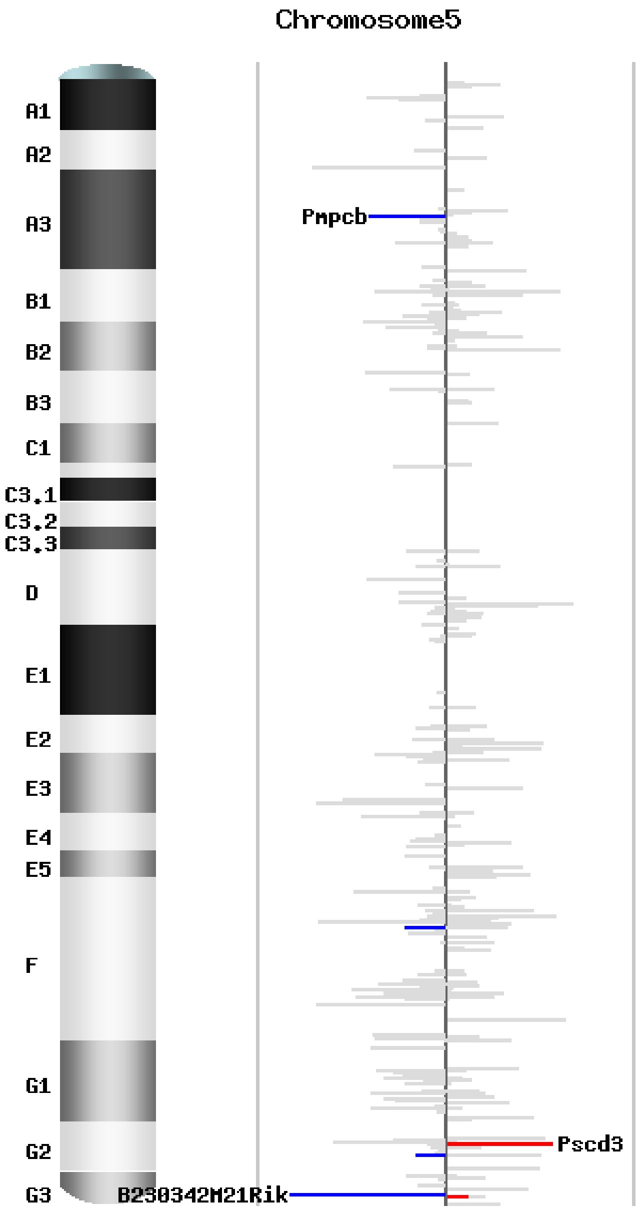
Chromosome5
A1
A2
A3
B1
B2
B3
C1
C3.1
C3.2
C3.3
D
E1
E2
E3
E4
E5
F
G1
G2
G3
Pmpcb
Pscd3
B230342M21Rik

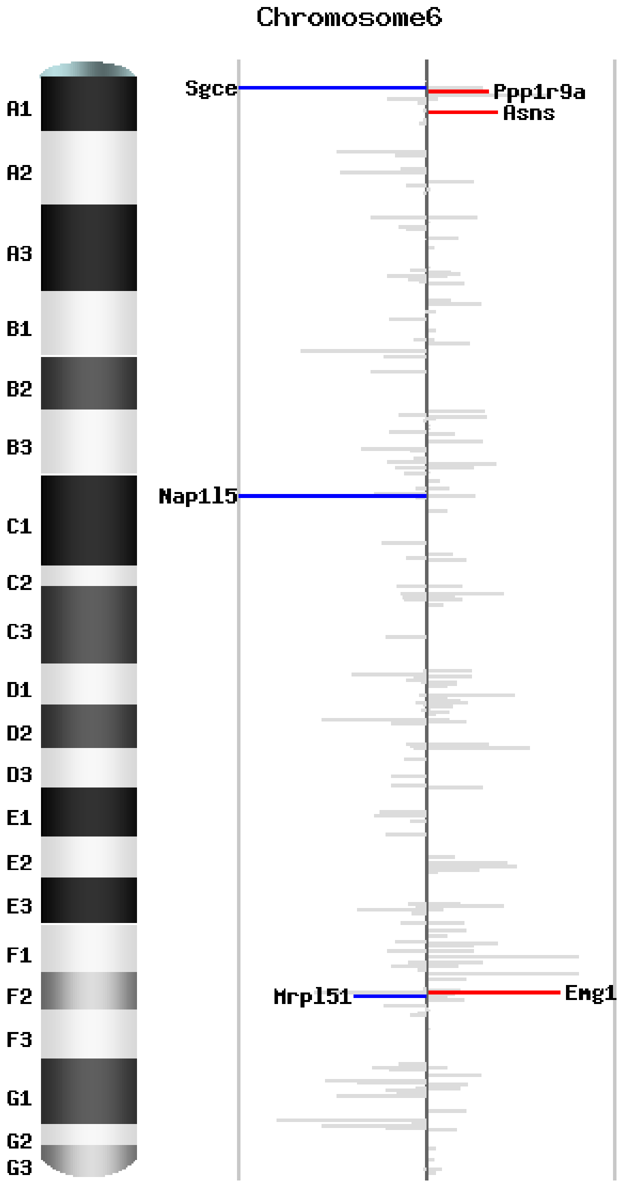
Chromosome6
A1
A2
A3
B1
B2
B3
C1
C2
C3
D1
D2
D3
E1
E2
E3
F1
F2
F3
G1
G2
G3
Sgce
Ppp1r9a
Asns
Nap1l5
Mrpl51
Emg1

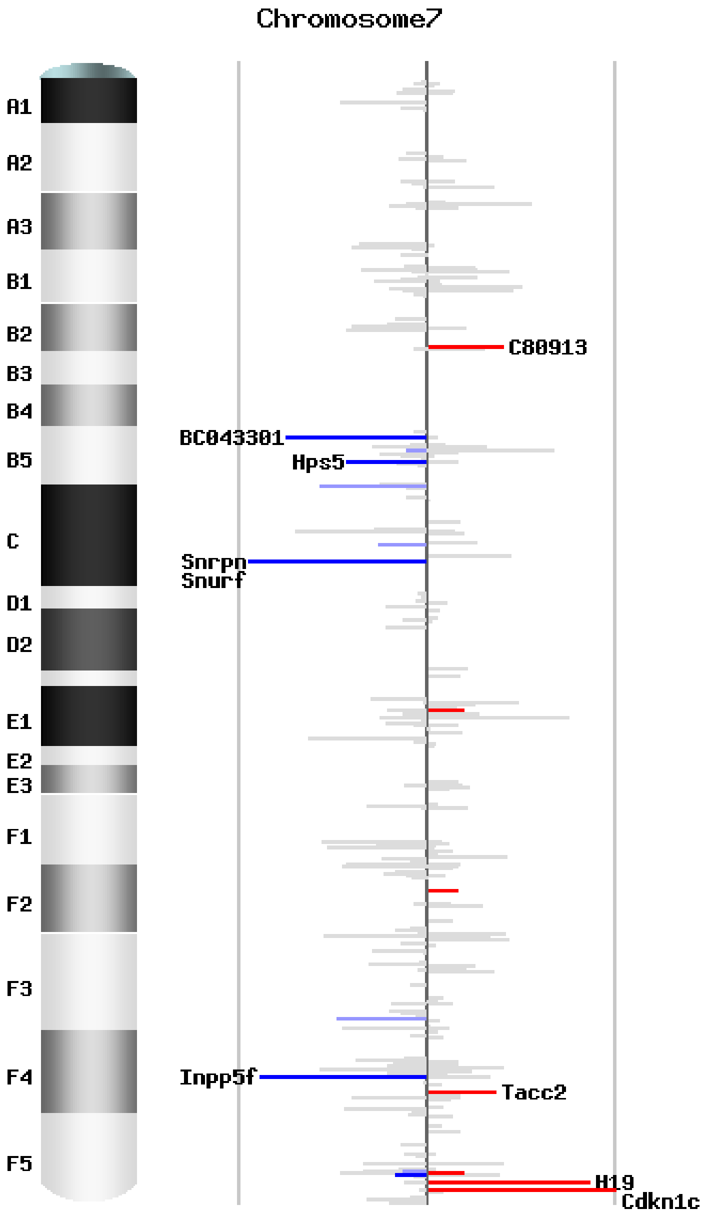
Chromosome7
A1
A2
A3
B1
B2
B3
B4
B5
C
D1
D2
E1
E2
E3
F1
F2
F3
F4
F5
C80913
BC043301
Hps5
Snrpn
Snurf
Inpp5f
Tacc2
H19
Cdkn1c

Chromosome8

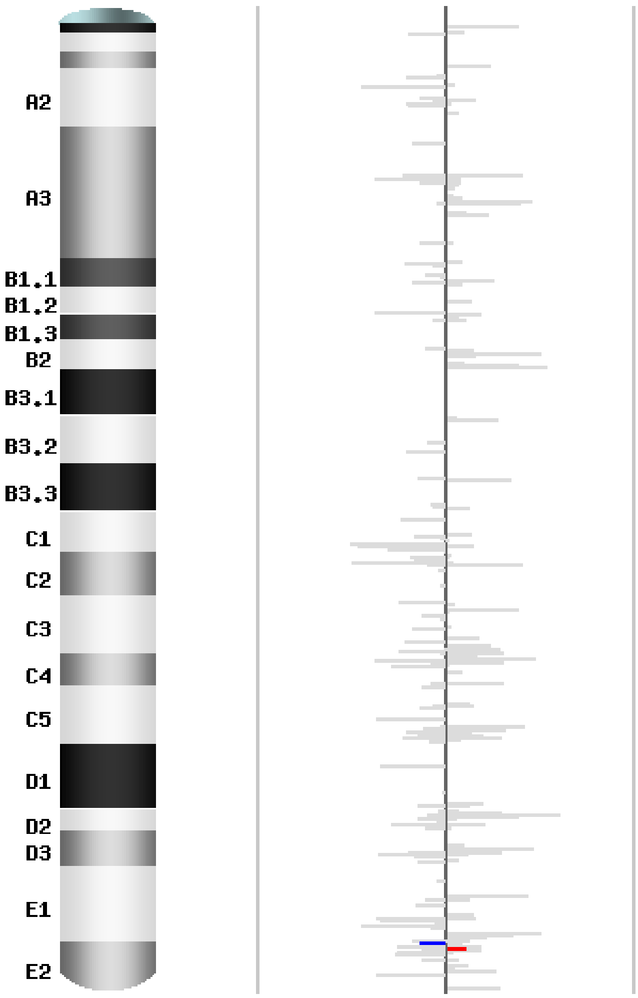

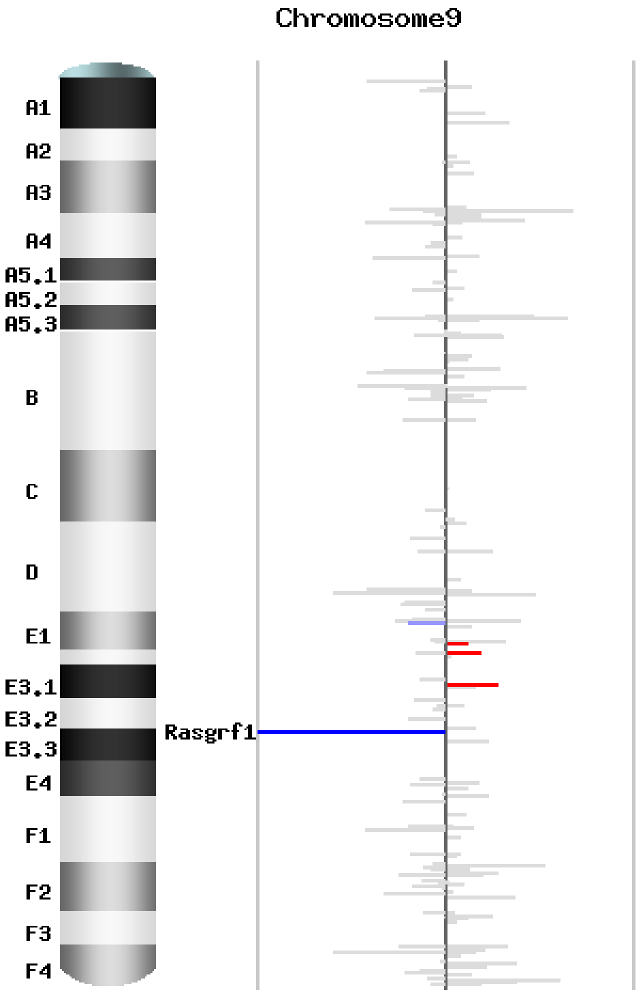

Chromosome9
A1
A2
A3
A4
A5.1
A5.2
A5.3
B
C
D
E1
E3.1
E3.2
Rasgrf1
E3.3
E4
F1
F2
F3
F4

Chromosome10

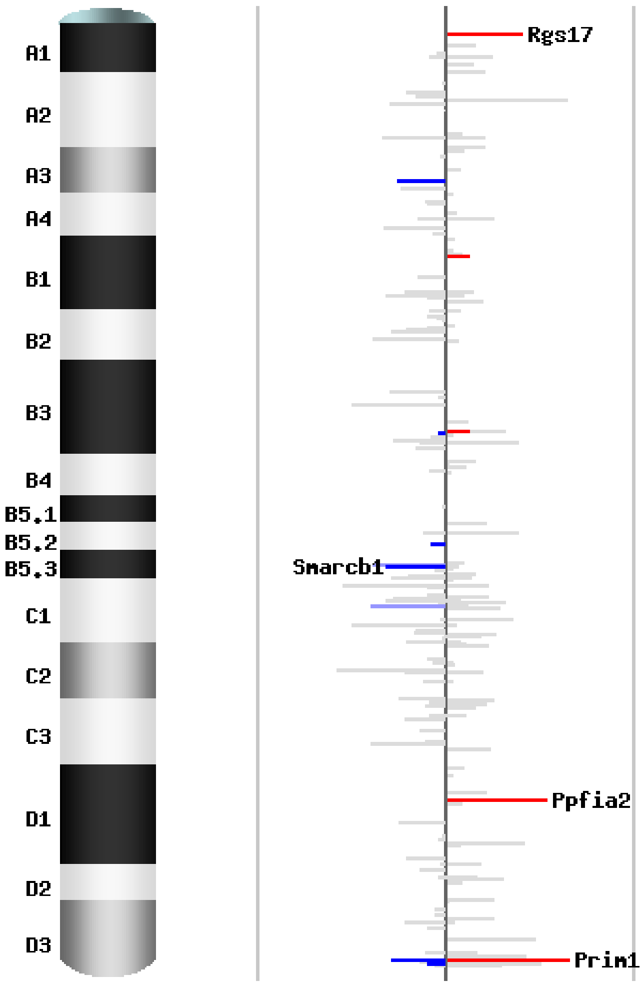

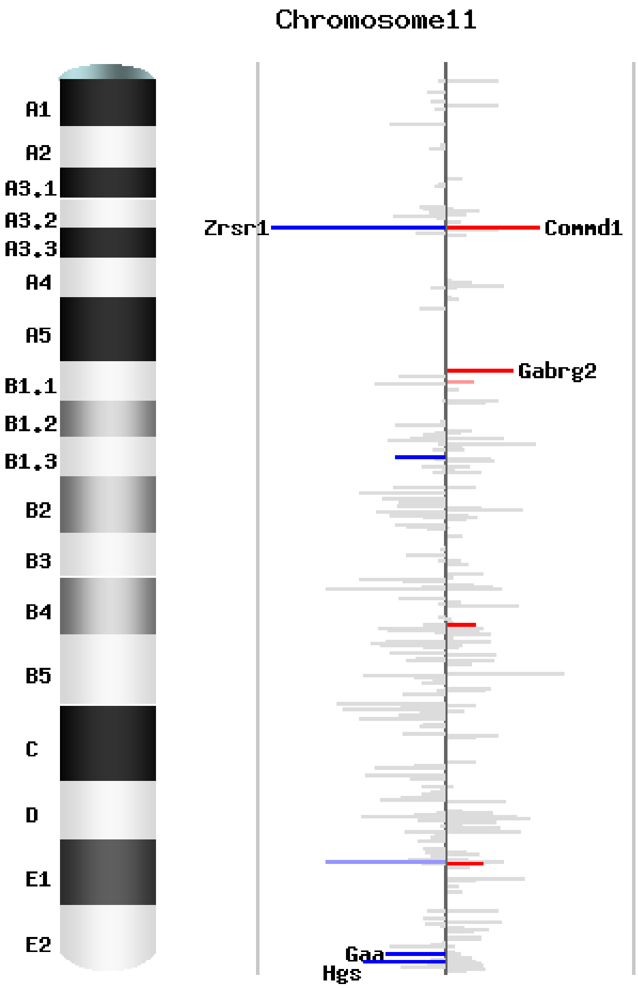

Chromosome11
A1
A2
A3.1
A3.2
A3.3
A4
A5
B1.1
B1.2
B1.3
B2
B3
B4
B5
C
D
E1
E2
Zrsr1
Commd1
Gabrg2
Gaa
Hgs

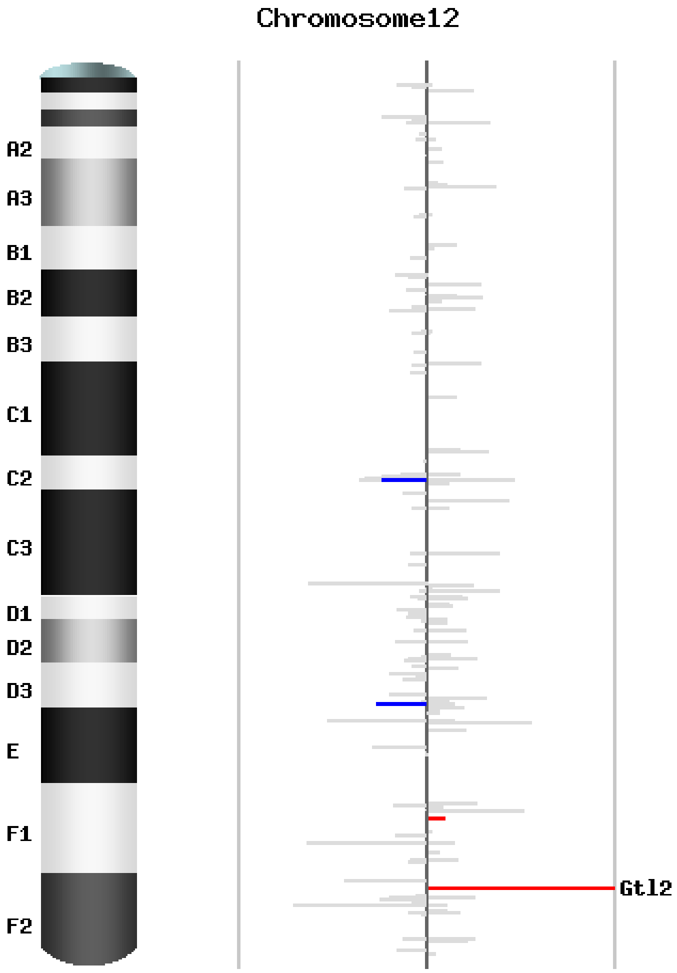

Chromosome12
A2
A3
B1
B2
B3
C1
C2
C3
D1
D2
D3
E
F1
F2
Gtl2

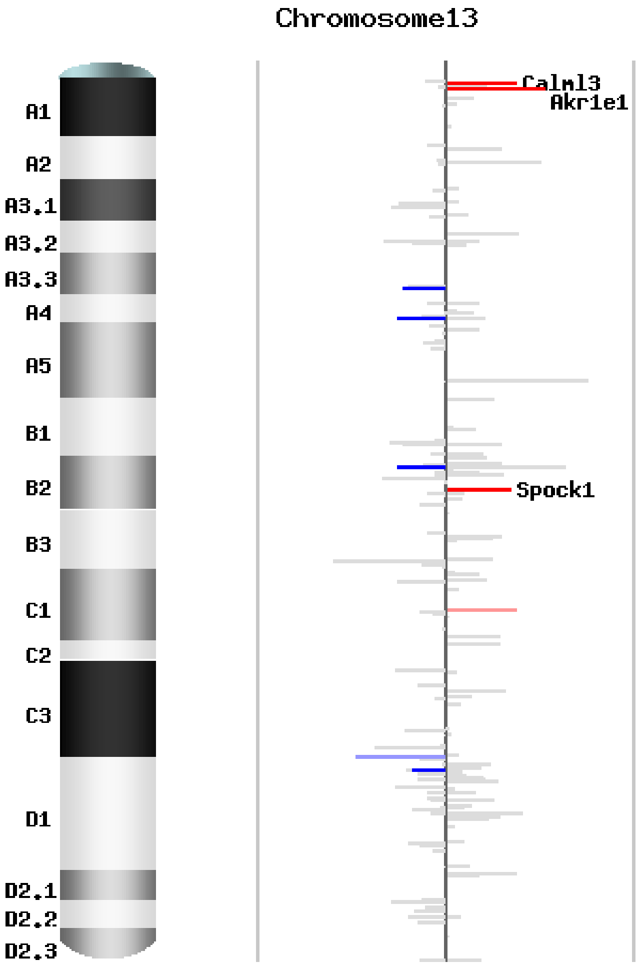

Chromosome13
Calml3
Akr1e1
Spock1
A1
A2
A3.1
A3.2
A3.3
A4
A5
B1
B2
B3
C1
C2
C3
D1
D2.1
D2.2
D2.3

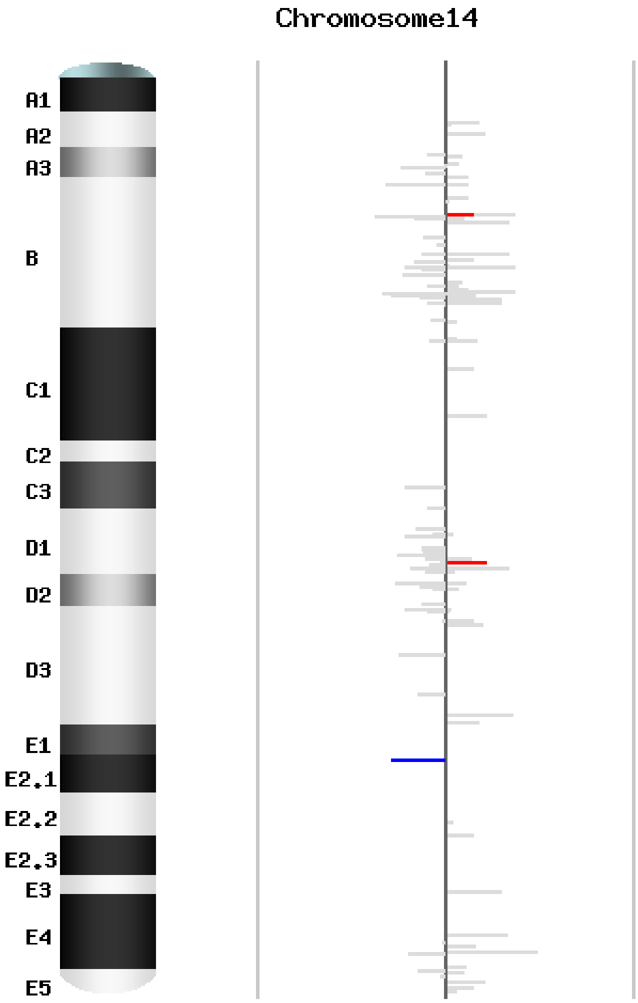
Chromosome14
A1
A2
A3
B
C1
C2
C3
D1
D2
D3
E1
E2.1
E2.2
E2.3
E3
E4
E5

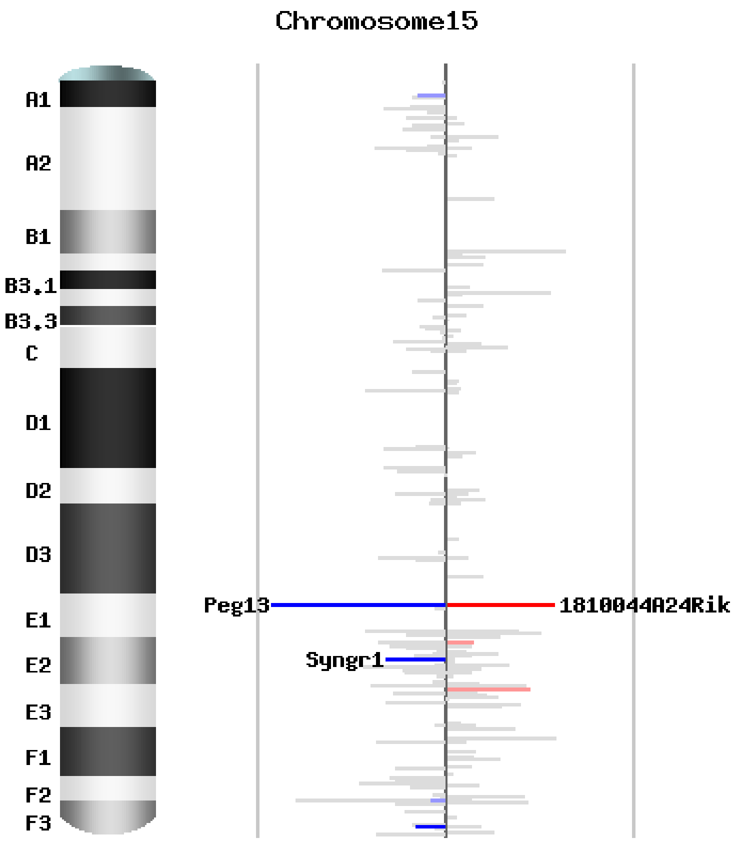
Chromosome15
A1
A2
B1
B3.1
B3.3
C
D1
D2
D3
E1
E2
E3
F1
F2
F3
Peg13
1810044A24Rik
Syngr1

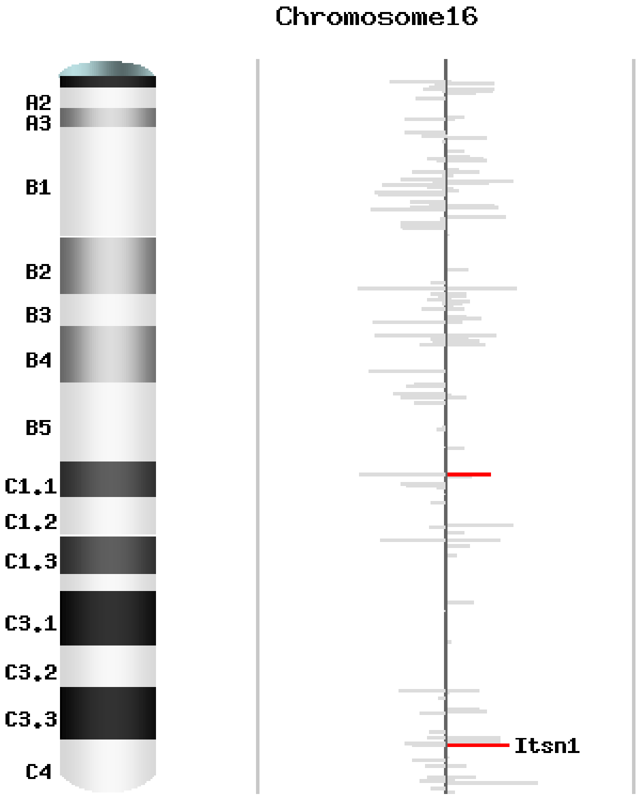
Chromosome16
A2
A3
B1
B2
B3
B4
B5
C1.1
C1.2
C1.3
C3.1
C3.2
C3.3
C4
Itsn1

Chromosome17

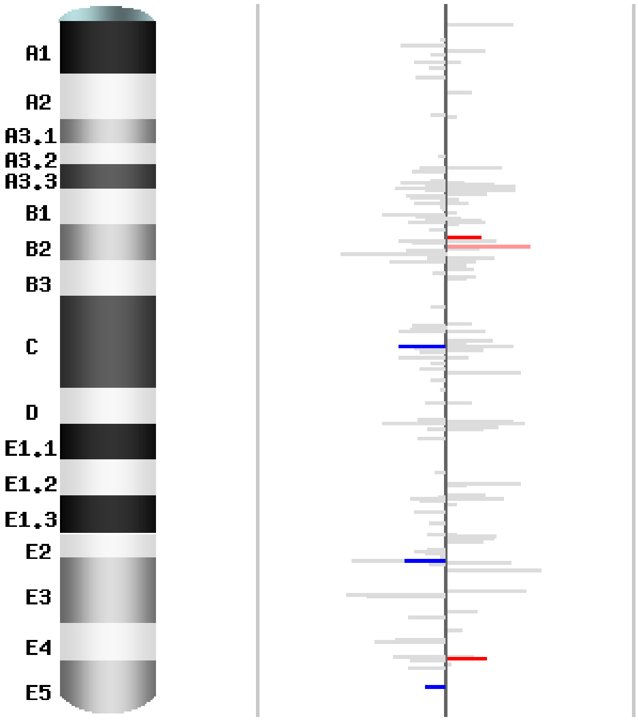

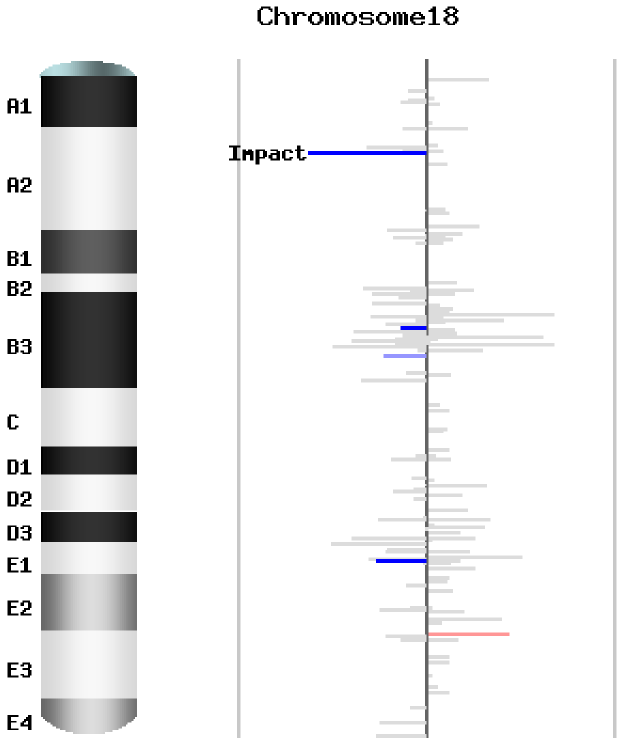
Chromosome18
A1
A2
B1
B2
B3
C
D1
D2
D3
E1
E2
E3
E4
Impact

Chromosome19

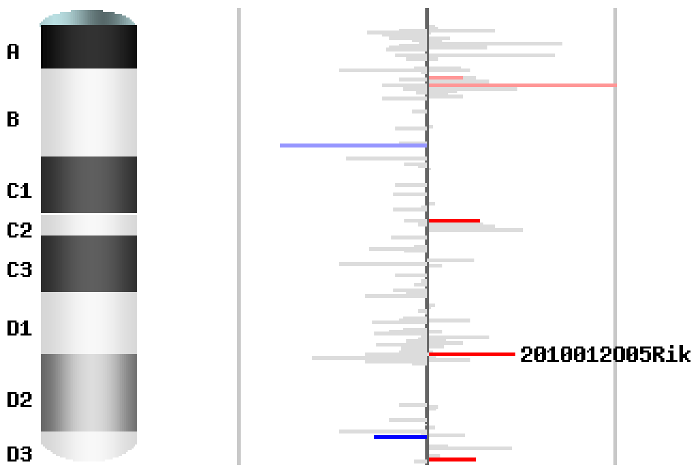

Figure S1.14. Genome-wide plot of imprinting status. The plots contain 5,076 unique Entrez genes covered by Solexa with counts no less than 4 in both reciprocal crosses. The heights of the bar is the difference of the AKR percentage in the two reciprocal crosses (p1-p2), representing the intensity of imprinting. The color stands for the direction of imprinting, blue for paternal and red for maternal. The intensity of the color represent the significance, grey for not significant (q-value ≥ 0.10), lighter blue and red for marginally significant ($0.05 \leq$ q-value < 0.10), darker blue and red for significant (q-value < 0.05). The gene name is shown if $|p1-p2| \geq 0.3$.

Figure S1.15. Distribution of number of Perlegen SNPs per RefSeq genes.

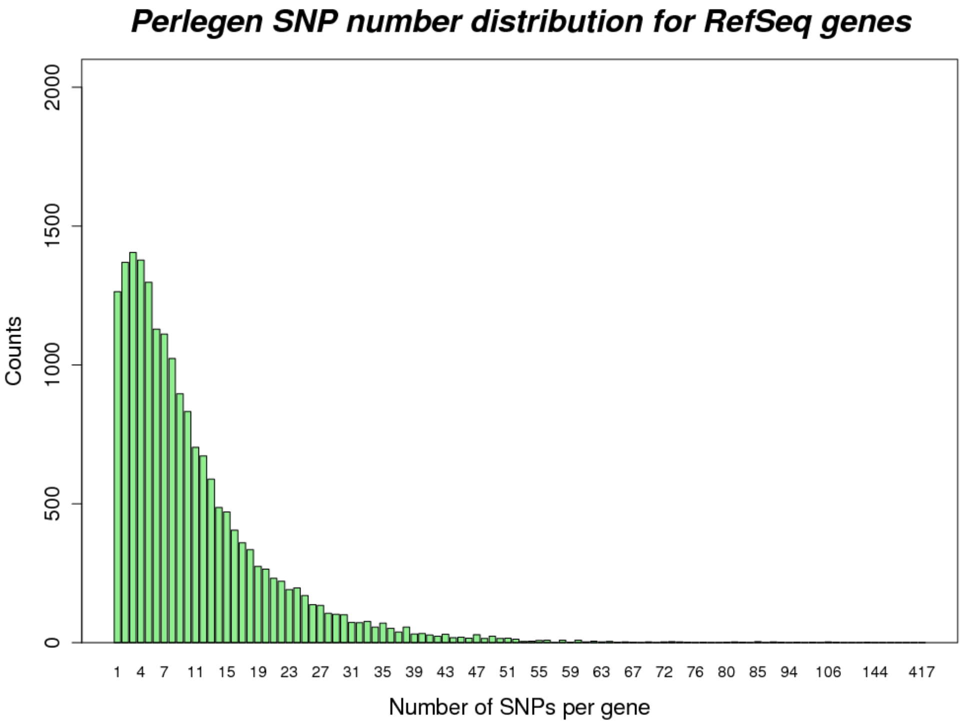

Figure S1.15. Distribution of number of Perlegen SNPs per RefSeq genes.

Figure S1.16. The quality score distribution of the Solexa SNPs.

A 1). Quality score of Solexa SNPs of lane 1, PWD x AKR, by SNP class.

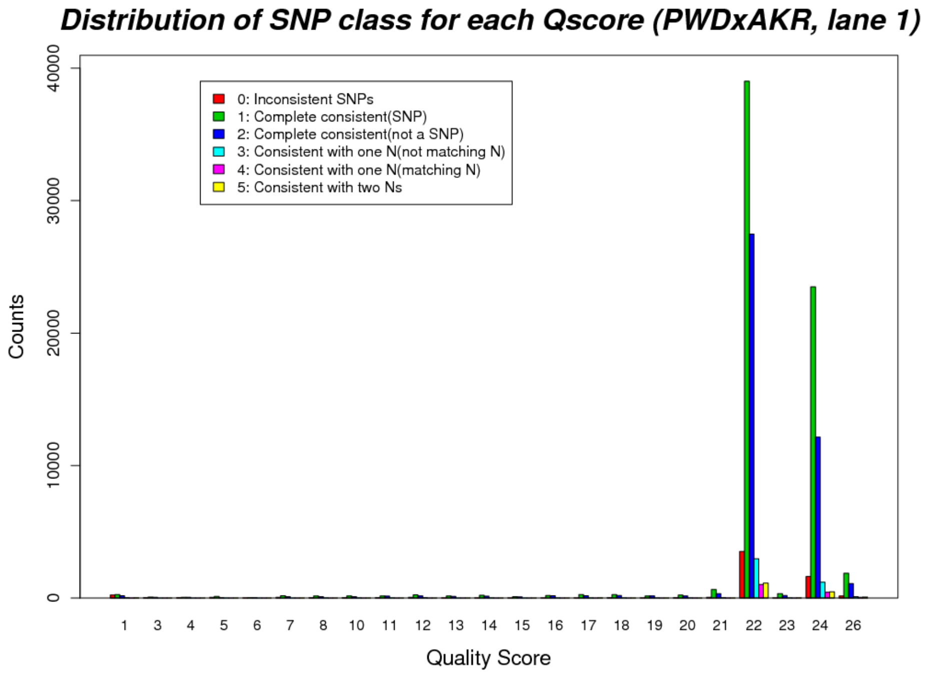

A 2). Quality score of Solexa SNPs of lane 2, PWD x AKR, by SNP class.

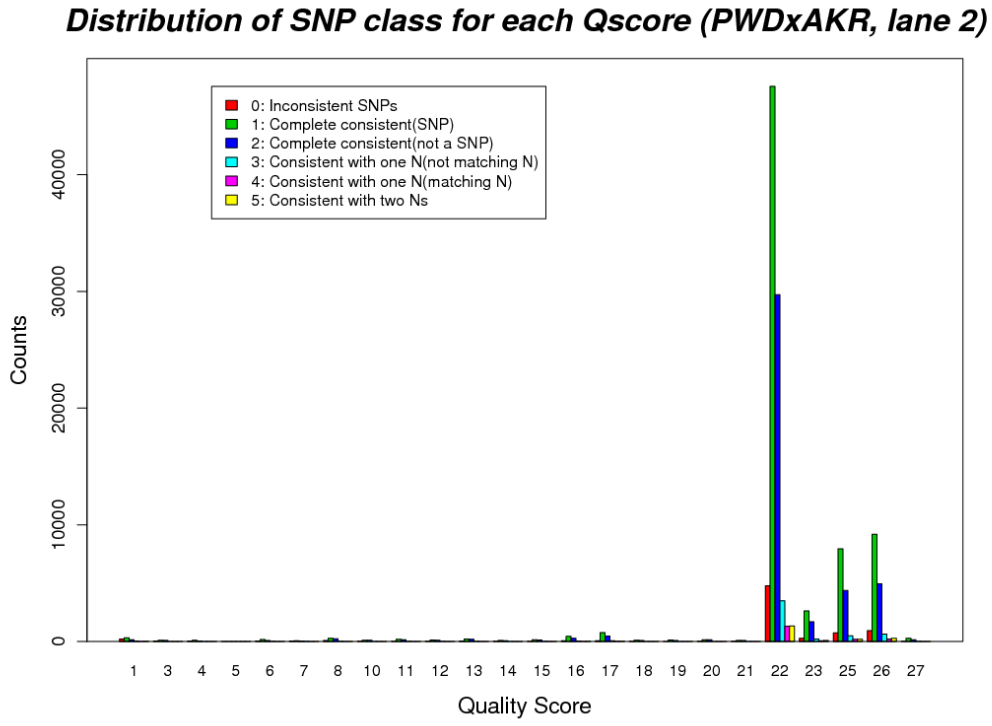

A 3). Quality score of Solexa SNPs of lane 3, PWD x AKR, by SNP class.

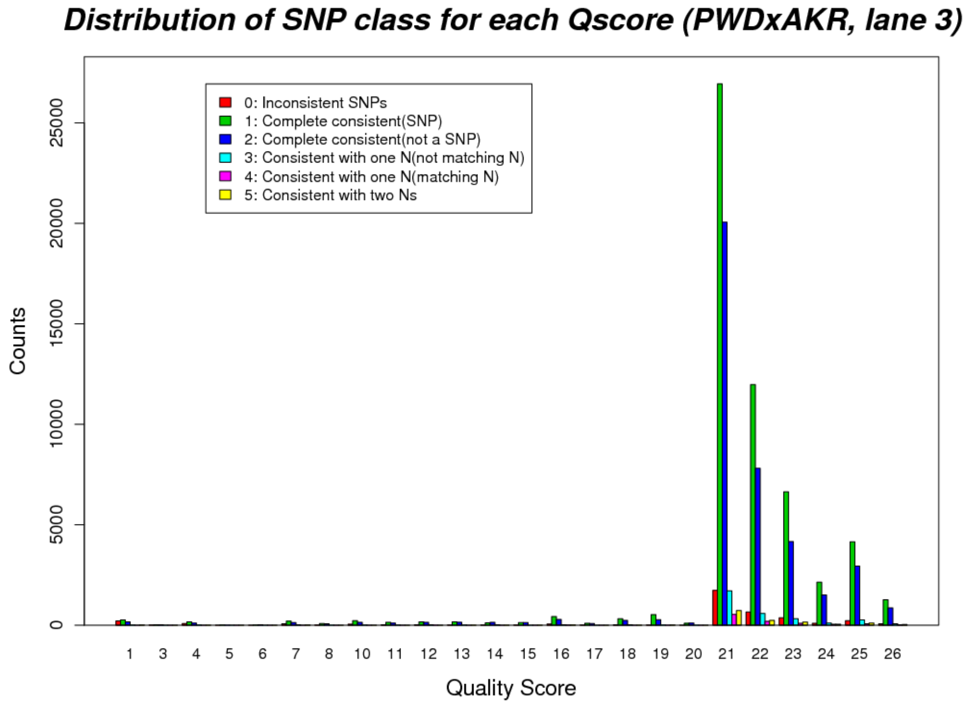

A 4). Quality score of Solexa SNPs of lane 4, PWD x AKR, by SNP class.

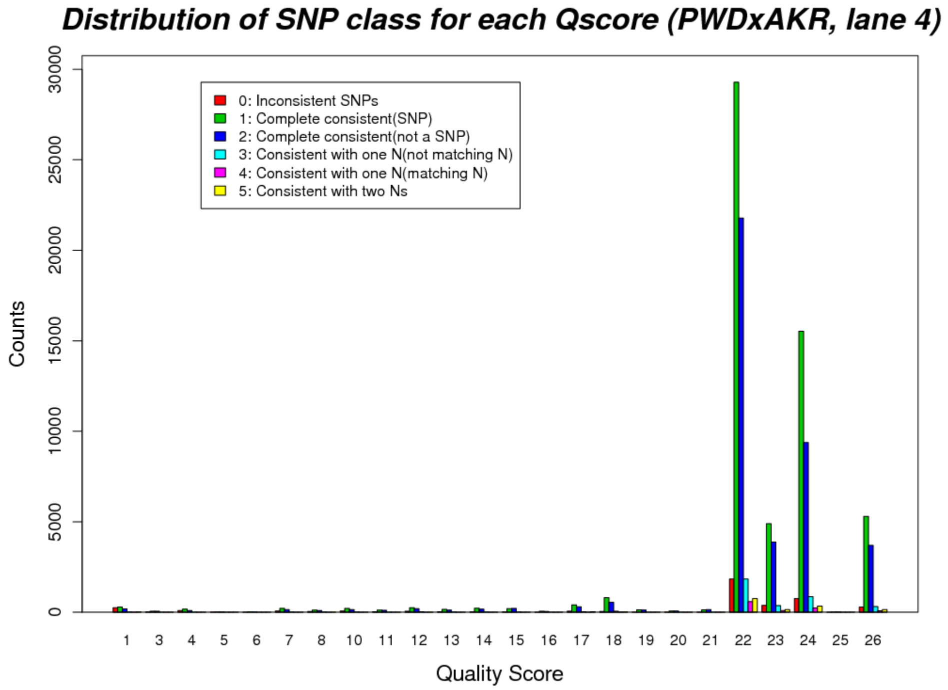

A 5). Quality score of Solexa SNPs of lane 6, PWD x AKR, by SNP class.

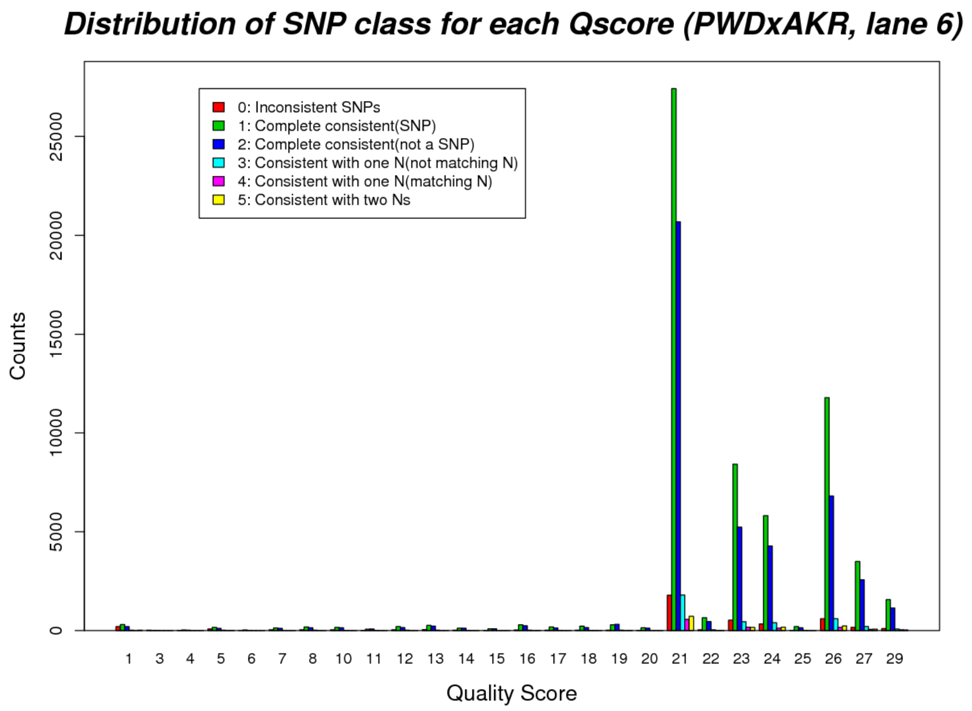

A 6). Quality score of Solexa SNPs of lane 7, PWD x AKR, by SNP class.

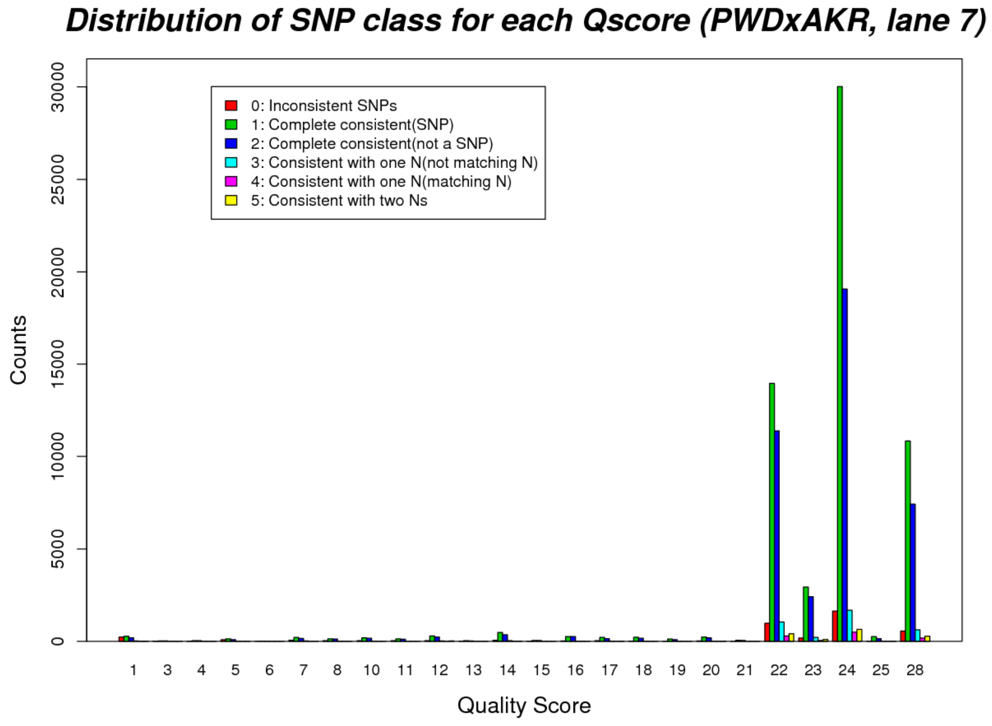

A 7). Quality score of Solexa SNPs of lane 8, PWD x AKR, by SNP class.

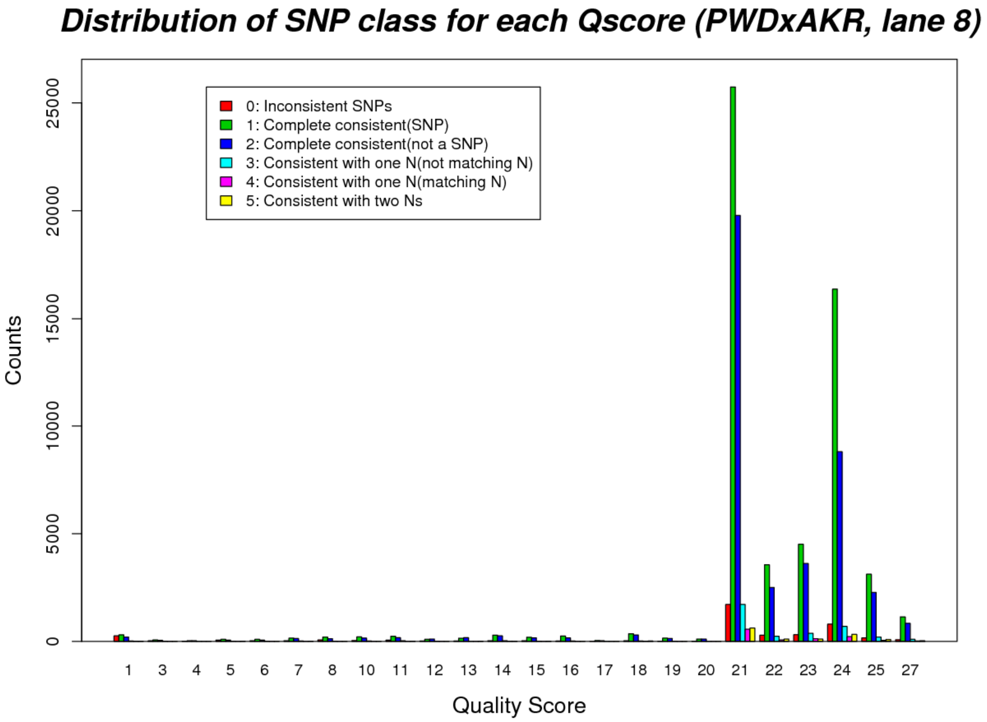

B 1). Quality score of Solexa SNPs of lane 1, AKR x PWD, by SNP class.

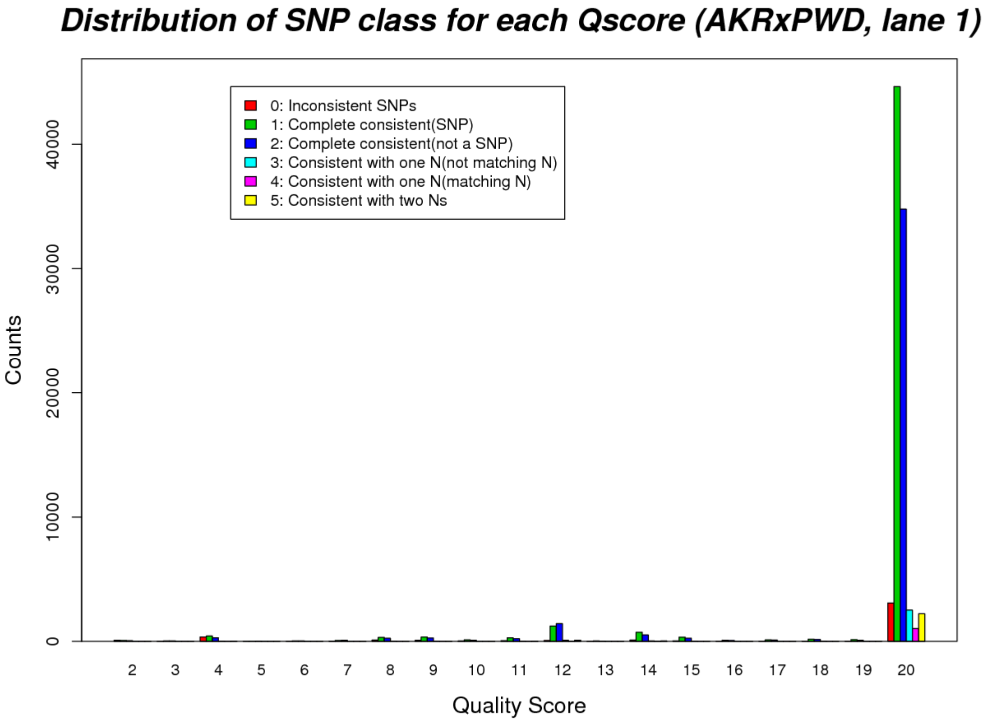

B 2). Quality score of Solexa SNPs of lane 2, AKR x PWD, by SNP class.

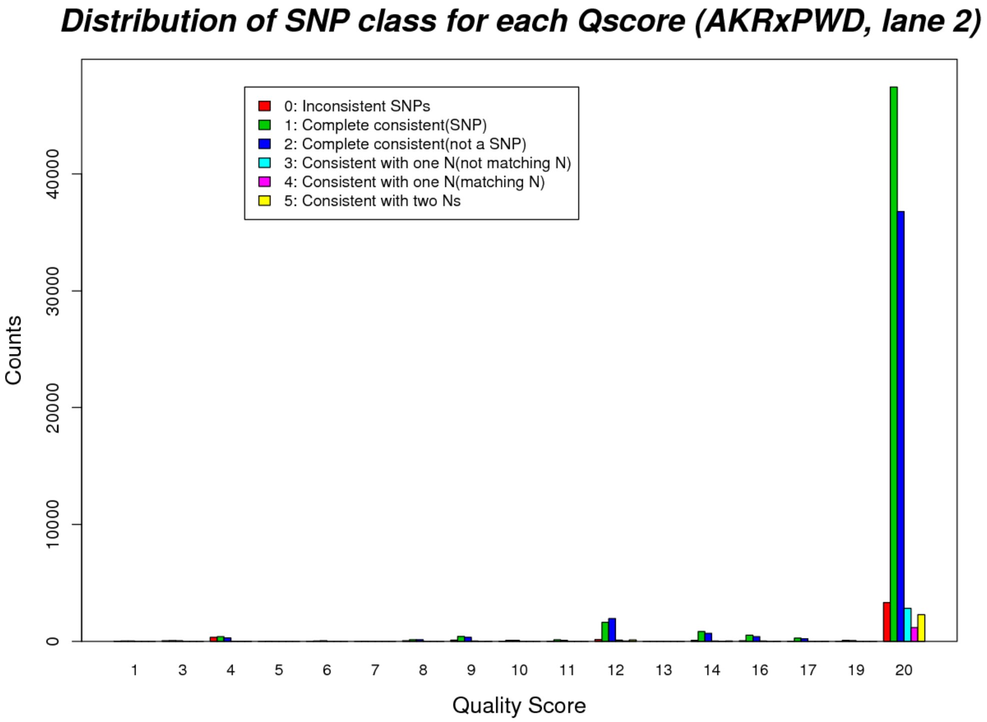

B 3). Quality score of Solexa SNPs of lane 3, AKR x PWD, by SNP class.

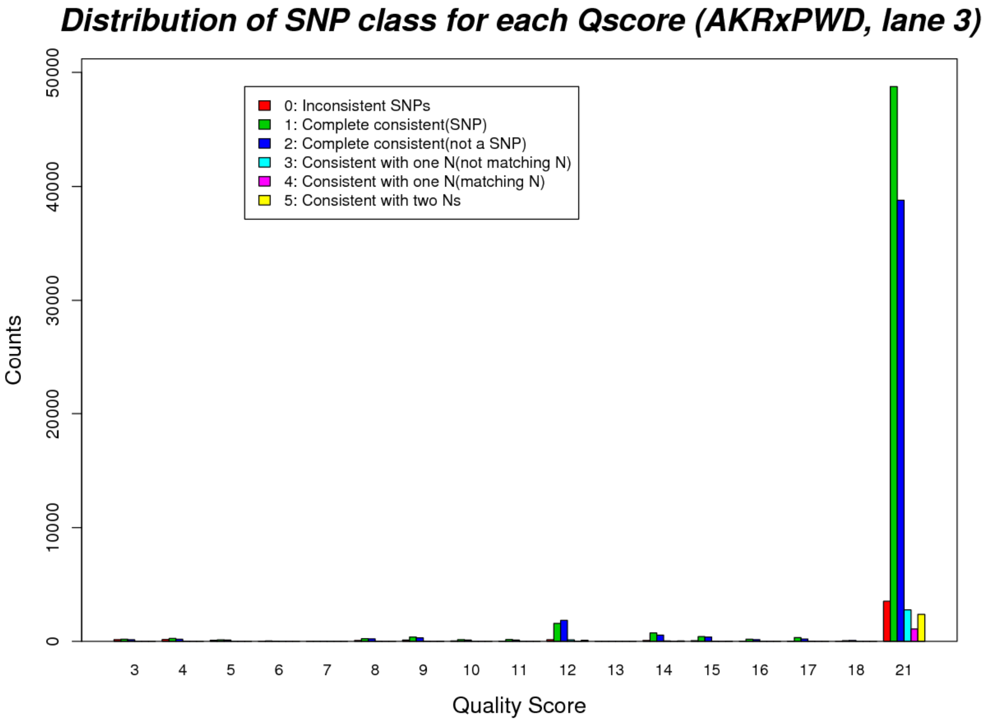

B 4). Quality score of Solexa SNPs of lane 4, AKR x PWD, by SNP class.

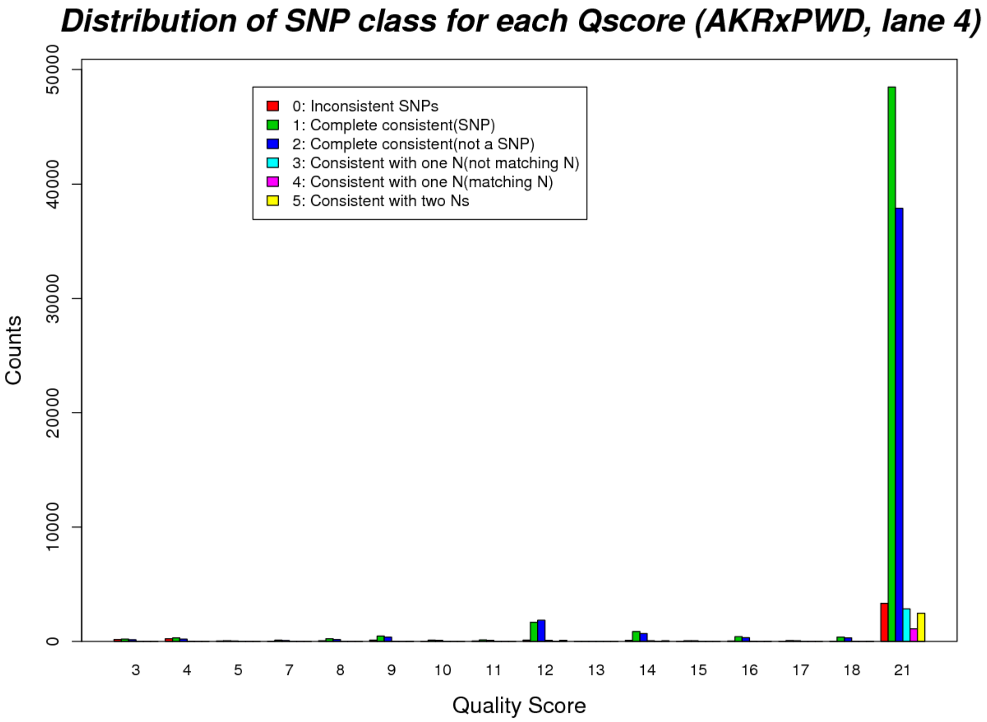

B 5). Quality score of Solexa SNPs of lane 5, AKR x PWD, by SNP class.

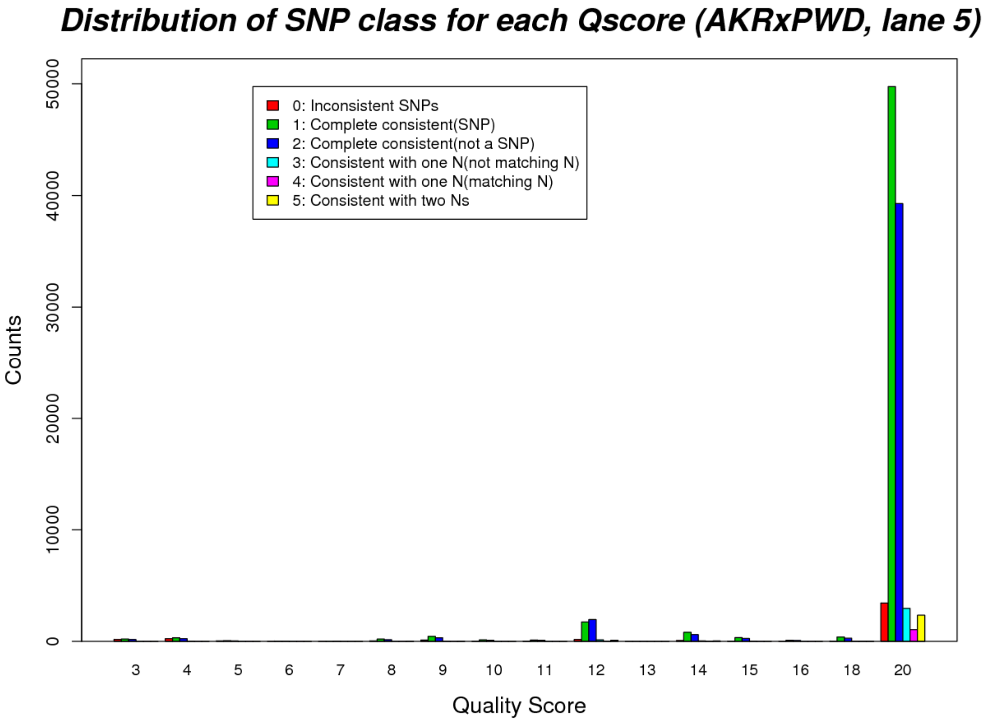

B 6). Quality score of Solexa SNPs of lane 6, AKR x PWD, by SNP class.

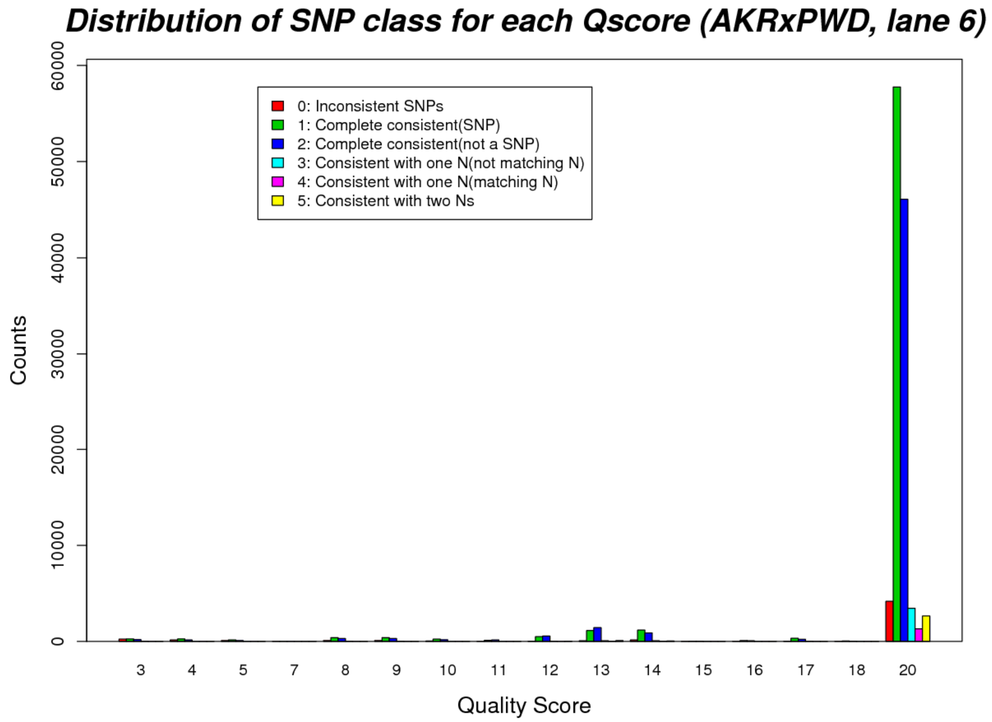

B 7). Quality score of Solexa SNPs of lane 7, AKR x PWD, by SNP class.

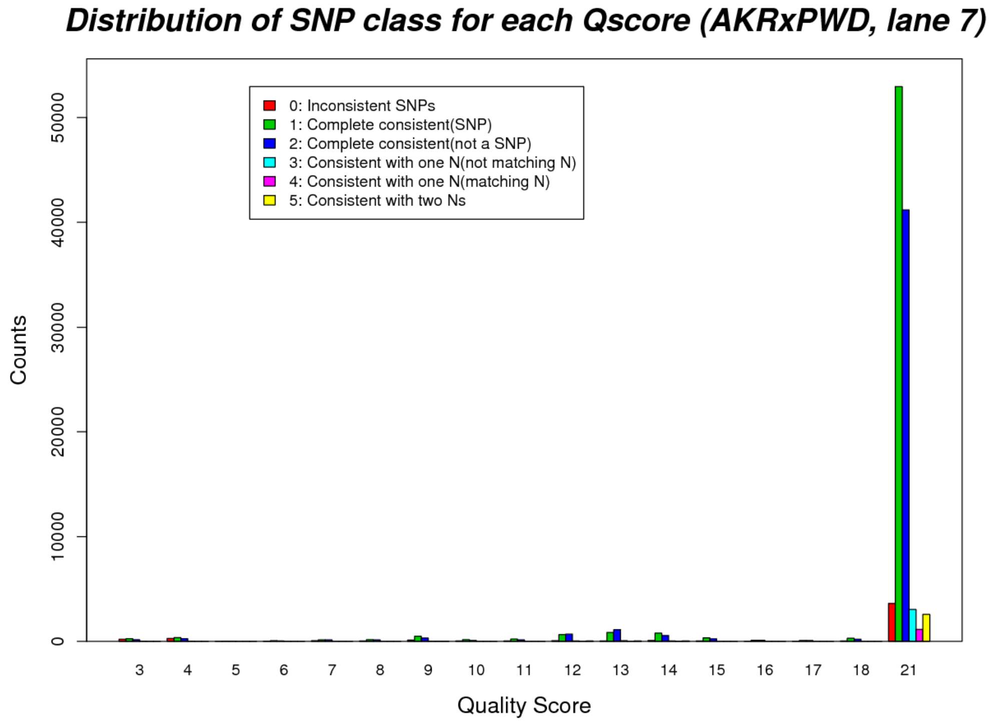

B 8). Quality score of Solexa SNPs of lane 8, AKR x PWD, by SNP class.

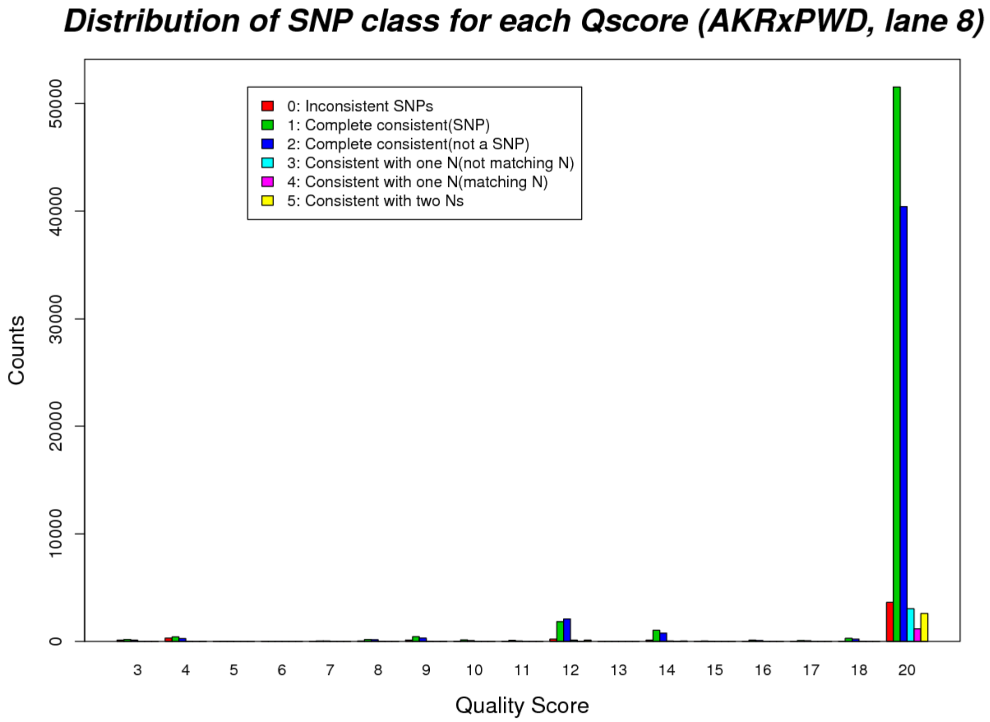

Figure S1.16. The quality score distribution of the Solexa SNPs. A, Quality score distribution for the seven lanes in the Solexa run of the PWD x AKR sample. **B,** Quality score distribution for the eight lanes in the Solexa run of the AKR x PWD sample.

Figure S1.17. The distribution of number of mismatches in the SNP-containing Solexa reads.

A 1).Mismatch score distribution of Solexa SNPs of lane 1, PWD x AKR, by SNP class.

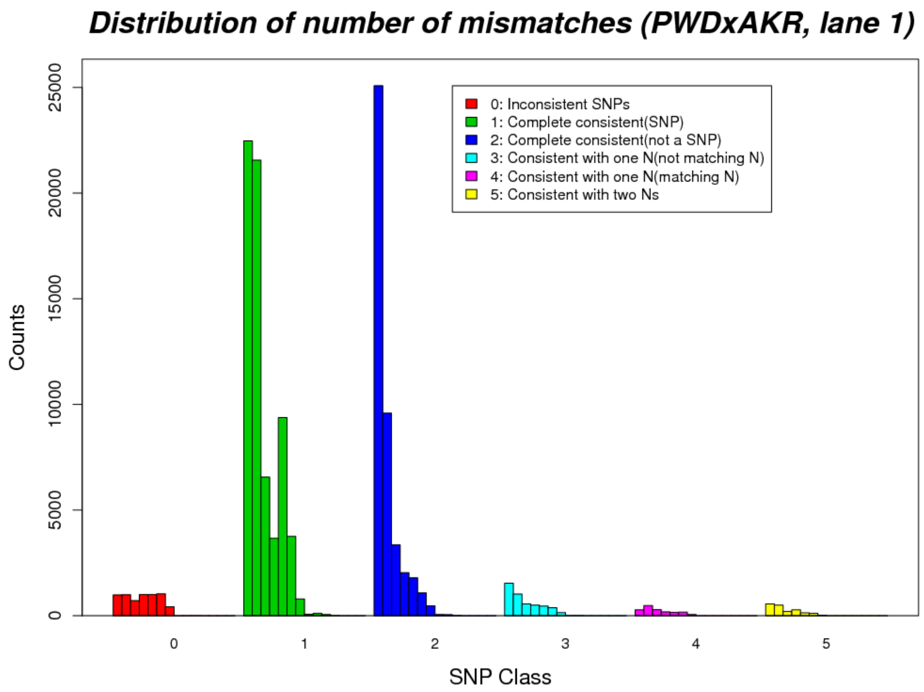

A 2).Mismatch score distribution of Solexa SNPs of lane 2, PWD x AKR, by SNP class.

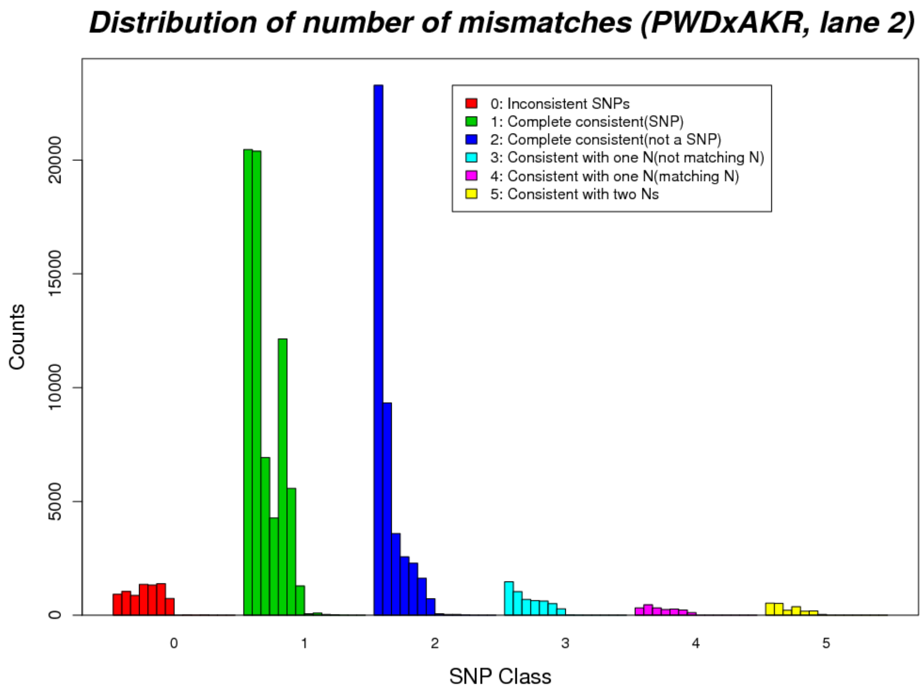

A 3).Mismatch score distribution of Solexa SNPs of lane 3, PWD x AKR, by SNP class.

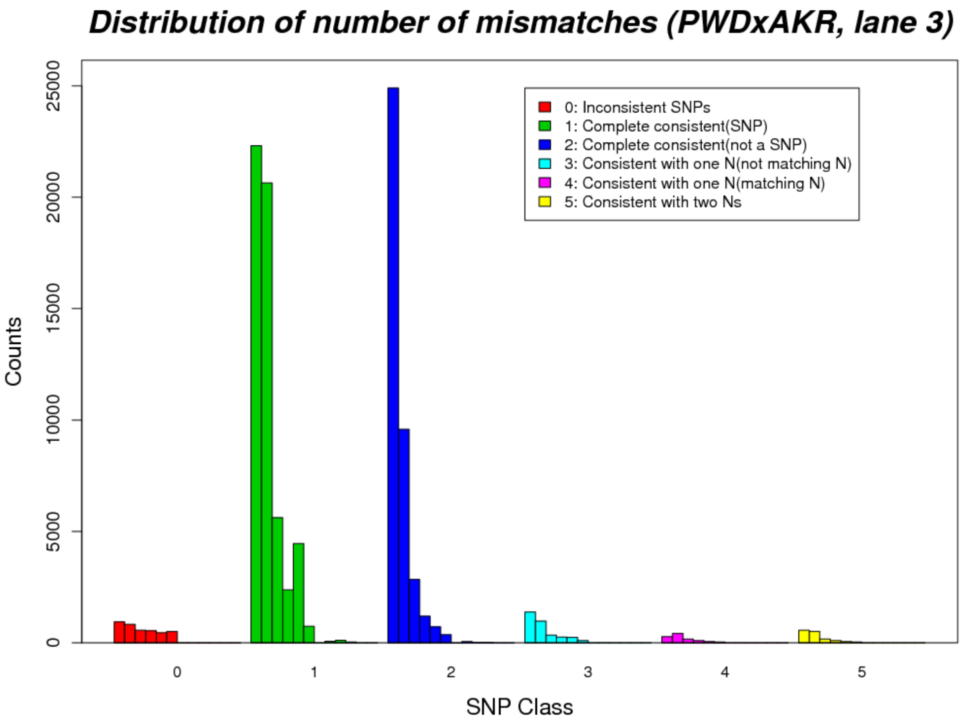

A 4).Mismatch score distribution of Solexa SNPs of lane 4, PWD x AKR, by SNP class.

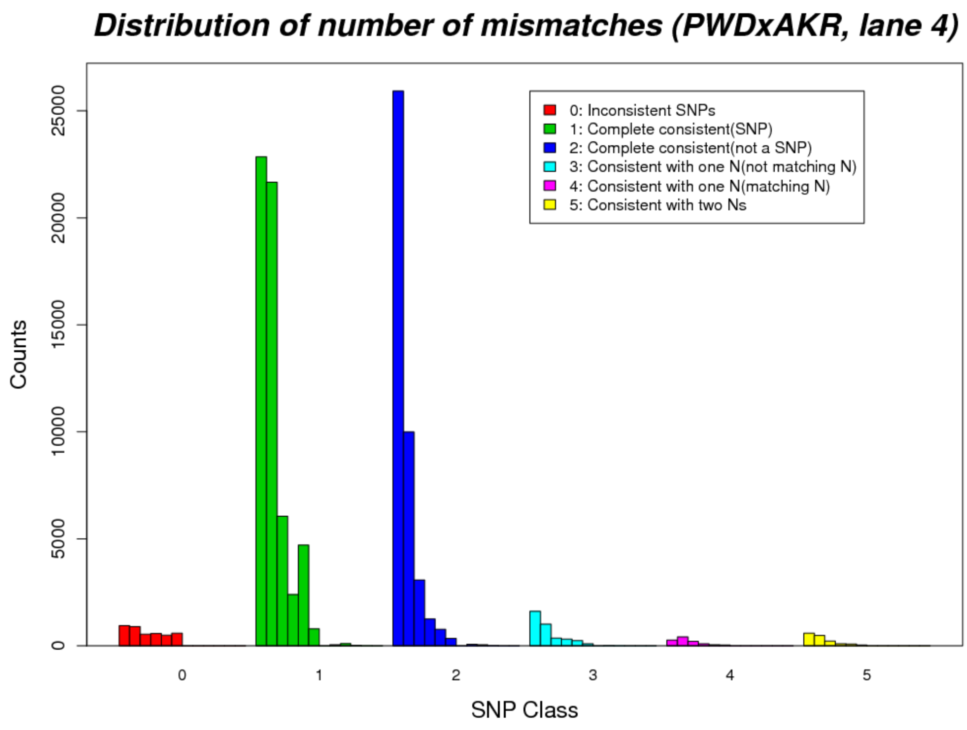

A 5).Mismatch score distribution of Solexa SNPs of lane 6, PWD x AKR, by SNP class.

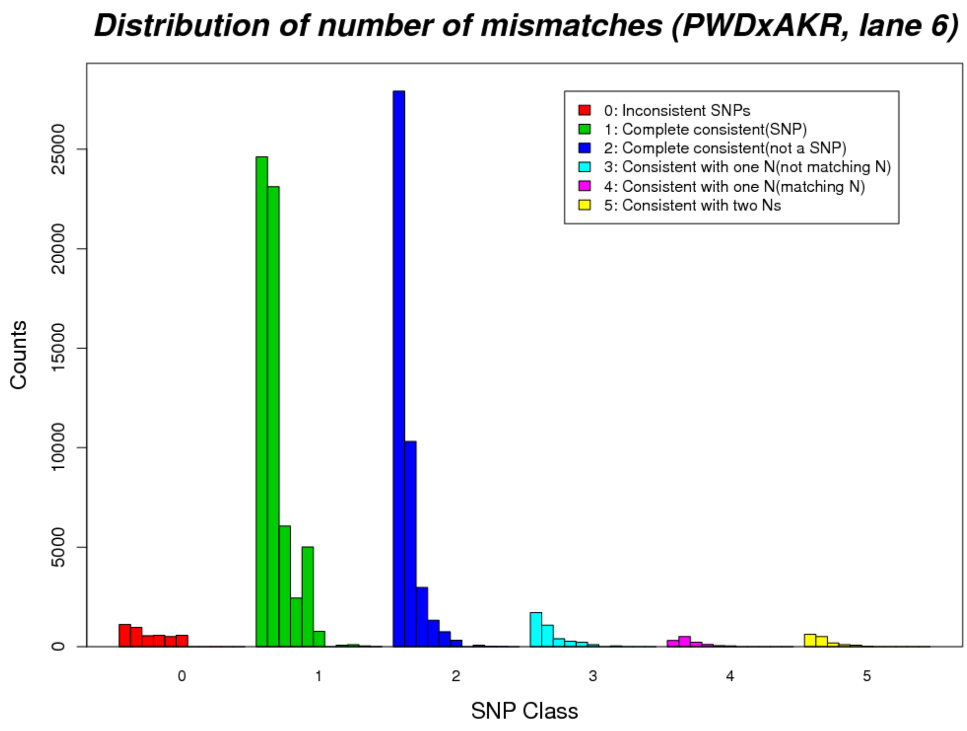

A 6).Mismatch score distribution of Solexa SNPs of lane 7, PWD x AKR, by SNP class.

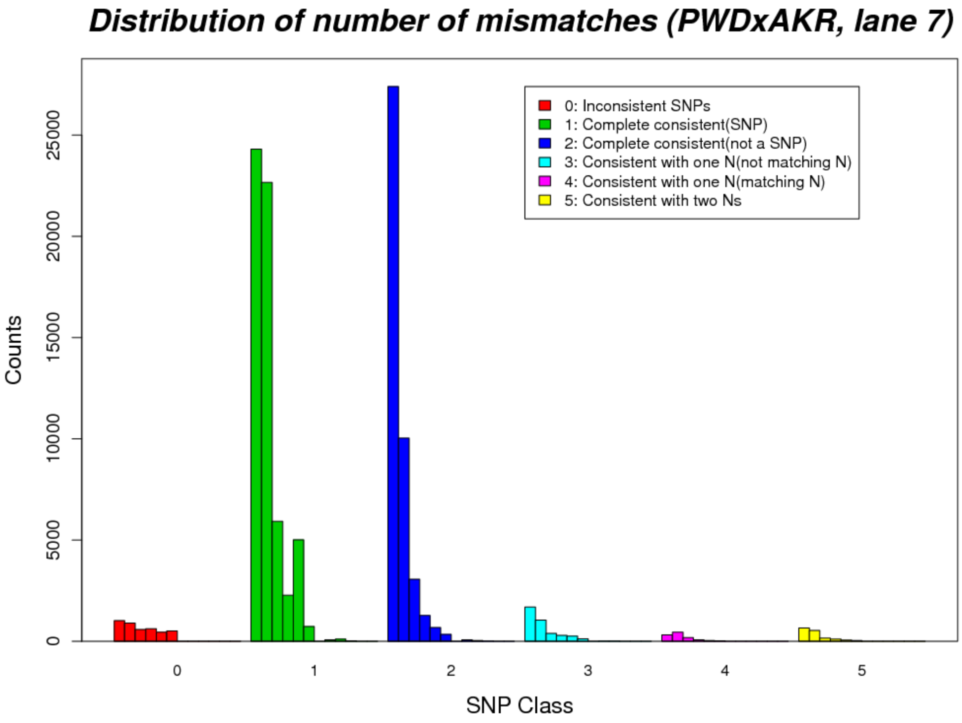

A 7).Mismatch score distribution of Solexa SNPs of lane 8, PWD x AKR, by SNP class.

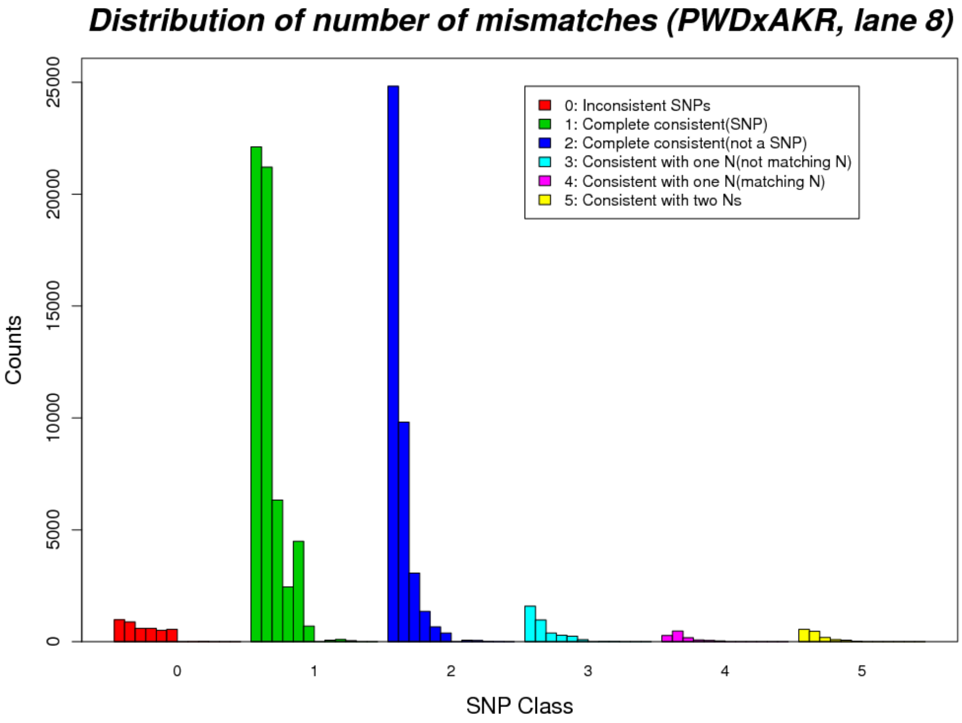

B1).Mismatch score distribution of Solexa SNPs of lane 1, AKR x PWD, by SNP class.

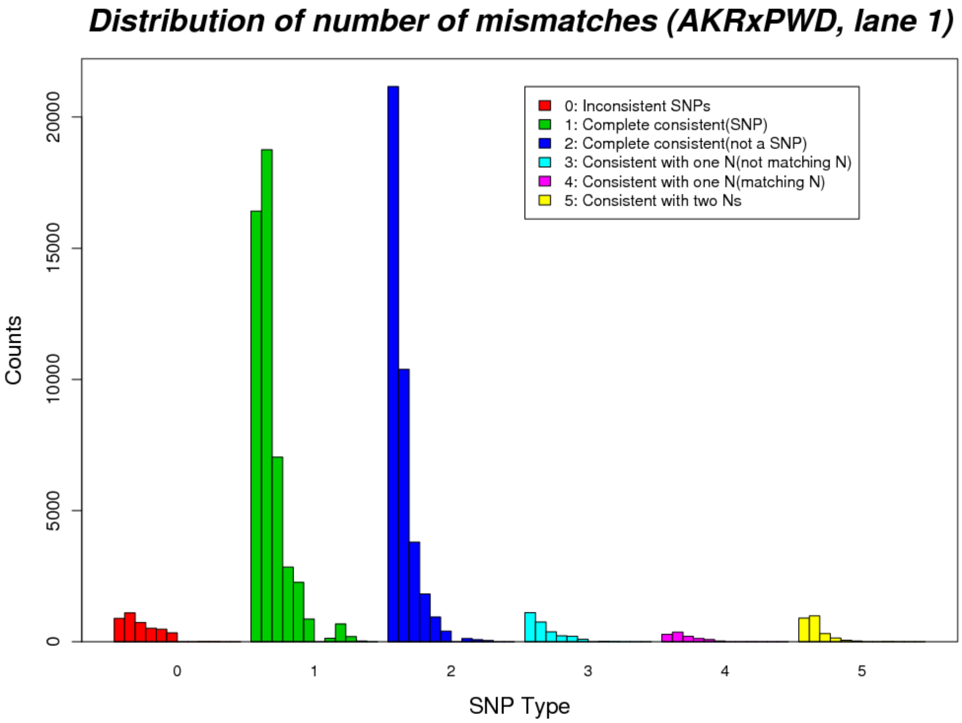

B 2).Mismatch score distribution of Solexa SNPs of lane 2, AKR x PWD, by SNP class.

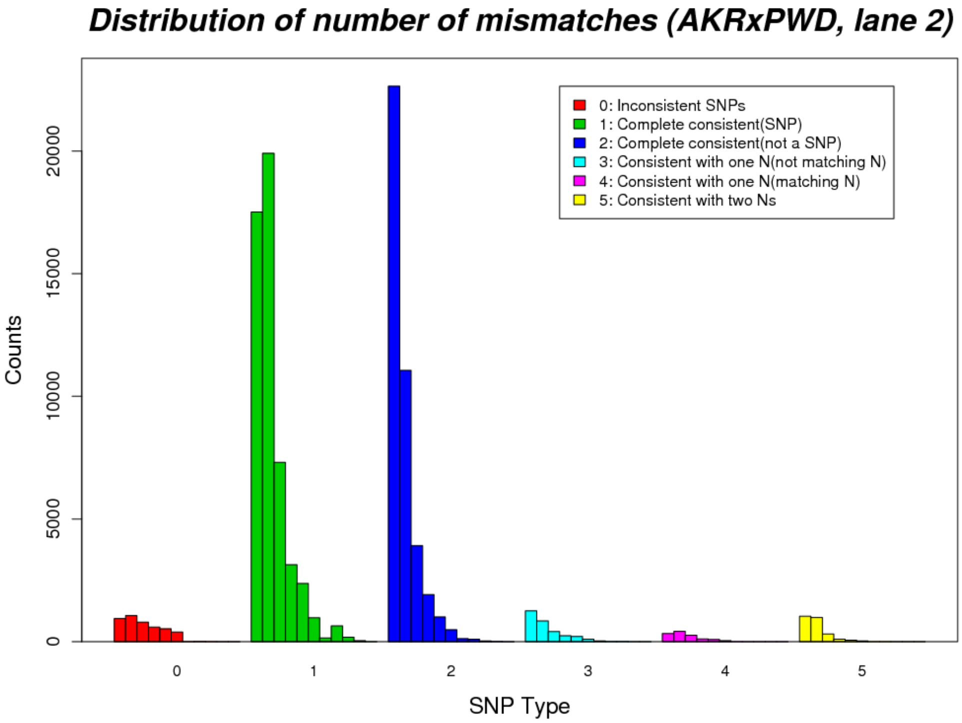

B 3).Mismatch score distribution of Solexa SNPs of lane 3, AKR x PWD, by SNP class.

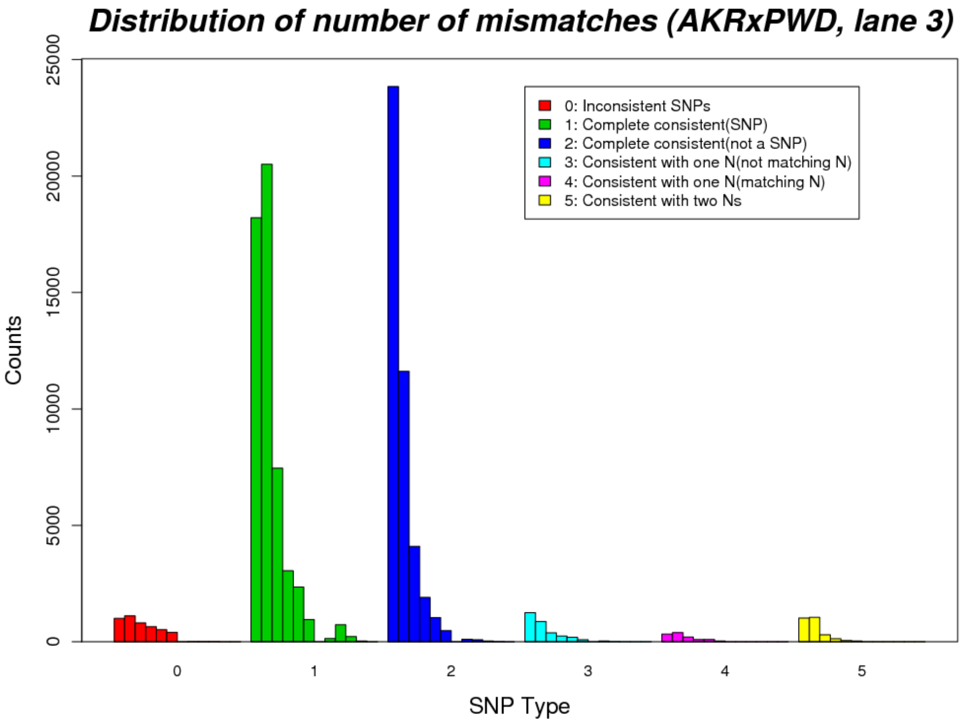

B 4).Mismatch score distribution of Solexa SNPs of lane 4, AKR x PWD, by SNP class.

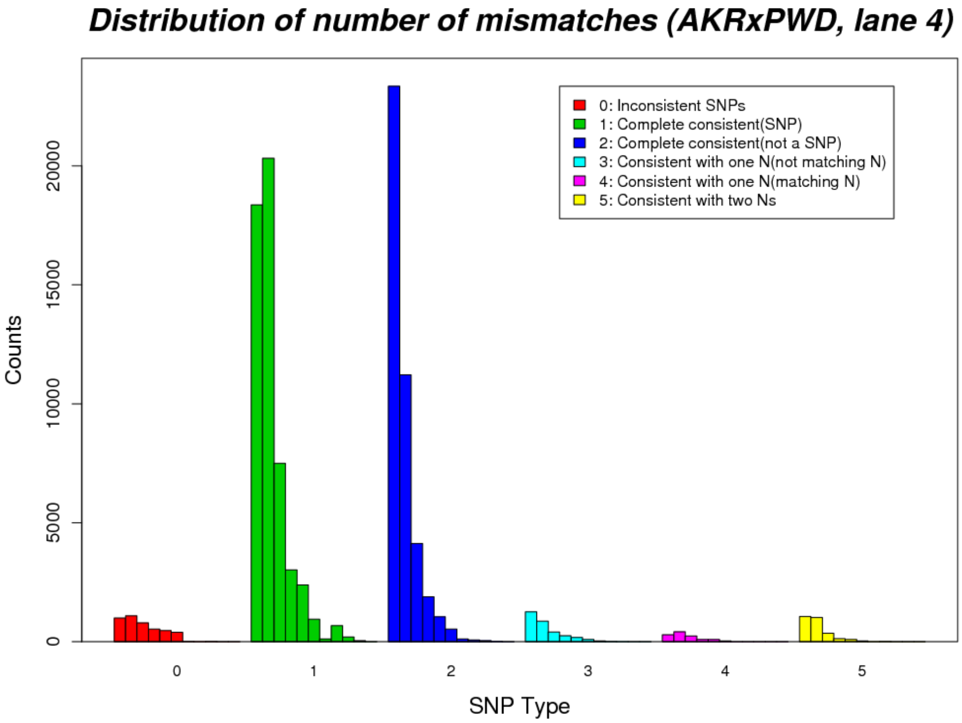

B 5).Mismatch score distribution of Solexa SNPs of lane 5, AKR x PWD, by SNP class.

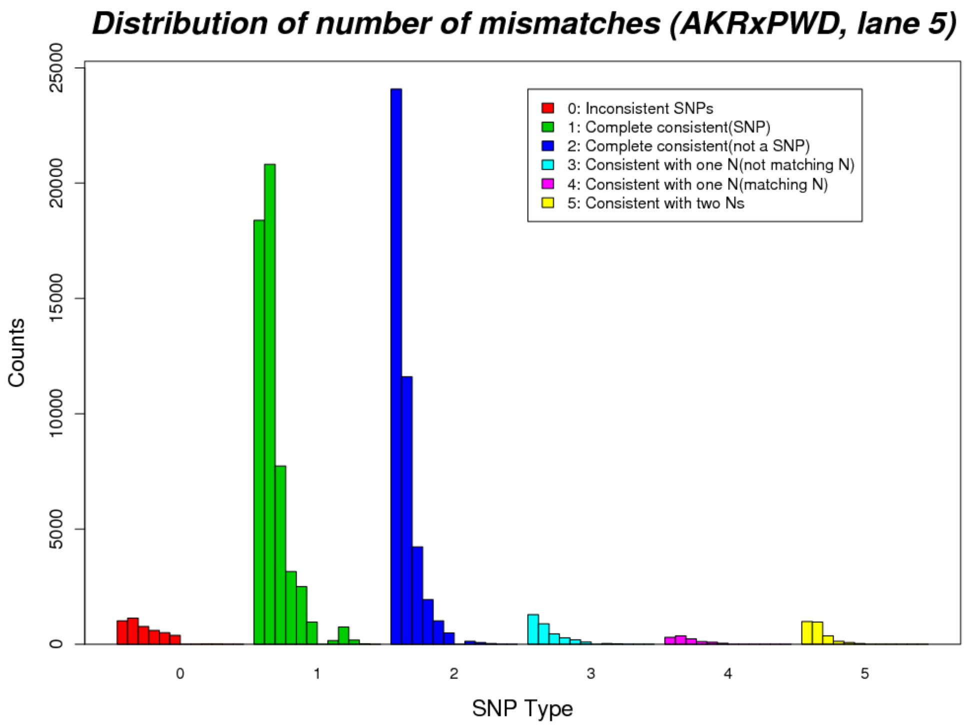

B 6).Mismatch score distribution of Solexa SNPs of lane 6, AKR x PWD, by SNP class.

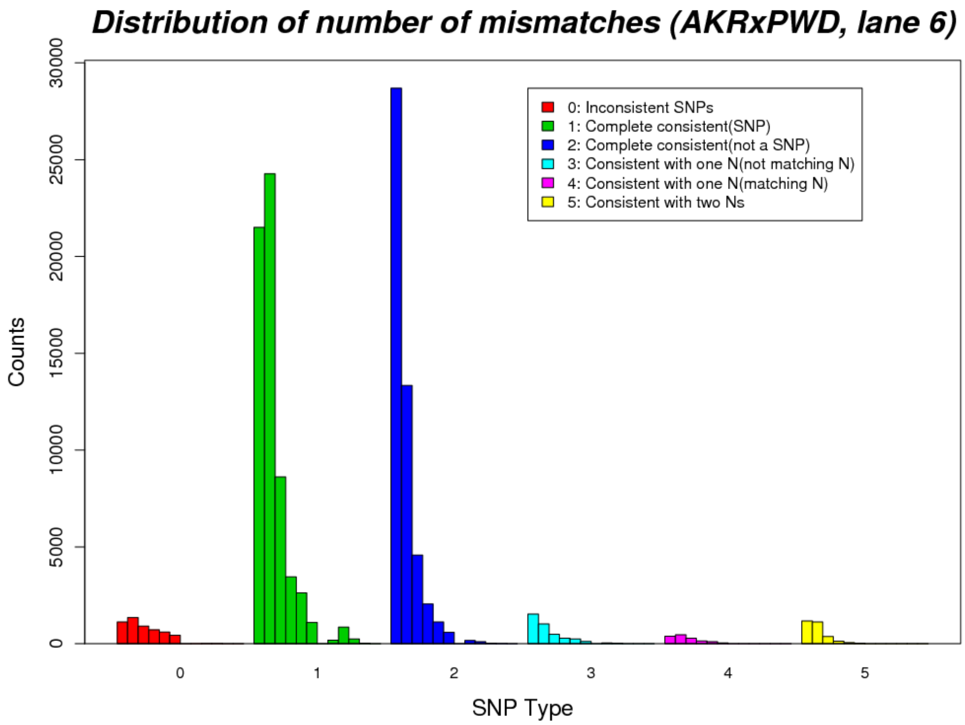

B 7).Mismatch score distribution of Solexa SNPs of lane 7, AKR x PWD, by SNP class.

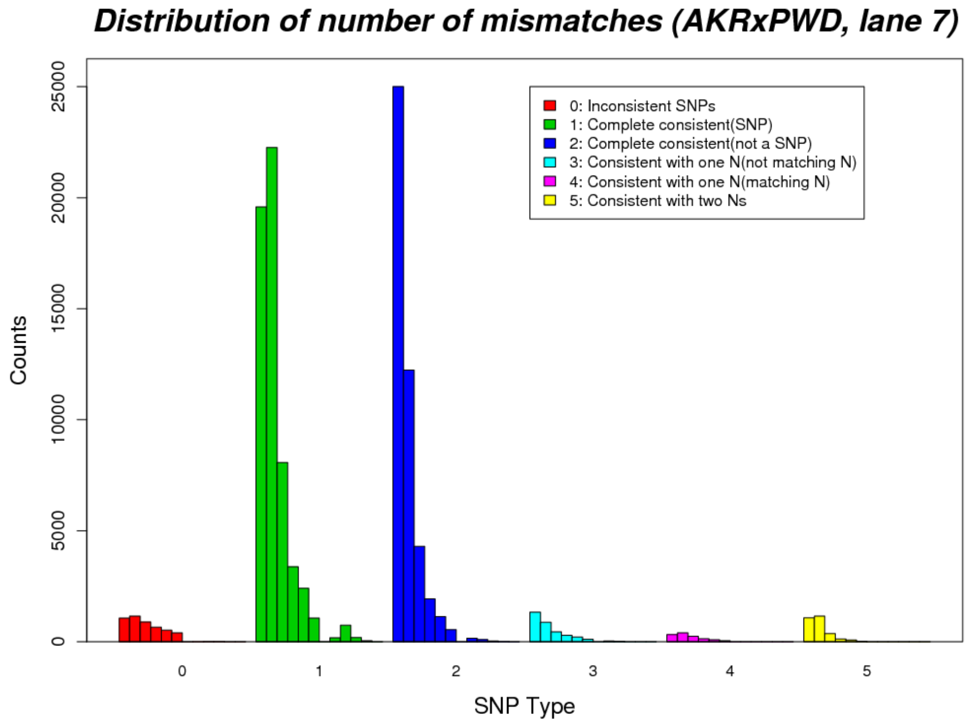

B 8).Mismatch score distribution of Solexa SNPs of lane 8, AKR x PWD, by SNP class.

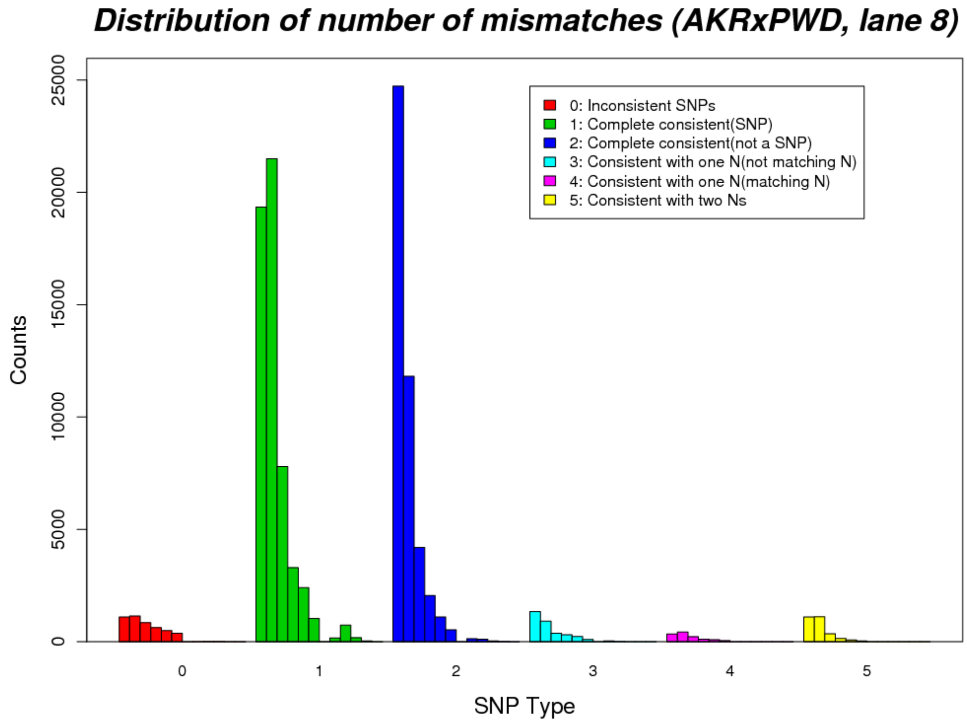

Figure S1.17. The distribution of number of mismatches in the SNP-containing Solexa reads. A, for the seven lanes in the Solexa run of the PWD x AKR sample. **B,** for the eight lanes in the Solexa run of the AKR x PWD sample. Figure legend: the colors stand for the SNP classes; the heights of the bars (from left to right) are the counts of SNP-containing reads with 0, 1, 2, … mismatches. For the Class 1 SNP (super clean SNP used in following analysis), the two bars of 0 and 1 mismatch are extremely high with about 20,000 counts, representing the perfectly aligned reads the reference and alternative SNP alleles. This pattern is not seen in any other SNP classes. In the inconsistent SNP class (class 0), the number of mismatches are uniformly distributed.

Figure S1.18. The quality score distribution of the Solexa SNPs.

A 1). Match length of Solexa SNPs of lane 1, PWD x AKR, by SNP class.

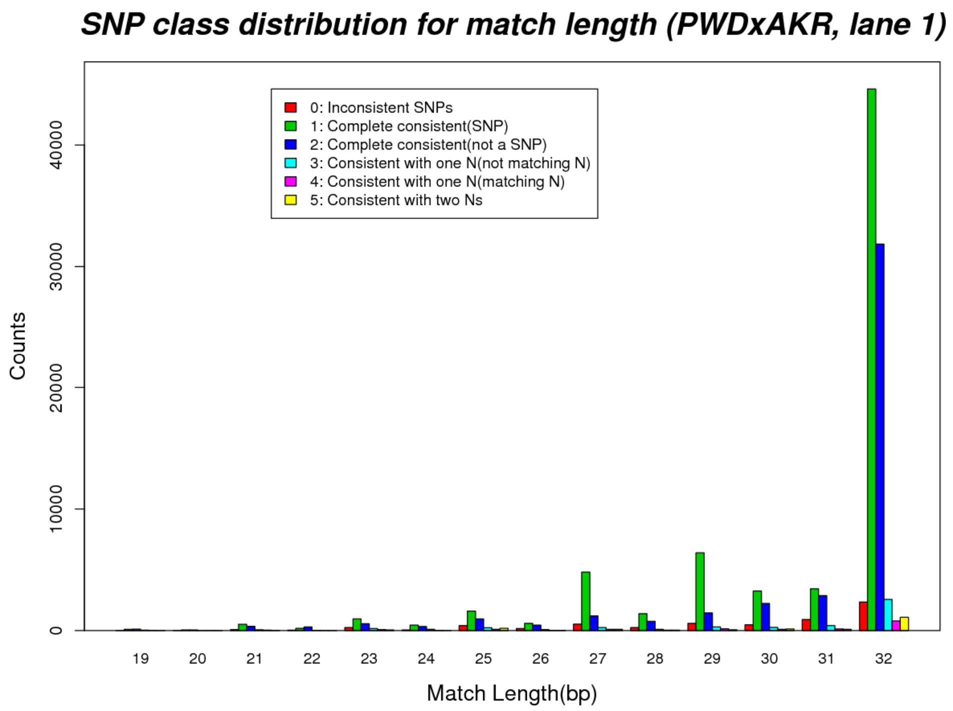

A 2). Match length of Solexa SNPs of lane 2, PWD x AKR, by SNP class.

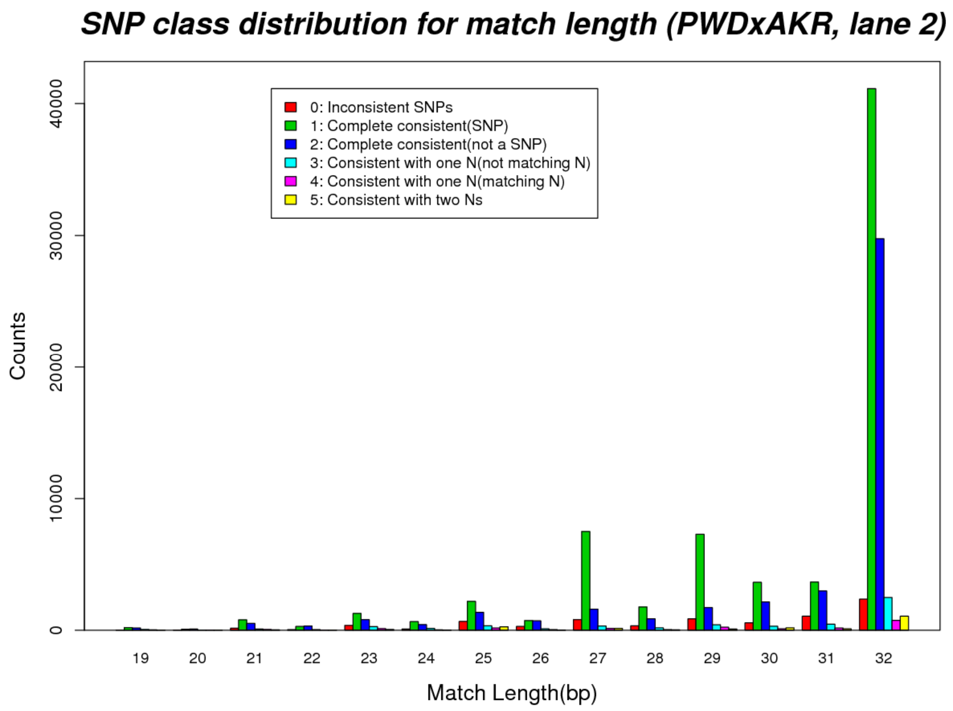

A 3). Match length of Solexa SNPs of lane 3, PWD x AKR, by SNP class.

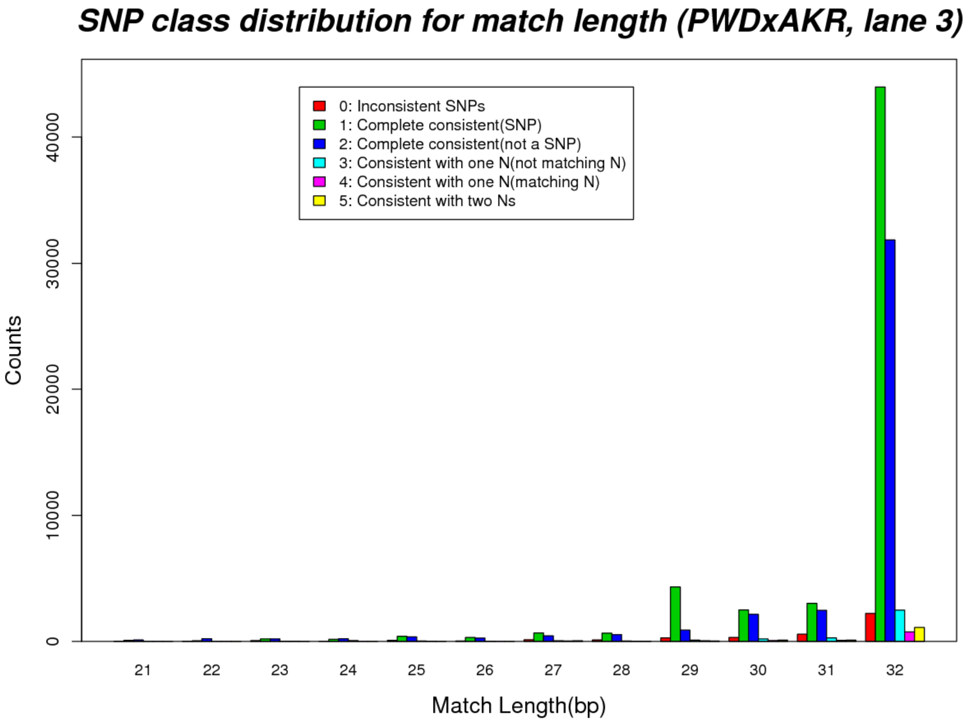

A 4). Match length of Solexa SNPs of lane 4, PWD x AKR, by SNP class.

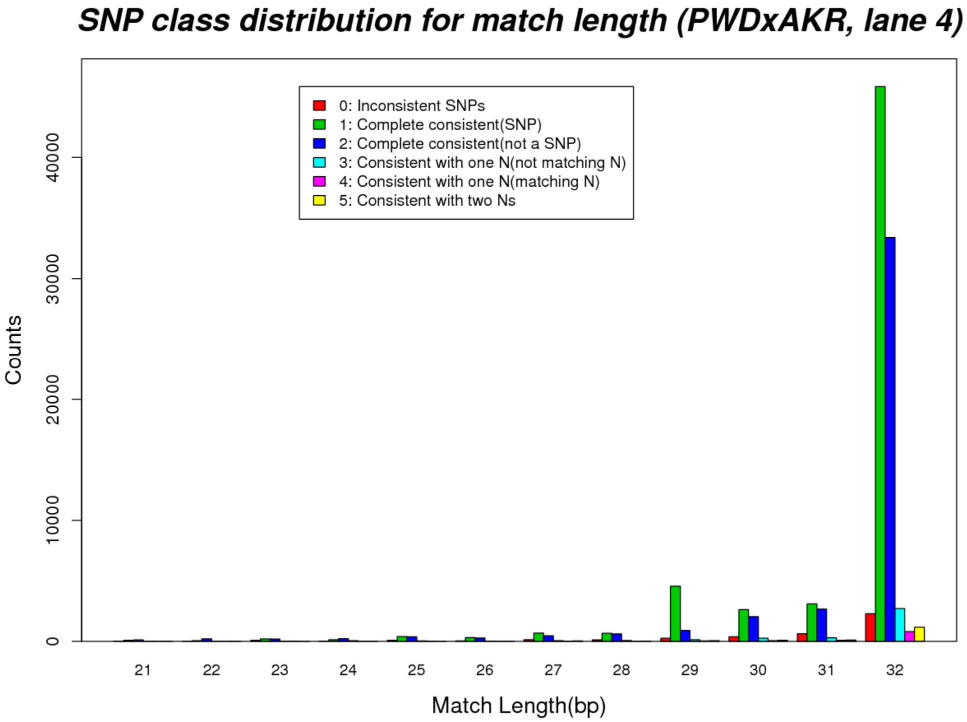

A 5). Match length of Solexa SNPs of lane 6, PWD x AKR, by SNP class.

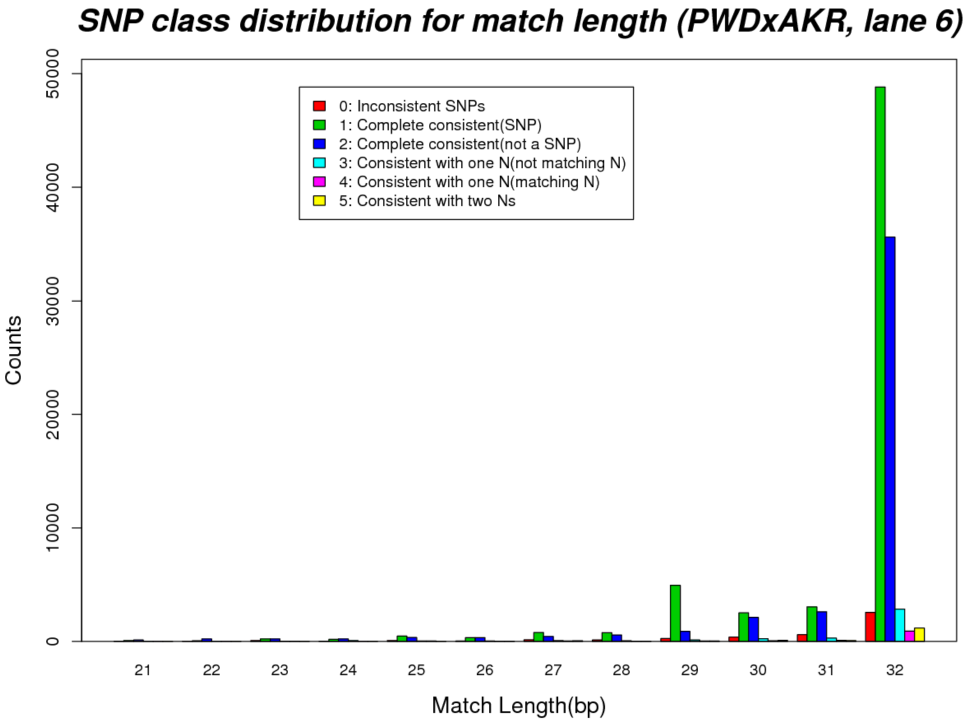

A 6). Match length of Solexa SNPs of lane 7, PWD x AKR, by SNP class.

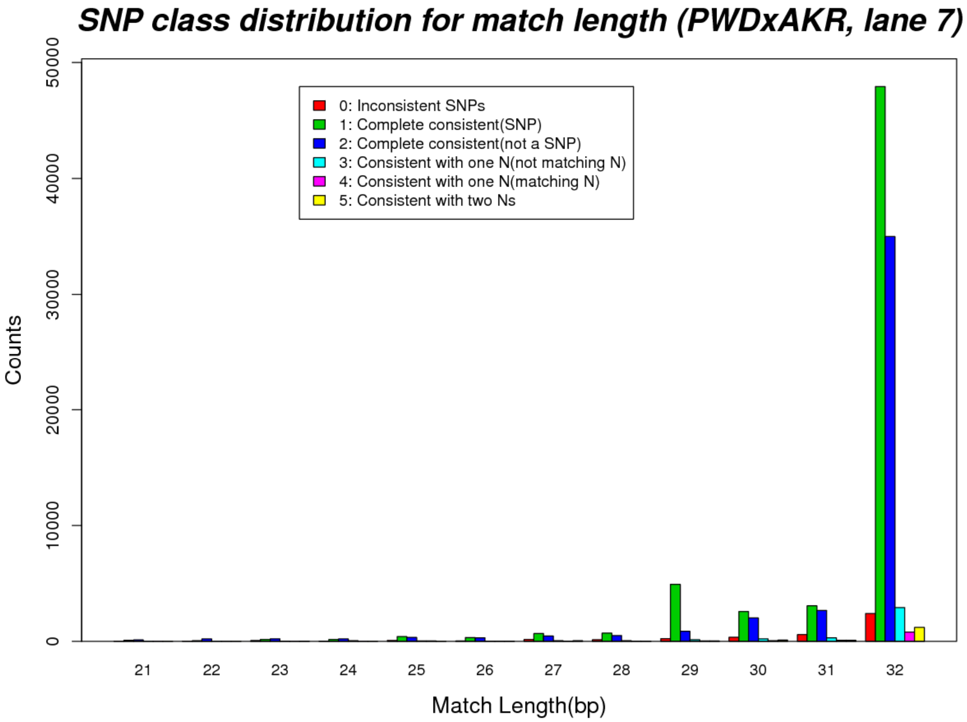

A 7). Match length of Solexa SNPs of lane 8, PWD x AKR, by SNP class.

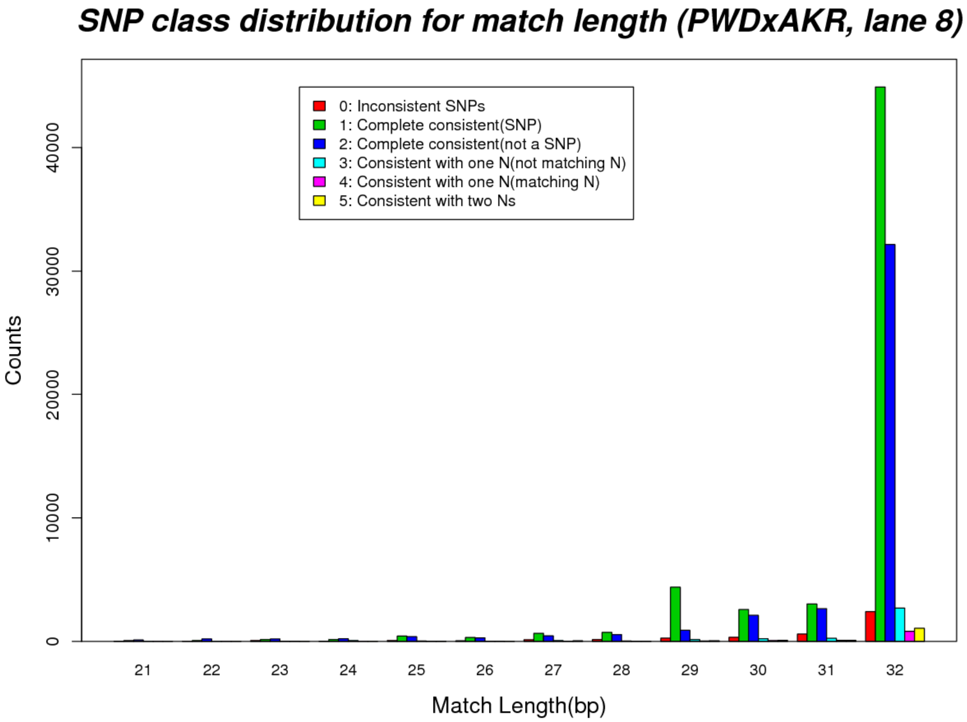

B 1). Match length of Solexa SNPs of lane 1, AKR x PWD, by SNP class.

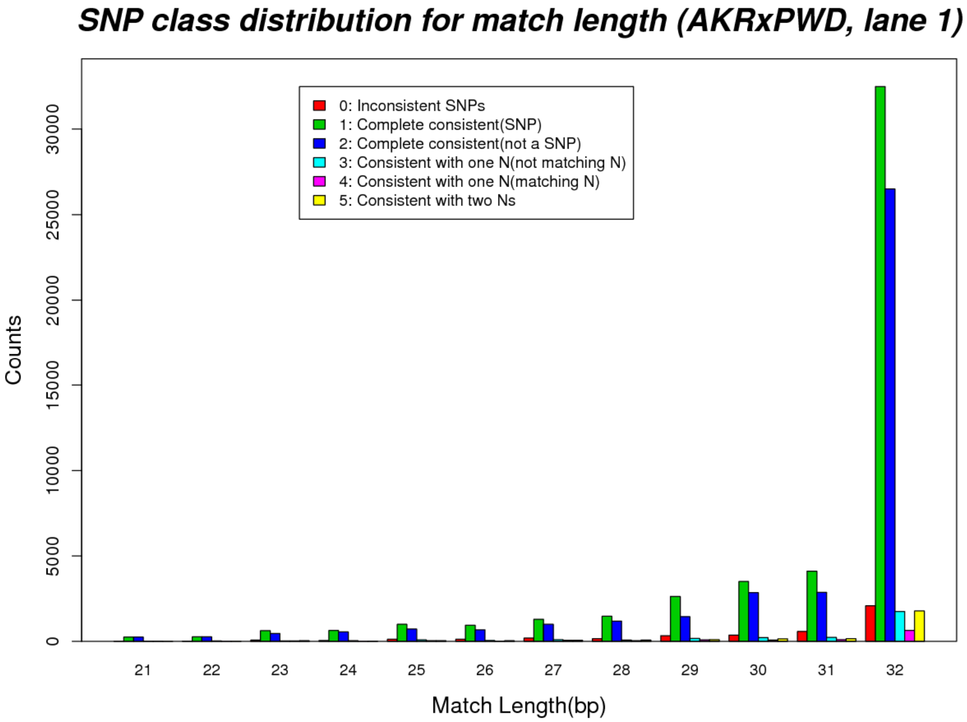

B 2). Match length of Solexa SNPs of lane 2, AKR x PWD, by SNP class.

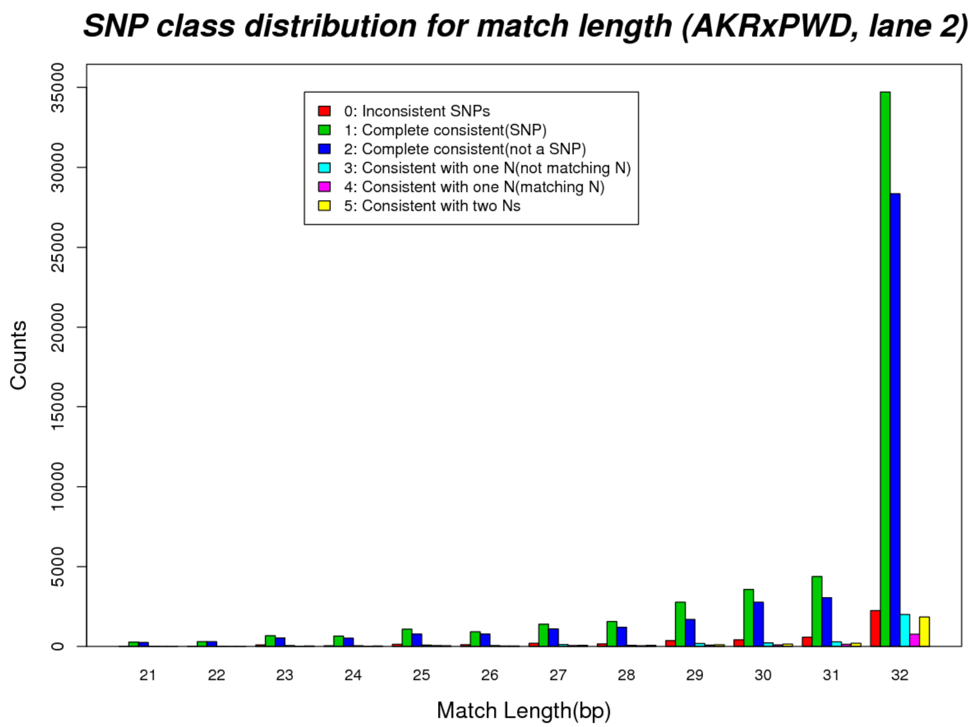

B 3). Match length of Solexa SNPs of lane 3, AKR x PWD, by SNP class.

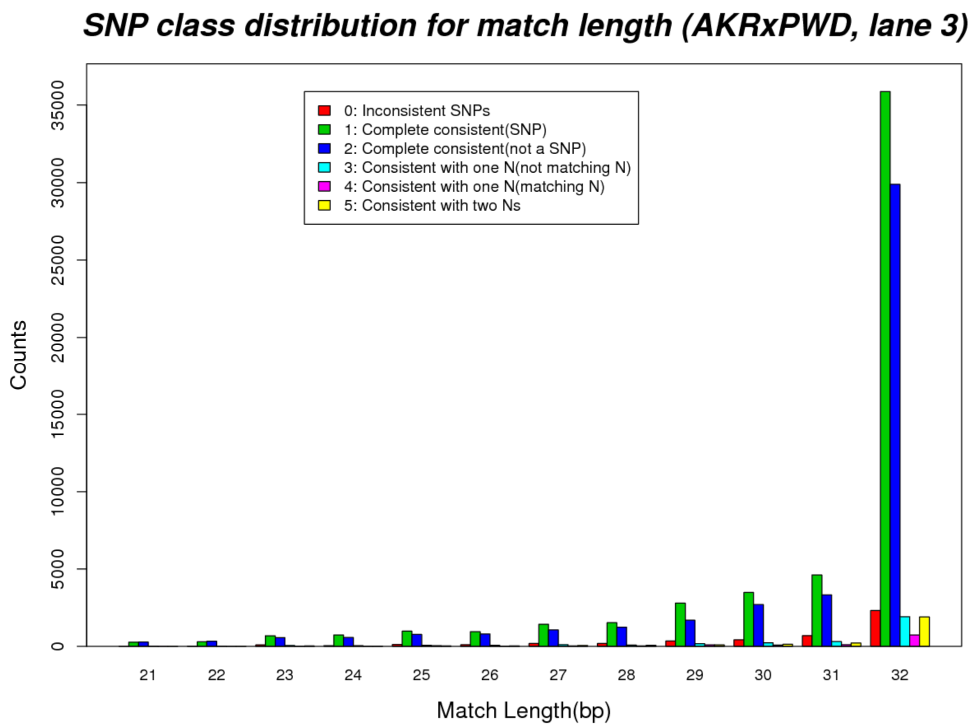

B 4). Match length of Solexa SNPs of lane 4, AKR x PWD, by SNP class.

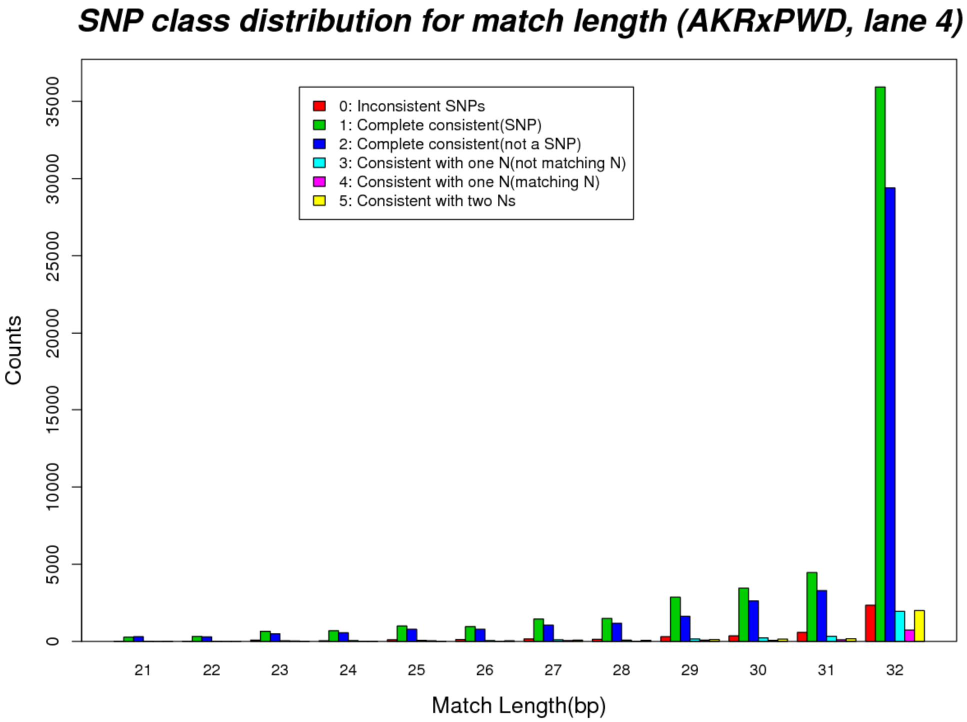

B 5). Match length of Solexa SNPs of lane 5, AKR x PWD, by SNP class.

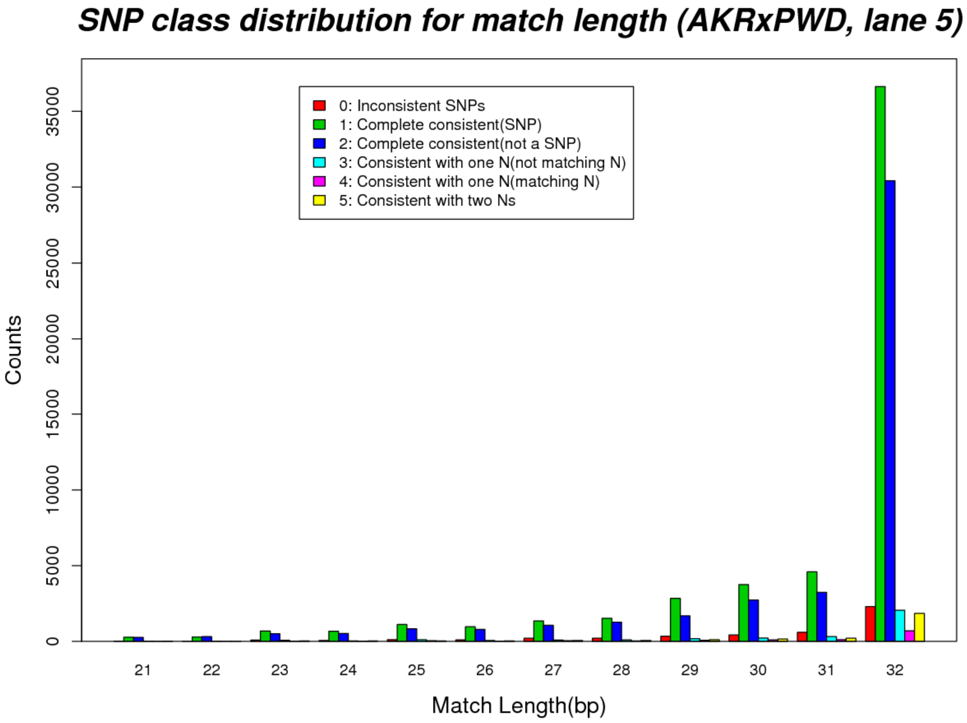

B 6). Match length of Solexa SNPs of lane 6, AKR x PWD, by SNP class.

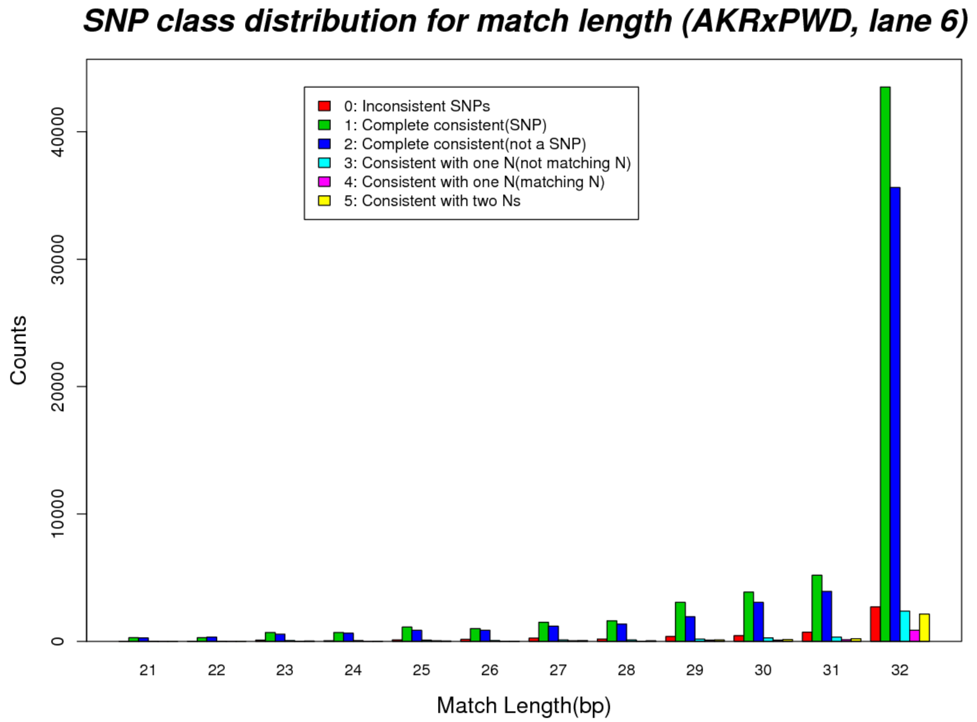

B 7). Match length of Solexa SNPs of lane 7, AKR x PWD, by SNP class.

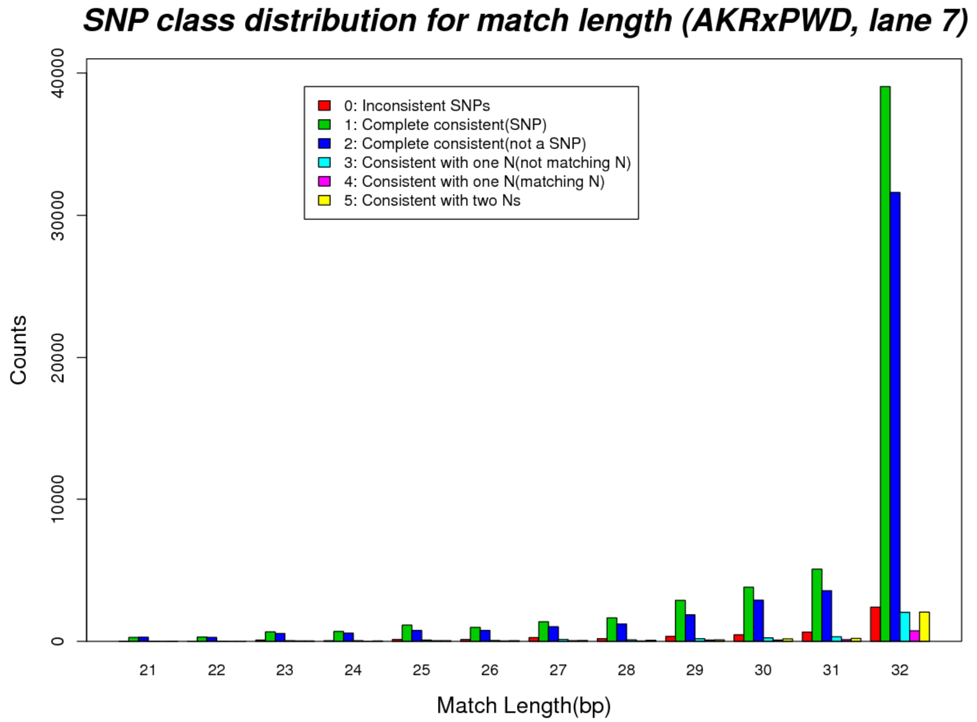

B 8). Match length of Solexa SNPs of lane 8, AKR x PWD, by SNP class.

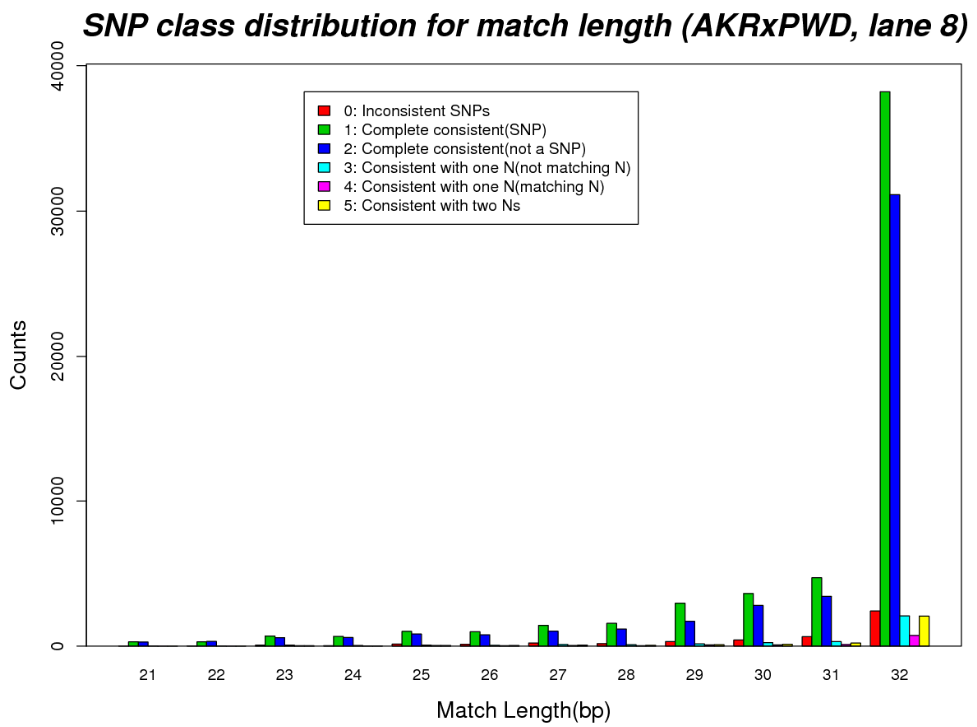

Figure S1.18. The quality score distribution of the Solexa SNPs. A, Match length distribution for the seven lanes in the Solexa run of the PWD x AKR sample. **B,** Match length distribution for the eight lanes in the Solexa run of the AKR x PWD sample.

Figure S1.19. SNP calling for the Perlegen missing data.

A. Summary of coverage of the Perlegen RefSeq SNPs.

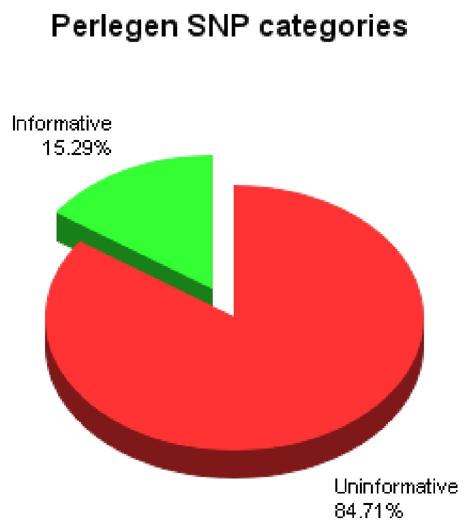

B. Summary of the Solexa Informative SNPs.

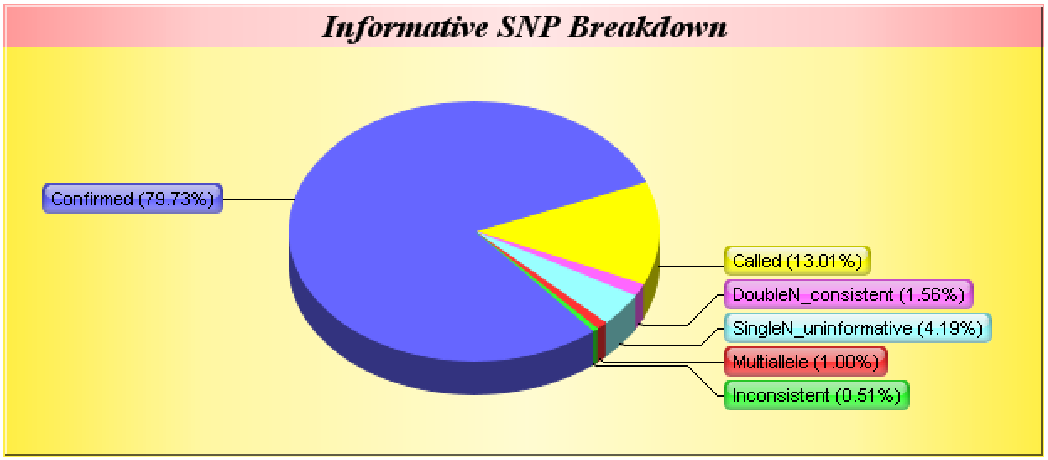

Figure S1.19. SNP calling for the Perlegen missing data. A, Summary of coverage of the Perlegen RefSeq SNPs. Informative: SNP counts >= 1 in both the two reciprocal crosses. **B,** Summary of the Solexa Informative SNPs. Confirmed: Perlegen SNPs that present in Soelxa and the genotypes agree with each other. Called: Perlegen SNPs with missing data in AKR and PWD that called based on the Solexa information.

Figure S1.20. Coverage distribution in the Solexa data.

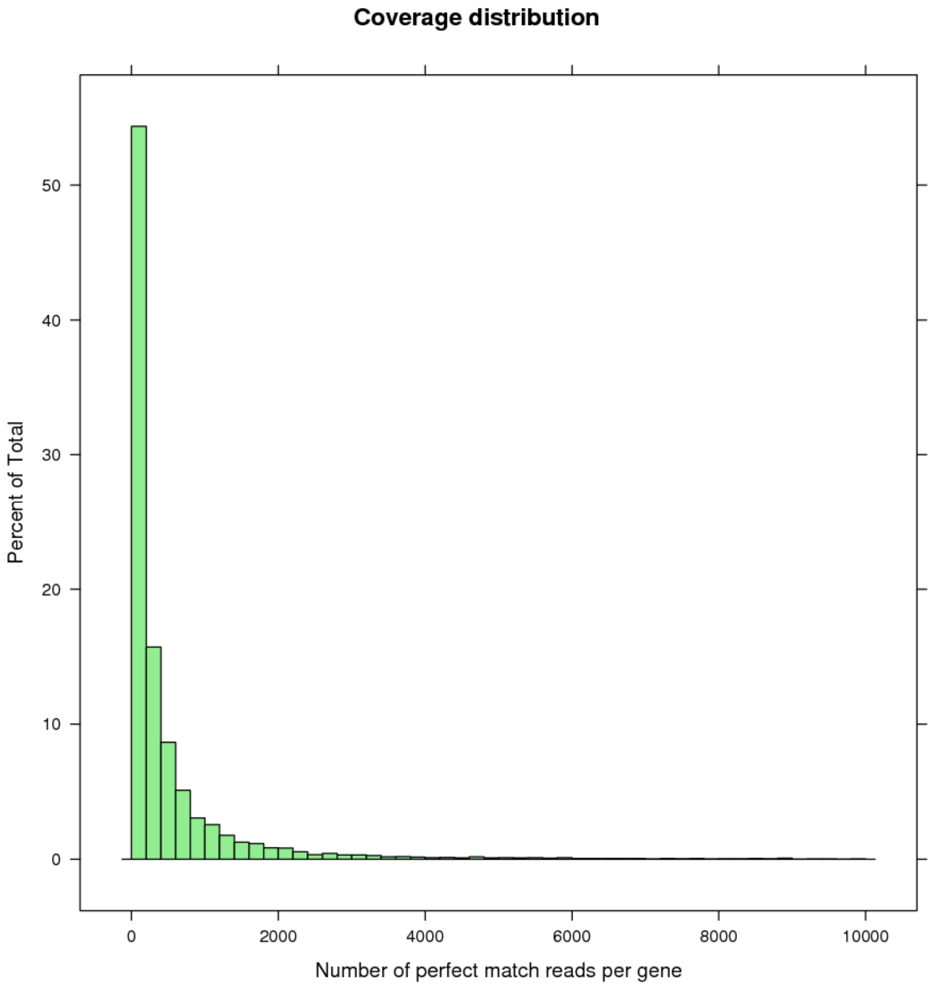

Figure S1.20. Coverage distribution in the Solexa data. Histogram of number of perfect match reads per gene for 15,491 RefSeq genes covered by at least one perfect match read in each of the two reciprocal crosses. (Only RefSeq genes with 10,000 or less perfect match reads are shown. There are 124 genes with number of perfect match reads > 10,000.)
