# Supplementary material for: Transcriptome-Wide Identification of Novel Imprinted Genes in Neonatal Mouse Brain
Source: PLoS One. 2008 Dec 4;3(12):e3839. doi: 10.1371/journal.pone.0003839 (PMC2585789; doi:10.1371/journal.pone.0003839)
Supplement: Table S1 — This file contains tables S1.1 through S1.19 as a bookmarked pdf. (0.35 MB PDF) [file pone.0003839.s002.pdf]

Table S1.1 Summary of blastn results

Cross	Lane	Total reads	Matched reads	Percentage of match	No match	Poly A/N*	Total hits	hits/read
PWD x AKR	s1	4,619,970	1,202,604	26.03%	3,409,387	7,979	4,369,063	3.63
	s2	4,295,871	1,200,111	24.73%	3,088,792	6,968	4,408,399	3.67
	s3	4,722,842	1,095,349	23.19%	3,615,132	12,361	3,852,524	3.52
	s4	4,853,113	1,126,465	23.21%	3,713,847	12,801	4,009,494	3.56
	s6	5,158,778	1,193,386	23.13%	3,953,819	11,573	4,249,988	3.56
	s7	5,053,146	1,173,701	23.23%	3,868,074	11,371	4,178,607	3.56
	s8	4,816,019	1,116,624	23.19%	3,688,339	11,056	3,919,609	3.51
AKR x PWD	s1	4,096,916	1,241,763	30.31%	2,777,901	77,252	3,749,816	3.02
	s2	4,339,623	1,322,613	30.48%	2,922,408	94,602	3,996,721	3.02
	s3	4,447,068	1,361,104	30.61%	2,990,873	95,091	4,126,160	3.03
	s4	4,397,822	1,348,417	30.66%	2,956,351	93,054	4,073,444	3.02
	s5	4,399,210	1,369,262	31.13%	2,932,067	97,881	4,150,208	3.03
	s6	4,509,790	1,417,377	31.43%	2,992,308	100,105	4,289,787	3.03
	s7	4,493,249	1,444,386	32.15%	2,946,392	102,471	4,345,896	3.01
	s8	4,827,209	1,576,487	32.66%	3,140,904	109,818	4,746,400	3.01

*: Low complexity reads including polyA and polyT

Table S1.2. Criteria for SNP filters of the Solexa data

Filter 1
• Type 1 SNP only.<br>• Q-score of the SNP position >= 10<br>• Mismatch score for reads containing reference allele: 3<br>• Mismatch score for reads containing alternative allele: 4
**Filter 2**
• Type 1 SNP only.<br>• Q-score of the SNP position >= 10<br>• SNP within 1.3kb to the 3'-end.<br>• Mismatch score for reads containing reference allele: 3<br>• Mismatch score for reads containing alternative allele: 4<br>• Match length >= 28.

Table S1.3. Summary of gene coverage and total SNP counts after filtering.

Filter	Gene Coverage***		PWD x AKR		AKR x PWD	
	RefSeq genes	Entrez gene	AKR*	PWD**	AKR*	PWD**
Filter 1	5,533	5,076	175,560 (54.73%)	145,244 (45.27%)	174,300 (53.23%)	153,151 (46.77%)
Filter 2	4,467	4,116	145,778 (54.47%)	121,853 (45.53%)	133,507 (52.44%)	121,096 (47.56%)

*: Total counts of AKR alleles
**: Total counts of PWD alleles
***: Genes covered by a total count of 4 or more in both cross

Table S1.4. Definition of p_1 and p_2.

	AKR allele counts	PWD allele counts	
**PWD x AKR**	a	b	$\boldsymbol{p_1} = a/(a+b)$
**AKR x PWD**	c	d	$\boldsymbol{p_2} = c/(c+d)$

Table S1.5. Detecting genomic imprinting.

$p_1 \approx p_2 \approx 0.5$	Relatively equal expression from the two parental copies.
$p_1 \approx p_2 > 0.5$	eQTL with higher expression from the AKR strain
$p_1 \approx p_2 < 0.5$	eQTL with higher expression from the PWD strain
$p_1 \neq p_2$, $p_1 > 0.5$, $p_2 < 0.5$	Paternally expressed imprinted candidate gene
$p_1 \neq p_2$, $p_1 < 0.5$, $p_2 > 0.5$	Maternally expressed imprinted candidate gene

Table S1.6. Summary of known imprinted genes covered in Solexa reads.

Type	Count	Description
No_RefSeq	37	The gene is not in the RefSeq database
No_SNP	16	There is no SNP in the RefSeq transcript.
No_counts	12	There is SNP site within the transcripts, but there is no counts in the filtered Solexa data.
Covered	33	The gene is covered in the Solexa data.

Table S1.7. Known imprinted genes covered in Solexa data.

MGIsymbol	Expressed allele	PWD x AKR		AKR x PWD		Storer-Kim p-value	Pyrosquencing		Reported imprinting status in brain and placenta	Reference	Total number of perfect ly matched reads
		AKR_count	PWD_count	AKR_count	PWD_count		p1	p2			
Gtl2 *	M	1	339	193	1	0	0.000	1.000	placenta	1	452
H19 *	M	2	14	61	1	1.68E-12	0.094	1.000	fetal brain	2	247
Cdkn1c *	M	0	8	13	0	9.46E-07	0.036	1.000	whole body of neonates	3	521
Commd1	M	12	33	22	7	2.52E-05	0.412	0.725	brain	4, 24	574
Ppp1r9a *	M	36	54	67	26	1.18E-05			placenta, partially in neonatal brain	5	2117
Asb4	M	3	5	8	0	0.01293			brain & placenta	6	158
Calcr	M	0	1	6	0	0.05666			embryonic and adult brain	7	77
Ube3a *	M	3	10	4	2	0.08215	0.392	0.755	brain	8	1660
Gnas *	M	181	214	101	96	0.21673	0.459	0.562	other (embryos), Imprinted in pituitary	9	15998
Gatm	M	4	5	15	7	0.22627			placenta	10	637
Tnfrsf23	M	2	2	2	0	0.37311			placenta	11	17[$]
Zim1	M	0	1	2	1	0.5			other tissue, biallelic in neonatal brain	12	19[$]
Dcn	M	16	10	4	4	0.59255			placenta	6	344
Nap1l4 *	M	9	12	181	214	0.79689			placenta	13	1175
Osbpl5	M	3	2	5	4	0.96653			placenta	13	157
Grb10 *	M	2	3	6	10	0.98295	0.609	0.522	placenta and brain	14, 15	508
Slc22a2	M	0	1	0	1	1			placenta	16	11[$]
Nnat	P	1182	1	21	1853	0	1.000	0.000	neonatal brain	17	8561
Snurf	P	888	1	1	18	0	1.000	0.000	neonatal and adult brain	18	23679
Snrpn	P	889	1	1	19	0	1.000	0.000	neonatal and adult brain	18	23310
Peg13 *	P	168	0	6	74	0	0.988	0.030	adult brain	19	1088
Nap1l5 *	P	22	0	0	67	2.42E-22	1.000	0.000	adult brain	19	2329
Inpp5f_v2	P	38	3	14	80	4.29E-19	0.919	0.078	neonatal brain	20	5509
Sgce	P	9	0	0	54	6.01E-12			adult brain & placenta	21	313
Rasgrf1 *	P	16	0	0	20	2.56E-11	1.000	0.000	neonatal brain	22	93
Impact	P	15	6	8	83	6.24E-09	0.791	0.198	embryonic and adult brain	23	1953
Zrsr1	P	11	0	1	14	4.48E-07	0.975	0.004	embryonic, neonatal and adult brain	24	204
Magel2	P	2	0	0	5	0.01615			adult brain	25	171
Rtl1	P	0	3	2	0	0.0576			brain and placenta	26	62
Peg3	P	2	0	0	2	0.125			embryonic, neonatal and adult brain	27	52
Igf2 *	P	52	43	20	27	0.17846	0.641	0.499	placenta, biallelic in fetal brain	28, 2	247
Copg2	P	0	1	2	0	0.22222			embryonic, neonatal and adult brain	29	302
Slc38a4	P	2	2	12	6	0.58003			brain and placenta	6	45[$]

*: assembly information used.

[$]: Low expression level.

known maternally expression imprinted genes that are significant in the Solexa data
known maternally expression imprinted genes that are not significant in the Solexa data
known paternally expression imprinted genes that are significant in the Solexa data
known paternally expression imprinted genes that are not significant in the Solexa data

References:

[1] J. V. Schmidt, P. G. Matteson, B. K. Jones et al., *Genes & development* **14** (16), 1997 (2000).
[2] M. Hemberger, C. Redies, R. Krause et al., *Development genes and evolution* **208** (7), 393 (1998).
[3] I. Hatada and T. Mukai, *Nature genetics* **11** (2), 204 (1995).
[4] Z. Zhang, K. Joh, H. Yatsuki et al., *Gene* **366** (1), 77 (2006).
[5] R. Ono, H. Shiura, H. Aburatani et al., *Genome Res* **13** (7), 1696 (2003).
[6] Y. Mizuno, Y. Sotomaru, Y. Katsuzawa et al., *Biochemical and biophysical research communications* **290** (5), 1499 (2002).
[7] H. Hoshiya, M. Meguro, A. Kashiwagi et al., *Journal of human genetics* **48** (4), 208 (2003).
[8] U. Albrecht, J. S. Sutcliffe, B. M. Cattanach et al., *Nature genetics* **17** (1), 75 (1997).
[9] J. Peters, S. F. Wroe, C. A. Wells et al., *Proceedings of the National Academy of Sciences of the United States of America* **96** (7), 3830 (1999).
[10] L. L. Sandell, X. J. Guan, R. Ingram et al., *Proceedings of the National Academy of Sciences of the United States of America* **100** (8), 4622 (2003).
[11] L. Clark, M. Wei, G. Cattoretti et al., *BMC genetics* **3**, 11 (2002).
[12] J. Kim, X. Lu, and L. Stubbs, *Human molecular genetics* **8** (5), 847 (1999).
[13] S. Engemann, M. Strodicke, M. Paulsen et al., *Human molecular genetics* **9** (18), 2691 (2000).
[14] T. Hikichi, T. Kohda, T. Kaneko-Ishino et al., *Nucleic acids research* **31** (5), 1398 (2003).
[15] N. Miyoshi, Y. Kuroiwa, T. Kohda et al., *Proceedings of the National Academy of Sciences of the United States of America* **95** (3), 1102 (1998).
[16] R. Zwart, F. Sleutels, A. Wutz et al., *Genes & development* **15** (18), 2361 (2001).
[17] F. Kagitani, Y. Kuroiwa, S. Wakana et al., *Nucleic acids research* **25** (17), 3428 (1997).
[18] S. E. Leff, C. I. Brannan, M. L. Reed et al., *Nature genetics* **2** (4), 259 (1992).
[19] R. J. Smith, W. Dean, G. Konfortova et al., *Genome Res* **13** (4), 558 (2003).
[20] J. D. Choi, L. A. Underkoffler, A. J. Wood et al., *Molecular and cellular biology* **25** (13), 5514 (2005).
[21] G. Piras, A. El Kharroubi, S. Kozlov et al., *Molecular and cellular biology* **20** (9), 3308 (2000).
[22] C. Plass, H. Shibata, I. Kalcheva et al., *Nature genetics* **14** (1), 106 (1996).
[23] Y. Hagiwara, M. Hirai, K. Nishiyama et al., *Proceedings of the National Academy of Sciences of the United States of America* **94** (17), 9249 (1997).
[24] Y. Wang, K. Joh, S. Masuko et al., *Molecular and cellular biology* **24** (1), 270 (2004).
[25] I. Boccaccio, H. Glatt-Deeley, F. Watrin et al., *Human molecular genetics* **8** (13), 2497 (1999).
[26] H. Seitz, N. Youngson, S. P. Lin et al., *Nature genetics* **34** (3), 261 (2003).
[27] Y. Kuroiwa, T. Kaneko-Ishino, F. Kagitani et al., *Nature genetics* **12** (2), 186 (1996).
[28] T. M. DeChiara, E. J. Robertson, and A. Efstratiadis, *Cell* **64** (4), 849 (1991).
[29] Y. J. Lee, C. W. Park, Y. Hahn et al., *FEBS letters* **472** (2-3), 230 (2000).

Table S1.8. Sense-antisense pairs in known imprinted genes.

Chr band	Type	MGIsymbol	Expressed allele	Description	Reference
2 E1-H3	coding-gene	Gnas	M	Stimulatory G-protein, alpha subunit	1,2
2 E1-H3	antisense	Nespas	P	Nesp antisense	
6 A3	coding-gene	Copg2	P	Coatomer protein complex subunit	3
6 A3	antisense	Copg2as2	M	COPG2 antisense	
7 B5	coding-gene	Mkrn3 (Zfp127)	P	Makorin, ring finger protein	4
7 B5	antisense	Zfp127as	P	Mkrn3 antisense	
7 A2-B1	coding-gene	Usp29	P	Ubiquitin-specific protease	5
7 A2-B1	antisense	Zim3	M	antisense of Usp29	6
7 B5	coding-gene	Ube3a	M	Ubiquitin protein ligase	7
7 B5	antisense	UBE3A-AS	P	UBE3A antisense	8
7 F5	coding-gene	Igf2	P	Insulin-like growth factor 2	9
7 F5	antisense	Igf2as	P	IGF2 antisense	10
7 F5	coding-gene	Kcnq1	M	Voltage-gated potassium channel	11,12
7 F5	antisense	Kcnq1ot1	P	KCNQ1 antisense	13
12 F1	coding-gene	Rtl1(Peg11)	P	Retrotransposon-like 1, like gag protein	14
12 F1	antisense	anti-Rtl1	M	Rtl1 antisense	14
17 A1	coding-gene	Igf2r	M	Insulin-like growth factor receptor 2	15
17 A1	antisense	Air	P	Igf2r antisense	16
X D	Non-coding RNA	Xist	P	XIST	17
X D	antisense	Tsix	M	XIST antisense	18

Stain used and references:

#	Strains used	Reference
1	MatDp.dist2 (maternal duplivation) and PatDp.dist2 (paternal duplication) from C3H/HeH	1. Peters J, Wroe SF, Wells CA, Miller HJ, Bodle D, et al. (1999) A cluster of oppositely imprinted transcripts at the Gnas locus in the distal imprinting region of mouse chromosome 2. Proc Natl Acad Sci U S A 96: 3830-3835.
2	C57BL/6J X CBA/Ca	2. Coombes C, Arnaud P, Gordon E, Dean W, Coar EA, et al. (2003) Epigenetic properties and identification of an imprint mark in the Nesp-Gnasxl domain of the mouse Gnas imprinted locus. Mol Cell Biol 23: 5475-5488.
3	C57BL/6J X KJR/Msf	3. Lee YJ, Park CW, Hahn Y, Park J, Lee J, et al. (2000) Mit1/Lb9 and Copg2, new members of mouse imprinted genes closely linked to Peg1/Mest(1). FEBS Lett 472: 230-234.

4	129/RI × M.spretus	4. Jong MT, Carey AH, Caldwell KA, Lau MH, Handel MA, et al. (1999) Imprinting of a RING zinc-finger encoding gene in the mouse chromosome region homologous to the Prader-Willi syndrome genetic region. Hum Mol Genet 8: 795-803.
5	129/Sv and C57BL/6J BAC library	5. Kim J, Noskov VN, Lu X, Bergmann A, Ren X, et al. (2000) Discovery of a novel, paternally expressed ubiquitin-specific processing protease gene through comparative analysis of an imprinted region of mouse chromosome 7 and human chromosome 19q13.4. Genome Res 10: 1138-1147.
6	cDNA from 129/Sv, BALB/c, C3H and M.spretus	6. Kim J, Bergmann A, Wehri E, Lu X, Stubbs L (2001) Imprinting and evolution of two Kruppel-type zinc-finger genes, ZIM3 and ZNF264, located in the PEG3/USP29 imprinted domain. Genomics 77: 91-98.
7	mouse with partial UPD (uniparental disomy), strain not mentioned in the paper	7. Albrecht U, Sutcliffe JS, Cattanach BM, Beechey CV, Armstrong D, et al. (1997) Imprinted expression of the murine Angelman syndrome gene, Ube3a, in hippocampal and Purkinje neurons. Nat Genet 17: 75-78.
8	CAST.c7 x129/Sv	8. Chamberlain SJ, Brannan CI (2001) The Prader-Willi syndrome imprinting center activates the paternally expressed murine Ube3a antisense transcript but represses paternal Ube3a. Genomics 73: 316-322.
9	129/Sv/Ev, MF1, BALB/c, DBA/2J and C57BL/6J	9. DeChiara TM, Robertson EJ, Efstratiadis A (1991) Parental imprinting of the mouse insulin-like growth factor II gene. Cell 64: 849-859.
10	C57BL/6 x CBA	10. Moore T, Constancia M, Zubair M, Bailleul B, Feil R, et al. (1997) Multiple imprinted sense and antisense transcripts, differential methylation and tandem repeats in a putative imprinting control region upstream of mouse Igf2. Proc Natl Acad Sci U S A 94: 12509-12514.
11	BAC, YAC and cDNA library from C57BL/6	11. Paulsen M, Davies KR, Bowden LM, Villar AJ, Franck O, et al. (1998) Syntenic organization of the mouse distal chromosome 7 imprinting cluster and the Beckwith-Wiedemann syndrome region in chromosome 11p15.5. Hum Mol Genet 7: 1149-1159.
12	FVB × M.castaneus	12. Gould TD, Pfeifer K (1998) Imprinting of mouse Kvlqt1 is developmentally regulated. Hum Mol Genet 7: 483-487.
13	PWK x C57BL/6	13. Fitzpatrick GV, Soloway PD, Higgins MJ (2002) Regional loss of imprinting and growth deficiency in mice with a targeted deletion of KvDMR1. Nat Genet 32: 426-431.
14	UPD (uniparental disomy) mice, strain not mentioned in the paper	14. Seitz H, Youngson N, Lin SP, Dalbert S, Paulsen M, et al. (2003) Imprinted microRNA genes transcribed antisense to a reciprocally imprinted retrotransposon-like gene. Nat Genet 34: 261-262.
15	BLAB/c x C3H	15. Barlow DP, Stoger R, Herrmann BG, Saito K, Schweifer N (1991) The mouse insulin-like growth factor type-2 receptor is imprinted and closely linked to the Tme locus. Nature 349: 84-87.
16	C57BL/c × M.spretus	16. Hu JF, Balaguru KA, Ivaturi RD, Oruganti H, Li T, et al. (1999) Lack of reciprocal genomic imprinting of sense and antisense RNA of mouse insulin-like growth factor II receptor in the central nervous system. Biochem Biophys Res Commun 257: 604-608.
17	C57BL/6J X CBA/Ca	17. Kay GF, Barton SC, Surani MA, Rastan S (1994) Imprinting and X chromosome counting mechanisms determine Xist expression in early mouse development. Cell 77: 639-650.
18	C57BL/6 x JF1	18. Sado T, Wang Z, Sasaki H, Li E (2001) Regulation of imprinted X-chromosome inactivation in mice by Tsix. Development 128: 1275-1286.

Table S1.9. Closely linked and reciprocal imprinted genes covered in Solexa data.

Gene_name	Chr	PWD x AKR		AKR x PWD		q-value	AKR percentage		IP status
		AKR	PWD	AKR	PWD		p1	p2	
Peg13	chr15	168	0	6	74	0	1.000	0.075	100%
1810044A24Rik	chr15	7	20	25	5	1.08E-03	0.259	0.833	Partially
Nnat	chr2	1182	1	21	1853	0	0.999	0.011	100%
Blcap	chr2	6	13	15	2	0.0252	0.316	0.882	partially
Zrsr1	chr11	11	0	1	14	6.68E-05	1.000	0.067	100%
Commd1	chr11	12	33	22	7	2.55E-03	0.267	0.759	partially

Table S1.10. Non-significant genes with total counts 25 or more in each of the two reciprocal crosses.

RefSeq_ID	PWD x AKR		AKR x PWD		Gene_name	Chr	RefSeq_len	B37_SNP_position	q-value	p1	p2
	AKR	PWD	AKR	PWD							
NM_009438	221	199	18	9	Sntg1	chr1	4193	9193264	0.37945	0.895	0.667
NM_010517	15	14	61	42	3110035E14Rik	chr1	3187	9616434	0.947901	0.571	0.592
NM_026302	16	26	74	32	Stau2	chr1	2198	16334335	0.947901	0.667	0.698
NM_012021	59	26	106	86	Gdap1	chr1	3934	17151276	0.852521	0.484	0.552
NM_177681	36	17	27	21	Ogfrl1	chr1	4844	23375818	0.947901	0.5	0.563
NM_021548	104	89	34	13	1110058L19Rik	chr1	660	24010006	0.947901	0.667	0.723
NM_007502	77	57	48	58	Bai3	chr1	5457	25185678	0.947901	0.417	0.453
NM_013917	91	126	158	45	Ptp4a1	chr1	4153	31001822	0.419101	0.654	0.778
NM_133837	52	104	71	97	B230209C24Rik	chr1	2318	33940325	0.712972	0.527	0.423
NM_008974	39	40	13	17	Dst	chr1	17277	34172986	0.947901	0.391	0.433
NM_057172	19	10	22	21	BC043098	chr1	4955	34870617	0.855763	0.607	0.512
NM_080555	20	28	49	36	Plekhb2	chr1	3317	34934064	0.947901	0.63	0.576
NM_033623	21	19	26	21	4632411B12Rik	chr1	4663	36414969	0.80737	0.659	0.553
NM_180678	58	40	17	29	Actr1b	chr1	2077	36758142	0.947901	0.412	0.37
NM_001042634	187	103	368	242	Mrpl30	chr1	691	37954568	0.504192	0.669	0.603
NM_001039934	125	195	37	38	Txndc9	chr1	4596	38043980	0.947901	0.429	0.493
NM_134065	30	14	55	44	Eif5b	chr1	7797	38108780	0.947901	0.603	0.556
NM_011261	54	40	26	20	Rev1	chr1	4279	38155856	0.947901	0.593	0.565
NM_025550	22	20	111	89	Chst10	chr1	3135	38954911	0.537791	0.465	0.555
NM_199021	33	16	139	62	Pdcl3	chr1	1706	39053906	0.405723	0.581	0.692
NM_010684	119	74	56	31	Mrps9	chr1	1365	42962311	0.677707	0.516	0.644
NM_009133	128	148	85	94	Tpp2	chr1	4649	44058975	0.935339	0.423	0.475
NM_146165	193	54	29	23	Bivm	chr1	3529	44200106	0.947901	0.493	0.558
NM_001013778	58	49	26	13	Col3a1	chr1	4786	45403996	0.915297	0.767	0.667
NM_011436	16	28	47	35	Wdr75	chr1	2612	45879770	0.947901	0.625	0.573
NM_027009	18	24	29	22	Tmeff2	chr1	3340	51242688	0.947901	0.6	0.569
NM_007793	198	77	49	54	Myo1b	chr1	4933	51853906	0.783653	0.613	0.476
NM_172562	84	117	39	34	Sf3b1	chr1	6192	55068964	0.805861	0.659	0.534
NM_015762	21	7	88	42	Prei3	chr1	2845	55210999	0.775128	0.59	0.677
NM_009670	27	25	34	20	Satb2	chr1	5299	56954826	0.903782	0.54	0.63
NM_001030274	165	154	50	55	Clk1	chr1	1818	58480848	0.097758	0.645	0.476
NM_025790	62	45	116	43	Ndufb3	chr1	532	58652657	0.601754	0.615	0.73
NM_152810	22	26	34	39	Als2	chr1	6348	59294046	0.947901	0.542	0.466
NM_139272	35	18	85	94	Ndufs1	chr1	2564	63199462	0.847106	0.542	0.475
NM_009130	36	31	184	154	Eef1b2	chr1	857	63225106	0.467305	0.629	0.544
NM_019571	20	28	59	71	Adam23	chr1	6656	63642430	0.459791	0.606	0.454
NM_019393	126	185	166	94	Idh1	chr1	2270	65217542	0.063455	0.477	0.638
NM_011653	727	388	370	788	Mtap2	chr1	5535	66485497	0.301202	0.391	0.32
NM_008187	11	18	17	23	Lancl1	chr1	4270	67085096	0.947901	0.419	0.425
NM_007998	35	67	16	20	Fn1	chr1	8312	71695775	0.80737	0.585	0.444
NM_207650	53	22	48	36	Mar4	chr1	4217	72582049	0.947901	0.54	0.571
NM_007694	151	113	84	82	Rpl37a	chr1	431	72760121	0.942332	0.567	0.506
NM_020494	99	114	135	104	Igfbp5	chr1	5854	72920447	0.903782	0.605	0.565
NM_027289	44	21	76	102	Arpc2	chr1	1597	74314392	0.525209	0.527	0.427
NM_021518	21	12	40	31	Dnajb10	chr1	1855	75241700	0.58647	0.445	0.563
NM_010064	91	72	12	15	Farsb	chr1	2132	78440320	0.947901	0.448	0.444
NM_011955	44	33	39	30	Mrpl44	chr1	1424	79777605	0.624757	0.75	0.565

NM_008963	218	154	135	95	Serpine2	chr1	2151	79813448	0.947901	0.574	0.587
NM_029447	178	67	211	244	5230400G24Rik	chr1	1920	82748413	0.947901	0.452	0.464
NM_029814	58	117	62	69	4930544G21Rik	chr1	6841	83285397	0.498119	0.644	0.473
NM_025624	1149	1459	401	377	Dner	chr1	3664	84442060	0.915297	0.552	0.515
NM_133922	7	19	23	9	Itm2c	chr1	2024	87804656	0.947901	0.66	0.719
NM_001037999	61	77	21	14	Eif4e2	chr1	3394	89136200	0.947901	0.571	0.6
NM_001005223	58	31	16	9	Atg16l1	chr1	3066	89687176	0.947901	0.64	0.64
NM_018804	149	157	28	7	Hdac4	chr1	3960	93926709	0.762643	0.671	0.8
NM_013899	38	65	34	27	Ndufa10	chr1	1222	94367466	0.947901	0.495	0.557
NM_145398	19	22	29	30	Rnpepl1	chr1	2489	94812486	0.744899	0.32	0.492
NM_009819	42	21	17	16	Mterfd2	chr1	3315	95201238	0.815655	0.651	0.515
NM_008971	33	13	27	16	Bok	chr1	1432	95591943	0.947901	0.625	0.628
NM_181394	25	17	180	198	Tsn	chr1	3040	120205360	0.947901	0.443	0.476
NM_012015	18	11	38	30	Mki67ip	chr1	1646	120229541	0.947901	0.556	0.559
NM_178764	127	122	21	24	Clasp1	chr1	7827	120504980	0.941916	0.371	0.467
NM_026069	138	108	150	311	Dbi	chr1	593	122009935	0.255447	0.442	0.325
NM_010497	71	78	36	55	Dpp10	chr1	4634	125750758	0.071722	0.673	0.396
NM_011294	106	109	35	19	Ubxd2	chr1	4093	130175429	0.941663	0.552	0.648
NM_175398	17	9	9	25	Mcm6	chr1	2901	130256041	0.355031	0.545	0.265
NM_009443	29	22	34	21	Pfkfb2	chr1	4506	132611110	0.947901	0.571	0.618
NM_001081360	21	11	55	67	Srgap2	chr1	8040	133260600	0.901101	0.517	0.451
NM_024243	34	43	20	23	Slc41a1	chr1	4752	133743597	0.525209	0.66	0.465
NM_177298	122	124	5	21	Ripk5	chr1	6385	134363066	0.813566	0.333	0.192
NM_009308	16	10	46	34	Zc3h11a	chr1	4395	135543992	0.813566	0.483	0.575
NM_025509	40	45	47	40	Rnpep	chr1	2249	137180206	0.947901	0.462	0.54
NM_008721	160	155	53	52	Timm17a	chr1	917	137206414	0.947901	0.466	0.505
NM_029354	22	47	52	38	Nav1	chr1	8841	137368992	0.84529	0.657	0.578
NM_178880	30	13	70	47	Tmem9	chr1	1445	137930663	0.947901	0.638	0.598
NM_026676	15	72	109	59	Kif21b	chr1	9119	138070743	0.933057	0.598	0.649
NM_019913	179	146	36	58	Camsap1l1	chr1	7823	138221347	0.18768	0.656	0.383
NM_054053	28	4	30	19	Glrx2	chr1	3482	145596791	0.947901	0.654	0.612
NM_026780	27	32	72	77	Trove2	chr1	8736	145624090	0.947901	0.548	0.483
NM_011034	295	310	99	108	Tpr	chr1	7529	152296289	0.936394	0.429	0.478
NM_023312	157	111	69	76	Ivns1abp	chr1	3513	153210662	0.805861	0.37	0.476
NM_182997	22	20	163	185	Arpc5	chr1	1782	154621425	0.84104	0.402	0.468
NM_011406	35	19	51	91	Smg7	chr1	5840	154707590	0.947901	0.386	0.359
NM_026506	20	22	25	32	6430517E21Rik	chr1	4169	160285993	0.942325	0.525	0.439
NM_001038993	119	54	56	42	Astn1	chr1	7181	160587288	0.602323	0.455	0.571
NM_025515	22	12	279	168	Cacybp	chr1	1493	162138691	0.624757	0.549	0.624
NM_026845	43	27	15	14	Prdx6	chr1	2334	163174355	0.947901	0.434	0.517
NM_010887	41	40	195	160	Dnm3	chr1	7534	164283688	0.947901	0.573	0.549
NM_133703	55	27	353	262	Kifap3	chr1	3907	165845884	0.947901	0.574	0.574
NM_172401	33	29	19	18	Atp1b1	chr1	2422	166368147	0.947901	0.568	0.514
NM_019827	37	17	24	18	Mpzl1	chr1	2356	167522548	0.947901	0.622	0.571
NM_024284	9	23	24	14	Dusp12	chr1	1413	172815229	0.783653	0.786	0.632
NM_010699	49	139	49	65	Sdhc	chr1	1170	173080700	0.947901	0.455	0.43
NM_170593	36	52	80	55	Ndufs2	chr1	1606	173177139	0.682906	0.505	0.593
NM_024220	206	220	44	39	Ufc1	chr1	964	173224831	0.947901	0.554	0.53
NM_027400	63	71	13	21	Fh1	chr1	1588	177549194	0.785646	0.527	0.382
NM_010897	20	13	84	122	2310005N03Rik	chr1	448	180251876	0.710344	0.49	0.408
NM_130895	32	10	15	15	Smyd3	chr1	3769	181335462	0.726189	0.342	0.5
NM_144901	48	208	59	36	Srp9	chr1	1388	184061972	0.947901	0.553	0.621

NM_133218	121	92	27	21	Slc30a10	chr1	5836	187291763	0.945732	0.462	0.563
NM_009441	176	130	18	14	Nenf	chr1	658	193130695	0.673494	0.372	0.563
NM_019489	232	52	45	0	Plxna2	chr1	11049	196638409	0.947901	1	1
NM_001081251	77	48	23	11	Akap12	chr1	6198	5988621	0.792184	0.529	0.676
NM_015733	23	9	48	44	Ppp1r14c	chr1	2524	6922985	0.947901	0.567	0.522
NM_009136	16	14	64	39	Pcmt1	chr1	1580	7359885	0.947901	0.652	0.621
NM_025546	68	38	29	20	Stxbp5	chr1	9074	9584842	0.633314	0.393	0.592
NM_029274	37	33	18	17	Sf3b5	chr1	727	12728339	0.641017	0.67	0.514
NM_028105	22	23	36	27	Vta1	chr1	1343	14404779	0.947901	0.578	0.571
NM_198326	136	121	23	11	Reps1	chr1	2618	17842924	0.947901	0.593	0.676
NM_033370	10	27	287	308	Ahi1	chr1	4751	20798941	0.947901	0.515	0.482
NM_010360	26	31	69	95	Stx7	chr1	2500	23907460	0.301202	0.569	0.421
NM_022432	15	23	66	38	Ptprk	chr1	6177	28316448	0.947901	0.586	0.635
NM_008567	18	15	54	76	Echdc1	chr1	3048	29064982	0.72236	0.563	0.415
NM_011200	51	27	198	116	Hddc2	chr1	718	31047788	0.947901	0.659	0.631
NM_008298	48	34	18	12	Rwdd1	chr1	1082	33739246	0.943372	0.519	0.6
NM_025374	172	56	85	52	Tspyl1	chr1	2670	34004530	0.945732	0.543	0.62
NM_025291	50	60	67	122	Hdac2	chr1	2004	36721613	0.601949	0.5	0.354
NM_015824	47	26	19	9	Lama4	chr1	5663	38829765	0.86501	0.538	0.679
NM_001040699	20	29	90	65	Fyn	chr1	3258	39284534	0.805861	0.494	0.581
NM_176942	10	16	53	30	2410016F19Rik	chr1	1020	39977452	0.920095	0.574	0.639
NM_009946	71	58	63	45	Ostm1	chr1	3001	42421032	0.947901	0.604	0.583
NM_001008543	108	26	99	57	Cd24a	chr1	1825	43302439	0.920751	0.593	0.635
NM_028811	89	41	51	30	Gja1	chr1	3105	56107472	0.805861	0.519	0.63
NM_028632	57	71	74	78	Lims1	chr1	4244	57884466	0.947901	0.567	0.487
NM_011732	1064	1051	59	51	D10Ertd641e	chr1	1329	59465784	0.915297	0.619	0.536
NM_009908	94	70	89	85	Ascc1	chr1	1524	59562670	0.381503	0.667	0.511
NM_023172	447	324	47	39	Sar1a	chr1	2602	61154270	0.57016	0.393	0.547
NM_013581	10	15	82	30	Ank3	chr1	6966	69486740	0.189309	0.519	0.732
NM_031405	38	89	27	12	Ube2d1	chr1	1427	70718171	0.833193	0.813	0.692
NM_001081240	25	8	37	41	Specc1l	chr1	5923	74774985	0.933057	0.385	0.474
NM_177630	68	70	44	20	Ddt	chr1	609	75234005	0.947901	0.653	0.688
NM_013531	44	19	15	13	Dip2a	chr1	6356	75784304	0.947901	0.561	0.536
NM_021436	39	15	47	6	Pcbp3	chr1	2012	76355341	0.457473	0.735	0.887
NM_016797	62	47	23	26	Adarb1	chr1	6572	76822389	0.150615	0.762	0.469
NM_010838	68	18	16	12	1810008A18Rik	chr1	2415	76960124	0.947901	0.541	0.571
NM_133685	30	27	252	157	Cstb	chr1	620	77889740	0.158693	0.72	0.616
NM_053071	32	31	71	68	Polr2e	chr1	1022	79498817	0.923252	0.444	0.511
NM_008077	55	62	110	92	Rps15	chr1	500	79756489	0.947901	0.575	0.545
NM_024221	33	42	62	45	Apc2	chr1	9074	79780622	0.947901	0.576	0.579
NM_026684	42	82	27	20	Ap3d1	chr1	4730	80193682	0.86501	0.468	0.574
NM_021568	50	18	47	38	Timm13	chr1	1225	80362758	0.947901	0.493	0.553
NM_026254	24	11	36	31	Sgta	chr1	1966	80514970	0.947901	0.482	0.537
NM_010942	174	122	222	145	Eef2	chr1	3111	80639433	0.611212	0.55	0.605
NM_022310	55	36	17	9	Atcay	chr1	3677	80687264	0.859249	0.523	0.654
NM_007624	6	22	38	45	Nfyb	chr1	2552	82215284	0.947901	0.488	0.458
NM_178056	127	127	125	9	Txnrd1	chr1	3310	82358745	0.153968	0.75	0.933
NM_019780	157	141	41	53	Aldh1l2	chr1	5959	82990176	0.897731	0.524	0.436
NM_017367	67	47	15	34	Nuak1	chr1	5032	83843528	0.701206	0.463	0.306
NM_144518	28	23	73	47	Ckap4	chr1	2915	83989290	0.751919	0.71	0.608
NM_019413	41	37	29	20	Cry1	chr1	3035	84611372	0.677707	0.412	0.592
NM_019439	107	115	26	33	Prdm4	chr1	3935	85370149	0.947901	0.371	0.441

NM_009250	89	66	40	35	D10Wsu52e	chr1	1979	85418480	0.947901	0.491	0.533
NM_146193	39	52	21	6	Fbxo7	chr1	1886	85509537	0.947901	0.68	0.778
NM_134118	91	153	51	45	Timp3	chr1	4681	85808893	0.450421	0.31	0.531
NM_026388	84	113	44	60	Pmch	chr1	748	87553835	0.947901	0.458	0.423
NM_026937	48	24	29	51	4930547N16Rik	chr1	3628	87609280	0.841387	0.462	0.363
NM_025551	80	57	22	25	Ccdc53	chr1	1053	87708534	0.947901	0.482	0.468
NM_027117	14	18	47	26	Gnptab	chr1	5204	87909758	0.947901	0.615	0.644
NM_026516	36	34	125	89	Slc25a3	chr1	1512	90580853	0.947901	0.554	0.584
NM_026545	18	9	200	357	Metap2	chr1	4990	93353945	0.450833	0.451	0.359
NM_022004	202	164	174	192	Ndufa12	chr1	561	93666070	0.405723	0.584	0.475
NM_001037758	22	15	1709	1468	D10Ertd322e	chr1	666	94963110	0.947901	0.536	0.538
NM_027671	34	4	44	25	Ube2n	chr1	3851	95006417	0.805861	0.535	0.638
NM_148938	87	72	78	73	Nudt4	chr1	2929	95015165	0.805861	0.609	0.517
NM_011625	69	66	12	13	Atp2b1	chr1	4760	98478131	0.883593	0.59	0.48
NM_019648	69	84	27	13	Slc6a15	chr1	3642	102880726	0.947901	0.648	0.675
NM_008791	49	28	18	9	Ccdc59	chr1	1078	105284275	0.670341	0.462	0.667
NM_025391	18	10	89	53	Nap1l1	chr1	3927	110933474	0.947901	0.648	0.627
NM_178227	47	64	50	36	Krr1	chr1	3555	111422428	0.947901	0.618	0.581
NM_021896	50	53	128	69	4921506J03Rik	chr1	3607	112362814	0.601949	0.577	0.65
NM_026850	93	67	66	43	Cct2	chr1	1962	116499115	0.947901	0.604	0.606
NM_011780	43	28	50	34	Tmem5	chr1	1393	121526086	0.947901	0.659	0.595
NM_001081188	76	56	29	38	AI851790	chr1	4153	123175995	0.783653	0.536	0.433
NM_015729	28	22	25	20	Ctdsp2	chr1	4136	126436826	0.806039	0.4	0.556
NM_024191	69	23	27	33	Cdk4	chr1	1365	126504092	0.947901	0.484	0.45
NM_013540	88	77	22	12	Tspan31	chr1	1543	126505349	0.853013	0.762	0.647
NM_010307	119	94	240	142	Kif5a	chr1	6015	126685098	0.851121	0.589	0.628
NM_011595	9	20	19	15	Shmt2	chr1	2280	126954766	0.947901	0.529	0.559
NM_001004143	88	72	80	71	Tmem4	chr1	1017	127763251	0.947901	0.472	0.53
NM_010075	60	45	52	42	Pa2g4	chr1	2432	127994904	0.947901	0.5	0.553
NM_007465	19	11	35	37	Dnajc14	chr1	4230	128255928	0.915297	0.389	0.486
NM_133907	90	83	92	100	1110005A23Rik	chr1	893	128283624	0.896733	0.541	0.479
NM_001039368	79	43	163	165	Bloc1s1	chr1	550	128356995	0.947901	0.49	0.497
NM_027189	47	64	126	151	Adcy1	chr1	12259	7060286	0.780086	0.518	0.455
NM_145371	57	57	38	26	Vstm2	chr1	3229	16182547	0.947901	0.515	0.594
NM_026921	47	45	131	145	1500041B16Rik	chr1	1569	16978486	0.947901	0.511	0.475
NM_008628	27	25	102	35	Actr2	chr1	3659	19994389	0.947901	0.758	0.745
NM_133872	45	44	35	28	Rab1	chr1	2657	20124770	0.679858	0.696	0.556
NM_178055	129	161	55	31	Slc1a4	chr1	3885	20213948	0.947901	0.609	0.64
NM_028248	23	29	202	158	Mdh1	chr1	1758	21464206	0.844976	0.608	0.561
NM_011670	285	303	25	18	Spnb2	chr1	9049	30054247	0.798753	0.453	0.581
NM_001039466	34	34	386	280	Nsg2	chr1	2243	31958778	0.947901	0.561	0.58
NM_145558	21	33	20	24	Kcnip1	chr1	1783	33545623	0.677707	0.595	0.455
NM_080837	16	30	159	125	Pttg1	chr1	1683	43239116	0.076793	0.419	0.56
NM_001033474	278	204	13	15	Ublcp1	chr1	2051	44279510	0.947901	0.444	0.464
NM_001005868	28	16	56	74	Lsm11	chr1	6453	45746078	0.947901	0.45	0.431
NM_026879	50	61	11	14	Cyfip2	chr1	6352	46093543	0.947901	0.413	0.44
NM_026145	13	25	21	24	Gnb2l1	chr1	1254	48619668	0.947901	0.5	0.467
NM_029759	29	41	69	52	Mapk9	chr1	4356	49696910	0.947901	0.58	0.57
NM_172430	29	16	18	11	Rasgef1c	chr1	2248	49793052	0.805861	0.489	0.621
NM_138748	78	45	26	36	Sqstm1	chr1	2000	50013792	0.947901	0.475	0.419
NM_024258	6	24	67	33	Zfp354c	chr1	5367	50629149	0.863684	0.6	0.67
NM_029979	113	58	96	107	0610009B22Rik	chr1	795	51502066	0.866345	0.515	0.473

NM_008862	24	30	45	32	D11Ertd497e	chr1	1853	51790270	0.947901	0.535	0.584
NM_015827	11	16	58	58	Ube2b	chr1	1630	51799366	0.915297	0.419	0.5
NM_197985	19	15	117	72	Vdac1	chr1	1766	52202655	0.690932	0.527	0.619
NM_021388	23	27	33	36	9530068E07Rik	chr1	2467	52221577	0.933057	0.544	0.478
NM_134054	40	15	23	23	Rapgef6	chr1	8212	54511251	0.836801	0.633	0.5
NM_001025612	51	47	61	43	Cdc42se2	chr1	2985	54569915	0.641017	0.463	0.587
NM_173865	35	18	18	15	Anxa6	chr1	2661	54825227	0.947901	0.563	0.545
NM_011149	51	47	164	152	Sparc	chr1	2299	55212138	0.633314	0.59	0.519
NM_018796	154	91	40	28	Atox1	chr1	501	55263977	0.555395	0.405	0.588
NM_009720	17	25	20	13	G3bp1	chr1	2685	55313186	0.947901	0.519	0.606
NM_144525	23	11	23	21	Cops3	chr1	1583	59650473	0.947901	0.6	0.523
NM_026932	194	375	120	62	Usp22	chr1	4280	60977268	0.457616	0.55	0.659
NM_177167	43	31	29	30	Aldh3a2	chr1	3131	61075732	0.947901	0.538	0.492
NM_007510	270	223	16	20	Epn2	chr1	4209	61359731	0.817221	0.567	0.444
NM_024256	33	13	26	18	Prpsap2	chr1	1857	61543259	0.80737	0.493	0.591
NM_176930	18	18	36	22	Ttc19	chr1	3419	62128125	0.947901	0.643	0.621
NM_029293	84	66	68	19	Zfp287	chr1	5021	62542211	0.947901	0.771	0.782
NM_181400	44	58	45	22	AU040829	chr1	4099	64880584	0.947901	0.685	0.672
NM_008980	48	36	22	19	Map2k4	chr1	3694	65569809	0.947901	0.564	0.537
NM_025597	48	30	89	112	Trappc1	chr1	745	69139248	0.947901	0.413	0.443
NM_207205	63	23	41	67	Nlgn2	chr1	5034	69640383	0.844976	0.457	0.38
NM_016892	252	357	1032	825	Psmb6	chr1	798	70340749	0.481745	0.523	0.556
NM_133955	24	40	7	23	Spag7	chr1	1017	70477602	0.846223	0.359	0.233
NM_001081109	16	16	28	14	4933427D14Rik	chr1	4332	72011984	0.947901	0.667	0.667
NM_009120	24	37	36	15	Med31	chr1	1179	72028485	0.947901	0.747	0.706
NM_021336	35	31	29	37	Pafah1b1	chr1	5831	74512912	0.947901	0.483	0.439
NM_030254	28	25	40	34	Tsr1	chr1	3395	74720883	0.947901	0.525	0.541
NM_026058	24	8	38	36	Srr	chr1	3168	74723217	0.947901	0.515	0.514
NM_007505	87	71	99	78	Crk	chr1	3620	75519415	0.838727	0.68	0.559
NM_025797	58	51	29	22	Blmh	chr1	2240	76800051	0.947901	0.517	0.569
NM_198831	39	28	76	45	Taok1	chr1	12411	77373322	0.947901	0.598	0.628
NM_025381	49	64	46	40	Sdf2	chr1	1300	78068469	0.947901	0.562	0.535
NM_133807	40	31	56	9	Nf1	chr1	11847	79392761	0.144447	0.606	0.862
NM_028659	34	34	13	20	Lig3	chr1	5945	82615342	0.947901	0.438	0.394
NM_019774	30	32	22	30	Ap2b1	chr1	5411	83183224	0.721145	0.554	0.423
NM_027231	115	89	28	15	Tada2l	chr1	2119	83923509	0.160657	0.418	0.651
NM_026498	81	42	17	21	Acaca	chr1	9054	84213407	0.947901	0.5	0.447
NM_011350	16	15	19	26	Znhit3	chr1	983	84727762	0.243569	0.652	0.422
NM_010418	20	24	26	30	Tmem49	chr1	2773	86457063	0.853013	0.593	0.464
NM_178705	19	23	50	54	Cltc	chr1	6178	86570753	0.947901	0.512	0.481
NM_019675	112	92	66	70	Gdpd1	chr1	2326	86887360	0.657386	0.598	0.485
NM_199011	10	16	162	85	Trim37	chr1	5649	87031855	0.523991	0.565	0.656
NM_016738	48	30	55	70	Ppm1e	chr1	6315	87057798	0.525209	0.581	0.44
NM_026420	282	248	17	30	Sfrs1	chr1	5066	87865975	0.724838	0.533	0.362
NM_007604	42	39	18	18	Mrps23	chr1	1302	88023623	0.947901	0.52	0.5
NM_007743	20	26	232	171	Nme1	chr1	1235	93829589	0.915297	0.53	0.576
NM_025338	122	113	47	68	Lrrc59	chr1	2810	94504612	0.459791	0.563	0.409
NM_018753	150	58	308	426	Atp5g1	chr1	4675	95936305	0.805861	0.457	0.42
NM_019640	32	46	58	58	Kpnb1	chr1	5909	97041884	0.947901	0.474	0.5
NM_025816	106	52	22	21	Lasp1	chr1	3433	97698195	0.947901	0.588	0.512
NM_133740	15	11	28	6	Igfbp4	chr1	2069	98913100	0.222809	0.517	0.824
NM_028020	19	16	43	12	Smarce1	chr1	2960	99091724	0.947901	0.717	0.782

NM_133797	10	17	63	34	Hap1	chr1	3651	100215644	0.947901	0.571	0.649
NM_018814	59	19	54	33	Cnp	chr1	2283	100441903	0.947901	0.667	0.621
NM_146121	20	15	16	15	Nkiras2	chr1	2013	100487358	0.947901	0.483	0.516
NM_021608	123	118	19	19	Tubg1	chr1	1609	100987411	0.665043	0.333	0.5
NM_201638	46	29	89	60	Rpl27	chr1	536	101304056	0.945732	0.556	0.597
NM_178709	28	28	124	100	Nbr1	chr1	4444	101441923	0.677707	0.473	0.554
NM_145380	61	45	72	67	Mpp3	chr1	2860	101886463	0.780488	0.434	0.518
NM_011434	229	52	44	18	Eftud2	chr1	3397	102709280	0.947901	0.645	0.71
NM_008943	146	97	10	17	Nmt1	chr1	1838	102927122	0.677707	0.586	0.37
NM_021316	23	25	41	34	Gosr2	chr1	3082	103558979	0.947901	0.61	0.547
NM_172892	23	5	19	14	Nsf	chr1	3766	103744107	0.947901	0.654	0.576
NM_026828	29	12	51	29	Mapt	chr1	5213	104189417	0.395208	0.791	0.638
NM_080640	30	16	18	11	Cyb561	chr1	2474	105795041	0.947901	0.519	0.621
NM_008229	21	21	58	49	Wdr68	chr1	5765	105919448	0.947901	0.584	0.542
NM_010443	12	18	30	23	Ccdc47	chr1	3270	106077483	0.947901	0.622	0.566
NM_145760	16	11	56	30	Psmc5	chr1	1277	106122967	0.947901	0.661	0.651
NM_027098	214	106	37	34	Psmd12	chr1	1723	107359320	0.80737	0.655	0.521
NM_053197	27	23	90	50	Prkar1a	chr1	3323	109524418	0.805861	0.566	0.643
NM_001024853	23	20	47	23	Cog1	chr1	3060	113522262	0.311611	0.4	0.671
NM_197987	105	81	52	38	D11Wsu99e	chr1	2635	113522791	0.857111	0.475	0.578
NM_008946	1040	947	34	110	Ttyh2	chr1	3456	114581941	0.677707	0.356	0.236
NM_010269	16	22	29	18	Slc9a3r1	chr1	1922	115042086	0.947901	0.578	0.617
NM_145142	114	131	47	14	C630004H02Rik	chr1	3237	115222986	0.947901	0.83	0.77
NM_017393	41	49	39	11	Acox1	chr1	4065	116060090	0.379948	0.56	0.78
NM_001081249	19	10	26	10	Srp68	chr1	2546	116126782	0.947901	0.655	0.722
NM_008634	101	64	42	11	Exoc7	chr1	2094	116166339	0.710344	0.649	0.792
NM_024189	25	48	31	32	Birc5	chr1	977	117716699	0.947901	0.516	0.492
NM_029711	99	89	36	38	Eif4a3	chr1	1489	119161316	0.909313	0.413	0.486
NM_018757	94	30	55	73	Mrpl12	chr1	963	120349497	0.807174	0.526	0.43
NM_080635	31	19	19	18	Anapc11	chr1	3213	120466849	0.931904	0.412	0.514
NM_009679	34	19	16	16	Foxk2	chr1	5362	121168170	0.947901	0.462	0.5
NM_134115	31	21	123	97	Dnmt3a	chr1	5424	3911461	0.947901	0.555	0.559
NM_025378	38	25	118	129	C030014M07Rik	chr1	6547	3999711	0.947901	0.461	0.478
NM_177474	32	12	7	19	C330021A05Rik	chr1	4695	4103107	0.811974	0.429	0.269
NM_022992	20	24	15	36	1110057K04Rik	chr1	2714	8234045	0.246799	0.532	0.294
NM_178600	56	33	86	88	Laptm4a	chr1	2130	8945413	0.805861	0.605	0.494
NM_018829	19	12	75	125	Vsnl1	chr1	1889	11338831	0.947901	0.438	0.375
NM_001025264	27	27	123	86	D12Ertd553e	chr1	4781	12380934	0.783653	0.523	0.589
NM_175102	77	38	34	32	Ddx1	chr1	2488	13251777	0.947901	0.526	0.515
NM_009242	148	103	63	29	AW125753	chr1	3402	14154510	0.947901	0.607	0.685
NM_007907	394	322	42	22	Pdia6	chr1	2125	17290792	0.86501	0.558	0.656
NM_030743	92	74	29	30	Itgb1bp1	chr1	1874	21292007	0.947901	0.519	0.492
NM_026812	81	65	54	62	Iah1	chr1	941	21329257	0.947901	0.447	0.466
NM_001081210	21	7	20	27	Adam17	chr1	4410	21379212	0.947901	0.5	0.426
NM_029810	36	18	199	218	C330002I19Rik	chr1	7402	25701798	0.947901	0.451	0.477
NM_134024	20	40	106	157	Rps7	chr1	682	29320305	0.947901	0.404	0.403
NM_030886	26	19	15	19	Ttc15	chr1	2966	29435153	0.947901	0.5	0.441
NM_011520	51	43	19	22	Tssc1	chr1	1683	29436741	0.92262	0.561	0.463
NM_134084	56	22	1236	1382	Dld	chr1	2258	32034478	0.947901	0.452	0.472
NM_146141	84	65	26	16	Pbef1	chr1	2664	33536116	0.947901	0.695	0.619
NM_011890	12	15	430	329	Ankmy2	chr1	2380	36903189	0.947901	0.559	0.567
NM_001038613	49	54	29	23	Etv1	chr1	4067	39592392	0.947901	0.625	0.558

NM_009608	0	46	25	26	Lrrn3	chr1	3238	42178370	0.853013	0.584	0.49
NM_144512	34	12	181	97	Nrcam	chr1	4484	45488926	0.601938	0.5	0.651
NM_016792	17	15	50	25	Akap6	chr1	10263	53896849	0.947901	0.686	0.667
NM_031869	21	15	138	98	1110002B05Rik	chr1	1363	55746554	0.509331	0.727	0.585
NM_026082	26	21	99	265	Cfl2	chr1	2940	55959937	0.844976	0.364	0.272
NM_017392	19	14	24	10	Brms1l	chr1	2566	56969415	0.941581	0.6	0.706
NM_175172	64	68	36	43	Trappc6b	chr1	1272	60149122	0.853013	0.532	0.456
NM_054093	45	21	69	84	Fkbp3	chr1	945	66170028	0.947901	0.47	0.451
NM_025638	52	35	187	175	Rps29	chr1	271	70258758	0.947901	0.52	0.517
NM_001024508	69	85	37	18	Klhdc2	chr1	1637	70410995	0.419101	0.438	0.673
NM_178602	32	28	36	32	Sdccag1	chr1	3720	70421401	0.947901	0.488	0.529
NM_025455	481	408	1	31	Pygl	chr1	2780	71323830	0.941158	0.075	0.031
NM_172468	38	29	42	45	Psma3	chr1	1348	72085560	0.947901	0.463	0.483
NM_010240	301	96	37	13	Timm9	chr1	997	72237606	0.459791	0.535	0.74
NM_025868	47	43	69	36	Daam1	chr1	6004	72996819	0.783653	0.552	0.657
NM_008112	52	25	39	52	Ppm1a	chr1	2033	73895240	0.947901	0.481	0.429
NM_025602	18	21	73	77	Syt16	chr1	2705	75367764	0.947901	0.514	0.487
NM_177763	90	61	130	101	Atp6v1d	chr1	1410	79944043	0.853013	0.494	0.563
NM_009786	90	74	51	38	6330442E10Rik	chr1	3880	80078765	0.947901	0.604	0.573
NM_011341	35	19	67	40	Pcnx	chr1	12139	83096092	0.540466	0.756	0.626
NM_001081324	103	84	43	47	Psen1	chr1	3023	85075188	0.479272	0.601	0.478
NM_026329	1610	1483	47	34	Npc2	chr1	3278	86113860	0.947901	0.61	0.58
NM_016669	38	28	56	39	Fcf1	chr1	730	86321471	0.427963	0.445	0.589
NM_010329	43	48	32	12	Dlst	chr1	2726	86473724	0.945228	0.66	0.727
NM_078479	34	49	18	9	Nek9	chr1	5403	86649812	0.805861	0.481	0.667
NM_029632	20	21	18	15	0610007P14Rik	chr1	1185	87163120	0.947901	0.485	0.545
NM_009722	24	22	42	47	1700019E19Rik	chr1	773	87423580	0.947901	0.468	0.472
NM_133673	26	25	12	22	Gstz1	chr1	1656	88505234	0.710344	0.216	0.353
NM_007386	32	12	28	30	Ahsa1	chr1	1330	88614134	0.947901	0.463	0.483
NM_008047	54	41	40	18	Adck1	chr1	2287	89699661	0.459791	0.489	0.69
NM_027432	99	40	91	62	Sel1l	chr1	6300	93080100	0.947901	0.598	0.595
NM_018749	102	88	45	41	Zc3h14	chr1	3557	100025339	0.877763	0.444	0.523
NM_008304	15	18	54	50	Eml5	chr1	10136	100125219	0.947901	0.528	0.519
NM_177561	14	11	52	38	Psmc1	chr1	1502	101361279	0.947901	0.569	0.578
NM_178626	37	43	54	25	Cpsf2	chr1	4840	103226871	0.947901	0.659	0.684
NM_026644	29	45	106	70	Ddx24	chr1	3128	104663855	0.18768	0.465	0.602
NM_145919	25	20	38	51	Dicer1	chr1	9851	105969324	0.947901	0.367	0.427
NM_028343	21	19	26	33	2410024A21Rik	chr1	10173	106907477	0.945732	0.529	0.441
NM_177356	29	10	20	19	4933433P14Rik	chr1	2787	106939081	0.92262	0.406	0.513
NM_023908	19	15	43	28	Papola	chr1	4518	107075446	0.624131	0.444	0.606
NM_019552	19	9	119	104	Ppp2r5c	chr1	2377	111809260	0.801072	0.597	0.534
NM_078478	20	29	252	172	Dync1h1	chr1	14398	111903364	0.947901	0.588	0.594
NM_008820	19	11	18	17	Bag5	chr1	1800	112948258	0.947901	0.591	0.514
NM_011112	20	25	47	19	2810002N01Rik	chr1	1026	112971881	0.947901	0.765	0.712
NM_009365	34	59	55	42	Klc1	chr1	2398	113032656	0.696682	0.466	0.567
NM_007495	61	73	36	63	Ppp1r13b	chr1	4355	113104630	0.381503	0.511	0.364
NM_008729	38	33	35	31	Tmem179	chr1	2373	113738969	0.86501	0.429	0.53
NM_001081344	11	17	40	49	AW555464	chr1	6640	113981946	0.947901	0.395	0.449
NM_008676	78	87	107	82	Gdi2	chr1	2681	3564670	0.653115	0.675	0.566
NM_016857	24	13	53	45	Pitrm1	chr1	3547	6578692	0.947901	0.487	0.541
NM_024465	29	136	205	179	Pfkp	chr1	3986	6647955	0.947901	0.556	0.534
NM_001004363	19	22	28	14	Gng4	chr1	2976	13919840	0.947901	0.719	0.667

NM_027296	18	27	21	31	Stard3nl	chr1	1301	19468454	0.947901	0.345	0.404
NM_025471	14	12	24	32	Epdr1	chr1	2362	19686355	0.255447	0.682	0.429
NM_007478	69	55	65	99	Them2	chr1	686	24923211	0.118088	0.579	0.396
NM_023211	184	242	81	46	Nrsn1	chr1	2103	25354226	0.805861	0.541	0.638
NM_018769	15	10	81	18	Tubb2a	chr1	1615	34166215	0.947901	0.829	0.818
NM_011660	15	22	499	206	Tubb2b	chr1	1922	34218890	0.947901	0.703	0.708
NM_011185	202	200	18	70	Riok1	chr1	2435	38152800	0.677707	0.322	0.205
NM_025409	18	21	33	42	Txndc5	chr1	2639	38598824	0.947901	0.449	0.44
NM_028392	28	35	49	34	Phactr1	chr1	3849	43230838	0.947901	0.6	0.59
NM_011694	58	52	79	53	Spin1	chr1	4136	51245265	0.945732	0.655	0.598
NM_019982	89	81	28	23	Auh	chr1	1345	52936267	0.687044	0.36	0.549
NM_025826	18	21	12	13	Nfil3	chr1	2019	53062818	0.947901	0.556	0.48
NM_021881	20	9	184	165	Sfxn1	chr1	2795	54201986	0.947901	0.54	0.527
NM_007771	14	20	88	116	Cplx2	chr1	4925	54484462	0.431606	0.55	0.431
NM_054046	37	38	66	37	4732471D19Rik	chr1	3244	54652041	0.927748	0.552	0.641
NM_027398	72	49	74	57	4833439L19Rik	chr1	2624	54653819	0.562014	0.37	0.565
NM_053273	16	29	16	24	D13Wsu177e	chr1	1734	54691028	0.784904	0.523	0.4
NM_026246	15	22	78	56	Higd2a	chr1	613	54691609	0.86501	0.525	0.582
NM_177235	39	35	19	22	Tmed9	chr1	1463	55698265	0.947901	0.497	0.463
NM_177214	24	35	80	60	Caml	chr1	1387	55733208	0.947901	0.636	0.571
NM_133973	308	303	7	18	H2afy	chr1	1403	56197530	0.26307	0.621	0.28
NM_175185	95	91	32	26	Smad5	chr1	6504	56842361	0.947901	0.553	0.552
NM_024242	19	40	26	48	Ubqln1	chr1	2399	58284546	0.677707	0.454	0.351
NM_021028	13	14	43	22	Ntrk2	chr1	7022	59036573	0.945732	0.581	0.662
NM_134082	40	27	33	56	Isca1	chr1	1915	59857211	0.525209	0.511	0.371
NM_198306	20	47	20	7	Dapk1	chr1	5903	60826720	0.947901	0.727	0.741
NM_013648	23	9	14	16	Ctsl	chr1	1971	64470357	0.947901	0.419	0.467
NM_023402	298	248	14	32	Brd9	chr1	2494	74097925	0.602323	0.448	0.304
NM_010363	16	58	196	133	Sdha	chr1	2859	74482356	0.947901	0.581	0.596
NM_019735	85	59	23	15	Gpr98	chr1	19328	81772010	0.261477	0.875	0.605
NM_008450	69	79	201	154	Cetn3	chr1	1013	81935837	0.947901	0.525	0.566
NM_008996	39	17	15	26	Mef2c	chr1	2430	83659478	0.807174	0.519	0.366
NM_013685	33	41	39	39	Ccnh	chr1	1976	85352386	0.706186	0.375	0.5
NM_027226	33	28	13	12	Rasa1	chr1	5101	85390066	0.947901	0.579	0.52
NM_026842	119	143	926	1098	Cox7c	chr1	449	86184479	0.805861	0.385	0.458
NM_026533	63	35	120	89	Edil3	chr1	5328	89459293	0.947901	0.573	0.574
NM_019880	87	59	16	21	Vcan	chr1	12432	89842842	0.593057	0.655	0.432
NM_029635	242	172	29	35	Tmem167	chr1	3144	90244666	0.947901	0.467	0.453
NM_133718	18	11	108	80	Ssbp2	chr1	3866	91840309	0.947901	0.605	0.574
NM_201226	25	24	14	13	Zfyve16	chr1	5451	93291202	0.947901	0.577	0.519
NM_008707	17	12	17	18	Lhfpl2	chr1	4348	94962697	0.673494	0.692	0.486
NM_172860	33	15	17	9	Ap3b1	chr1	4020	95301986	0.947901	0.577	0.654
NM_022319	73	59	19	11	Wdr41	chr1	3063	95791753	0.945732	0.531	0.633
NM_153064	98	96	18	21	Aggf1	chr1	3213	96140733	0.812477	0.615	0.462
NM_023243	27	45	114	86	Hmgcr	chr1	4444	97436099	0.947901	0.528	0.57
NM_028190	18	11	42	18	Hexb	chr1	1750	97968061	0.838923	0.583	0.7
NM_025516	86	102	196	232	Enc1	chr1	4695	98020510	0.947901	0.434	0.458
NM_001003971	21	17	13	14	Mrps27	chr1	1849	100114821	0.947901	0.393	0.481
NM_007615	20	11	16	21	Mtap1b	chr1	8818	100278136	0.487509	0.612	0.432
NM_025511	70	73	13	17	Pik3r1	chr1	6915	102527846	0.947901	0.394	0.433
NM_024432	101	85	37	43	Sfrs12	chr1	4063	104550669	0.866345	0.373	0.463
NM_023514	33	31	19	25	Erbb2ip	chr1	6602	104679333	0.562731	0.636	0.432

NM_172589	18	8	54	39	Nln	chr1	3780	104862854	0.232461	0.727	0.581
NM_013678	23	20	17	14	Ipo11	chr1	4282	107715138	0.947901	0.556	0.548
NM_016709	9	16	25	14	Dimt1	chr1	2038	107747177	0.866442	0.517	0.641
NM_026508	16	14	32	13	Gpbp1	chr1	3451	112228252	0.749267	0.837	0.711
NM_175036	49	25	27	24	Snag1	chr1	4443	114382456	0.947901	0.464	0.529
NM_025424	16	27	193	343	Ndufs4	chr1	656	115178192	0.252235	0.506	0.36
NM_145457	9	16	43	36	Paip1	chr1	4668	120246571	0.677707	0.36	0.544
NM_010097	58	60	49	22	4833420G17Rik	chr1	2637	120274565	0.947901	0.66	0.69
NM_173374	16	14	56	36	Pdhb	chr1	1485	9005290	0.405087	0.44	0.609
NM_009030	17	9	68	47	Cadps	chr1	5475	13383370	0.858255	0.508	0.591
NM_177718	65	59	99	61	Synpr	chr1	2541	14446849	0.947901	0.559	0.619
NM_182841	26	14	51	42	Thoc7	chr1	883	14783737	0.947901	0.552	0.548
NM_001035854	46	37	54	135	Psmd6	chr1	1313	14944742	0.108104	0.524	0.286
NM_011490	38	31	14	17	Slc4a7	chr1	7389	15628676	0.947901	0.56	0.452
NM_010266	27	22	87	87	Top2b	chr1	5652	17262893	0.947901	0.549	0.5
NM_023374	384	309	62	37	Nkiras1	chr1	4592	19116297	0.947901	0.608	0.626
NM_026950	38	45	24	12	Ube2e2	chr1	1410	19406582	0.947901	0.662	0.667
NM_009696	31	29	46	57	Ap3m1	chr1	3099	21854088	0.673494	0.613	0.447
NM_175451	49	20	10	15	Polr3a	chr1	4683	25284571	0.947901	0.448	0.4
NM_011969	552	471	43	30	Ppif	chr1	1560	26517801	0.641958	0.718	0.589
NM_028487	36	7	42	35	Appl1	chr1	6961	27771669	0.947901	0.532	0.545
NM_009838	221	158	20	24	Erc2	chr1	6083	28590239	0.823948	0.588	0.455
NM_172396	36	85	84	61	Selk	chr1	1018	30786565	0.805861	0.518	0.579
NM_181405	8	17	21	19	Cacna1d	chr1	8705	31304189	0.870979	0.417	0.525
NM_027539	43	40	52	33	Glt8d1	chr1	1758	31824693	0.947901	0.609	0.612
NM_025855	18	14	65	20	Pbrm1	chr1	8117	31933736	0.370167	0.616	0.765
NM_026784	177	118	17	27	Nt5dc2	chr1	1435	31952126	0.100058	0.677	0.386
NM_152812	16	21	23	28	Stab1	chr1	7995	31981608	0.828498	0.575	0.451
NM_030561	12	22	99	54	Sh3bp5	chr1	2487	32200914	0.770715	0.553	0.647
NM_172525	18	8	31	34	Sncg	chr1	726	35186537	0.947901	0.556	0.477
NM_023565	53	57	45	36	Pcdh21	chr1	4364	37911317	0.933287	0.645	0.556
NM_009007	157	142	40	31	Ghitm	chr1	2476	37946866	0.646096	0.408	0.563
NM_011278	39	37	41	21	5730469M10Rik	chr1	1537	41827047	0.805861	0.52	0.661
NM_026792	18	13	91	83	Ctsb	chr1	4738	63741362	0.947901	0.493	0.523
NM_021884	28	16	138	106	Elp3	chr1	2722	66205113	0.37945	0.685	0.566
NM_027166	52	52	33	27	Clu	chr1	1669	66600370	0.947901	0.594	0.55
NM_025944	28	26	67	55	Trim35	chr1	3684	66928629	0.524573	0.661	0.549
NM_027188	25	48	57	27	Stmn4	chr1	1249	66980342	0.473107	0.549	0.679
NM_016683	25	24	52	64	Pnma2	chr1	4365	67538491	0.947901	0.474	0.448
NM_025327	72	62	16	16	Bnip3l	chr1	3237	67604148	0.947901	0.471	0.5
NM_024472	16	20	11	17	Nefl	chr1	2014	68705532	0.943216	0.5	0.393
NM_024236	28	35	50	53	Nefm	chr1	3244	68738571	0.947901	0.446	0.485
NM_001080128	57	48	25	16	Xpo7	chr1	15118	71103060	0.947901	0.677	0.61
NM_146169	16	16	466	337	Itm2b	chr1	1773	73768222	0.947901	0.557	0.58
NM_133942	14	12	37	38	Med4	chr1	1304	73918252	0.833193	0.593	0.493
NM_172963	43	7	85	67	Sucla2	chr1	2108	73995309	0.947901	0.551	0.559
NM_009656	30	15	139	136	Esd	chr1	1618	75149567	0.947901	0.522	0.505
NM_013813	57	40	41	27	Cldn10	chr1	1903	119272919	0.824752	0.5	0.603
NM_009691	62	54	20	25	Farp1	chr1	4857	121681742	0.677707	0.597	0.444
NM_001038587	34	40	66	32	Dock9	chr1	7910	122097626	0.817803	0.579	0.673
NM_173876	20	11	42	23	Fgf14	chr1	2831	124592006	0.947901	0.595	0.646
NM_175095	67	42	141	137	Sepp1	chr1	2030	3229130	0.947901	0.53	0.507

NM_008142	105	74	82	118	Rpl37	chr1	835	5068591	0.066417	0.561	0.41
NM_009199	37	20	477	266	Slc1a3	chr1	4147	8638333	0.37945	0.547	0.642
NM_025665	21	24	31	31	Bxdc2	chr1	2654	10415550	0.947901	0.478	0.5
NM_027530	101	94	111	66	Sub1	chr1	3321	11912621	0.199722	0.493	0.627
NM_001009818	162	211	31	37	Rnasen	chr1	4185	12864879	0.947901	0.5	0.456
NM_028814	31	14	85	44	Ctnnd2	chr1	5959	30599336	0.611248	0.535	0.659
NM_028521	22	18	128	118	Mar6	chr1	6236	31408122	0.935791	0.444	0.52
NM_025283	59	41	29	16	Sdc2	chr1	3313	32962720	0.646096	0.455	0.644
NM_009128	241	236	361	616	Cox6c	chr1	527	35867036	0.392046	0.508	0.369
NM_133900	18	16	266	228	Polr2k	chr1	619	36106551	0.405087	0.648	0.538
NM_020582	625	662	222	267	Zfp706	chr1	4485	36928594	0.947901	0.422	0.454
NM_177784	36	34	120	130	Ncald	chr1	3708	37301974	0.947901	0.503	0.48
NM_001001986	9	25	61	35	Azin1	chr1	4868	38430125	0.947901	0.634	0.635
NM_053078	105	98	16	20	Baalc	chr1	2524	38780711	0.543527	0.652	0.444
NM_170669	47	39	52	41	Eif3s6	chr1	1541	43114203	0.947659	0.495	0.559
NM_007863	53	69	19	14	5730410E15Rik	chr1	2948	44509092	0.947901	0.514	0.576
NM_011894	42	34	71	82	Eif3s3	chr1	1254	51696911	0.525209	0.62	0.464
NM_178605	58	53	87	65	Rad21	chr1	3405	51814081	0.947901	0.561	0.572
NM_011722	67	44	65	54	Enpp2	chr1	3242	54751523	0.947901	0.6	0.546
NM_026122	18	17	61	49	Mrpl13	chr1	786	55388852	0.947901	0.557	0.555
NM_009273	18	21	59	35	Tmem65	chr1	3644	58620955	0.947901	0.582	0.628
NM_019748	126	111	134	135	Ndufb9	chr1	651	58765388	0.339976	0.58	0.498
NM_153535	16	22	59	72	Sqle	chr1	2748	59162397	0.783653	0.537	0.45
NM_182807	60	52	80	47	C920006C10Rik	chr1	5210	65702776	0.947901	0.674	0.63
NM_031185	27	24	131	119	Vps28	chr1	885	76452699	0.947901	0.494	0.524
NM_019716	60	52	131	58	Txn2	chr1	1282	77755066	0.065669	0.551	0.693
NM_001009948	33	49	85	105	Eif3s7	chr1	1910	77792317	0.611248	0.537	0.447
NM_009270	58	50	43	25	Pdxp	chr1	2018	78749729	0.947901	0.68	0.632
NM_176832	20	26	33	25	Nol12	chr1	1993	78771594	0.947901	0.602	0.569
NM_026102	32	26	70	51	Eif3s6ip	chr1	1893	78920100	0.947901	0.542	0.579
NM_001033354	12	20	125	63	Polr2f	chr1	612	78982022	0.459791	0.564	0.665
NM_007638	45	48	49	67	Csnk1e	chr1	2760	79256732	0.846223	0.495	0.422
NM_010305	38	41	41	75	Slc25a17	chr1	1667	81159481	0.863684	0.44	0.353
NM_009449	25	1	25	30	Phf5a	chr1	1648	81695700	0.947901	0.459	0.455
NM_172633	46	26	27	22	Aco2	chr1	2785	81745078	0.947901	0.51	0.551
NM_033560	27	26	60	44	Pmm1	chr1	1228	81788293	0.947901	0.622	0.577
NM_027481	9	25	13	13	Nhp2l1	chr1	1257	81877989	0.947901	0.484	0.5
NM_019806	9	26	81	75	Sep3	chr1	4731	82122589	0.947901	0.532	0.519
NM_001033136	12	18	72	63	1500032L24Rik	chr1	608	82179305	0.947901	0.588	0.533
NM_025606	23	17	48	28	Ndufa6	chr1	485	82180700	0.947901	0.6	0.632
NM_010514	52	43	25	20	Mcat	chr1	1893	83379686	0.873506	0.68	0.556
NM_011936	18	14	83	53	Mpped1	chr1	3255	83688445	0.947901	0.604	0.61
NM_010209	29	26	31	28	Sult4a1	chr1	2446	83920396	0.947901	0.455	0.525
NM_028146	56	28	45	24	Ldoc1l	chr1	4447	84383942	0.410704	0.493	0.652
NM_007803	70	56	84	98	Atxn10	chr1	2425	85292811	0.947901	0.457	0.462
NM_008227	24	7	52	55	Yaf2	chr1	2192	93114504	0.525209	0.342	0.486
NM_207225	51	25	19	23	Twf1	chr1	2973	94416918	0.250288	0.717	0.452
NM_010863	19	12	62	67	Nell2	chr1	3385	95177209	0.947901	0.456	0.481
NM_029758	103	94	27	32	Cacnb3	chr1	2837	98474040	0.947901	0.491	0.458
NM_009622	103	96	77	40	Arf3	chr1	3481	98569203	0.673494	0.556	0.658
NM_021604	149	133	302	227	Tuba1a	chr1	1591	98780977	0.066417	0.652	0.571
NM_009095	240	323	59	57	Tegt	chr1	2340	99238939	0.947901	0.543	0.509

NM_144522	13	16	20	15	Zfp740	chr1	3760	102043161	0.947901	0.6	0.571
NM_023173	22	6	37	56	Map3k12	chr1	5158	102328421	0.947901	0.464	0.398
NM_025898	24	31	72	90	Atp5g2	chr1	647	102495502	0.900976	0.5	0.444
NM_007834	46	29	20	7	Trap1	chr1	2297	4045453	0.677707	0.533	0.741
NM_053084	65	56	13	17	Coro7	chr1	3518	4679607	0.947901	0.333	0.433
NM_026558	41	14	17	9	Hmox2	chr1	1255	4765850	0.540466	0.4	0.654
NM_010324	28	18	65	40	5730403B10Rik	chr1	3898	4789870	0.863684	0.544	0.619
NM_027204	30	27	22	21	Mgrn1	chr1	3138	4937365	0.947901	0.531	0.512
NM_031179	27	14	117	135	BC024814	chr1	1347	8490209	0.947901	0.452	0.464
NM_054102	17	29	26	26	Abat	chr1	4593	8620756	0.86501	0.408	0.5
NM_029721	150	136	39	37	Carhsp1	chr1	2849	8663733	0.947901	0.473	0.513
NM_001039522	28	12	31	58	Nubp1	chr1	1276	10420363	0.139629	0.571	0.348
NM_001081458	114	77	47	81	BC068110	chr1	3321	10447095	0.189309	0.542	0.367
NM_010233	24	17	169	126	Snn	chr1	2919	11072688	0.947901	0.573	0.573
NM_007488	135	222	54	13	Rsl1d1	chr1	1839	11201396	0.347816	0.642	0.806
NM_130884	113	151	23	18	Parn	chr1	2902	13666013	0.863684	0.438	0.561
NM_028782	30	35	40	2	Mpv17l	chr1	3056	13948791	0.883593	0.889	0.952
NM_023153	16	17	58	20	2900011O08Rik	chr1	2355	14100703	0.358439	0.549	0.744
NM_144806	142	146	66	47	Ube2v2	chr1	4179	15554767	0.947901	0.523	0.584
NM_025849	15	21	13	80	2410018G20Rik	chr1	1010	15862547	0.179836	0.319	0.14
NM_175145	29	25	43	47	Dnm1l	chr1	4039	16346167	0.947901	0.5	0.478
NM_016802	15	14	41	35	Ypel1	chr1	2659	17084820	0.947901	0.517	0.539
NM_001081306	23	20	26	27	Pcqap	chr1	3383	17651422	0.945732	0.593	0.491
NM_008492	268	173	27	19	Dgcr6	chr1	1262	18071178	0.947901	0.559	0.587
NM_146012	10	15	25	19	D16H22S680E	chr1	1500	18310970	0.947901	0.512	0.568
NM_007749	30	48	21	17	Lamp3	chr1	3343	19706418	0.611248	0.744	0.553
NM_019979	187	174	26	28	B3gnt5	chr1	4098	19765204	0.947901	0.538	0.481
NM_026391	53	43	46	71	Klhl24	chr1	6683	20124587	0.933057	0.483	0.393
NM_001077507	181	214	27	37	Eif2b5	chr1	2579	20509255	0.833298	0.543	0.422
NM_007520	14	14	24	29	Ap2m1	chr1	2057	20541919	0.555395	0.642	0.453
NM_009003	33	31	25	46	2510009E07Rik	chr1	5130	21650854	0.947901	0.347	0.352
NM_138590	44	50	23	33	Senp2	chr1	3109	22046915	0.947901	0.44	0.411
NM_053092	29	17	26	16	Sst	chr1	599	23890860	0.947901	0.605	0.619
NM_183178	32	28	38	22	1600021P15Rik	chr1	3080	28826485	0.753949	0.524	0.633
NM_008640	23	15	29	39	Tmem44	chr1	4241	30547501	0.876897	0.505	0.426
NM_011188	51	34	22	10	Pak2	chr1	4135	32052464	0.945732	0.586	0.688
NM_027722	39	25	24	10	Fbxo45	chr1	4110	32238648	0.915297	0.605	0.706
NM_009513	33	28	31	25	Tfrc	chr1	4838	32630890	0.906185	0.65	0.554
NM_007983	18	15	68	35	Fyttd1	chr1	4255	32906331	0.710344	0.541	0.66
NM_153410	29	18	66	64	Snx4	chr1	2442	33298656	0.947901	0.53	0.508
NM_021880	69	53	17	22	Itgb5	chr1	2981	33899464	0.945732	0.348	0.436
NM_172652	58	30	17	16	Sec22a	chr1	3992	35363943	0.947901	0.48	0.515
NM_183270	23	21	90	42	Fstl1	chr1	3609	37834047	0.602323	0.568	0.682
NM_025282	14	13	31	51	Lrrc58	chr1	8592	37886860	0.947901	0.442	0.378
NM_001081394	9	20	79	13	Gsk3b	chr1	8298	38243868	0.265596	0.685	0.859
NM_029004	69	72	139	127	Adprh	chr1	1583	38450274	0.947901	0.51	0.523
NM_024178	49	61	23	19	Ktelc1	chr1	2641	38529573	0.947901	0.614	0.548
NM_026352	72	58	171	173	Atp6v1a	chr1	3946	44114731	0.923252	0.545	0.497
NM_010129	28	16	100	46	Senp7	chr1	4802	56188519	0.706466	0.553	0.685
NM_028018	48	35	16	20	Tbc1d23	chr1	3664	57214376	0.450421	0.686	0.444
NM_001083807	15	16	17	16	Arl6	chr1	1326	59638805	0.947901	0.6	0.515
NM_029406	24	34	19	6	Pros1	chr1	3270	62927887	0.92262	0.853	0.76

NM_027815	46	25	90	69	Chmp2b	chr1	1838	65562727	0.550672	0.45	0.566
NM_001025438	25	11	177	88	Robo1	chr1	7568	73043375	0.352547	0.526	0.668
NM_178939	114	62	31	17	Robo2	chr1	8047	74035238	0.947901	0.655	0.646
NM_080560	38	33	469	379	Cxadr	chr1	5538	78338878	0.947901	0.537	0.553
NM_009076	43	7	138	145	App	chr1	3140	85120940	0.947901	0.482	0.488
NM_027464	13	12	21	30	Usp16	chr1	2862	87483225	0.523991	0.2	0.412
NM_145138	13	14	161	122	Cct8	chr1	2391	87483797	0.891737	0.519	0.569
NM_008054	38	39	32	56	Bach1	chr1	5858	87732866	0.805861	0.5	0.364
NM_007506	212	252	81	30	Sod1	chr1	661	90224623	0.552476	0.815	0.73
NM_025835	39	15	99	74	Tmem50b	chr1	2237	91580487	0.947901	0.537	0.572
NM_018760	34	33	53	57	Son	chr1	8451	91677684	0.296511	0.698	0.482
NM_016908	21	12	39	16	Cbr1	chr1	1081	93608095	0.947901	0.696	0.709
NM_009260	29	35	305	145	Ttc3	chr1	7295	94645774	0.139629	0.575	0.678
NM_010288	28	26	24	24	Dscr3	chr1	2419	94748021	0.805861	0.613	0.5
NM_025894	19	10	76	28	Wrb	chr1	2483	96378872	0.947901	0.688	0.731
NM_138656	20	28	207	212	Pcp4	chr1	669	96747211	0.352547	0.636	0.494
NM_025304	45	48	16	42	5730437N04Rik	chr1	971	5439601	0.947901	0.308	0.276
NM_011879	25	18	64	56	Qk	chr1	6887	10407408	0.710344	0.69	0.533
NM_010411	28	16	56	51	Agpat4	chr1	1910	12412331	0.602323	0.392	0.523
NM_011287	77	70	29	27	4932442K08Rik	chr1	4540	15569914	0.947901	0.6	0.518
NM_030147	19	15	283	227	Psmb1	chr1	1036	15635197	0.677707	0.502	0.555
NM_019641	28691	18755	22	22	Ppp2r1a	chr1	2256	21097450	0.947901	0.544	0.5
NM_016909	51	23	56	57	Abca3	chr1	6453	24544598	0.947901	0.484	0.496
NM_145420	26	6	70	40	Rnps1	chr1	1631	24562481	0.753949	0.543	0.636
NM_001081235	38	33	12	16	Tsc2	chr1	6147	24765981	0.947901	0.536	0.429
NM_172561	14	25	8	17	Slc9a3r2	chr1	2085	24778788	0.947901	0.423	0.32
NM_145518	58	49	41	22	Syngr3	chr1	1959	24822274	0.947901	0.577	0.651
NM_178051	28	15	264	326	Ndufb10	chr1	657	24859107	0.381503	0.339	0.447
NM_153082	12	16	18	10	Hagh	chr1	1148	25001238	0.157861	0.281	0.643
NM_010325	39	39	21	33	Gnptg	chr1	1234	25371429	0.947901	0.423	0.389
NM_025630	16	10	12	169	0610007P22Rik	chr1	1160	25379141	0.194015	0.172	0.066
NM_001080747	37	38	24	15	Itfg3	chr1	2629	26357314	0.947901	0.631	0.615
NM_145564	35	25	19	25	Luc7l	chr1	4993	26419045	0.684867	0.621	0.432
NM_010063	131	163	47	48	Ergic1	chr1	2734	26792165	0.947901	0.529	0.495
NM_007688	12	21	38	20	Atp6v0e	chr1	800	26836191	0.947901	0.596	0.655
NM_018744	16	24	31	33	Nudt3	chr1	2171	27717065	0.947901	0.515	0.484
NM_029432	46	28	213	155	Rpl10a	chr1	700	28466442	0.841387	0.524	0.579
NM_001083321	21	18	23	30	Stk38	chr1	3288	29114513	0.641958	0.596	0.434
NM_026421	16	17	25	12	Sfrs3	chr1	2566	29178752	0.947901	0.607	0.676
NM_133850	64	59	29	27	Cpne5	chr1	4420	29299288	0.947901	0.519	0.518
NM_172516	13	26	66	17	Ppil1	chr1	1215	29400834	0.280466	0.614	0.795
NM_198305	17	16	50	50	BC004004	chr1	2627	29439189	0.733758	0.353	0.5
NM_138672	23	17	44	46	Mtch1	chr1	1918	29475743	0.677707	0.596	0.489
NM_010148	38	29	161	82	Glo1	chr1	1958	30749422	0.395208	0.754	0.663
NM_021386	33	33	17	39	Akap8	chr1	3695	32446409	0.488544	0.484	0.304
NM_019586	36	25	15	29	Rab11b	chr1	6065	33882839	0.947901	0.417	0.341
NM_030559	45	45	23	8	Stk19	chr1	1078	34960962	0.947901	0.8	0.742
NM_173437	65	34	16	10	Rpp21	chr1	500	36392958	0.947901	0.688	0.615
NM_022314	27	31	84	48	Ppp1r11	chr1	1694	37088479	0.619795	0.488	0.636
NM_028810	19	28	57	14	Zfp57	chr1	1689	37146755	0.947901	0.75	0.803
NM_001081039	33	24	139	101	Gabbr1	chr1	4486	37209139	0.445503	0.482	0.579
NM_010422	21	15	100	121	Pla2g7	chr1	1923	43739797	0.841387	0.516	0.452

NM_013767	54	55	51	24	Slc25a27	chr1	2687	43790357	0.947901	0.685	0.68
NM_026453	18	11	50	22	Cdc5l	chr1	2994	45553908	0.2416	0.458	0.694
NM_008618	132	85	119	88	Mea1	chr1	841	46819833	0.947901	0.593	0.575
NM_172757	111	25	13	14	Rpl7l1	chr1	2606	46918804	0.866345	0.621	0.481
NM_173759	53	44	21	24	AI314976	chr1	1135	48556166	0.947901	0.544	0.467
NM_025877	39	25	21	17	Plcl2	chr1	4138	50827039	0.947901	0.632	0.553
NM_026165	27	24	19	11	Satb1	chr1	2922	51944649	0.947901	0.667	0.633
NM_026064	168	99	16	33	Rab5a	chr1	2364	53645685	0.886392	0.44	0.327
NM_172265	19	16	29	40	Fsd1	chr1	1748	56135357	0.805861	0.533	0.42
NM_009717	28	20	21	10	Mpnd	chr1	1701	56156061	0.947901	0.63	0.677
NM_008214	30	37	20	28	Ubxd1	chr1	1714	56207738	0.684867	0.543	0.417
NM_178252	18	18	33	27	D17Wsu104e	chr1	913	56323328	0.459791	0.348	0.55
NM_026369	37	55	29	13	Dpp9	chr1	3456	56351247	0.844976	0.577	0.69
NM_008775	42	45	29	21	Lonp1	chr1	2970	56766219	0.805861	0.462	0.58
NM_177814	20	14	26	14	Clpp	chr1	990	57132930	0.460468	0.456	0.65
NM_016775	25	30	37	35	Slc25a23	chr1	3384	57183264	0.844976	0.609	0.514
NM_145544	98	46	343	62	Tubb4	chr1	2246	57219949	0.935791	0.828	0.847
NM_144530	42	45	60	23	Rab31	chr1	3416	66001460	0.301567	0.526	0.723
NM_025978	54	7	29	27	Ppp4r1	chr1	3864	66190204	0.947901	0.56	0.518
NM_198862	43	51	127	140	Ndufv2	chr1	1472	66438641	0.947901	0.51	0.476
NM_145491	12	24	18	7	1110012J17Rik	chr1	7238	66797788	0.753949	0.86	0.72
NM_001025067	10	28	90	91	Epb4.1l3	chr1	4051	69633905	0.744899	0.588	0.497
NM_011696	60	57	323	221	Mylc2b	chr1	939	71326582	0.677707	0.546	0.594
NM_133656	17	8	70	53	2900073G15Rik	chr1	1779	71344077	0.841387	0.629	0.569
NM_026119	35	24	42	63	Ypel5	chr1	2869	73200176	0.74999	0.5	0.4
NM_013737	65	61	22	29	2410091C18Rik	chr1	2303	79346762	0.928685	0.533	0.431
NM_028712	18	24	75	37	Tmem178	chr1	1696	81400222	0.450421	0.514	0.67
NM_001025246	19	22	47	59	Slc8a1	chr1	18599	82137692	0.289667	0.648	0.443
NM_029249	24	28	24	15	Eml4	chr1	5125	83877255	0.92262	0.533	0.615
NM_175258	19	11	16	18	Rhoq	chr1	4159	87396579	0.833193	0.333	0.471
NM_172624	30	22	51	39	Cript	chr1	1137	87434387	0.947901	0.579	0.567
NM_013853	28	26	18	36	Msh2	chr1	3056	88122775	0.525209	0.519	0.333
NM_009859	33	26	14	13	Arhgap12	chr1	4959	6112289	0.947901	0.485	0.519
NM_170760	31	34	123	120	Rab18	chr1	2257	6789192	0.945732	0.451	0.506
NM_011399	22	28	105	138	Riok3	chr1	3661	12307327	0.9341	0.383	0.432
NM_001080755	43	8	949	975	Ttr	chr1	1051	20832212	0.947901	0.499	0.493
NM_010267	44	47	40	40	Dtna	chr1	6321	23574080	0.210831	0.707	0.5
NM_172730	23	9	133	91	Mapre2	chr1	4022	23962492	0.947901	0.602	0.594
NM_028761	14	18	35	46	Zfp191	chr1	3143	24173116	0.933057	0.344	0.432
NM_172961	31	45	16	10	D030070L09Rik	chr1	2360	24272650	0.947901	0.571	0.615
NM_007460	22	25	23	23	Fhod3	chr1	5439	25291506	0.947901	0.423	0.5
NM_013922	63	42	16	15	5730494M16Rik	chr1	1234	25316754	0.947901	0.578	0.516
NM_009774	14	15	103	18	Syt4	chr1	3901	31597586	0.174288	0.615	0.851
NM_026061	23	28	71	90	Slc25a46	chr1	4371	31741114	0.845646	0.529	0.441
NM_025562	65	96	147	106	D0H4S114	chr1	2223	33597639	0.774165	0.517	0.581
NM_023167	46	18	19	8	Brd8	chr1	4677	34784211	0.833193	0.559	0.704
NM_172664	22	18	26	22	Cdc23	chr1	4134	34797074	0.945732	0.455	0.542
NM_030210	43	25	34	25	Ctnna1	chr1	3713	35413504	0.947901	0.632	0.576
NM_021789	88	66	215	244	Paip2	chr1	1424	35776084	0.488654	0.532	0.468
NM_010733	52	37	107	73	Sra1	chr1	893	36827082	0.342344	0.455	0.594
NM_010251	26	19	132	125	Ndufa2	chr1	529	36902187	0.947901	0.486	0.514
NM_008948	63	71	28	32	Ik	chr1	1996	36917111	0.833193	0.581	0.467

NM_177239	17	11	96	85	Hars	chr1	1902	36931030	0.833193	0.448	0.53
NM_010681	14	12	19	28	Pcdhb9	chr1	3055	37560665	0.947901	0.321	0.404
NM_013587	98	79	15	13	Pcdhga2	chr1	4537	37828970	0.947901	0.538	0.536
NM_178915	18	24	15	13	Pcdhgb2	chr1	4739	37849936	0.947901	0.514	0.536
NM_027959	24	19	15	13	Pcdhga7	chr1	4660	37874519	0.947901	0.514	0.536
NM_007926	50	55	15	13	Pcdhga8	chr1	4730	37885373	0.947901	0.514	0.536
NM_146153	66	53	15	13	Pcdhgb5	chr1	4522	37890936	0.947901	0.481	0.536
NM_016737	20	17	15	13	Pcdhga9	chr1	4717	37896650	0.947901	0.514	0.536
NM_001081298	34	17	15	15	Pcdhga12	chr1	5080	37925322	0.947901	0.5	0.5
NM_023429	29	40	15	13	Pcdhgc3	chr1	4687	38000805	0.947901	0.538	0.536
NM_025319	237	223	15	13	Pcdhgc4	chr1	5047	38000805	0.947901	0.538	0.536
NM_007467	107	85	15	13	Pcdhgc5	chr1	4708	38000805	0.947901	0.514	0.536
NM_178662	23	21	15	15	Hdac3	chr1	2005	38112238	0.825608	0.636	0.5
NM_018799	25	25	136	104	Rnf14	chr1	2940	38476426	0.947901	0.563	0.567
NM_025933	104	94	28	23	Nr3c1	chr1	6345	39582203	0.947901	0.596	0.549
NM_001048167	36	38	71	55	Ppp2r2b	chr1	2029	42861158	0.709822	0.444	0.563
NM_025670	49	41	42	41	Sema6a	chr1	6901	47451612	0.844976	0.4	0.506
NM_021426	33	20	12	29	Gramd3	chr1	2611	56591901	0.947901	0.375	0.293
NM_011074	15	10	118	128	A730017C20Rik	chr1	1810	59236051	0.844976	0.546	0.48
NM_023721	41	42	84	58	Dctn4	chr1	3714	60685905	0.296511	0.381	0.592
NM_001081422	16	11	62	56	Csnk1a1	chr1	2013	61747121	0.947901	0.545	0.525
NM_019707	17	15	23	27	Napg	chr1	3496	63156473	0.947901	0.462	0.46
NM_174997	71	46	60	26	Txnl1	chr1	2558	63851852	0.629123	0.531	0.698
NM_001039000	205	143	25	17	Fech	chr1	2901	64621186	0.162269	0.343	0.595
NM_001038492	19	10	150	117	Nars	chr1	2616	64670117	0.947901	0.542	0.562
NM_146094	32	29	40	18	Lman1	chr1	3575	66156384	0.158693	0.47	0.69
NM_029478	16	11	189	218	Gnal	chr1	5223	67384427	0.381503	0.559	0.464
NM_025433	18	11	171	18	Slmo1	chr1	2167	67640097	0.947901	0.923	0.905
NM_011216	153	202	65	53	Spire1	chr1	4761	67690067	0.783653	0.435	0.551
NM_172994	45	32	28	22	Rnmt	chr1	3928	68482476	0.947901	0.5	0.56
NM_138598	19	21	74	58	Tcf4	chr1	7462	69847424	0.677707	0.446	0.561
NM_172592	19	32	33	35	Smad4	chr1	3361	73837684	0.947901	0.469	0.485
NM_026592	56	51	8	17	Gm672	chr1	6192	75796972	0.947901	0.25	0.32
NM_001042617	32	31	34	21	Ier3ip1	chr1	1369	77179390	0.716713	0.462	0.618
NM_030057	75	66	26	38	Atp5a1	chr1	2443	78021449	0.509331	0.551	0.406
NM_010740	17	20	37	9	Mbp	chr1	4820	82726462	0.947901	0.786	0.804
NM_025447	15	14	18	9	Tshz1	chr1	5814	84181095	0.947901	0.614	0.667
NM_025982	32	10	141	79	Cyb5	chr1	749	85047239	0.528489	0.532	0.641
NM_025336	13	17	96	80	Cbln2	chr1	2201	86886101	0.780711	0.639	0.545
NM_013507	104	104	52	40	Mrpl21	chr1	992	3292469	0.947901	0.539	0.565
NM_080287	30	40	317	262	Ndufs8	chr1	963	3912618	0.947901	0.534	0.547
NM_173439	26	17	56	60	Ndufv1	chr1	1572	4011179	0.947901	0.5	0.483
NM_054054	18	34	118	151	Gstp2	chr1	785	4041671	0.947901	0.48	0.439
NM_011638	26	14	20	18	BC021614	chr1	683	4057628	0.947901	0.514	0.526
NM_028152	20	21	47	42	Ccs	chr1	1057	4839239	0.465109	0.414	0.528
NM_152915	107	87	17	20	Mrpl11	chr1	821	4962327	0.947901	0.485	0.459
NM_024206	45	35	28	17	Slc29a2	chr1	2033	5031014	0.947901	0.615	0.622
NM_139146	27	23	89	39	B3gnt1	chr1	2035	5040820	0.947901	0.655	0.695
NM_019879	39	32	88	69	Cfl1	chr1	1196	5493733	0.947901	0.598	0.561
NM_026482	49	34	18	12	Mrpl49	chr1	1722	6054612	0.684867	0.405	0.6
NM_028981	20	28	117	107	Prdx5	chr1	688	6981419	0.184023	0.694	0.522
NM_053086	38	21	30	16	Stip1	chr1	2080	7110010	0.86501	0.541	0.652

NM_025277	40	23	16	16	Rtn3	chr1	5030	7557724	0.915297	0.611	0.5
NM_020580	24	29	19	9	Polr2g	chr1	868	8867667	0.651863	0.521	0.679
NM_008717	21	38	405	273	Gng3	chr1	1699	8912106	0.947901	0.612	0.597
NM_011116	99	68	14	13	Bscl2	chr1	1654	8921932	0.947901	0.595	0.519
NM_026697	45	36	32	26	D19Ertd721e	chr1	1048	8948739	0.920751	0.647	0.552
NM_025364	46	39	14	13	1810009A15Rik	chr1	615	8964994	0.947901	0.554	0.519
NM_173399	23	52	12	13	B3gat3	chr1	1599	9000814	0.502018	0.717	0.48
NM_172436	18	26	134	104	Eef1g	chr1	1565	9051829	0.882258	0.53	0.563
NM_025887	11	14	82	53	Fads1	chr1	3408	10267469	0.850825	0.525	0.607
NM_153543	22	20	17	19	5730453I16Rik	chr1	3500	10620471	0.947901	0.43	0.472
NM_001013378	18	9	26	10	Mrpl16	chr1	1134	11848646	0.783653	0.575	0.722
NM_010518	182	119	25	10	AV312086	chr1	4054	12017965	0.947901	0.775	0.714
NM_053073	20	19	15	12	Osbp	chr1	4590	12066271	0.947901	0.452	0.556
NM_025344	16	13	24	9	Dtx4	chr1	5243	12566385	0.947901	0.676	0.727
NM_008138	85	141	127	88	Gnaq	chr1	5634	16460649	0.947901	0.574	0.591
NM_172255	55	52	60	78	Gda	chr1	5417	21508722	0.753949	0.551	0.435
NM_023464	49	41	87	78	Smarca2	chr1	5831	26848785	0.947901	0.5	0.527
NM_008802	39	39	23	19	D19Bwg1357e	chr1	3462	27495859	0.604275	0.727	0.548
NM_001013380	15	11	43	38	Slc1a1	chr1	3708	28987870	0.764991	0.649	0.531
NM_025514	26	16	17	14	Jak2	chr1	4947	29386426	0.947901	0.565	0.548
NM_028873	14	22	32	21	Minpp1	chr1	2619	32589122	0.947901	0.674	0.604
NM_001081011	45	42	119	91	Atad1	chr1	2813	32776098	0.947901	0.591	0.567
NM_009458	18	25	21	15	Stambpl1	chr1	1978	34314569	0.947901	0.579	0.583
NM_001081114	66	59	18	19	Ide	chr1	5104	37405084	0.947901	0.523	0.486
NM_027016	26	15	104	61	Sorbs1	chr1	5275	40467945	0.947901	0.618	0.63
NM_011965	45	66	63	57	Tm9sf3	chr1	6144	41313179	0.947901	0.533	0.525
NM_001026212	15	10	24	38	Mms19l	chr1	3965	42038828	0.876174	0.488	0.387
NM_025278	15	16	45	42	BC023055	chr1	1817	42153067	0.929707	0.446	0.517
NM_023831	91	63	19	21	Got1	chr1	2065	43590264	0.805861	0.609	0.475
NM_033564	24	3	415	346	Scd2	chr1	5032	44381069	0.766362	0.505	0.545
NM_144921	50	33	89	77	Ndufb8	chr1	641	44629516	0.863684	0.451	0.536
NM_172614	48	47	42	16	Sfxn3	chr1	2870	45130848	0.497334	0.54	0.724
NM_001030014	17	8	9	19	Btrc	chr1	6358	45606825	0.409809	0.595	0.321
NM_025974	229	174	33	26	Mgea5	chr1	4797	45857604	0.915297	0.482	0.559
NM_009840	69	64	52	40	Nolc1	chr1	3649	46156792	0.888353	0.644	0.565
NM_026468	64	64	180	155	Actr1a	chr1	2742	46456870	0.947901	0.513	0.537
NM_018743	43	59	412	478	Arl3	chr1	838	46626117	0.947901	0.466	0.463
NM_138669	33	47	25	15	D19Wsu162e	chr1	3921	46730963	0.947901	0.689	0.625
NM_026277	23	18	40	37	Nt5c2	chr1	2728	46979569	0.637683	0.667	0.519
NM_009930	23	7	34	15	Ina	chr1	2272	47097958	0.947901	0.654	0.694
NM_023799	41	44	36	38	Pcgf6	chr1	2126	47123499	0.947901	0.538	0.486
NM_026007	1255	1113	286	458	Usmg5	chr1	370	47160532	0.677707	0.432	0.384
NM_173181	34	33	43	55	Gsto1	chr1	1163	47938826	0.947901	0.451	0.439
NM_080469	26	18	36	19	Mxi1	chr1	4843	53448352	0.385624	0.806	0.655
NM_001003934	22	14	20	17	Shoc2	chr1	3964	54103964	0.947901	0.5	0.541
NM_183251	20	17	85	64	Tcf7l2	chr1	3230	56006168	0.947901	0.585	0.57
NM_009158	54	63	49	39	Hspa12a	chr1	5733	58902090	0.931904	0.481	0.557
NM_029334	32	40	105	75	4930506M07Rik	chr1	3767	59116445	0.608853	0.485	0.583
NM_025882	32	24	23	35	1810055E12Rik	chr1	2307	60911725	0.947901	0.464	0.397
NM_008704	70	62	21	29	Prdx3	chr1	1445	60941120	0.947901	0.483	0.42
NM_001081119	13	13	19	11	Sephs1	chr2	5329	4830794	0.947901	0.688	0.633
NM_181070	32	39	55	45	Phyh	chr2	1474	4859622	0.945732	0.486	0.55

NM_009846	201	138	70	71	Cdc123	chr2	1623	5765904	0.146417	0.333	0.496
NM_015751	152	142	40	34	Sec61a2	chr2	2369	5807597	0.947901	0.568	0.541
NM_026455	37	45	29	27	Kin	chr2	1379	10013550	0.947901	0.516	0.518
NM_175692	377	399	16	12	Rbm17	chr2	1583	11518348	0.947901	0.55	0.571
NM_008745	25	18	10	17	Pip5k2a	chr2	3471	18810697	0.935791	0.484	0.37
NM_026437	13	15	149	108	Gad2	chr2	5625	22546204	0.947901	0.551	0.58
NM_025578	41	40	74	48	Ssna1	chr2	864	25126700	0.915297	0.544	0.607
NM_008155	146	112	132	83	Npdc1	chr2	1473	25264449	0.296511	0.508	0.614
NM_028754	50	46	27	19	BC029214	chr2	997	25315043	0.947901	0.621	0.587
NM_199466	56	49	200	201	Ptgds	chr2	806	25323360	0.289667	0.586	0.499
NM_138668	21	12	66	74	Fbxw5	chr2	2359	25359235	0.947901	0.529	0.471
NM_009932	26	30	165	183	Phpt1	chr2	583	25429214	0.601949	0.56	0.474
NM_023215	20	21	24	19	Kcnt1	chr2	5893	25764790	0.947901	0.563	0.558
NM_011286	47	28	40	39	Gpsm1	chr2	3357	26200783	0.80737	0.617	0.506
NM_025554	28	35	14	16	D2Bwg1335e	chr2	2913	26203955	0.463681	0.707	0.467
NM_130878	20	11	26	11	Surf2	chr2	1170	26774719	0.706186	0.535	0.703
NM_153117	62	52	58	40	Olfm1	chr2	2686	28084609	0.646804	0.476	0.592
NM_026511	52	16	13	12	Med27	chr2	1232	29379889	0.947901	0.514	0.52
NM_146243	25	8	27	28	Spna2	chr2	8021	29886282	0.947901	0.522	0.491
NM_178655	174	127	62	57	Ppp2r4	chr2	2574	30302146	0.587635	0.634	0.521
NM_198108	29	36	29	13	Tor1b	chr2	2997	30813838	0.602323	0.51	0.69
NM_021446	16	17	22	19	BC005624	chr2	1293	30828685	0.947901	0.607	0.537
NM_009433	19	16	144	115	5830434P21Rik	chr2	8556	32086277	0.947901	0.546	0.556
NM_007404	25	22	102	104	Stxbp1	chr2	3874	32679065	0.947901	0.517	0.495
NM_026390	16	13	234	21	Rpl12	chr2	643	32818144	0.805861	0.86	0.918
NM_001039094	23	36	24	28	Garnl3	chr2	3387	32846109	0.933307	0.547	0.462
NM_011173	29	5	96	111	Hspa5	chr2	2539	34629364	0.381503	0.604	0.464
NM_025827	12	29	98	60	Rab14	chr2	3100	35036126	0.89468	0.556	0.62
NM_178399	129	97	75	73	Ndufa8	chr2	739	35904769	0.947901	0.534	0.507
NM_001040106	103	95	25	24	Rbm18	chr2	2452	35982702	0.947901	0.452	0.51
NM_028611	16	14	21	35	Rabgap1	chr2	4967	37421294	0.573356	0.571	0.375
NM_009615	13	13	80	59	Strbp	chr2	2847	37439201	0.947901	0.564	0.576
NM_029425	21	15	39	16	Orc4l	chr2	4029	48804699	0.947901	0.676	0.709
NM_001001881	17	32	18	29	Kif5c	chr2	6867	49626748	0.947901	0.436	0.383
NM_015747	21	14	34	22	2010311D03Rik	chr2	1302	50143318	0.947901	0.629	0.607
NM_001081557	45	33	14	12	Rnd3	chr2	2827	51004623	0.848802	0.404	0.538
NM_172661	220	183	77	59	Psmd14	chr2	1560	61638140	0.947901	0.525	0.566
NM_172302	55	73	57	77	Ppig	chr2	6297	69587917	0.947901	0.385	0.425
NM_001033347	24	29	19	28	Phospho2	chr2	2104	69634712	0.780086	0.55	0.404
NM_177700	15	14	70	44	Klhl23	chr2	4511	69673877	0.783653	0.514	0.614
NM_007508	61	51	282	207	Gad1	chr2	3231	70439150	0.44857	0.47	0.577
NM_133839	39	23	62	107	Gorasp2	chr2	2239	70528778	0.947901	0.38	0.367
NM_172701	62	34	38	47	Tlk1	chr2	4080	70624997	0.863684	0.55	0.447
NM_178613	13	19	293	145	Dync1i2	chr2	2443	71100873	0.25908	0.558	0.669
NM_010580	16	30	39	39	Slc25a12	chr2	2924	71162153	0.890243	0.409	0.5
NM_029601	44	50	14	13	Chn1	chr2	1025	73462938	0.947901	0.536	0.519
NM_033609	16	11	35	29	Ttc30b	chr2	2634	75774280	0.943372	0.459	0.547
NM_024203	18	12	35	32	Osbpl6	chr2	8055	76434049	0.947901	0.529	0.522
NM_001004361	26	19	25	27	Prkra	chr2	1554	76481318	0.947901	0.407	0.481
NM_025762	20	5	33	29	Pde1a	chr2	4271	79969058	0.947901	0.615	0.532
NM_133998	33	28	57	56	Dnajc10	chr2	4054	80193598	0.947901	0.489	0.504
NM_153406	15	24	37	41	Ctnnd1	chr2	5475	84490725	0.677707	0.645	0.474

NM_025389	14	20	53	28	Txndc14	chr2	1968	84515960	0.602323	0.522	0.654
NM_023210	21	17	96	88	Timm10	chr2	682	84670080	0.265994	0.369	0.522
NM_030225	107	55	67	58	Psmc3	chr2	1569	90899565	0.859534	0.47	0.536
NM_025997	24	20	37	32	Madd	chr2	6009	91019416	0.947901	0.533	0.536
NM_172271	40	30	19	14	Arhgap1	chr2	3086	91509819	0.947901	0.536	0.576
NM_009084	38	29	44	32	Trp53i11	chr2	2687	93040748	0.813566	0.463	0.579
NM_175149	18	29	80	87	Hsd17b12	chr2	1839	93955217	0.947901	0.458	0.479
NM_172590	17	15	39	17	Api5	chr2	3702	94267219	0.947901	0.731	0.696
NM_133722	41	30	103	73	B230118H07Rik	chr2	2269	101469090	0.86501	0.523	0.585
NM_024182	41	66	20	23	Commd9	chr2	1175	101739028	0.712972	0.585	0.465
NM_001025372	43	37	171	127	Trim44	chr2	5612	102140320	0.947901	0.549	0.574
NM_029436	14	15	18	22	Apip	chr2	896	102931990	0.677707	0.59	0.45
NM_026503	18	9	56	52	Cat	chr2	2551	103325178	0.947901	0.477	0.519
NM_177326	17	12	35	57	D430041D05Rik	chr2	10124	104095228	0.936394	0.453	0.38
NM_178110	58	44	85	102	Pcid1	chr2	1230	104855000	0.490713	0.575	0.455
NM_133360	25	25	21	14	Slc12a6	chr2	5970	112200822	0.947901	0.677	0.6
NM_011462	55	29	114	161	2900064A13Rik	chr2	1072	112307347	0.947901	0.385	0.415
NM_175199	25	27	2	26	Actc1	chr2	1390	113874942	0.607002	0	0.071
NM_001040396	118	94	62	37	Mrg1	chr2	2900	115890553	0.947901	0.639	0.626
NM_133198	4	49	58	41	Srp14	chr2	790	118305115	0.791261	0.462	0.586
NM_026113	58	43	76	49	Disp2	chr2	6430	118615568	0.14354	0.409	0.608
NM_026914	20	14	46	53	Ccdc32	chr2	1870	118852998	0.947901	0.5	0.465
NM_133780	48	64	30	24	1200015F23Rik	chr2	2170	118973185	0.780488	0.4	0.556
NM_025598	40	47	25	38	Mapkbp1	chr2	6943	119852056	0.947901	0.405	0.397
NM_145542	27	28	13	21	Zfp106	chr2	9100	120359323	0.947901	0.464	0.382
NM_029861	47	45	39	42	Ccndbp1	chr2	1572	120842192	0.947901	0.438	0.481
NM_021559	11	21	64	57	Ascc3l1	chr2	6740	127065882	0.696682	0.407	0.529
NM_001081276	13	22	96	56	Tmem127	chr2	4401	127083009	0.806139	0.537	0.632
NM_025449	34	24	48	30	Tmem87b	chr2	4631	128677556	0.511027	0.442	0.615
NM_011289	149	119	31	14	Slc20a1	chr2	3272	129036539	0.945732	0.6	0.689
NM_026127	62	32	33	28	Nol5a	chr2	1904	130101824	0.947901	0.576	0.541
NM_009196	17	20	78	81	Idh3b	chr2	1505	130109503	0.805861	0.428	0.491
NM_028235	17	20	58	42	Vps16	chr2	2617	130263697	0.846223	0.5	0.58
NM_176987	16	13	79	97	Ptpra	chr2	3070	130379165	0.562731	0.571	0.449
NM_010910	17	17	74	63	4930402H24Rik	chr2	4975	130646267	0.839655	0.622	0.54
NM_009169	48	46	17	9	1700037H04Rik	chr2	1241	130972173	0.947901	0.586	0.654
NM_013855	60	64	19	22	Rnf24	chr2	5498	131127809	0.947901	0.5	0.463
NM_145557	8	19	34	36	Rassf2	chr2	4646	131828677	0.947901	0.549	0.486
NM_133348	139	111	79	31	Chgb	chr2	2733	132618289	0.204536	0.572	0.718
NM_025862	10	17	219	158	Snap25	chr2	2135	136607140	0.947901	0.579	0.581
NM_016860	162	154	119	106	Snrpb2	chr2	1230	142890913	0.947901	0.494	0.529
NM_028963	38	19	109	79	Pcsk2	chr2	4720	143641474	0.947901	0.614	0.58
NM_025463	77	62	233	187	Dstn	chr2	1639	143767870	0.947901	0.559	0.555
NM_146107	14	20	47	46	Snx5	chr2	2375	144076019	0.947901	0.548	0.505
NM_001032727	37	35	24	35	Polr3f	chr2	4160	144367389	0.947901	0.423	0.407
NM_025442	17	40	28	21	Cd93	chr2	6638	148265467	0.86501	0.459	0.571
NM_133749	15	24	28	37	Napb	chr2	3693	148558078	0.947901	0.419	0.431
NM_026203	104	98	43	22	Zfp120	chr2	4096	149940211	0.947901	0.68	0.662
NM_153563	42	37	16	21	Pygb	chr2	3860	150655400	0.947901	0.356	0.432
NM_178888	29	24	3	36	Abhd12	chr2	1967	150672006	0.709822	0.176	0.077
NM_201357	23	18	21	35	Nsfl1c	chr2	1355	151335272	0.447777	0.529	0.375
NM_080633	49	47	31	21	Snph	chr2	4689	151426725	0.947901	0.516	0.596

NM_009451	849	176	33	31	Nrsn2	chr2	1437	152199895	0.774973	0.402	0.516
NM_207583	21	19	99	40	Pdrg1	chr2	1229	152834746	0.807174	0.648	0.712
NM_007414	51	49	17	15	Tspyl3	chr2	3070	153048467	0.947901	0.5	0.531
NM_008388	46	47	27	16	Asxl1	chr2	6674	153225408	0.947901	0.606	0.628
NM_025605	25	25	12	65	8430427H17Rik	chr2	5820	153246412	0.782756	0.265	0.156
NM_025614	137	127	31	40	Commd7	chr2	3467	153454974	0.844976	0.52	0.437
NM_010726	34	36	218	164	Mapre1	chr2	7330	153598445	0.945732	0.536	0.571
NM_018800	25	19	92	23	Cbfa2t2	chr2	6100	154363745	0.695724	0.688	0.8
NM_134255	27	24	62	51	Ergic3	chr2	1297	155841741	0.698719	0.457	0.549
NM_026275	14	19	1379	1486	A930034L06Rik	chr2	5864	158211394	0.947901	0.486	0.481
NM_009828	11	16	229	129	Ywhab	chr2	2775	163843114	0.490713	0.721	0.64
NM_027453	26	16	19	17	Ctsa	chr2	3547	164666396	0.86501	0.655	0.528
NM_007896	118	102	29	28	Elmo2	chr2	4600	165140258	0.915297	0.429	0.509
NM_146036	31	36	10	17	Zfp334	chr2	5033	165204704	0.947901	0.333	0.37
NM_172606	16	20	52	37	Cse1l	chr2	3505	166771206	0.747754	0.482	0.584
NM_008845	15	16	21	29	Stau1	chr2	2996	166780499	0.751919	0.551	0.42
NM_053102	78	62	25	23	B4galt5	chr2	4205	167138710	0.947901	0.482	0.521
NM_001039472	67	45	78	90	Zfp313	chr2	3001	167340222	0.648107	0.554	0.464
NM_027373	16	13	17	21	Ube2v1	chr2	1950	167433177	0.947901	0.493	0.447
NM_178745	192	126	14	12	Ptpn1	chr2	4213	167800318	0.947901	0.552	0.538
NM_011889	41	36	15	11	Atp9a	chr2	3529	168474164	0.947901	0.54	0.577
NM_026324	15	15	19	102	Vapb	chr2	6969	173603621	0.780086	0.257	0.157
NM_001037756	15	10	101	96	Gnas	chr2	3733	174123878	0.805861	0.458	0.513
NM_178347	20	24	61	53	Th1l	chr2	2279	174250641	0.882217	0.453	0.535
NM_001045533	27	23	177	164	Atp5e	chr2	419	174288018	0.947901	0.527	0.519
NM_029409	56	68	43	40	Phactr3	chr2	2658	178070258	0.947901	0.568	0.518
NM_009418	47	64	389	382	Psma7	chr2	881	179773442	0.72737	0.54	0.505
NM_008991	54	41	39	26	Rps21	chr2	396	179992374	0.947901	0.533	0.6
NM_025411	76	62	17	13	Cables2	chr2	2945	179993431	0.947901	0.563	0.567
NM_029654	18	16	25	23	C030019F02Rik	chr2	944	180677759	0.86501	0.623	0.521
NM_025623	14	11	13	23	Arfgap1	chr2	2595	180715826	0.58647	0.593	0.361
NM_026091	17	12	25	16	Chrna4	chr2	4509	180763082	0.947901	0.686	0.61
NM_001033286	12	14	94	110	2700038C09Rik	chr2	797	180922089	0.947901	0.46	0.461
NM_146093	22	12	38	81	Stmn3	chr2	1136	181049195	0.194015	0.464	0.319
NM_013884	35	36	15	19	Arfrp1	chr2	2404	181092708	0.947901	0.378	0.441
NM_025607	24	13	47	38	Dnajc5	chr2	3674	181286200	0.84104	0.455	0.553
NM_027457	12	27	37	20	Polr3k	chr2	2925	181605105	0.947901	0.667	0.649
NM_007466	19	7	99	69	Pkia	chr3	3683	7442165	0.552476	0.444	0.589
NM_018745	26	15	50	64	3110050N22Rik	chr3	3361	7551577	0.915297	0.507	0.439
NM_134141	25	20	89	79	Stmn2	chr3	1904	8541914	0.947901	0.559	0.53
NM_009698	131	177	41	21	Tpd52	chr3	2293	8929745	0.602323	0.5	0.661
NM_011787	40	35	124	153	Zfp704	chr3	13849	9565087	0.204536	0.568	0.448
NM_009602	27	27	34	20	Snx16	chr3	2287	10420862	0.947901	0.586	0.63
NM_009761	72	81	71	52	Armc1	chr3	2955	19032240	0.947901	0.585	0.577
NM_178737	127	64	17	25	Pde7a	chr3	3165	19136522	0.877763	0.5	0.405
NM_011801	21	24	233	141	Rpl22l1	chr3	477	28705712	0.947901	0.605	0.623
NM_175383	19	10	90	72	Tloc1	chr3	3966	30717531	0.915297	0.634	0.556
NM_026070	27	29	22	28	Prkci	chr3	4465	30949212	0.947901	0.5	0.44
NM_029505	35	31	31	30	Gnb4	chr3	2753	32497066	0.411721	0.698	0.508
NM_009804	21	23	17	10	Mrpl47	chr3	850	32629050	0.947901	0.629	0.63
NM_175003	76	35	23	6	Ttc14	chr3	11072	33710230	0.844976	0.885	0.793
NM_009658	61	14	43	11	Dcun1d1	chr3	3545	35818002	0.161627	0.525	0.796

NM_019835	27	29	31	67	Ccna2	chr3	2827	36463910	0.924899	0.407	0.316
NM_207219	37	31	45	128	Exosc9	chr3	1622	36464390	0.062787	0.405	0.26
NM_029716	15	13	50	82	Slc7a11	chr3	9227	50239323	0.947901	0.318	0.379
NM_009870	30	32	93	59	Rab33b	chr3	3488	51299072	0.947901	0.592	0.612
NM_133700	20	14	30	16	Cog6	chr3	2920	52821072	0.947901	0.65	0.652
NM_025821	44	49	33	22	Lhfp	chr3	2107	53065376	0.947901	0.531	0.6
NM_175454	44	9	30	32	Exosc8	chr3	1316	54532686	0.947901	0.512	0.484
NM_175642	25	35	17	28	Alg5	chr3	1471	54550495	0.945732	0.298	0.378
NM_007684	53	48	45	18	Rfxap	chr3	2185	54608327	0.947901	0.68	0.714
NM_029846	16	9	32	12	Commd2	chr3	2961	57454006	0.785646	0.615	0.727
NM_153557	18	11	168	135	2810407C02Rik	chr3	3575	58395344	0.947901	0.557	0.554
NM_007509	37	105	26	21	Rap2b	chr3	4197	61169218	0.817803	0.429	0.553
NM_021492	68	54	176	83	Serpini1	chr3	2944	75418507	0.409809	0.574	0.68
NM_009723	13	16	46	52	Ppid	chr3	1728	79407507	0.805861	0.554	0.469
NM_009680	15	11	282	364	Gria2	chr3	6841	80545008	0.381503	0.533	0.437
NM_145851	18	14	52	46	Glrb	chr3	2988	80664171	0.947901	0.559	0.531
NM_013472	18	14	81	46	Gucy1a3	chr3	4722	81948843	0.342045	0.485	0.638
NM_026009	23	14	32	21	Plrg1	chr3	1771	82876144	0.947901	0.56	0.604
NM_001081960	81	87	27	25	Trim2	chr3	7177	83997190	0.947901	0.538	0.519
NM_028386	33	34	70	43	Dclk2	chr3	4061	86709876	0.750419	0.518	0.619
NM_026396	22	24	15	27	Crabp2	chr3	900	87756948	0.947901	0.36	0.357
NM_153508	25	30	11	14	Ubqln4	chr3	3372	88372225	0.947901	0.529	0.44
NM_013799	50	36	37	29	2810403A07Rik	chr3	2964	88502813	0.774973	0.689	0.561
NM_194462	43	48	426	305	Syt11	chr3	4585	88576371	0.151666	0.487	0.583
NM_007620	32	14	17	13	Msto1	chr3	1791	88716673	0.947901	0.643	0.567
NM_025983	164	147	59	37	Rusc1	chr3	3388	88895966	0.805861	0.484	0.615
NM_133766	29	14	32	21	Fdps	chr3	1223	88904636	0.947901	0.647	0.604
NM_026487	39	27	34	20	Hcn3	chr3	3268	88956967	0.774044	0.774	0.63
NM_024166	60	21	16	10	Scamp3	chr3	1482	88986307	0.947901	0.584	0.615
NM_007471	121	130	69	39	Trim46	chr3	2731	89046146	0.573356	0.5	0.639
NM_019767	49	37	67	81	Krtcap2	chr3	516	89053636	0.74999	0.537	0.453
NM_019665	24	16	132	114	Pmvk	chr3	989	89271490	0.719377	0.6	0.537
NM_028840	31	22	69	53	Adar	chr3	5870	89555713	0.744899	0.459	0.566
NM_016778	25	15	44	48	Chrnb2	chr3	5358	89566935	0.947901	0.5	0.478
NM_009689	16	15	26	5	4933434E20Rik	chr3	1504	89865638	0.947901	0.8	0.839
NM_024173	91	91	19	13	Tpm3	chr3	1906	89903790	0.826184	0.466	0.594
NM_184053	83	91	105	121	Ilf2	chr3	1973	90292238	0.947901	0.493	0.465
NM_015730	24	11	43	69	Snapap	chr3	1898	90294487	0.947901	0.355	0.384
NM_183165	16	17	53	70	2500003M10Rik	chr3	3247	90310604	0.947901	0.488	0.431
NM_025272	53	36	59	40	Snx27	chr3	6259	94305716	0.805861	0.524	0.596
NM_007636	90	59	27	34	Psmb4	chr3	923	94690005	0.947901	0.465	0.443
NM_010761	21	27	12	13	Prune	chr3	3160	95085562	0.947901	0.486	0.48
NM_001024602	15	23	15	17	Ensa	chr3	2276	95433673	0.890049	0.6	0.469
NM_009829	78	19	13	12	Prpf3	chr3	2732	95659633	0.947901	0.536	0.52
NM_029702	17	28	99	90	Mrps21	chr3	427	95674488	0.603385	0.41	0.524
NM_054052	14	12	21	25	Anp32e	chr3	2826	95749478	0.92262	0.553	0.457
NM_199310	47	47	19	17	Pex11b	chr3	1580	96447630	0.947901	0.517	0.528
NM_007437	21	18	7	26	Prkab2	chr3	4278	97474034	0.176806	0.524	0.212
NM_001080384	28	29	45	20	Wdr3	chr3	3458	99966272	0.947901	0.655	0.692
NM_145221	25	22	41	27	Gdap2	chr3	2871	100005953	0.58647	0.421	0.603
NM_007461	27	15	83	54	Igsf3	chr3	4565	101261778	0.520239	0.733	0.606
NM_146033	279	220	78	549	Csde1	chr3	4130	102859344	0.290673	0.188	0.124

NM_011792	53	27	34	36	Syt6	chr3	4416	103436452	0.933057	0.568	0.486
NM_015731	67	57	28	47	Lrig2	chr3	7134	104298033	0.833193	0.263	0.373
NM_027404	26	18	41	34	Slc16a1	chr3	4426	104460634	0.941663	0.459	0.547
NM_007596	21	12	23	26	Kcnd3	chr3	7182	105262174	0.947901	0.532	0.469
NM_173016	52	40	68	6	6530418L21Rik	chr3	3538	105522281	0.067764	0.654	0.919
NM_019718	208	238	58	37	Wdr77	chr3	3553	105770293	0.668559	0.712	0.611
NM_144869	19	18	43	42	Slc6a17	chr3	6326	107303446	0.947431	0.571	0.506
NM_144558	66	68	49	35	6330569M22Rik	chr3	3235	107427544	0.947901	0.532	0.583
NM_015740	49	51	127	106	Ahcyl1	chr3	3866	107476947	0.947901	0.491	0.545
NM_009672	12	16	101	58	Gstm5	chr3	911	107701122	0.318235	0.456	0.635
NM_007586	16	36	43	27	Gstm1	chr3	1286	107819504	0.947901	0.574	0.614
NM_007581	26	27	57	40	Gnai3	chr3	3211	107926471	0.947901	0.545	0.588
NM_207678	18	20	42	36	Psma5	chr3	1217	108082661	0.947901	0.533	0.538
NM_146124	15	13	52	18	Celsr2	chr3	10543	108215323	0.617974	0.576	0.743
NM_011789	49	36	113	231	Wdr47	chr3	4096	108447470	0.525209	0.431	0.328
NM_009685	16	10	57	34	Extl2	chr3	2840	115729949	0.525209	0.46	0.626
NM_007687	58	39	92	73	Rtcd1	chr3	1493	116210926	0.947901	0.532	0.558
NM_001082483	47	55	57	43	D3Bwg0562e	chr3	5706	117038393	0.947901	0.577	0.57
NM_018742	26	21	41	34	4833424O15Rik	chr3	4044	117323967	0.947901	0.583	0.547
NM_133764	228	179	10	21	Alg14	chr3	910	121064566	0.807174	0.445	0.323
NM_008144	22	15	29	26	Cnn3	chr3	2003	121160245	0.947901	0.569	0.527
NM_178645	45	42	67	50	Abcd3	chr3	3399	121482487	0.947901	0.568	0.573
NM_001039939	20	13	24	22	Arhgap29	chr3	5133	121693983	0.751955	0.692	0.522
NM_009686	24	9	20	26	G430022H21Rik	chr3	2595	123088719	0.525209	0.613	0.435
NM_009721	21	16	56	42	Camk2d	chr3	4286	126541229	0.805361	0.694	0.571
NM_175312	18	11	287	184	Ank2	chr3	5790	126684813	0.929497	0.578	0.609
NM_009795	56	66	27	14	Larp7	chr3	2135	127249226	0.947901	0.667	0.659
NM_001081378	209	254	75	43	Elovl6	chr3	5951	129338189	0.947901	0.672	0.636
NM_172717	31	15	52	31	Sec24b	chr3	4702	129743614	0.947901	0.647	0.627
NM_021505	107	73	39	89	2310008M10Rik	chr3	1066	130412183	0.286602	0.471	0.305
NM_029007	17	11	25	19	Scye1	chr3	1122	132346765	0.86501	0.476	0.568
NM_029277	16	17	57	66	Ppa2	chr3	1225	133040903	0.652252	0.564	0.463
NM_198111	35	16	37	14	Tspan5	chr3	3172	138566253	0.078999	0.417	0.725
NM_016843	16	19	114	40	Rap1gds1	chr3	3516	138713490	0.841387	0.681	0.74
NM_007590	394	147	22	33	Hs2st1	chr3	4748	144128152	0.947901	0.429	0.4
NM_146247	33	40	322	276	Sep15	chr3	1515	144259864	0.947901	0.557	0.538
NM_172635	31	9	113	40	Lphn2	chr3	6052	148553471	0.86501	0.667	0.739
NM_028717	26	22	44	73	Fubp1	chr3	6491	151895528	0.186303	0.655	0.376
NM_146168	12	13	53	16	Zzz3	chr3	7601	152124214	0.870583	0.843	0.768
NM_144885	37	24	68	82	Negr1	chr3	4985	156679173	0.945732	0.39	0.453
NM_011811	13	16	83	22	Rps20	chr4	502	3761623	0.947901	0.817	0.79
NM_153195	17	8	40	25	Sdcbp	chr4	2550	6320175	0.947901	0.63	0.615
NM_053204	20	16	46	35	Nsmaf	chr4	3494	6362228	0.947901	0.58	0.568
NM_153585	48	22	8	22	Rab2	chr4	2057	8488306	0.112395	0.636	0.267
NM_010027	32	17	146	76	6720467C03Rik	chr4	1349	12098326	0.805861	0.745	0.658
NM_177664	30	22	50	37	Otud6b	chr4	3311	14745385	0.744899	0.432	0.575
NM_023314	20	15	27	12	Fut9	chr4	11990	25547590	0.947901	0.727	0.692
NM_001039514	22	23	52	52	Ube2j1	chr4	3522	33136675	0.844976	0.59	0.5
NM_010926	28	18	26	32	Orc3l	chr4	3793	34562144	0.381503	0.644	0.448
NM_026065	442	382	45	18	1810030N24Rik	chr4	927	34725511	0.677707	0.538	0.714
NM_133769	19	27	19	16	Aco1	chr4	4319	40144646	0.625773	0.727	0.543
NM_153166	70	65	44	60	Dnaja1	chr4	3323	40680388	0.395208	0.585	0.423

NM_013492	57	39	36	30	Chmp5	chr4	1372	40911633	0.093787	0.331	0.545
NM_133833	27	42	23	21	Ube2r2	chr4	3312	41138419	0.945732	0.424	0.523
NM_028194	24	11	19	14	Wdr40a	chr4	3402	41238435	0.947901	0.552	0.576
NM_008033	31	23	38	32	Unc13b	chr4	6374	43276000	0.947901	0.51	0.543
NM_138677	5	45	34	39	Clta	chr4	1109	44045297	0.947901	0.491	0.466
NM_009923	30	15	38	39	Rnf38	chr4	5040	44156446	0.121045	0.688	0.494
NM_197982	14	135	129	109	D4Wsu132e	chr4	3203	44944082	0.80737	0.468	0.542
NM_019794	14	12	24	38	Zbtb5	chr4	4219	45004137	0.877306	0.307	0.387
NM_130450	78	38	54	33	Igfbpl1	chr4	2729	45826267	0.947901	0.602	0.621
NM_030205	10	20	180	30	Tmeff1	chr4	2442	48675159	0.329905	0.722	0.857
NM_013509	20	11	37	53	Mrpl50	chr4	2103	49527284	0.947901	0.485	0.411
NM_146099	31	14	61	34	2810432L12Rik	chr4	2847	49597809	0.72737	0.519	0.642
NM_010174	55	21	32	14	Rnf20	chr4	4172	49668354	0.947901	0.633	0.696
NM_153743	121	97	30	15	Nipsnap3a	chr4	1507	53034281	0.929452	0.56	0.667
NM_029976	23	20	33	87	Txn1	chr4	1051	57956580	0.712972	0.405	0.275
NM_024181	23	24	49	39	Gng10	chr4	1120	59053958	0.893596	0.635	0.557
NM_013902	195	220	15	16	Ugcg	chr4	3719	59234115	0.947901	0.441	0.484
NM_172625	16	12	22	31	Snx30	chr4	7507	59914751	0.653115	0.567	0.415
NM_030238	368	258	90	100	Atp6v1g1	chr4	1104	63211163	0.947901	0.5	0.474
NM_008010	15	11	42	17	Tnc	chr4	6834	63681233	0.947901	0.658	0.712
NM_013932	11	15	234	259	Trim32	chr4	3184	65274199	0.805861	0.537	0.475
NM_134469	33	18	50	43	Tle1	chr4	4308	71830228	0.947901	0.475	0.538
NM_178896	21	12	7	20	3110001D03Rik	chr4	769	74923396	0.801072	0.417	0.259
NM_001081490	21	13	20	18	Sh3gl2	chr4	2817	85034761	0.947901	0.564	0.526
NM_028013	26	15	66	49	Mllt3	chr4	4793	87452182	0.947901	0.621	0.574
NM_148948	11	19	75	76	Mysm1	chr4	7420	94637217	0.859534	0.607	0.497
NM_009984	13	18	32	49	Dock7	chr4	7043	98756219	0.604997	0.553	0.395
NM_007960	25	15	43	31	Pgm2	chr4	2302	99659569	0.947901	0.61	0.581
NM_001081191	56	50	154	95	Jak1	chr4	5299	100866411	0.947901	0.628	0.618
NM_145139	135	114	77	44	Dnajc6	chr4	5225	101311888	0.947901	0.645	0.636
NM_145422	27	28	56	46	Leprot	chr4	1827	101331621	0.710828	0.662	0.549
NM_016903	106	97	39	34	Pde4b	chr4	4371	102277818	0.947901	0.579	0.534
NM_017477	42	37	41	22	Ppap2b	chr4	3162	104829988	0.279798	0.417	0.651
NM_011431	20	11	18	17	Dhcr24	chr4	4028	106260236	0.947901	0.557	0.514
NM_175276	11	15	21	8	Ttc4	chr4	2085	106351406	0.947901	0.692	0.724
NM_013908	27	24	40	27	Ssbp3	chr4	3211	106720893	0.947901	0.61	0.597
NM_198412	20	11	23	32	Lrp8	chr4	7674	107546208	0.915297	0.513	0.418
NM_019771	174	137	67	59	0610037L13Rik	chr4	1551	107569729	0.947901	0.521	0.532
NM_053090	18	24	35	30	Btf3l4	chr4	3111	108503162	0.945732	0.619	0.538
NM_027270	40	37	154	132	Rab3b	chr4	3320	108615798	0.947901	0.569	0.538
NM_134040	20	18	24	35	Faf1	chr4	4452	109635718	0.805861	0.545	0.407
NM_026225	39	21	367	285	Prdx1	chr4	1417	116371925	0.194319	0.488	0.563
NM_011807	22	21	22	23	Prnpip1	chr4	1576	117287813	0.871565	0.591	0.489
NM_021449	14	19	17	16	Ebna1bp2	chr4	2941	118299899	0.464992	0.341	0.515
NM_013494	25	12	24	42	Ybx1	chr4	2191	118954774	0.381503	0.503	0.364
NM_010103	51	38	46	46	Ctps	chr4	2681	120225599	0.947901	0.534	0.5
NM_011991	15	10	271	34	Ppie	chr4	1168	122807685	0.280466	0.817	0.889
NM_198303	47	31	30	20	Hpcal4	chr4	4652	122870503	0.947901	0.568	0.6
NM_001003908	22	21	95	153	Ndufs5	chr4	540	123395359	0.065669	0.517	0.383
NM_025785	16	14	17	14	Rragc	chr4	2621	123612913	0.947901	0.614	0.548
NM_001080932	24	28	16	13	Utp11l	chr4	953	124370756	0.947901	0.469	0.552
NM_023047	22	13	24	18	1810007P19Rik	chr4	2152	125766520	0.945732	0.646	0.571

NM_007930	139	181	54	31	Lsm10	chr4	869	125775321	0.947901	0.597	0.635
NM_001081419	23	18	16	19	Thrap3	chr4	4350	125863711	0.865057	0.555	0.457
NM_001038619	82	61	77	46	Trim62	chr4	3216	128586284	0.92412	0.569	0.626
NM_001080797	27	28	43	42	Yars	chr4	2874	128895834	0.947901	0.5	0.506
NM_027922	43	31	37	39	Rbbp4	chr4	4407	128999107	0.744899	0.654	0.487
NM_021356	20	20	126	124	Zbtb8os	chr4	660	129024165	0.947901	0.527	0.504
NM_007861	84	102	14	22	Eif3s2	chr4	1114	129277677	0.866345	0.5	0.389
NM_001025192	73	63	66	39	Ccdc28b	chr4	856	129299867	0.617761	0.541	0.629
NM_009931	59	47	89	44	Ptp4a2	chr4	2789	129526298	0.252235	0.494	0.669
NM_026695	48	47	12	14	Pef1	chr4	1551	129802314	0.947901	0.515	0.462
NM_133916	19	32	49	16	Fabp3	chr4	669	129992701	0.947901	0.724	0.754
NM_010243	24	9	17	11	Wdr57	chr4	1613	130067093	0.947901	0.581	0.607
NM_080285	28	15	120	65	Sdc3	chr4	4972	130379466	0.616107	0.543	0.649
NM_053272	39	31	670	469	Stmn1	chr4	965	134029551	0.844976	0.605	0.588
NM_009818	36	21	15	43	2410166I05Rik	chr4	1836	134088122	0.563807	0.414	0.259
NM_010201	22	15	33	30	Tmem57	chr4	3843	134386471	0.947901	0.486	0.524
NM_183171	38	32	117	119	Tmem50a	chr4	1117	134465572	0.947901	0.471	0.496
NM_009940	55	52	35	16	Syf2	chr4	1276	134492955	0.296511	0.458	0.686
NM_027148	21	20	64	42	Rcan3	chr4	5062	134981360	0.947901	0.598	0.604
NM_028044	82	62	35	14	Fuca1	chr4	2503	135495156	0.104358	0.442	0.714
NM_172442	23	11	36	26	1110049F12Rik	chr4	1554	135531634	0.947901	0.551	0.581
NM_146087	42	35	20	39	Aof2	chr4	2999	136148893	0.482412	0.506	0.339
NM_138587	76	77	162	106	Sdhb	chr4	1091	140534966	0.750419	0.554	0.604
NM_007759	9	16	21	23	Casp9	chr4	3896	141371691	0.444564	0.719	0.477
NM_007805	14	13	103	74	Pdpn	chr4	1769	142857383	0.617974	0.473	0.582
NM_026170	27	24	75	130	Park7	chr4	878	150281248	0.947901	0.405	0.366
NM_172920	16	14	90	83	Camta1	chr4	8423	150518187	0.944988	0.577	0.52
NM_001025947	25	25	125	117	Acot7	chr4	1452	151580897	0.922218	0.556	0.517
NM_025648	7	26	24	20	Rpl22	chr4	2081	151707487	0.947901	0.519	0.545
NM_138583	21	20	41	22	Lrrc47	chr4	3395	153394423	0.719164	0.51	0.651
NM_013716	14	13	25	28	Wdr8	chr4	1600	153530274	0.947901	0.486	0.472
NM_016856	56	29	78	66	1200015A19Rik	chr4	1756	153531617	0.43881	0.426	0.542
NM_001007571	82	91	13	16	Gnb1	chr4	2834	154931629	0.75561	0.587	0.448
NM_015744	24	16	23	33	Nadk	chr4	2943	154961491	0.947901	0.471	0.411
NM_016772	75	55	18	14	Ccnl2	chr4	2442	155197679	0.947901	0.474	0.563
NM_019936	125	91	104	140	Aurkaip1	chr4	793	155205802	0.465109	0.519	0.426
NM_027652	56	102	25	16	BC002216	chr4	2662	155243426	0.749267	0.444	0.61
NM_016748	31	27	10	21	Cpsf3l	chr4	2347	155261018	0.58691	0.543	0.323
NM_007798	35	36	48	48	Sdf4	chr4	5174	155387349	0.602323	0.648	0.5
NM_025869	17	13	15	37	9430015G10Rik	chr4	2539	155499572	0.947901	0.296	0.288
NM_029653	24	9	324	236	Agrn	chr4	6866	155559234	0.760279	0.528	0.579
NM_010047	19	15	26	14	Klhl17	chr4	2679	155603699	0.833193	0.515	0.65
NM_172380	35	22	64	64	Akap9	chr5	12105	4079780	0.947901	0.473	0.5
NM_026365	21	21	18	21	Pftk1	chr5	4911	5195357	0.860132	0.6	0.462
NM_031156	23	21	133	101	Gnai1	chr5	3193	17797427	0.780086	0.481	0.568
NM_146100	17	9	57	55	Psmc2	chr5	1782	21308907	0.805861	0.6	0.509
NM_029626	39	25	129	171	Reln	chr5	11702	21516989	0.281941	0.574	0.43
NM_010825	39	22	83	86	Srpk2	chr5	6436	23030336	0.947901	0.526	0.491
NM_020295	14	12	39	43	Drctnnb1a	chr5	5652	23497592	0.947901	0.429	0.476
NM_029665	15	12	34	20	Abcf2	chr5	2488	24076962	0.825272	0.519	0.63
NM_010317	46	18	79	100	Dpp6	chr5	3842	28036063	0.433765	0.571	0.441
NM_001025245	22	6	22	22	Lmbr1	chr5	4949	29704873	0.947901	0.538	0.5

NM_008143	17	17	72	38	Ube3c	chr5	5033	30001600	0.381503	0.52	0.655
NM_172529	11	15	39	29	Hadhb	chr5	1963	30507499	0.47658	0.389	0.574
NM_010716	14	18	103	165	D5Wsu178e	chr5	6769	30593923	0.947901	0.354	0.384
NM_026148	17	13	51	30	Dpysl5	chr5	5077	31101004	0.947901	0.629	0.63
NM_027898	54	141	26	29	1110039B18Rik	chr5	2762	31179073	0.543527	0.676	0.473
NM_021554	19	25	15	23	Ppm1g	chr5	2165	31508582	0.947901	0.445	0.395
NM_023331	102	83	22	11	Xab1	chr5	1847	31813339	0.947901	0.739	0.667
NM_001081279	22	9	29	22	Ppp1cb	chr5	4024	32794133	0.947901	0.524	0.569
NM_020590	32	26	85	145	Pisd	chr5	2150	33117087	0.165895	0.496	0.37
NM_028185	49	60	19	20	Fgfr3	chr5	4214	34078898	0.947901	0.577	0.487
NM_011941	17	25	42	21	Letm1	chr5	3480	34125229	0.947901	0.658	0.667
NM_177301	14	27	88	55	Rnf4	chr5	2936	34693939	0.738849	0.513	0.615
NM_001039347	33	29	73	74	Lrpap1	chr5	2922	35440228	0.86501	0.554	0.497
NM_178610	21	13	34	33	Afap1	chr5	6617	36345300	0.947901	0.552	0.507
NM_010787	73	50	176	145	Mrfap1	chr5	1579	37186134	0.947901	0.519	0.548
NM_008449	17	22	24	11	Ppp2r2c	chr5	4066	37318780	0.866345	0.584	0.686
NM_008627	16	14	21	15	Stx18	chr5	1562	38527588	0.947901	0.531	0.583
NM_010306	60	50	130	135	Nsg1	chr5	2140	38528777	0.347661	0.588	0.491
NM_146145	86	51	32	13	A230054D04Rik	chr5	10646	42223668	0.86501	0.593	0.711
NM_019657	27	32	67	54	Qdpr	chr5	1326	45841346	0.750419	0.444	0.554
NM_026490	21	15	31	19	Ugdh	chr5	2461	65818760	0.947901	0.59	0.62
NM_010687	29	23	21	10	Apbb2	chr5	3362	66934804	0.947901	0.727	0.677
NM_027352	27	44	428	560	Uchl1	chr5	995	67078364	0.490713	0.485	0.433
NM_153058	97	64	82	73	Limch1	chr5	6142	67447645	0.947901	0.528	0.529
NM_145958	14	11	30	14	Gabra4	chr5	4123	72024918	0.86501	0.578	0.682
NM_030145	96	110	29	11	Fryl	chr5	11488	73411432	0.947901	0.686	0.725
NM_175462	18	14	34	65	Ociad1	chr5	1505	73704770	0.865502	0.42	0.343
NM_019564	26	26	54	42	Ociad2	chr5	2201	73727159	0.753949	0.458	0.563
NM_001001980	56	50	20	17	Dcun1d4	chr5	4063	73949359	0.947901	0.636	0.541
NM_181796	59	64	48	28	Sgcb	chr5	3727	74026509	0.633314	0.444	0.632
NM_027168	60	31	53	19	Usp46	chr5	3225	74412735	0.653115	0.56	0.736
NM_016961	76	55	18	12	Kit	chr5	5132	76049338	0.947901	0.54	0.6
NM_138721	71	48	34	36	Exoc1	chr5	3395	76997060	0.947901	0.519	0.486
NM_026068	254	86	121	92	Paics	chr5	2394	77396499	0.947901	0.585	0.568
NM_026347	21	26	65	82	Rufy3	chr5	4036	89070260	0.762186	0.518	0.442
NM_026374	36	37	32	19	Slc4a4	chr5	7492	89467557	0.80299	0.507	0.627
NM_013886	18	9	16	24	Ankrd17	chr5	10066	90719989	0.672243	0.578	0.4
NM_001081049	30	22	34	29	Rchy1	chr5	1967	92386624	0.947901	0.48	0.54
NM_145527	32	28	14	19	G3bp2	chr5	4276	92493477	0.947901	0.491	0.424
NM_010513	19	18	124	198	Sep11	chr5	5123	93600514	0.785646	0.434	0.385
NM_010308	152	105	295	331	Ccni	chr5	2348	93616823	0.355031	0.588	0.471
NM_009157	22	17	117	83	Hnrpd	chr5	6663	100387431	0.947901	0.553	0.585
NM_008403	14	13	17	10	Enoph1	chr5	1806	100496864	0.846101	0.485	0.63
NM_025486	17	12	40	38	BC062109	chr5	3100	100524700	0.753949	0.65	0.513
NM_026896	19	18	255	338	Mrps18c	chr5	526	101228579	0.947901	0.444	0.43
NM_025280	16	15	48	72	Mapk10	chr5	7203	103425720	0.895717	0.462	0.4
NM_013551	13	13	46	30	Sparcl1	chr5	2797	104542469	0.695676	0.492	0.605
NM_026242	41	38	23	9	Spp1	chr5	1410	104868369	0.947901	0.629	0.719
NM_021099	34	29	10	29	Brdt	chr5	4710	107815124	0.915297	0.346	0.256
NM_001004164	16	10	25	14	Dgkq	chr5	4612	109089569	0.465109	0.385	0.641
NM_019694	50	26	34	22	Chfr	chr5	3144	110599932	0.947901	0.674	0.607
NM_175386	17	15	90	79	D5Ertd585e	chr5	5241	110685457	0.947901	0.581	0.533

NM_023403	94	83	40	21	Pgam5	chr5	2039	110698722	0.947901	0.645	0.656
NM_008255	47	42	38	53	Galnt9	chr5	1237	111049823	0.697733	0.299	0.418
NM_013535	104	95	44	35	Pitpnb	chr5	2761	111816839	0.562014	0.41	0.557
NM_008410	160	127	131	84	Mn1	chr5	6128	111884244	0.8481	0.535	0.609
NM_010688	20	14	206	197	Asphd2	chr5	1604	112814505	0.947901	0.493	0.511
NM_029884	21	25	77	48	Sez6l	chr5	6073	112901830	0.677707	0.524	0.616
NM_026240	15	25	15	11	Kctd10	chr5	3058	114820146	0.555395	0.342	0.577
NM_010404	16	12	42	35	Ube3b	chr5	5191	114869516	0.647521	0.682	0.545
NM_026759	68	54	24	23	Triap1	chr5	1049	115792889	0.947901	0.509	0.511
NM_010358	66	49	17	27	Prkab1	chr5	2051	116474109	0.602323	0.583	0.386
NM_172252	76	65	155	166	Wsb2	chr5	2438	117820820	0.947901	0.511	0.483
NM_028007	20	15	68	33	Fbxo21	chr5	3909	118458056	0.833193	0.583	0.673
NM_008379	45	50	50	39	Rph3a	chr5	4143	121459435	0.942332	0.627	0.562
NM_026530	34	20	409	293	Rpl6	chr5	1219	121658575	0.947901	0.602	0.583
NM_011828	18	24	25	23	Aldh2	chr5	2207	122030701	0.750419	0.667	0.521
NM_029657	34	30	19	25	Pptc7	chr5	4611	122770923	0.947901	0.515	0.432
NM_010316	290	184	196	246	Vps29	chr5	991	122813160	0.381503	0.527	0.443
NM_010362	41	50	43	20	Atp2a2	chr5	4353	122939251	0.621704	0.522	0.683
NM_021295	13	18	85	63	Anapc5	chr5	2760	123257333	0.947901	0.594	0.574
NM_178797	15	14	42	26	Rnf34	chr5	1980	123318173	0.947901	0.622	0.618
NM_029931	41	25	25	11	Rhof	chr5	2143	123568301	0.947901	0.656	0.694
NM_001083897	23	14	25	20	Sbno1	chr5	10071	124860170	0.947901	0.633	0.556
NM_172739	29	18	10	21	Eif2b1	chr5	1938	125028471	0.601949	0.5	0.323
NM_026826	196	245	12	22	Zfp664	chr5	4139	125368177	0.947901	0.345	0.353
NM_001048177	26	20	69	55	Aacs	chr5	3175	125996636	0.86501	0.632	0.556
NM_026160	37	37	14	27	Gbas	chr5	2115	130263461	0.947901	0.425	0.341
NM_010492	34	32	21	9	Psph	chr5	1482	130284924	0.770114	0.529	0.7
NM_174998	25	19	570	485	Cct6a	chr5	2189	130321475	0.744899	0.583	0.54
NM_029934	22	15	197	79	Chchd2	chr5	910	130363139	0.947901	0.741	0.714
NM_013785	29	31	16	18	0610007L01Rik	chr5	3607	130718496	0.80299	0.31	0.471
NM_172610	64	42	92	69	Sbds	chr5	1495	130724203	0.947901	0.547	0.571
NM_207217	41	24	32	22	Gtf2i	chr5	4392	134771437	0.844976	0.493	0.593
NM_025553	16	17	19	19	Stx1a	chr5	2071	135526106	0.947901	0.538	0.5
NM_008617	171	203	82	101	Mdh2	chr5	1297	136266018	0.947901	0.457	0.448
NM_029017	22	13	86	55	Ywhag	chr5	3510	136384468	0.947901	0.585	0.61
NM_023290	19	12	110	130	Rabl5	chr5	911	137389080	0.947901	0.456	0.458
NM_022417	35	18	48	54	Fis1	chr5	775	137442092	0.858549	0.404	0.471
NM_008139	144	107	18	18	Ars2	chr5	3039	137737068	0.379948	0.299	0.5
NM_010799	29	14	48	50	Prkar1b	chr5	2583	139584492	0.947901	0.554	0.49
NM_178603	16	17	34	41	Zfand2a	chr5	3115	139960279	0.947901	0.5	0.453
NM_177093	19	24	14	19	Eif3s9	chr5	2966	140916960	0.947901	0.373	0.424
NM_023505	17	9	12	18	Zfp12	chr5	4271	144008567	0.27518	0.679	0.4
NM_007516	78	63	279	203	Rac1	chr5	2284	144278049	0.72737	0.525	0.579
NM_010298	19	15	56	58	2810453I06Rik	chr5	2215	144323843	0.26307	0.671	0.491
NM_008095	17	23	298	48	Jtv1	chr5	1072	144665522	0.248764	0.781	0.861
NM_019650	50	32	78	36	Lmtk2	chr5	8114	144948158	0.525209	0.5	0.684
NM_138593	24	12	39	38	Arpc1a	chr5	1588	145869289	0.947901	0.57	0.506
NM_026472	55	44	18	22	Ptcd1	chr5	3014	145915988	0.947901	0.415	0.45
NM_018741	56	37	266	321	Atp5j2	chr5	496	145952427	0.792244	0.486	0.453
NM_013559	102	84	19	9	Zkscan5	chr5	3134	145982482	0.750419	0.51	0.679
NM_008078	59	48	451	466	Pomp	chr5	734	148687248	0.191221	0.441	0.492
NM_010629	124	92	14	13	Uspl1	chr5	3125	150024941	0.883593	0.667	0.519

NM_009582	13	15	98	76	Hsp110	chr5	3478	150431890	0.947901	0.548	0.563
NM_201354	9	27	24	10	Rfc3	chr5	1215	152453734	0.296511	0.429	0.706
NM_027350	117	99	55	36	Col1a2	chr6	5143	4490674	0.525209	0.435	0.604
NM_024174	26	24	39	13	Casd1	chr6	3760	4592497	0.150651	0.463	0.75
NM_024193	19	14	130	134	Dync1i1	chr6	2579	5977485	0.844976	0.446	0.492
NM_001081079	33	33	85	100	Shfm1	chr6	491	6508473	0.945732	0.511	0.459
NM_028132	114	73	30	44	Tac1	chr6	1034	7512646	0.947901	0.42	0.405
NM_033582	21	18	27	28	Ica1	chr6	2090	8704622	0.947901	0.515	0.491
NM_013625	43	46	46	36	B630005N14Rik	chr6	4147	13576079	0.947901	0.621	0.561
NM_024197	52	53	161	233	Capza2	chr6	2015	17615540	0.573288	0.519	0.409
NM_033585	21	18	105	61	Cttnbp2	chr6	5931	18383862	0.947901	0.651	0.633
NM_033590	19	18	104	100	D6Wsu176e	chr6	2739	22293280	0.947901	0.497	0.51
NM_023526	48	31	14	22	Ptprz1	chr6	8068	23001998	0.805861	0.535	0.389
NM_019746	53	46	464	461	Ndufa5	chr6	549	24477279	0.947901	0.511	0.502
NM_028273	40	22	11	27	Snd1	chr6	3482	28657064	0.947901	0.378	0.289
NM_011035	39	51	76	74	Calu	chr6	3216	29324688	0.947901	0.477	0.507
NM_026614	90	86	137	119	Atp6v1f	chr6	635	29420070	0.58647	0.434	0.535
NM_013880	24	14	28	13	Zc3hc1	chr6	1847	30337477	0.947901	0.719	0.683
NM_145131	19	20	19	6	Tsga14	chr6	3393	30610911	0.947901	0.707	0.76
NM_023409	50	32	31	25	Chchd3	chr6	1432	32742393	0.858672	0.433	0.554
NM_001033174	14	17	26	6	Akr1b3	chr6	1402	34267418	0.947901	0.813	0.813
NM_010885	86	91	33	20	Slc13a4	chr6	3422	35257495	0.573356	0.821	0.623
NM_175933	29	14	65	50	Ptn	chr6	2021	36693436	0.947901	0.529	0.565
NM_001077495	39	60	34	29	Trim24	chr6	4053	37916057	0.947901	0.607	0.54
NM_009787	11	15	14	20	Pdia4	chr6	2610	47756610	0.947901	0.423	0.412
NM_008633	52	41	27	20	Krba1	chr6	3986	48365778	0.261888	0.269	0.574
NM_172416	29	19	397	315	Atp6v0e2	chr6	1786	48490048	0.947901	0.56	0.558
NM_007531	23	20	45	28	Tmem176b	chr6	1659	48790389	0.947901	0.633	0.616
NM_026737	129	152	54	15	Tra2a	chr6	1839	49213932	0.947901	0.774	0.783
NM_016744	16	10	194	126	Npy	chr6	561	49777509	0.947901	0.6	0.606
NM_028388	99	95	14	21	Dfna5h	chr6	2133	50211741	0.712972	0.6	0.4
NM_138671	41	46	14	14	Cbx3	chr6	1851	51432521	0.431606	0.214	0.5
NM_134094	149	147	155	112	Tax1bp1	chr6	2970	52716072	0.562014	0.671	0.581
NM_013602	35	14	55	76	Gars	chr6	2390	55029252	0.23556	0.592	0.42
NM_011958	23	11	66	55	Adcyap1r1	chr6	6176	55447272	0.947901	0.538	0.545
NM_153057	43	36	19	22	Neurod6	chr6	2154	55628507	0.844976	0.583	0.463
NM_027654	35	30	25	15	Kbtbd2	chr6	2799	56729004	0.947901	0.56	0.625
NM_019840	22	16	44	12	Nt5c3	chr6	1568	56838393	0.947901	0.731	0.786
NM_011119	13	13	18	25	AW146242	chr6	2906	57724731	0.947901	0.48	0.419
NM_020610	17	18	20	33	Gng12	chr6	4133	66970959	0.895717	0.484	0.377
NM_019837	53	50	17	14	Rnf181	chr6	1397	72309941	0.943216	0.649	0.548
NM_023858	15	17	109	36	Tgoln1	chr6	5025	72565521	0.277199	0.569	0.752
NM_008741	189	148	36	21	Suclg1	chr6	1211	73225371	0.908076	0.549	0.632
NM_173757	11	17	50	61	Ctnna2	chr6	4046	77555214	0.176665	0.667	0.45
NM_008792	54	34	31	24	Mrpl19	chr6	5016	81915748	0.947901	0.583	0.564
NM_008740	17	9	107	107	Pole4	chr6	1694	82597767	0.896765	0.571	0.5
NM_015781	35	19	35	12	Sema4f	chr6	4067	82867468	0.459791	0.516	0.745
NM_008691	50	62	51	28	Paip2b	chr6	3323	83757513	0.750419	0.5	0.646
NM_010945	29	21	15	18	Zfml	chr6	6344	83933978	0.910787	0.356	0.455
NM_008825	24	18	32	19	Sfxn5	chr6	3646	85217872	0.947901	0.667	0.627
NM_017373	15	12	56	40	Cct7	chr6	1879	85417099	0.780086	0.484	0.583
NM_144898	18	10	132	59	Snrpg	chr6	393	86328850	0.202927	0.476	0.691

NM_001005748	18	12	42	35	Tia1	chr6	4418	86380250	0.947901	0.561	0.545
NM_010914	349	366	87	63	2610209M04Rik	chr6	836	86625376	0.783653	0.467	0.58
NM_177906	28	5	111	98	Aak1	chr6	19363	86953034	0.947901	0.52	0.531
NM_053134	9	19	39	29	Copg	chr6	4014	87862991	0.947901	0.532	0.574
NM_008173	56	38	36	23	C130022K22Rik	chr6	1757	91849412	0.86501	0.719	0.61
NM_016978	20	19	89	108	Mrps25	chr6	6076	92128732	0.945732	0.506	0.452
NM_133800	74	49	26	36	Magi1	chr6	4969	93765782	0.828443	0.538	0.419
NM_025987	15	10	11	30	Arl6ip5	chr6	1339	97182586	0.602323	0.455	0.268
NM_133666	110	110	51	44	Trnt1	chr6	2207	106729385	0.712511	0.4	0.537
NM_033595	20	20	27	31	Crbn	chr6	4051	106744229	0.947901	0.424	0.466
NM_033591	19	18	7	39	Edem1	chr6	5858	108805532	0.947901	0.1	0.152
NM_144870	214	187	86	86	Lhfpl4	chr6	4782	113121481	0.604997	0.596	0.5
NM_033583	19	18	21	27	Jagn1	chr6	1133	113397498	0.947901	0.5	0.438
NM_028806	42	32	39	33	Tmem111	chr6	1958	113481496	0.947901	0.511	0.542
NM_026004	19	7	25	27	Sec13	chr6	1301	113686422	0.915297	0.563	0.481
NM_145525	18	16	19	17	Atp2b2	chr6	7060	113767208	0.947901	0.448	0.528
NM_019703	130	104	15	17	Slc6a1	chr6	4153	114265796	0.947901	0.526	0.469
NM_178708	19	15	30	20	Mkrn2	chr6	2241	115563383	0.947901	0.613	0.6
NM_019632	39	54	20	38	Adipor2	chr6	3963	119307261	0.487509	0.559	0.345
NM_033575	19	18	21	16	Erc1	chr6	8760	119774530	0.947901	0.556	0.568
NM_028017	18	21	112	126	Atp6v1e1	chr6	1219	120772627	0.508205	0.548	0.471
NM_026703	78	68	17	14	Slc6a13	chr6	2150	121287335	0.602323	0.739	0.548
NM_016743	82	98	22	19	Iqsec3	chr6	6840	121363302	0.770622	0.375	0.537
NM_011069	15	14	18	15	Phc1	chr6	3683	122284163	0.947901	0.481	0.545
NM_020271	51	24	56	30	Pex5	chr6	3074	124346904	0.947901	0.674	0.651
NM_021524	41	18	56	73	Clstn3	chr6	3937	124400730	0.947901	0.455	0.434
NM_026441	17	16	54	35	Phb2	chr6	1360	124664940	0.947901	0.535	0.607
NM_033581	21	18	82	63	Grcc10	chr6	568	124690607	0.947901	0.523	0.566
NM_033592	19	18	29	18	Eno2	chr6	2656	124718323	0.947901	0.645	0.617
NM_001042623	50	54	292	252	Tpi1	chr6	1594	124764401	0.947901	0.543	0.537
NM_020569	51	75	73	18	Ccnd2	chr6	5772	127096144	0.947901	0.804	0.802
NM_033577	13	14	48	38	Gabarapl1	chr6	1773	129491515	0.947901	0.552	0.558
NM_023456	39	26	34	58	Ptpro	chr6	5075	137410282	0.859246	0.431	0.37
NM_025939	38	27	10	15	BC027061	chr6	2155	142310369	0.947901	0.485	0.4
NM_028024	14	15	290	220	Ldhb	chr6	1324	142456412	0.806139	0.608	0.569
NM_010864	36	22	12	22	Cmas	chr6	1687	142723698	0.324299	0.573	0.353
NM_011482	31	33	53	0	Tuba3b	chr6	1573	145569637	0.783653	0.962	1
NM_008786	30	16	19	19	Mlstd1	chr6	3397	148129750	0.947901	0.517	0.5
NM_009122	32	16	65	58	Leng4	chr7	2880	3644365	0.947901	0.595	0.528
NM_017400	20	17	32	31	Ttyh1	chr7	3888	4086866	0.947901	0.48	0.508
NM_026073	57	68	45	44	Syt5	chr7	1764	4497845	0.805861	0.636	0.506
NM_011201	16	13	38	37	Rps5	chr7	763	13507687	0.786421	0.426	0.507
NM_010817	78	61	22	16	Napa	chr7	2332	16702582	0.780086	0.436	0.579
NM_025436	23	33	20	16	Mrg2	chr7	1781	16771572	0.947901	0.533	0.556
NM_011875	34	33	43	53	Sae1	chr7	1871	16952223	0.763276	0.532	0.448
NM_009093	193	178	25	17	Grlf1	chr7	8393	17146972	0.947901	0.617	0.595
NM_011290	533	352	284	118	Calm3	chr7	2233	17505000	0.947901	0.728	0.706
NM_011184	607	703	13	12	Rtn2	chr7	1947	19881163	0.696682	0.719	0.52
NM_172707	22	20	36	26	Gemin7	chr7	763	20150344	0.490713	0.423	0.581
NM_011874	35	29	489	309	Apoe	chr7	1266	20281675	0.727568	0.517	0.613
NM_022980	64	43	40	36	Atp1a3	chr7	4043	25785763	0.888353	0.602	0.526
NM_024499	27	29	69	49	Tmem91	chr7	874	26460101	0.947901	0.589	0.585

NM_026557	24	26	59	51	Pld3	chr7	2246	28330255	0.907627	0.593	0.536
NM_001039089	49	33	14	16	Psmc4	chr7	1436	28833969	0.947901	0.547	0.467
NM_001081086	20	32	19	10	Sirt2	chr7	1863	29573170	0.445503	0.395	0.655
NM_011300	903	1333	34	65	Hnrpl	chr7	2097	29605738	0.947901	0.341	0.343
NM_009091	65	48	44	39	Ech1	chr7	1138	29615050	0.947901	0.577	0.53
NM_177242	17	16	48	84	Eif3s12	chr7	773	29765513	0.562731	0.5	0.364
NM_133692	22	22	18	29	Psmd8	chr7	1529	29965339	0.329905	0.667	0.383
NM_080853	32	18	74	90	Zfp260	chr7	3851	30892620	0.947901	0.475	0.451
NM_018853	160	409	40	38	Capns1	chr7	1461	30977190	0.947901	0.459	0.513
NM_019570	16	11	105	76	Clip3	chr7	3300	31092940	0.915297	0.528	0.58
NM_148930	32	14	391	263	Aplp1	chr7	2389	31220142	0.866345	0.557	0.598
NM_009009	55	43	19	32	Snx26	chr7	4415	31317857	0.820336	0.5	0.373
NM_021305	21	16	61	46	Psenen	chr7	670	31347416	0.947901	0.6	0.57
NM_026434	19	23	31	23	U2af1l4	chr7	864	31349764	0.863684	0.477	0.574
NM_001081247	13	16	104	47	Wbp7	chr7	8457	31361794	0.347816	0.529	0.689
NM_025587	32	28	22	65	Gramd1a	chr7	2681	31927435	0.947901	0.277	0.253
NM_020012	99	77	156	139	Gpi1	chr7	1982	35015179	0.92262	0.566	0.529
NM_027541	15	13	13	18	Pepd	chr7	1876	35706457	0.636108	0.633	0.419
NM_023281	132	95	291	260	Pdcd5	chr7	642	36431168	0.947901	0.535	0.528
NM_133704	12	13	32	97	Pop4	chr7	1214	39051169	0.947901	0.245	0.248
NM_026147	76	17	31	25	Etfb	chr7	835	50707734	0.947901	0.505	0.554
NM_009255	136	101	362	635	Rps11	chr7	611	52379722	0.947901	0.359	0.363
NM_145516	68	40	197	272	Rpl13a	chr7	803	52381174	0.067764	0.526	0.42
NM_023537	37	28	19	44	Pih1d1	chr7	1101	52413067	0.805861	0.414	0.302
NM_007453	23	30	219	94	Ftl1	chr7	986	52713627	0.602323	0.758	0.7
NM_009001	49	43	20	20	Emp3	chr7	695	53174806	0.805861	0.636	0.5
NM_019708	46	62	24	18	Nomo1	chr7	4129	53338625	0.947901	0.544	0.571
NM_029971	27	32	20	115	Ldha	chr7	1681	54110529	0.289667	0.261	0.148
NM_008947	37	28	18	20	Tsg101	chr7	1832	54144575	0.724675	0.636	0.474
NM_008910	25	27	14	29	Prmt3	chr7	2495	57112745	0.459791	0.577	0.326
NM_178639	30	15	137	74	Slc17a6	chr7	4078	58877526	0.947901	0.64	0.649
NM_016891	37	31	63	38	Luzp2	chr7	5274	62090617	0.540466	0.452	0.624
NM_133485	38	29	43	24	Herc2	chr7	15363	63484678	0.523991	0.455	0.642
NM_029763	11	15	38	21	Gabra5	chr7	2674	64744509	0.385288	0.385	0.644
NM_145417	12	14	25	16	Apba2	chr7	3334	71898333	0.947901	0.643	0.61
NM_175498	18	20	215	145	Tm2d3	chr7	1611	72846488	0.301202	0.5	0.597
NM_181650	13	22	11	22	Snrpa1	chr7	1113	73219405	0.575419	0.53	0.333
NM_025517	42	37	16	14	Igf1r	chr7	11978	75372391	0.947901	0.514	0.533
NM_008923	36	29	23	7	Slco3a1	chr7	4697	81463360	0.602323	0.559	0.767
NM_011886	45	32	83	74	Mrpl46	chr7	1173	85927726	0.947901	0.551	0.529
NM_017374	76	94	44	11	Mrps11	chr7	880	85937632	0.525209	0.659	0.8
NM_025498	33	22	77	90	Hddc3	chr7	860	87490595	0.648107	0.555	0.461
NM_026440	14	14	131	118	Ap3b2	chr7	3816	88621975	0.947901	0.557	0.526
NM_030564	28	17	19	17	2610204K14Rik	chr7	1423	88912439	0.947901	0.545	0.528
NM_025390	26	80	59	42	Btbd1	chr7	3020	88941903	0.416102	0.429	0.584
NM_012052	421	296	40	27	Hdgfrp3	chr7	5868	89029429	0.947901	0.667	0.597
NM_027324	148	126	24	20	Sh3gl3	chr7	1597	89455314	0.947901	0.541	0.545
NM_023248	81	67	104	94	Mesdc2	chr7	4235	91049795	0.947901	0.531	0.525
NM_178607	20	20	43	41	2210412D01Rik	chr7	2252	91262790	0.945732	0.577	0.512
NM_153781	16	29	225	309	Arnt2	chr7	6006	91510066	0.803541	0.378	0.421
NM_011871	11	16	54	25	Tmem135	chr7	3502	96487105	0.617974	0.525	0.684
NM_013725	587	1050	43	33	Dlg2	chr7	7252	99435068	0.947901	0.512	0.566

NM_016784	14	11	16	33	Ccdc90b	chr7	1212	99730521	0.134726	0.647	0.327
NM_013872	56	34	406	324	Ndufc2	chr7	613	104548782	0.301202	0.484	0.556
NM_175092	21	11	17	9	1810020D17Rik	chr7	866	104706668	0.918799	0.541	0.654
NM_009143	181	141	58	64	Pak1	chr7	3081	105059698	0.947901	0.433	0.475
NM_152824	72	59	27	19	Mtap6	chr7	4962	106464322	0.85781	0.486	0.587
NM_013663	17	11	403	295	Rps3	chr7	1879	106627955	0.947901	0.587	0.577
NM_025321	87	104	11	16	Pold3	chr7	3002	107270002	0.947901	0.5	0.407
NM_016858	42	29	17	8	Chchd8	chr7	834	107687631	0.805861	0.523	0.68
NM_011563	423	462	94	39	Mrpl48	chr7	968	107731629	0.601754	0.582	0.707
NM_175400	22	10	141	198	Rab6	chr7	3403	107787922	0.947901	0.413	0.416
NM_008882	26	0	109	61	B930006L02Rik	chr7	6610	107987989	0.199736	0.51	0.641
NM_182999	31	18	30	21	2400001E08Rik	chr7	1075	109060159	0.940278	0.5	0.588
NM_175549	19	10	66	33	Apbb1	chr7	2593	112707142	0.947901	0.615	0.667
NM_009048	16	11	138	161	Eif3s5	chr7	1252	116085082	0.909636	0.552	0.462
NM_028230	18	16	61	47	Tub	chr7	5941	116173864	0.947901	0.576	0.565
NM_009155	80	71	50	31	Rpl27a	chr7	1112	116665590	0.947901	0.652	0.617
NM_019535	22	17	42	39	Tmem9b	chr7	1685	116879591	0.947901	0.444	0.519
NM_016807	29	17	31	37	Nrip3	chr7	3913	116924916	0.947901	0.486	0.456
NM_133231	17	8	190	162	Eif4g2	chr7	7760	118213890	0.915297	0.5	0.54
NM_011975	30	16	40	33	Btbd10	chr7	2563	120459487	0.947901	0.588	0.548
NM_025901	22	11	75	86	Copb1	chr7	3315	121384190	0.405087	0.27	0.466
NM_026799	28	28	64	117	Psma1	chr7	1255	121413296	0.918799	0.405	0.354
NM_011967	105	92	137	111	Rps13	chr7	521	123475904	0.698463	0.643	0.552
NM_133735	39	55	282	170	Rps15a	chr7	5158	125258362	0.780086	0.547	0.624
NM_026308	55	25	103	81	Coq7	chr7	871	125676771	0.947901	0.514	0.56
NM_008997	20	28	24	7	9030624J02Rik	chr7	3101	125981673	0.805861	0.648	0.774
NM_007452	29	31	47	60	Crym	chr7	1256	127333284	0.602323	0.576	0.439
NM_133648	21	10	174	128	Uqcrc2	chr7	1882	127802664	0.947901	0.603	0.576
NM_001081203	19	11	47	49	Mettl9	chr7	1850	128219741	0.947901	0.432	0.49
NM_008014	49	61	80	49	Dctn5	chr7	1517	129292531	0.474379	0.51	0.62
NM_207209	44	24	26	22	Prkcb1	chr7	8830	129711710	0.947901	0.492	0.542
NM_145452	22	16	52	40	Lcmt1	chr7	1369	130571668	0.842379	0.484	0.565
NM_019658	17	17	92	78	Tufm	chr7	1609	133634070	0.947901	0.517	0.541
NM_008983	17	12	22	14	Tbc1d10b	chr7	3614	134342926	0.792244	0.448	0.611
NM_008857	16	16	15	18	Vkorc1	chr7	764	135038937	0.603149	0.629	0.455
NM_008855	30	31	135	153	Tgfb1i1	chr7	3058	135395602	0.602323	0.366	0.469
NM_011254	32	31	57	41	Brwd2	chr7	4519	136774267	0.883593	0.514	0.582
NM_009079	14	13	51	30	Ate1	chr7	4727	137662877	0.947901	0.581	0.63
NM_008950	41	21	38	16	Plekha1	chr7	3321	138056322	0.744899	0.538	0.704
NM_017475	54	34	34	28	Htra1	chr7	2002	138128698	0.947901	0.5	0.548
NM_008945	20	23	24	14	Acadsb	chr7	3243	138568088	0.70756	0.462	0.632
NM_175445	28	23	44	29	Bub3	chr7	2055	138714444	0.851937	0.483	0.603
NM_025441	20	21	23	16	Oat	chr7	2155	139768020	0.947901	0.513	0.59
NM_024287	43	61	16	21	Ppp2r2d	chr7	2079	146074288	0.805861	0.552	0.432
NM_026517	49	32	31	27	Bet1l	chr7	1406	148039435	0.947901	0.553	0.534
NM_029457	11	14	54	44	Psmd13	chr7	1563	148084270	0.947901	0.507	0.551
NM_173347	17	18	34	40	Ifitm3	chr7	648	148195636	0.636367	0.603	0.459
NM_146081	14	11	37	19	Cend1	chr7	1659	148612464	0.561144	0.479	0.661
NM_021526	115	104	20	27	Igf2	chr7	4038	149836672	0.780086	0.547	0.426
NM_008973	63	56	48	55	Cttn	chr7	3029	151651884	0.780711	0.556	0.466
NM_008540	15	17	26	21	Lass4	chr8	3668	4525484	0.601486	0.75	0.553
NM_009295	121	113	22	27	Abhd13	chr8	5074	9991418	0.947901	0.5	0.449

NM_009333	103	73	53	36	Col4a1	chr8	6636	11312669	0.947901	0.557	0.596
NM_024270	19	36	11	19	Col4a2	chr8	6184	11448675	0.929497	0.464	0.367
NM_177102	43	30	14	12	Pcid2	chr8	2408	13100382	0.947901	0.559	0.538
NM_018861	42	27	110	34	Lamp1	chr8	2265	13173664	0.141878	0.617	0.764
NM_026211	77	78	18	18	Fbxo25	chr8	2008	13939801	0.947901	0.533	0.5
NM_019776	14	23	12	18	Agpat5	chr8	3829	18884057	0.750419	0.581	0.4
NM_146032	19	10	67	47	Vdac3	chr8	1345	23699244	0.830253	0.513	0.588
NM_028052	19	15	50	55	Ap3m2	chr8	3401	23914250	0.947901	0.53	0.476
NM_029682	33	24	36	37	Agpat6	chr8	3838	24301362	0.901101	0.422	0.493
NM_011506	38	31	47	37	Adam9	chr8	4003	26116729	0.947901	0.532	0.56
NM_030706	21	18	31	12	Tacc1	chr8	7695	26292620	0.947901	0.639	0.721
NM_023056	31	18	30	26	Hgsnat	chr8	2679	27078839	0.947901	0.457	0.536
NM_019790	36	24	69	62	Fnta	chr8	1881	27117721	0.947901	0.574	0.527
NM_011599	19	21	33	22	Dusp26	chr8	1747	32207301	0.947901	0.567	0.6
NM_011018	19	21	22	24	Rbm13	chr8	1857	32277035	0.813566	0.621	0.478
NM_020050	16	20	109	131	Ppp2cb	chr8	1836	34729872	0.947901	0.447	0.454
NM_031998	58	24	66	77	Ubxd6	chr8	2817	34745797	0.947901	0.443	0.462
NM_080557	44	39	191	168	Dctn6	chr8	926	35160287	0.783653	0.604	0.532
NM_175212	32	23	38	18	Mfhas1	chr8	6408	36739903	0.947901	0.71	0.679
NM_008541	21	17	154	80	Tusc3	chr8	1527	40068938	0.601754	0.528	0.658
NM_172752	15	10	109	69	Vps37a	chr8	6379	41636151	0.783653	0.509	0.612
NM_001033270	14	11	97	67	Mtmr7	chr8	3446	41692225	0.371307	0.408	0.591
NM_029068	17	12	30	23	BC035537	chr8	5479	46466670	0.780086	0.421	0.566
NM_198102	24	7	45	27	Sorbs2	chr8	6259	46911266	0.947901	0.6	0.625
NM_025285	38	30	23	9	1810047C23Rik	chr8	1556	47082155	0.947901	0.636	0.719
NM_001024206	33	47	104	91	Spcs3	chr8	3482	55610356	0.947901	0.484	0.533
NM_012030	26	19	32	68	Scrg1	chr8	700	59956198	0.433765	0.533	0.32
NM_009223	82	61	36	37	Clcn3	chr8	5238	63417370	0.750419	0.645	0.493
NM_020584	27	13	26	18	Cpe	chr8	2103	67098280	0.947901	0.676	0.591
NM_009415	127	107	52	80	Sc4mol	chr8	1824	67202518	0.947901	0.411	0.394
NM_009311	37	51	34	95	Atp6v1b2	chr8	2738	71636462	0.947901	0.261	0.264
NM_028711	665	306	145	59	Ndufa13	chr8	1266	72418107	0.159615	0.586	0.711
NM_001034964	84	52	20	29	Sf4	chr8	2287	72595806	0.783653	0.265	0.408
NM_009409	39	32	40	37	Rab3a	chr8	1526	73281819	0.947901	0.533	0.519
NM_011647	15	13	45	119	Tpm4	chr8	2082	74676017	0.947901	0.333	0.274
NM_026959	17	15	16	23	2510049I19Rik	chr8	1921	74746242	0.947901	0.451	0.41
NM_011430	20	16	32	49	9130011J15Rik	chr8	2842	75094787	0.744899	0.298	0.395
NM_027935	96	108	76	72	Large	chr8	3669	75738156	0.947901	0.558	0.514
NM_178703	20	18	22	21	AI931714	chr8	2781	80104599	0.395208	0.758	0.512
NM_030018	44	38	266	287	Lsm6	chr8	2107	81336918	0.947901	0.466	0.481
NM_016801	14	12	98	104	Abce1	chr8	3813	82211570	0.947901	0.517	0.485
NM_175101	24	23	22	20	Gab1	chr8	4877	83324196	0.947901	0.5	0.524
NM_001001491	13	26	187	232	Scoc	chr8	1931	85966470	0.947901	0.426	0.446
NM_008063	17	8	37	33	Gpsn2	chr8	1146	86118339	0.329905	0.373	0.529
NM_011585	32	25	11	111	Ddx39	chr8	1634	86244486	0.947901	0.094	0.09
NM_025435	48	39	129	160	D8Ertd738e	chr8	603	86770184	0.947901	0.474	0.446
NM_133352	24	21	10	27	Farsa	chr8	1846	87392913	0.947901	0.212	0.27
NM_011607	79	41	398	419	Prdx2	chr8	942	87497998	0.947901	0.478	0.487
NM_013835	17	14	27	22	Tnpo2	chr8	4865	87581303	0.947901	0.541	0.551
NM_013895	35	36	19	17	Dhps	chr8	1332	87597202	0.947901	0.489	0.528
NM_198214	16	15	43	24	Orc6l	chr8	1300	87831663	0.754784	0.536	0.642
NM_011590	82	94	19	22	Dnaja2	chr8	2839	88064009	0.947901	0.538	0.463

NM_011416	48	48	166	186	Neto2	chr8	5646	88193572	0.602323	0.551	0.472
NM_153059	27	14	42	30	Itfg1	chr8	2195	88360291	0.947901	0.571	0.583
NM_019442	32	8	32	55	Lonp2	chr8	2872	89189673	0.945732	0.293	0.368
NM_012058	26	21	34	12	Heatr3	chr8	3190	90695308	0.84104	0.816	0.739
NM_024225	23	19	37	50	Fto	chr8	3586	93925771	0.783653	0.563	0.425
NM_025335	28	32	247	175	Gnao1	chr8	2968	96490904	0.947901	0.591	0.585
NM_011522	15	11	35	34	Amfr	chr8	3694	96524295	0.947901	0.533	0.507
NM_025439	90	51	35	18	Mt1	chr8	402	96703158	0.947901	0.714	0.66
NM_175328	35	19	15	13	Arl2bp	chr8	1930	97197779	0.409809	0.75	0.536
NM_025804	71	62	47	28	Ciapin1	chr8	4327	97352265	0.947901	0.556	0.627
NM_007854	24	15	31	15	Gtl3	chr8	1217	97946048	0.26307	0.379	0.674
NM_013761	51	48	26	38	Got2	chr8	2345	98401634	0.844976	0.5	0.406
NM_011428	55	40	25	12	Tk2	chr8	2385	106755112	0.690932	0.481	0.676
NM_009215	52	34	11	15	Dync1li2	chr8	3145	106949372	0.883593	0.577	0.423
NM_019953	17	19	41	22	Hspc171	chr8	757	107852818	0.947901	0.586	0.651
NM_011990	14	30	10	18	Nip7	chr8	1350	109582693	0.42885	0.643	0.357
NM_023672	25	16	17	21	Nob1	chr8	1647	109948654	0.879992	0.561	0.447
NM_145390	33	28	58	41	Psmd7	chr8	1599	110104967	0.947901	0.561	0.586
NM_153176	16	24	89	45	2400003C14Rik	chr8	2304	112207643	0.805861	0.578	0.664
NM_177089	23	13	11	17	Calb2	chr8	1462	112680844	0.947901	0.308	0.393
NM_001005507	44	70	35	22	Cog4	chr8	2754	113405390	0.677707	0.504	0.614
NM_144825	52	35	63	57	Cfdp1	chr8	1215	114292454	0.947901	0.467	0.525
NM_009263	22	13	12	13	Kars	chr8	1953	114527284	0.80737	0.63	0.48
NM_020267	112	92	33	20	Terf2ip	chr8	3599	114543590	0.947901	0.675	0.623
NM_013873	15	18	118	110	AI427515	chr8	3655	116895704	0.947901	0.565	0.518
NM_025303	34	17	19	11	BC060631	chr8	4885	119483542	0.940846	0.517	0.633
NM_024272	49	32	18	9	Cdh13	chr8	2290	121491953	0.86501	0.531	0.667
NM_133854	11	20	118	83	Hsdl1	chr8	3140	122091813	0.710828	0.511	0.587
NM_026669	82	69	29	26	Cox4nb	chr8	4933	123180786	0.947901	0.609	0.527
NM_025382	36	38	46	37	Map1lc3b	chr8	1712	124121889	0.947901	0.5	0.554
NM_029701	45	48	10	26	Mvd	chr8	1767	124967275	0.805322	0.417	0.278
NM_172804	54	51	180	245	Aprt	chr8	866	125098539	0.947901	0.425	0.424
NM_026933	28	27	23	25	Spg7	chr8	2558	125621394	0.947901	0.4	0.479
NM_023055	11	15	32	8	Rpl13	chr8	710	125628985	0.465109	0.615	0.8
NM_145076	17	11	84	62	Tcf25	chr8	2695	125927527	0.947901	0.534	0.575
NM_130796	13	15	30	17	Def8	chr8	3756	125985376	0.689001	0.493	0.638
NM_021335	44	45	30	8	Dbndd1	chr8	1564	126033850	0.77027	0.667	0.789
NM_144867	120	10	33	27	Rhou	chr8	3367	126185003	0.520642	0.375	0.55
NM_001076554	106	97	15	24	Rab4a	chr8	1382	126358704	0.805861	0.516	0.385
NM_020618	38	15	35	37	Abcb10	chr8	4325	126491961	0.602769	0.679	0.486
NM_133668	56	45	19	30	Galnt2	chr8	4131	126868087	0.179836	0.66	0.388
NM_009261	31	24	16	27	2310022B05Rik	chr8	3326	127163444	0.947901	0.383	0.372
NM_009274	40	36	32	22	Tsnax	chr8	2391	127556696	0.844355	0.689	0.593
NM_013697	907	912	57	11	Birc2	chr9	3155	7834004	0.383093	0.633	0.838
NM_181266	17	8	49	46	Mtmr2	chr9	4935	13609824	0.947901	0.469	0.516
NM_134151	27	27	39	24	Endod1	chr9	4470	14158589	0.947901	0.634	0.619
NM_178648	31	39	73	47	0610040D20Rik	chr9	1152	14314955	0.805861	0.485	0.608
NM_025899	143	94	15	10	Mrpl4	chr9	1312	20811282	0.859423	0.719	0.6
NM_023045	21	10	27	17	Dpy19l1	chr9	4474	24245217	0.947901	0.533	0.614
NM_026031	15	17	100	107	Sep7	chr9	2398	25115934	0.86501	0.559	0.483
NM_025645	25	18	12	13	Acad8	chr9	2865	26785908	0.947901	0.37	0.48
NM_153194	15	10	21	18	Vps26b	chr9	3608	26837523	0.947901	0.5	0.538

NM_011981	38	42	139	16	Opcml	chr9	6002	28727877	0.947901	0.848	0.897
NM_024475	16	20	90	54	Aplp2	chr9	3490	30969362	0.728089	0.534	0.625
NM_175552	19	10	15	23	Kirrel3	chr9	3698	34842454	0.936394	0.5	0.395
NM_133756	51	18	18	29	Ddx25	chr9	1675	35354456	0.947901	0.423	0.383
NM_018871	137	97	20	17	Fez1	chr9	1289	36671093	0.947901	0.543	0.541
NM_028599	30	18	407	212	AW551984	chr9	4293	39407239	0.947901	0.665	0.658
NM_021324	12	13	110	90	Scn3b	chr9	4267	40098830	0.419101	0.423	0.55
NM_178811	14	14	20	11	Sorl1	chr9	6938	41830507	0.301202	0.364	0.645
NM_025970	29	26	13	13	Hmbs	chr9	1611	44152188	0.947901	0.5	0.5
NM_001081750	10	19	19	13	Slc37a4	chr9	2109	44206506	0.947901	0.68	0.594
NM_001081413	26	25	117	110	Trappc4	chr9	1196	44215247	0.853013	0.571	0.515
NM_178027	24	24	30	28	1500035H01Rik	chr9	3337	44600498	0.886203	0.591	0.517
NM_133349	149	149	23	23	Mll1	chr9	16439	44653576	0.947901	0.577	0.5
NM_028360	18	10	290	321	Fxyd6	chr9	1804	45203247	0.329905	0.552	0.475
NM_021499	53	56	81	39	Bace1	chr9	4099	45668385	0.947901	0.663	0.675
NM_023716	258	109	49	93	Rnf214	chr9	3782	45708049	0.477128	0.5	0.345
NM_009466	23	16	85	67	Pafah1b2	chr9	4241	45775842	0.835862	0.483	0.559
NM_173392	15	11	30	21	Zfp259	chr9	2984	46090473	0.947901	0.556	0.588
NM_001013745	33	11	254	645	Rplp1	chr9	468	61761110	0.947901	0.281	0.283
NM_027946	45	32	22	27	Anp32a	chr9	2105	62225136	0.947901	0.429	0.449
NM_021885	72	53	29	50	Ppib	chr9	979	65914391	0.468051	0.52	0.367
NM_009450	237	49	29	50	Snx22	chr9	2555	65914391	0.468051	0.52	0.367
NM_144839	49	25	58	29	Grinl1a	chr9	2172	71326465	0.665352	0.533	0.667
NM_011650	89	112	33	56	Arpp19	chr9	4032	74906008	0.210985	0.539	0.371
NM_011752	20	16	125	83	Myo5a	chr9	11684	75069246	0.947901	0.621	0.601
NM_033526	27	24	27	21	Leo1	chr9	2192	75314042	0.793113	0.7	0.563
NM_001081300	27	17	110	215	Scg3	chr9	2161	75491221	0.085256	0.537	0.338
NM_025418	48	35	39	47	Elovl5	chr9	2773	77830507	0.933287	0.529	0.453
NM_023249	30	28	45	35	Fbxo9	chr9	1858	77956845	0.947901	0.618	0.563
NM_023585	23	21	58	67	Tmem30a	chr9	3652	79617189	0.710828	0.621	0.464
NM_013699	31	46	22	37	Hmgn3	chr9	823	83040061	0.783653	0.514	0.373
NM_198617	20	20	93	121	Atp1b3	chr9	1833	96240772	0.247107	0.575	0.435
NM_023230	74	76	46	23	Clstn2	chr9	4494	97483046	0.693737	0.553	0.667
NM_178411	9	18	51	53	Rbp1	chr9	2628	98345026	0.947901	0.508	0.49
NM_145367	35	43	37	22	Copb2	chr9	3043	98480708	0.525209	0.407	0.627
NM_025388	67	54	42	9	Pccb	chr9	2291	100913217	0.80737	0.722	0.824
NM_177325	42	38	92	140	Anapc13	chr9	381	102536488	0.299056	0.595	0.397
NM_172054	24	32	12	21	Abhd14a	chr9	1258	106347918	0.641958	0.556	0.364
NM_012038	21	27	16	36	Gnai2	chr9	2177	107522390	0.910682	0.376	0.308
NM_133208	37	11	29	14	Rbm5	chr9	3085	107643156	0.947901	0.696	0.674
NM_172735	41	16	49	42	Ihpk1	chr9	4429	107948058	0.947901	0.483	0.538
NM_145967	17	16	30	29	Nicn1	chr9	2103	108198160	0.926181	0.586	0.508
NM_011673	15	19	135	78	Rhoa	chr9	2126	108239985	0.80737	0.517	0.634
NM_172745	60	56	45	27	Nme6	chr9	1025	109745090	0.498119	0.758	0.625
NM_011743	13	15	123	96	Mtap4	chr9	5681	109984838	0.947901	0.559	0.562
NM_026893	32	26	18	13	Cspg5	chr9	2144	110158649	0.945911	0.493	0.581
NM_207301	55	25	114	116	Clasp2	chr9	5627	113827795	0.947901	0.482	0.496
NM_026521	86	118	84	105	Ubp1	chr9	3749	113884724	0.947901	0.403	0.444
NM_021539	116	111	21	10	Cnot10	chr9	2853	114510483	0.947901	0.686	0.677
NM_025842	304	311	309	266	Rpl14	chr9	935	120483427	0.892484	0.568	0.537
NM_028209	18	8	116	49	Exosc7	chr9	1037	123045047	0.365698	0.576	0.703

Table S1.11. Known imprinted genes reported to be imprinted in placenta.

mouse Chr band	type	RefSeq ID / GenBank ID	Entrez gene ID	MGIsymbol	expressed _allele	References
2 E1-H3	coding-gene	AF175305	-	Nesp	M	1
2 E5	coding-gene	NM_025961.5	67092	Gatm	M	2
6 A1	coding-gene	NM_011360.2	20392	Sgce	P	3
6 A1	coding-gene	NM_001040611.1	170676	Peg10	P	4
6 A1	coding-gene	NM_023048.5	65255	Asb4	M	5
6 A1	coding-gene	NM_181595.3	243725	Ppp1r9a	M	4
7 F5	coding-gene	NM_010514.2	16002	Igf2	P	6,7
7 F5	antisense	NR_002855	-	Igf2as	P	8
7 F5	coding-gene	NM_008554.2	17173	Ascl2 (Mash2)	M	9
7 F5	coding-gene	AF175771	-	Phemx (Tssc6)	M	10
7 F5	coding-gene	NM_020285.1	56844	Tssc4	M	11
7 F5	coding-gene	NM_009434	22113	Phlda2 (Tdag51)	M	12
7 F5	coding-gene	NM_008672.2	17955	Nap1l4	M	13
7 F5	coding-gene	NM_024290.3	79201	Tnfrsf23	M	14
7 F5	coding-gene	NM_024289.2	79196	Osbpl5	M	13
10 A1	coding-gene	NM_009538.1	22634	Plagl1	P	15
10 C3	coding-gene	NM_007833.4	13179	Dcn	M	5
11 A1	coding-gene	NM_010345.3	14783	Grb10 (Meg1)	M	16,17
12 F1	coding-gene	NM_010052.4	13386	Dlk1 (Peg9)	P	18
12 F1	Non-coding RNA	XR_035480.1	17263	Gtl2 (Meg3)	M	19
12 F1	coding-gene	NM_184109.1	353326	Rtl1(Peg11)	P	20
12 F1	antisense	-	-	anti-Rtl1 (antiPeg11)	M	20
12 F1	miRNA	-	-	miR-127 (antiPeg11)	M	20
12 F1	miRNA	-	-	miR-136 (antiPeg11)	M	20
12 F1	miRNA	-	-	miR-370	M	21
12 F1	Non-coding RNA	AJ517767	-	Mirg	M	21
15 F1	coding-gene	NM_027052.3	69354	Slc38a4	P	5
17 A1	coding-gene	NM_013667.2	20518	Slc22a2	M	22
17 A1	coding-gene	NM_011395.2	20519	Slc22a3	M	22

References:

1. J. Peters, S. F. Wroe, C. A. Wells et al., *Proceedings of the National Academy of Sciences of the United States of America* **96** (7), 3830 (1999).
2. L. L. Sandell, X. J. Guan, R. Ingram et al., *Proceedings of the National Academy of Sciences of the United States of America* **100** (8), 4622 (2003).
3. G. Piras, A. El Kharroubi, S. Kozlov et al., *Molecular and cellular biology* **20** (9), 3308 (2000).
4. R. Ono, H. Shiura, H. Aburatani et al., *Genome Res* **13** (7), 1696 (2003).
5. Y. Mizuno, Y. Sotomaru, Y. Katsuzawa et al., *Biochemical and biophysical research communications* **290** (5), 1499 (2002).
6. M. Hemberger, C. Redies, R. Krause et al., *Development genes and evolution* **208** (7), 393 (1998).
7. T. M. DeChiara, E. J. Robertson, and A. Efstratiadis, *Cell* **64** (4), 849 (1991).
8. T. Moore, M. Constancia, M. Zubair et al., *Proceedings of the National Academy of Sciences of the United States of America* **94** (23), 12509 (1997).

[9] F. Guillemot, T. Caspary, S. M. Tilghman et al., *Nature genetics* **9** (3), 235 (1995).
[10] D. Umlauf, Y. Goto, R. Cao et al., *Nature genetics* **36** (12), 1296 (2004).
[11] M. Paulsen, O. El-Maarri, S. Engemann et al., *Human molecular genetics* **9** (12), 1829 (2000).
[12] N. Qian, D. Frank, D. O'Keefe et al., *Human molecular genetics* **6** (12), 2021 (1997).
[13] S. Engemann, M. Strodicke, M. Paulsen et al., *Human molecular genetics* **9** (18), 2691 (2000).
[14] L. Clark, M. Wei, G. Cattoretti et al., *BMC genetics* **3**, 11 (2002).
[15] M. Kamiya, H. Judson, Y. Okazaki et al., *Human molecular genetics* **9** (3), 453 (2000).
[16] T. Hikichi, T. Kohda, T. Kaneko-Ishino et al., *Nucleic acids research* **31** (5), 1398 (2003).
[17] N. Miyoshi, Y. Kuroiwa, T. Kohda et al., *Proceedings of the National Academy of Sciences of the United States of America* **95** (3), 1102 (1998).
[18] S. Takada, M. Tevendale, J. Baker et al., *Curr Biol* **10** (18), 1135 (2000).
[19] J. V. Schmidt, P. G. Matteson, B. K. Jones et al., *Genes & development* **14** (16), 1997 (2000).
[20] H. Seitz, N. Youngson, S. P. Lin et al., *Nature genetics* **34** (3), 261 (2003).
[21] H. Seitz, H. Royo, M. L. Bortolin et al., *Genome Res* **14** (9), 1741 (2004).
[22] R. Zwart, F. Sleutels, A. Wutz et al., *Genes & development* **15** (18), 2361 (2001).

Table S1.12. Known imprinted genes covered by assembly of Solexa reads.

RefSeq_ID	PWD x AKR		AKR x PWD		Gene_name	Chr	RefSeq_len
	AKR_counts	PWD_counts	AKR_counts	PWD_counts			
NM_011245	16	0	0	20	Rasgrf1	chr9	4243
NR_002864	168	0	6	74	Peg13	chr15	4745
XR_035484	1	339	193	1	Gtl2	chr12	1890
NM_001077507	181	214	101	96	Gnas	chr2	3733
NM_001033962	3	10	4	2	Ube3a	chr7	4910
NM_010514	52	43	20	27	Igf2	chr7	4038
NM_008672	9	12	61	76	Nap1l4	chr7	2283
NM_009876	0	8	13	0	Cdkn1c	chr7	1849
NM_010345	2	3	6	10	Grb10	chr11	5015
NM_021432	22	0	0	67	Nap1l5	chr6	1909
NM_181595	36	54	67	26	Ppp1r9a	chr6	9547
NR_001592	2	14	61	1	H19	chr7	2615

Table S1.13. Novel SNPs called in known imprinted genes from assembly of the Solexa reads.

Gene	SNP	RefSeq pos	RefSeq length	RafSeq Allele	AKR allele	PWD allele	PWD x AKR		AKR x PWD		Pyro verification
							AKR_counts	PWD_counts	AKR_counts	PWD_counts	
Rasgrf1	NES14232081	3736	4243	T	T	C	1	0	0	8	
Rasgrf1	newSNP1	4176	4243	A	A	G	12	0	0	6	YES
Rasgrf1	newSNP2	4202	4243	C	C	T	3	0	0	6	
Peg13	newSNP1	4333	4745	C	C	T	42	0	4	31	
Peg13	newSNP2	4347	4745	C	C	T	38	0	2	21	
Peg13	newSNP3	4426	4745	G	G	A	88	0	0	22	YES
Gtl2	newSNP1	1557	1890	T	T	C	1	172	19	1	YES
Gtl2	newSNP2	1820	1890	G	G	A	0	48	67	0	
Gtl2	newSNP3	1822	1890	A	A	G	0	53	61	0	
Gtl2	newSNP4	1846	1890	A	A	G	0	66	46	0	
Gnas	newSNP1	3162	3733	G	G	C	181	214	101	96	YES
Ube3a	newSNP1	2363	4910	T	T	C	3	10	4	2	YES
Igf2	newSNP1	3831	4308	G	G	A	52	43	20	27	YES
Nap1l4	newSNP1	2221	2283	C	C	T	9	12	61	76	too close to 3'-end
Cdkn1c	newSNP1	1727	1849	C	C	T	0	8	13	0	YES
Grb10	newSNP1	4514	5015	G	G	A	2	3	6	10	YES
Nap1l5	newSNP1	1224	1909	C	C	T	22	0	0	67	YES
Ppp1r9a	NES10359547	9050	9547	T	T	G	9	16	14	13	
Ppp1r9a	newSNP1	9202	9547	A	A	G	12	20	28	7	
Ppp1r9a	NES10359548	9211	9547	C	C	G	15	18	25	6	
H19	newSNP1	2237	2615	G	G	A	1	5	30	0	
H19	newSNP2	2401	2615	A	A	G	1	5	8	0	
H19	newSNP3	2437	2615	G	G	A	0	4	23	1	YES

Table S1.14. Categories of Perlegen SNP in the RefSeq sequences.

Categories	Coordinates filter	Text match	Status	Number of RefSeq sequences	% Total
Conisistent	YES	YES	Consistent	172,104	83.31%
Corrected by text match *	YES	YES	Inconsistent, corrected by text match	15,679	7.59%
Coordinates information only **	YES	NO	Coordinates only	18,806	9.10%
Total				206,589	

*: There are discrepency between the RefSeq and genomic sequence alignment. The gaps in the alignment could be corrected by the text match.

**: Not all coordinate filtered SNPs have text match results, because of the exon-intron boundary problem.

Table S1.15. Consistency of Perlegen SNP alleles and the RefSeq alleles.

Categories	Example			Counts	% of Total
	RefSeq	P_reference	P_alternative		
RefSeq allele is conisistent with Perlegen reference allele	A	A	C	202,718	98.13%
RefSeq allele is conisistent with Perlegen alternative allele *	A	C	A	3,829	1.85%
RefSeq allele is NOT conisistent with Perlegen alleles **	A	G	T	42	0.02%
Total				206,589	

*: The Perlegen reference allele is from the reference genome sequence, which is C57BL/6. Not all the RefSeq sequences come from the C57BL/6 strain whose genome has been sequenced, so we saw 1.85% of the total SNPs matching the Perlegen alternative allele.

**: The inconsistent SNPs could be due to Perlegen genotyping error, RefSeq sequencing error or RNA editing.

Table S1.16. Solexa SNP categories.

Num	Desc	RefSeq	P1	P2	AKR	PWD	Solexa
0	inconsistencies						
1	complete consistent (SNPs)	A	A	C	A	C	A
2	complete consistent (not a SNP)	A	A	C	C	C	C
3	consistent with one N (not matching N)	A	A	C	N	C	C
4	consistent with one N (matching N)	A	A	C	N	C	A
5	consistent with two Ns	A	A	C	N	N	C

RefSeq: the SNP allele in the RefSeq database.

P1: the Perlgene reference SNP allele (the C57BL/6 strain).

P2: the Perlgene alternative SNP allele.

AKR: the AKR allele from Perlegen database (A, T, C, G or N, N stards for missing data or not tested).

PWD: PWD allele from Perlegen database (A, T, C, G or N, N stards for missing data or not tested).

Solexa: SNP allele extracted from matched Solexa reads.

Table S1.17. Class 1 SNPs excluded from the analysis.

SNP_ID	SNP_ID	SNP_ID	SNP_ID
NES14876696	NES10420240	NES13745918	NES12249028
NES09738047	NES10420226	NES11943616	NES15568316
NES08906420	NES10420223	NES09032590	NES17087050
NES09734134	NES15049856	NES08986232	NES17087048
NES08906420	NES14979137	NES08986229	NES17087049
NES09621998	NES14351208	NES14949585	NES17087047
NES11209005	NES11238747	NES16255894	NES11827956
NES15138749	NES17483578	NES17479911	NES09237212
NES08431278	NES17483577	NES16859105	NES09237207
NES15875741	NES17463891	NES09012401	NES09237201
NES15647702	NES11670031	NES17235026	NES09237213
NES11971118	NES16150391	NES12718299	NES09237210
NES17027650	NES09025562	NES12266934	NES09237205
NES08459947	NES16206267	NES08788167	NES09237202
NES08459950	NES08572902	NES08788166	NES16593135
NES15266287	NES14261667	NES12691145	NES08484090
NES14293779	NES12712485	NES12691167	NES14546805
NES14293775	NES17750869	NES11550691	NES14546803
NES14293777	NES17750860	NES11550693	NES14546811
NES14293778	NES15156758	NES12399504	NES14546813
NES11209751	NES15695629	NES15930027	NES14546807
NES13703477	NES11369015	NES08378706	NES16561739
NES10739665	NES10714421	NES08378705	NES14293775
NES10660704	NES10714429	NES08547137	NES14293777
NES16285283	NES15073396	NES15189427	NES14293778
NES16285283	NES09233319	NES08868052	NES14293779
NES10873154	NES09233320	NES12433551	NES16563387
NES15266522	NES15285346	NES17079130	NES16781606
NES15266522	NES09279050	NES08544194	NES15422496
NES15266522	NES14776963	NES15552234	NES09167411
NES15259264	NES14876696	NES14104611	NES11326902
NES10312342	NES11238747	NES15678869	NES15421000
NES14871332	NES17010767	NES12919589	NES13029528
NES08458564	NES09446275	NES16623938	NES09693916
NES14225365	NES08483436	NES08906420	NES08644572
NES10319035	NES08483415	NES09026926	NES13748736
NES16130718	NES08483435	NES13973227	NES16127294
NES16130720	NES16702750	NES17024295	NES11608738
NES16358702	NES17409492	NES17024369	NES11608740
NES08666099	NES15643653	NES08405830	NES10311402
NES13522888	NES17358191	NES14982676	NES16285283
NES10420230	NES17043132	NES15266522	NES16739532
NES10420224	NES09155409	NES17291091	NES13649859

SNP_ID	SNP_ID	SNP_ID	SNP_ID
NES17291097	NES09171102	NES17612490	NES10699965
NES17291098	NES11190333	NES10873154	NES13919030
NES14029298	NES14557213	NES14052359	NES14733595
NES10448144	NES14822643	NES08660120	NES14438492
NES12846890	NES13822060	NES14659667	NES10273169
NES16711194	NES09782280	NES11943616	NES15074478
NES17234293	NES10390975	NES15693632	NES09342784
NES13826540	NES10963117	NES16578142	NES11596049
NES08427844	NES14871332	NES13826540	NES14639695
NES13494138	NES15679880	NES16188935	NES10463847
NES15990474	NES16980640	NES09721304	NES15461042
NES13837750	NES16980639	NES13651334	NES17507704
NES09097215	NES10255687	NES09166157	NES11610293
NES10281979	NES15068410	NES09166158	NES14897932
NES10463847	NES16151877	NES11941242	NES11611860
NES10374048	NES16239673	NES11943616	NES08512189
NES16734327	NES15259264	NES11943616	NES15989288
NES17335704	NES15917484	NES11943616	NES15989279
NES14641192	NES15917483	NES11943616	NES15989289
NES10387308	NES15917573	NES11943616	NES16280702
NES12729871	NES08589567	NES11943616	NES13440047
NES09451085	NES11605743	NES11943616	NES09662684
NES15815096	NES11274422	NES14765284	
NES16541439	NES17539058	NES13626579	
NES16541431	NES16301862	NES15719085	
NES16541234	NES16590360	NES17122816	
NES08533698	NES10319035	NES15072297	
NES08533495	NES16696227	NES13664543	
NES16084189	NES13506336	NES11088397	
NES14293779	NES11254770	NES11088396	
NES14293775	NES15219205	NES15284784	
NES14293777	NES17346264	NES13782115	
NES14293778	NES17346231	NES16151953	
NES14173600	NES14438492	NES09201112	
NES13653669	NES08481849	NES09201111	
NES17613413	NES16234958	NES09201113	
NES17613433	NES15890171	NES09201066	
NES17612622	NES08732510	NES16918136	
NES14770826	NES08766821	NES11984138	
NES14770886	NES16824504	NES11984136	
NES10744906	NES14364801	NES11984133	
NES13655958	NES14364643	NES15329638	
NES11277835	NES11391135	NES12216222	
NES10384314	NES11391139	NES08975230	

Table S1.18. Summary of the Perlegen-Solexa consistency.

		Before Manual correction			After Manual correction		
Category	Desc.	Counts	Percentage	% of Informative	Counts	Percentage	% of Informative
1	Uninformative	175572	84.65%	-	175687	84.71%	-
2	Multiallele	2757	1.33%	8.66%	318	0.15%	1.05%
3	Inconsistent	149	0.07%	0.47%	161	0.08%	0.53%
4	Confirmed	23133	11.15%	72.67%	25289	12.19%	83.21%
5	Called	4055	1.96%	12.74%	4127	1.99%	13.58%
6	DoubleN_consistent	461	0.22%	1.45%	496	0.24%	1.63%
7	SingleN_uninformative	1280	0.62%	-	1329	0.64%	-
Total		207407	100.00%		207407	100.00%	

Table S1.19. Differential methylation status at the DMR of known imprinted genes.

#	mouse Chr band	type	RefSeq ID / GenBank ID	MGIsymbol	Expressed allele	Type	DMR	Methylated allele	Mouse_Tissue	Refer
1	2 E1-H3	coding-gene	AF175305	Nesp	M	No_RefSeq	CpG island is methylated at the paternal allele	P	Embryos	4
2	2 E1-H3	coding-gene	AJ245739	Gnasxl	P	No_RefSeq	CpG island is methylated at the maternal allele	M	Pg embryos	3, 4
3	2 E1-H3	Non-coding RNA	-	Exon1A	P	No_RefSeq	CpG island is methylated at the maternal allele	M	Pg embryos	3
4	2 E1-H3	coding-gene	NM_001077507.1; NM_001077510.1; NM_010309.3; NM_019690.2; NM_022000.2; NM_201616.1; NM_201617.1; NM_201618.1; NR_003258.1	Gnas	M	Covered	Nesp DMR is paternally methylated; Gnasxl and Exon1A DMRs are maternally methylated	M	Pg embryos	3,5
5	2 E1-H3	antisense*	NR_002846	Nespas	P	No_SNP	-	-	-	-
6	2 E5	coding-gene	NM_025961.5	Gatm	M	Covered	The CpG island near the genes is not methylated in both alleles	-	Placenta and embryonic liver	6
7	2 H1	coding-gene	NM_010923.2; NM_180960.2	Nnat (peg5)	P	Covered	5 differentially methylated CpG sites within the gene reigon	M	Embryos/Pg embryos	2, 3
8	5 E1	pseudogene	NG_005709	Mkrn1-ps1	P	No_RefSeq	Fully methylated from both alleles; Not transcribed and not an imprinted gene	P+M	Not mentioned in the paper	11
9	6 A1	coding-gene	NM_001042725.1; NM_007588.2	Calcr	M	Covered	Not essayed	-	-	7
10	6 A1	coding-gene	NM_011360.2	Sgce	P	Covered	Promoter region CpG island is differential methylated	M	Pg embryos/E10 embryos and placenta	3, 9
11	6 A1	coding-gene	NM_001040611.1; NM_130877.2	Peg10	P	No_counts	Promoter region CpG island is differential methylated	M	E10 embryos and placenta	9
12	6 A1	coding-gene	NM_181595.3	Ppp1r9a	M	Covered	the CpG island is not differentially methylated	-	normal, mUPD7, and pUPD7 lymphoblastoid cells	8
13	6 A1	coding-gene	NM_023048.5	Asb4	M	Covered	Promoter region CpG island is NOT differential methylated	-	E10 embryos and placenta	9
14	6 A3	coding-gene	NM_008590.1	Mest (Peg1)	P	No_SNP	CpG island is methylated at the maternal allele	M	Pg embryos	3
15	6 A3	antisense	NR_002845	Copg2as2	M	No_chr6_hits	-	-	-	-
16	6 A3	coding-gene	NM_017478.2	Copg2	P	Covered	Higher paternal methylation at the promoter-associated CpG island	P	Brain	10

#	mouse Chr band	type	RefSeq ID / GenBank ID	MGIsymbol	Expressed allele	Type	DMR	Methylated allele	Mouse_Tissue	Refer
17	6 C1	coding-gene	NM_021432.2	Nap1l5	P	Covered	CpG island is methylated at the maternal allele	M	Pg embryos	3
18	7 A2-B1	coding-gene	NM_008817.2	Peg3	P	Covered	CpG island is methylated at the maternal allele	M	Pg embryos	3
19	7 A2-B1	coding-gene	NM_011769.3	Zim1	M	Covered	DMR not studied; close to Peg3	-	-	-
20	7 A2-B1	coding-gene	XM_922461, XM_489216	Zim2	M**	No_SNP	DMR not studied; close to Peg3	-	-	-
21	7 A2-B1	coding-gene	NM_021323.2	Usp29	P	No_counts	DMR not studied; close to Peg3	-	-	-
22	7 A2-B1	antisense	AF365932	Zim3	M	No_RefSeq	-	-	-	-
23	7 A2-B1	Non-coding RNA	AF365933	Zfp264	P	No_RefSeq	DMR not studied; close to Peg3			
24	7 B5	coding-gene	NM_013788.1	Peg12 (Frat3)	P	No_SNP	CpG island is methylation from the maternal allele	M	brain of wild-type,Tg-PWS(del) and Tg-AS(del) mice	12
25	7 B5	coding-gene	NM_011746.2	Mkrn3 (Zfp127)	P	No_counts	Preferentially maternal methylation	M	adult brain	13
26	7 B5	antisense	-	Zfp127as	P	No_RefSeq	-	-	-	-
27	7 B5	coding-gene	XM_622091.3	Magel2	P	Covered	DMR not studied; close to Peg12	-	-	-
28	7 B5	coding-gene	NM_010882.3	Ndn	P	No_counts	Partially methylated at both paternal and maternal alleles	-	adult brain	14
29	7 B5	miRNA	-	Nccr	P	No_RefSeq	DMR not studied; close to Peg12	-	-	-
30	7 B5	coding-gene	NM_033174, NM_001082962, NM_005678, NM_022804	Snurf	P	Covered	CpG island is methylated at the maternal allele	M	Pg embryos	3
31	7 B5	coding-gene	NM_001082961.1; NM_001082962.1; NM_013670.3	Snrpn	P	Covered	CpG island is methylated at the maternal allele	M	Pg embryos	3
32	7 B5	snoRNA	-	HBII-13	P	No_RefSeq	-	-	-	-
33	7 B5	snoRNA	AF241256	Pwcr1	P	No_RefSeq	-	-	-	-
34	7 B5	snoRNA	-	HBII-52	P	No_RefSeq	-	-	-	-
35	7 B5	antisense	AK049008	UBE3A-AS	P	No_RefSeq	-	-	-	-
36	7 B5	coding-gene	NM_001033962.1; NM_011668.2; NM_173010.3	Ube3a	M	Covered	DMR not studied; close to Snrpn	-	-	-
37	7 F3	coding-gene	DQ648020	Inpp5f_v2	P	Covered	CpG1 is not methylated; CpG2 is DMR and it is only methylated in the paternal allele.	P	B6xCast.(8 weeks brain and kidney)	1
38	7 F5	Non-coding RNA	NR_001592	H19	M	Covered	upstream DMR is methylated only from the paternal allele	P	Not mentioned in the paper	15
39	7 F5	coding-gene	NM_010514.2	Igf2	P	Covered	DMR is methylated only from the paternal allele	P	Not mentioned in the paper	15

#	mouse Chr band	type	RefSeq ID / GenBank ID	MGIsymbol	Expressed allele	Type	DMR	Methylated allele	Mouse_Tissue	Refer
40	7 F5	antisense	NR_002855	Igf2as	P	No_SNP	-	-	-	-
41	7 F5	coding-gene	NM_008387.3	Ins2	P	No_SNP	DMR not studied; close to Igf2	-	-	-
42	7 F5	coding-gene	NM_008554.2	Ascl2 (Mash2)	M	No_counts	No differential methylation was detected	-	Embryos, placenta, V endoderm, YS mesoderm, adult liver	16
43	7 F5	coding-gene	AF175771	Phemx (Tssc6)	M	No_RefSeq	No differential methylation was detected	-	Embryos, placenta, V endoderm, YS mesoderm, adult liver	16
44	7 F5	coding-gene	NM_133655.1	Cd81 (Impt1)	M	No_SNP	No differential methylation was detected	-	Embryos, placenta, V endoderm, YS mesoderm, adult liver	16
45	7 F5	coding-gene	NM_020285.1; NM_138631.1	Tssc4	M	No_SNP	Slightly methylated on the paternal allele in the trophoblast and yolk sac endoderm	P	Embryos, placenta, V endoderm, YS mesoderm, adult liver	16
46	7 F5	coding-gene	NM_008434.2	Kcnq1	M	No_counts	No differential methylation was detected	-	Embryos, placenta, V endoderm, YS mesoderm, adult liver	16
47	7 F5	antisense	NR_001461	Kcnq1ot1	P	No_SNP	Maternal methylation at the KvDMR1 (IC2) CpG island	M	Embryos, placenta, V endoderm, YS mesoderm, adult liver	16
48	7 F5	coding-gene	NM_009876.3	Cdkn1c (p57KIP2)	M	Covered	Paternal methylation at the two Cdkn1c CpG islands	P	Embryos, placenta, V endoderm, YS mesoderm, adult liver	16
49	7 F5	Non-coding RNA	AF313042	Msuit1	M	No_RefSeq	No differential methylation was detected	-	Embryos, placenta, V endoderm, YS mesoderm, adult liver	16
50	7 F5	coding-gene	NM_001042760.1; NM_008767.2	Slc22a18	M	No_counts	No differential methylation was detected	-	Embryos, placenta, V endoderm, YS mesoderm, adult liver	16
51	7 F5	coding-gene	NM_009434	Phlda2 (Tdag51)	M	No_SNP	No differential methylation was detected	-	Embryos, placenta, V endoderm, YS mesoderm, adult liver	16
52	7 F5	coding-gene	NM_008672.2	Nap1l4	M	Covered	No differential methylation was detected	-	Embryos, placenta, V endoderm, YS mesoderm, adult liver	16
53	7 F5	coding-gene	NM_024290.3	Tnfrsf23	M	Covered	No differential methylation was detected	-	Embryos, placenta, V endoderm, YS mesoderm, adult liver	16
54	7 F5	coding-gene	NM_024289.2	Osbpl5	M	Covered	No differential methylation was detected	-	Embryos, placenta, V endoderm, YS mesoderm, adult liver	16
55	9 E3	Non-coding RNA	AK015891	4930524O08Rik,A19	P	No_RefSeq	-	-	-	-
56	9 E3	coding-gene	NM_001039655.1; NM_011245.2	Rasgrf1	P	Covered	The DMR is methylated at the paternal allele	P	Tail	17

#	mouse Chr band	type	RefSeq ID / GenBank ID	MGIsymbol	Expressed allele	Type	DMR	Methylated allele	Mouse_Tissue	Refer
57	10 A1	coding-gene	NM_009538.1	Plagl1	P	No_counts	CpG island is methylated at the maternal allele	M	Pg embryos	3
58	10 C3	coding-gene	NM_007833.4	Dcn	M	Covered	DMR not studied	-	-	-
59	11 A1	coding-gene	NM_010345.3	Grb10 (Meg1)	M(major type)	Covered	3 CpG islands. CpG1 is not differentially methylated. CpG2 is methylated only at the maternall allele.CpG3 is a tissue-specific DMR	M	Brain, UPD cell lines	18
60	11 A3	coding-gene	NM_144514.2	Commd1	M	Covered	CpG island is methylated at the maternal allele	M	Kidney	19
61	11 A3	coding-gene	NM_011663.2	Zrsr1 (U2af1-rs1)	P	Covered	CpG island is methylated at the maternal allele	M	Pg embryos	3
62	12 F1	coding-gene	NM_010052.4	Dlk1 (Peg9)	P	No_counts	The promoter region is not differentially methylated	-		20
63	12 F1	Non-coding RNA	-	Dlk1downstreamtranscripts	P	No_RefSeq	-	-	-	-
64	12 F1	Non-coding RNA	NR_003633.1; XR_035480.1; XR_035483.1; XR_035484.1	Gtl2 (Meg3)	M	Covered	Promoter CpG island is methylated at the paternal allele	P	mUPD12 and pUPD12 embryos	20
65	12 F1	miRNA	-	miR-337	M	No_RefSeq	-	-	-	-
66	12 F1	coding-gene	NM_184109.1	Rtl1 (Peg11)	P	Covered	Close to Gtl2 DMR	-	-	-
67	12 F1	antisense	-	anti-Rtl1 (antiPeg11)	M	No_RefSeq	-	-	-	-
68	12 F1	miRNA	-	miR-127 (antiPeg11)	M	No_RefSeq	-	-	-	-
69	12 F1	miRNA	-	miR-136 (antiPeg11)	M	No_RefSeq	-	-	-	-
70	12 F1	snoRNA	XM_901568	Rian (Meg8)	M	No_SNP	Close to Gtl2 DMR	-	-	21
71	12 F1	miRNA	-	miR-370	M	No_RefSeq	-	-	-	-
72	12 F1	snoRNA	-	MBII-78	M	No_RefSeq	-	-	-	-
73	12 F1	snoRNA	-	MBII-19	M	No_RefSeq	-	-	-	-
74	12 F1	snoRNA	-	14q(0)	M	No_RefSeq	-	-	-	-
75	12 F1	snoRNA	-	MBII-48	M	No_RefSeq	-	-	-	-
76	12 F1	snoRNA	-	MBII-49	M	No_RefSeq	-	-	-	-
77	12 F1	snoRNA	-	MBII-426	M	No_RefSeq	-	-	-	-
78	12 F1	snoRNA	-	MBII-343	M	No_RefSeq	-	-	-	-
79	12 F1	Non-coding RNA	AJ517767	Mirg	M	No_RefSeq	Close to Gtl2 DMR	-	-	21
80	12 F1	miRNA	-	miR-411	M	No_RefSeq	-	-	-	-
81	12 F1	miRNA	-	miR-380	M	No_RefSeq	-	-	-	-
82	12 F1	miRNA	-	miR-376b	M	No_RefSeq	-	-	-	-
83	12 F1	miRNA	-	miR-376	M	No_RefSeq	-	-	-	-
84	12 F1	miRNA	-	miR-134	M	No_RefSeq	-	-	-	-
85	12 F1	miRNA	-	miR-154	M	No_RefSeq	-	-	-	-
86	12 F1	miRNA	-	miR-410	M	No_RefSeq	-	-	-	-

#	mouse Chr band	type	RefSeq ID / GenBank ID	MGIsymbol	Expressed allele	Type	DMR	Methylated allele	Mouse_Tissue	Refer
87	12 F1	coding-gene	NM_172119.2	Dio3	P	No_SNP	Close to Gtl2 DMR	-	-	-
88	14 D2	coding-gene	NM_172812.2	Htr2a	M	No_counts	DMR not studied	-	-	-
89	15 D3	Non-coding RNA	NR_002864	Peg13	P	Covered	CpG island is methylated at the maternal allele	M	Pg embryos	3
90	15 D3	coding-gene	NM_001033876	Kcnk9	M	No_SNP	CpG island is methylated at the maternal allele	M	brain	22
91	15 F1	coding-gene	NM_027052.3	Slc38a4	P	Covered	CpG island is methylated at the maternal allele	M	Pg embryos	3
92	17 A1	coding-gene	NM_010515.2	Igf2r	M	No_counts	-	-	-	-
93	17 A1	antisense	NR_002853	Air	P	No_SNP	CpG island is methylated at the maternal allele	M	brain	23
94	17 A1	coding-gene	NM_013667.2	Slc22a2	M	Covered	Regulated by Igf2r-Air DMR	-	-	24
95	17 A1	coding-gene	NM_011395.2	Slc22a3	M	No_counts	Regulated by Igf2r-Air DMR	-	-	24
96	18 A2-B2	coding-gene	NM_008378.2	Impact	P	Covered	CpG island is methylated at the maternal allele	M	F1 hybrids of B6 and JF, tails	25
97	X D	Non-coding RNA	NR_001463.2; NR_001570.1	Xist	P	No_SNP	CpG island is methylated in sperm, but not oocyte	P	-	26
98	X D	antisense	NR_002844.1	Tsix	M	No_SNP	-	-	-	-

*: The DMR of the antisense transcript is usually the same as the sense one, since they are closely linked.

References:

1 J. D. Choi, L. A. Underkoffler, A. J. Wood et al., *Molecular and cellular biology* **25** (13), 5514 (2005).

2 N. Kikyo, C. M. Williamson, R. M. John et al., *Developmental biology* **190** (1), 66 (1997).

3 R. J. Smith, W. Dean, G. Konfortova et al., *Genome research* **13** (4), 558 (2003).

4 J. Peters, S. F. Wroe, C. A. Wells et al., *Proceedings of the National Academy of Sciences of the United States of America* **96** (7), 3830 (1999).

5 J. Liu, S. Yu, D. Litman et al., *Molecular and cellular biology* **20** (16), 5808 (2000).

6 L. L. Sandell, X. J. Guan, R. Ingram et al., *Proceedings of the National Academy of Sciences of the United States of America* **100** (8), 4622 (2003).

7 H. Hoshiya, M. Meguro, A. Kashiwagi et al., *Journal of human genetics* **48** (4), 208 (2003).

8 K. Nakabayashi, S. Makino, S. Minagawa et al., *Journal of medical genetics* **41** (8), 601 (2004).

9 R. Ono, H. Shiura, H. Aburatani et al., *Genome research* **13** (7), 1696 (2003).

10 J. Yun, C. W. Park, Y. J. Lee et al., *Mamm Genome* **14** (6), 376 (2003).

11 T. A. Gray, A. Wilson, P. J. Fortin et al., *Proceedings of the National Academy of Sciences of the United States of America* **103** (32), 12039 (2006).

12 J. H. Chai, D. P. Locke, T. Ohta et al., *Mamm Genome* **12** (11), 813 (2001).

13 A. Hershko, A. Razin, and R. Shemer, *Gene* **234** (2), 323 (1999).

14 M. L. Hanel and R. Wevrick, *Molecular and cellular biology* **21** (7), 2384 (2001).

15 A. C. Bell and G. Felsenfeld, *Nature* **405** (6785), 482 (2000).

16 A. Lewis, K. Mitsuya, D. Umlauf et al., *Nature genetics* **36** (12), 1291 (2004).

17 B. J. Yoon, H. Herman, A. Sikora et al., *Nature genetics* **30** (1), 92 (2002).

[18] P. Arnaud, D. Monk, M. Hitchins et al., *Human molecular genetics* **12** (9), 1005 (2003).
[19] Z. Zhang, K. Joh, H. Yatsuki et al., *Gene* **366** (1), 77 (2006).
[20] S. Takada, M. Tevendale, J. Baker et al., *Curr Biol* **10** (18), 1135 (2000).
[21] J. Cavaille, H. Seitz, M. Paulsen et al., *Human molecular genetics* **11** (13), 1527 (2002).
[22] N. Ruf, S. Bahring, D. Galetzka et al., *Human molecular genetics* **16** (21), 2591 (2007).
[23] J. F. Hu, K. A. Balaguru, R. D. Ivaturi et al., *Biochemical and biophysical research communications* **257** (2), 604 (1999).
[24] R. Zwart, F. Sleutels, A. Wutz et al., *Genes & development* **15** (18), 2361 (2001).
[25] K. Okamura, Y. Hagiwara-Takeuchi, T. Li et al., *Genome research* **10** (12), 1878 (2000).
[26] L. E. McDonald, C. A. Paterson, and G. F. Kay, *Genomics* **54** (3), 379 (1998).
