# Supplementary material for: Transcriptome-Wide Identification of Novel Imprinted Genes in Neonatal Mouse Brain
Source: PLoS One. 2008 Dec 4;3(12):e3839. doi: 10.1371/journal.pone.0003839 (PMC2585789; doi:10.1371/journal.pone.0003839)
Supplement: References S1 — Supporting references for Table 1. (0.03 MB DOC) [file pone.0003839.s003.doc]

**References S1. Supporting References for Table 1**

1. Kagitani F, Kuroiwa Y, Wakana S, Shiroishi T, Miyoshi N, et al. (1997) Peg5/Neuronatin is an imprinted gene located on sub-distal chromosome 2 in the mouse. Nucleic Acids Res 25: 3428-3432.

2. Leff SE, Brannan CI, Reed ML, Ozcelik T, Francke U, et al. (1992) Maternal imprinting of the mouse Snrpn gene and conserved linkage homology with the human Prader-Willi syndrome region. Nat Genet 2: 259-264.

3. Smith RJ, Dean W, Konfortova G, Kelsey G (2003) Identification of novel imprinted genes in a genome-wide screen for maternal methylation. Genome Res 13: 558-569.

4. Choi JD, Underkoffler LA, Wood AJ, Collins JN, Williams PT, et al. (2005) A novel variant of Inpp5f is imprinted in brain, and its expression is correlated with differential methylation of an internal CpG island. Mol Cell Biol 25: 5514-5522.

5. Piras G, El Kharroubi A, Kozlov S, Escalante-Alcalde D, Hernandez L, et al. (2000) Zac1 (Lot1), a potential tumor suppressor gene, and the gene for epsilon-sarcoglycan are maternally imprinted genes: identification by a subtractive screen of novel uniparental fibroblast lines. Mol Cell Biol 20: 3308-3315.

6. Plass C, Shibata H, Kalcheva I, Mullins L, Kotelevtseva N, et al. (1996) Identification of Grf1 on mouse chromosome 9 as an imprinted gene by RLGS-M. Nat Genet 14: 106-109.

7. Hagiwara Y, Hirai M, Nishiyama K, Kanazawa I, Ueda T, et al. (1997) Screening for imprinted genes by allelic message display: identification of a paternally expressed gene impact on mouse chromosome 18. Proc Natl Acad Sci U S A 94: 9249-9254.

8. Wang Y, Joh K, Masuko S, Yatsuki H, Soejima H, et al. (2004) The mouse Murr1 gene is imprinted in the adult brain, presumably due to transcriptional interference by the antisense-oriented U2af1-rs1 gene. Mol Cell Biol 24: 270-279.

9. Schmidt JV, Matteson PG, Jones BK, Guan XJ, Tilghman SM (2000) The Dlk1 and Gtl2 genes are linked and reciprocally imprinted. Genes Dev 14: 1997-2002.

10. Hemberger M, Redies C, Krause R, Oswald J, Walter J, et al. (1998) H19 and Igf2 are expressed and differentially imprinted in neuroectoderm-derived cells in the mouse brain. Dev Genes Evol 208: 393-402.

11. Hatada I, Mukai T (1995) Genomic imprinting of p57KIP2, a cyclin-dependent kinase inhibitor, in mouse. Nat Genet 11: 204-206.

12. Zhang Z, Joh K, Yatsuki H, Wang Y, Arai Y, et al. (2006) Comparative analyses of genomic imprinting and CpG island-methylation in mouse Murr1 and human MURR1 loci revealed a putative imprinting control region in mice. Gene 366: 77-86.
